# Proximal Steric Shielding Unlocks High Solid‐State Luminescence in Quinoxaline‐Fused Pyrene Heteroacenes

**DOI:** 10.1002/chem.71622

**Published:** 2026-08-25

**Authors:** Rafiq Ahamed, Sonal Patil, Madhusudan Dutta, Godi Kiran Venkata Sai, Sergey Fisher, Anil Bhadke, Rama Krishna Gamidi, Susnata Pramanik, Tomáš Šolomek, Sudhakar Gaikwad

**Affiliations:** ^1^ The Liebig Lab, Organic Chemistry Division CSIR‐National Chemical Laboratory (NCL) Pune Maharashtra India; ^2^ Department of Chemistry Indian Institute of Science Education and Research (IISER) Pune Pune India; ^3^ Van't Hoff Institute For Molecular Sciences University of Amsterdam Amsterdam Netherlands; ^4^ Department of Fibres and Textile Processing Institute of Chemical Technology (ICT) Mumbai Maharashtra India; ^5^ Department of Chemistry, Faculty of Science and Engineering SRM Institute of Science and Technology Kattankulathur Chennai India; ^6^ Yusuf Hamied Department of Chemistry University of Cambridge Cambridge UK

**Keywords:** azaacenes, hexacene, quinoxaline, solid‐state emission, steric shielding, supramolecular chemistry

## Abstract

Achieving high quantum yields in neat films of extended heteroacenes remains challenging because their large aromatic surfaces promote aggregation‐caused quenching (ACQ) and non‐radiative decay, while often limiting solubility and solution processability. Here we introduce proximal steric shielding to control solid‐state emission in quinoxaline‐fused pyrene heteroacenes. By systematically varying tri‐/tetraphenylethene and alkynylsilane substituents around the heteroacene core, we establish a clear structure–property relationship showing that solid‐state emission depends not simply on the amount of steric bulk, but on the spatial positioning of these substituents relative to the emissive π‐system. A modular synthetic route gives four soluble azaacenes with low solution PLQYs (6%–14%) but strongly enhanced emission in neat films. The optimised triPE‐TIPS derivative exhibits a PLQY of 82%, among the highest values reported for linearly fused azaacenes. Single‐crystal x‐ray analysis, Hirshfeld surface analysis, fluorescence lifetime measurements, aggregation studies, and DFT calculations collectively support a mechanism in which the matched proximal arrangement of triPE and TIPS groups suppresses close π–π contacts and reduces non‐radiative decay. Importantly, the enhanced neat‐film emission is distinct from conventional aggregation‐induced emission (AIE) behavior, instead arising from substituent‐position‐controlled molecular packing. These findings establish proximal steric shielding as a practical design strategy for suppressing solid‐state quenching in extended π‐systems.

## Graphical abstract

Proximal steric tuning of quinoxaline‐fused pyrene heteroacenes enables soluble derivatives that exhibit solid‐state photoluminescence quantum yields (PLQYs) up to 82%, surpassing previous benchmarks. This work reveals key structure–property relationships for next‐generation, processable organic emitters in advanced optoelectronic applications.

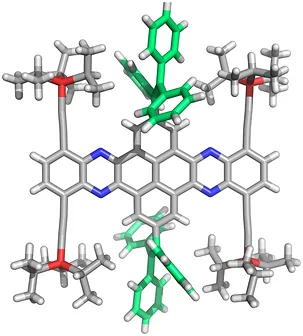

## 1 Introduction

Solid‐state organic emitters with high photoluminescence quantum yields (PLQYs) have attracted considerable attention because of their broad utility [1, 2] in photonic quantum technologies [3] and computation [4], bioimaging [5], and optoelectronic devices such as organic light‐emitting diodes (OLEDs) [6] and organic light‐emitting transistors (OLETs) [7]. When embedded in solid matrices, polymers, or thin films, organic fluorophores offer distinct advantages, including tunable optical properties, low production costs, thermal robustness, and scalability. Among these, polycyclic aromatic hydrocarbons (PAHs) such as pyrene, perylene, and rylene are widely used fluorophores [8]. Although these PAHs display strong emission in dilute solution, their PLQYs are typically quenched in the solid state due to intermolecular π–π interactions [8, 9, 10, 11], a phenomenon termed aggregation‐caused quenching (ACQ), which severely hampers their practical applications in optoelectronics and biomedical diagnostics.

To overcome ACQ, various strategies, including steric strain [12], aggregation‐induced emission (AIE) [13, 14, 15], use of heteroatoms [16, 17], macrocyclic bridging [18], polymorphism [19], steric shielding [20], and host‐guest interactions [21], have been explored to improve solid‐state emission (Figure 1). The effectiveness of bulky substituents in suppressing ACQ and enhancing solid‐state emission was elegantly demonstrated in the Würthner group's 2019 study on protein‐like enwrapped perylene bisimides [22]. However, in such systems, the chromophores are completely isolated, limiting the possibility of simultaneously achieving high PLQYs and interchromophore or chromophore‐matrix coupling. Although some of the above‐mentioned approaches improve solid‐state PLQYs, further molecular design strategies are still required to combine efficient low‐energy absorption, high solid‐state emission, and solution processability in extended π‐conjugated systems.

**FIGURE 1:**
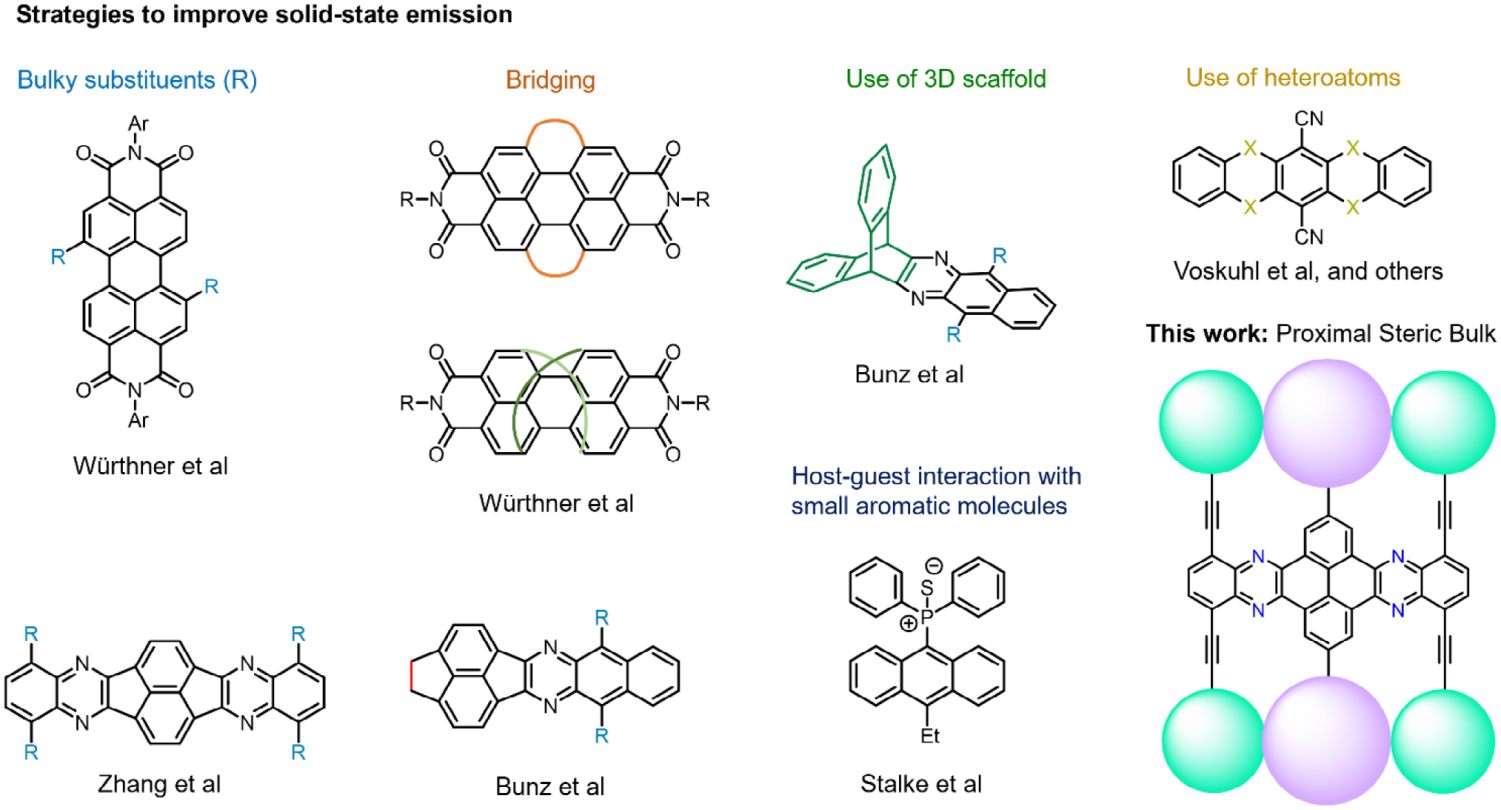
Strategies for improving solid‐state emission of PAHs and the proximal steric shielding introduced in this work.

Within this context, incorporation of heteroatoms into conjugated π‐systems offers an efficient strategy to tune the energies of the highest occupied molecular orbital (HOMO) and lowest unoccupied molecular orbital (LUMO), red‐shift absorption [16, 17, 23], and improve optoelectronic [24] and sensing properties [25]. Accordingly, *N*‐heteroacenes have emerged as promising candidates for next‐generation functional materials, particularly for optoelectronic devices [26, 27]. However, increasing the number of linearly fused aromatic rings significantly reduces their solubility due to the extended π‐surface, which promotes strong intermolecular interactions [28]. This, in turn, leads to poor solid‐state PLQYs and limited solution processability, which are key challenges for device fabrication [29]. Introducing steric bulk at the periphery of azaacenes or oligoazaacenes can improve solubility [30] and air stability. [31, 32, 33] Several studies have shown that even long or bulky alkyl/aryl or twisted groups on pyrene, quinoxalinophenanthrophenazine (QPP), and related heteroacene cores often fail to sufficiently disrupt intermolecular interactions [34, 35]. As a result, aggregation persists, leading to quenched [35, 36, 37, 38, 39, 40] or significantly diminished PLQYs [33, 37, 41, 42]. To the best of our knowledge, there is currently no solid‐state emitter based on pyrene‐fused azaacenes in neat films that emits light beyond 528 nm with a PLQY higher than 16% [23]. Consequently, the development of conjugated systems that absorb in the lower‐energy region of the solar spectrum while maintaining high solid‐state PLQYs remains a challenge for achieving improved material stability and device performance.

To address this challenge, we aimed to develop a strategy based on proximal steric engineering, in which bulky substituents are positioned in close proximity around the hexaacene backbone (Figure 2). The steric environment is tuned by a combination of TMS‐ or TIPS‐ethynyl groups with tri‐ and tetraphenylethene (TPE) substituents. The TPE groups are installed at the 2,7‐positions of the pyrene core, whereas the alkynylsilane substituents are placed on the phenyl groups of the quinoxaline moiety. This steric shielding is expected to impede cofacial π–π interactions and restrict intramolecular rotations, which could suppress non‐radiative decay and enable high solid‑state PLQYs while maintaining solubility in organic solvents. Guided by this design principle, we report the modular synthesis and characterisation of quinoxaline‐fused pyrene heteroacenes (QPHAs) functionalised with TPE and alkynylsilane substituents (Figure 2). Notably, a TPE‐PHA derivative achieves a remarkable PLQY of 82% in a neat film. This value substantially exceeds those previously reported for pyrene‐ (Φ = 16%) [23], iptycene‐ (Φ = 6%‒17%) [43, 44], dibenzobarrelene‐ (Φ = 24%) [45], acenaphthylene‐ (Φ = 30%) [46], and indaceno‐fused azaacenes (Φ = 30%) [47]. This study on QPHAs shows that proximal placement of triPE and TIPS groups is more important than steric bulk alone for suppressing non‐radiative decay. A comparison of selected solid‐state PLQYs for linearly fused azaacenes measured in neat films is provided in Table 1.

**FIGURE 2:**
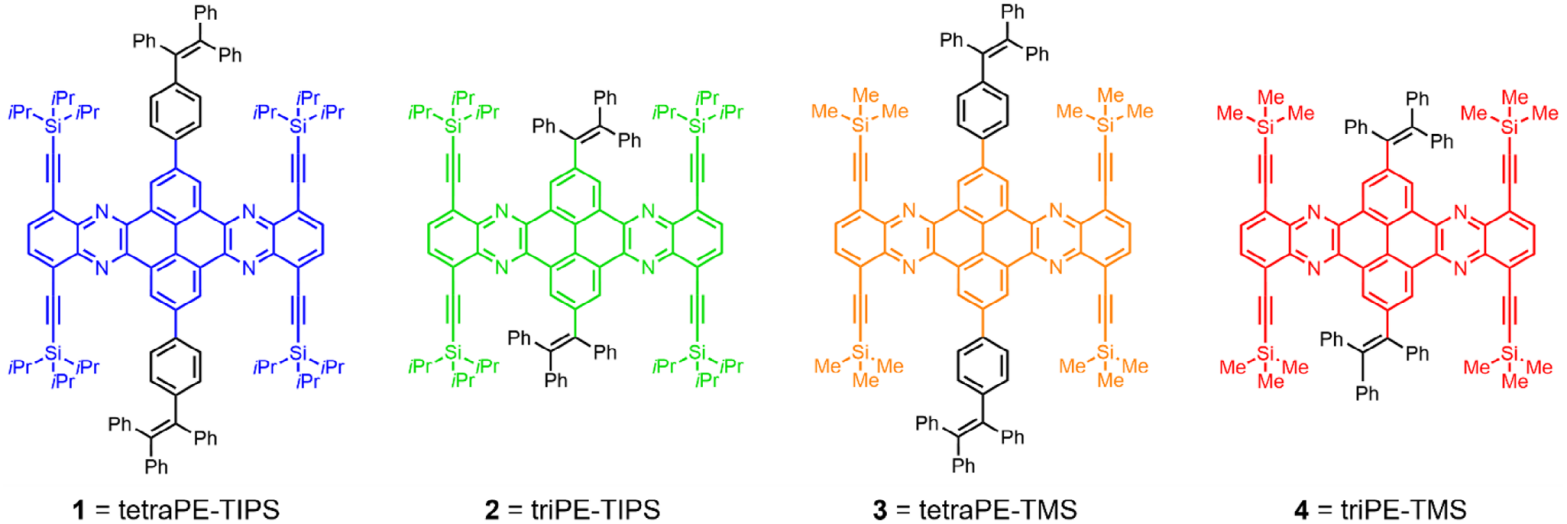
Chemical structures of tetra‐ and triphenylethylene appended quinoxaline‐fused pyrene heteroacenes (TPE‐QPHAs) **1**‒**4**.

**TABLE 1:** Reported PLQYs of selected linearly fused QPHA azaacenes.

Backbone	Strategy	Form	*λ*_*em*_	PLQY (%)	Reference
Pyrene‐QPP	Distal aryl substitution	Neat film	488	16	[23]
Iptycene‐azaacene	Rigid 3D steric blocking	Neat film	514	17	[43, 44]
Dibenzobarrelene‐azaacene	Rigid, bulky fused core	Neat film	598	24	[45]
Acenaphthylene‐azaacene	Backbone distortion	Neat film	567	30	[46]
Pyrene‐QPP (TPE‐QPHA **2**)	Proximal steric bulk	Neat film	524	**82**	**This work**

Importantly, this comparison shows that enhanced solid‐state emission is not governed simply by increasing substituent size. Instead, it depends on how close the steric groups are arranged around the heteroacene core, which determines whether close π‐contacts can exist and how radiative and nonradiative decay pathways compete.

## 2 Results and Discussion

### 2.1 Synthesis

The TPE‐functionalised quinoxaline‐fused pyrene heteroacenes **1**–**4** were synthesised via a modular synthetic route, as outlined in Scheme 1. At first, intermediates **5** and **8** were prepared (Scheme S1). Their experimental details and data are described in the Supporting Information.

**SCHEME 1:**
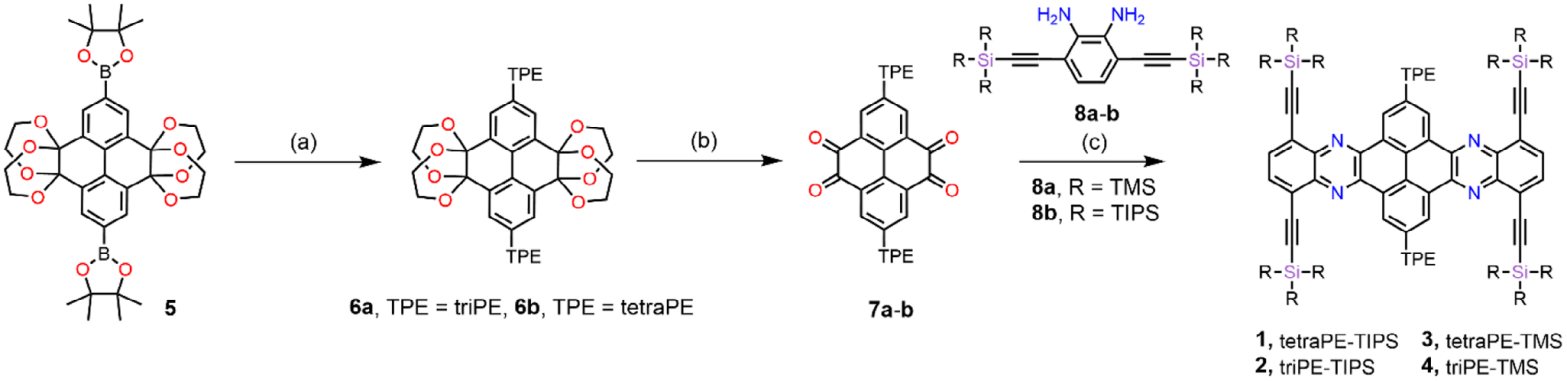
Synthesis of TPE‐QPHAs **1**−**4**: (a) triPE‐Br and tetraPE‐Br, Pd(PPh_3_)_4_, *^t^* Bu_3_P•HBF_4_, PhMe:EtOH:H_2_O (5:3:2, *v*/*v*/*v*), 85°C, 17 h, 77% (**6a**), 73% (**6b**); (b) TFA−H_2_O (9:1, *v*/*v*), 85−100°C, 12−48 h, 97% (**7a**), 97% (**7b**), and (c) **8a**−**b**, CHCl_3_─AcOH (5:1, *v*/*v*), 70°C, 12−15 h, 92% (**1**), 87% (**2**); 89% (**3**), 88% (**4**).

Suzuki‐Miyaura cross‐coupling reaction of **5** with 2‐bromo‐1,1,2‐triphenylethylene (triPE‐Br) and 4‐bromophenyl‐tetraphenylethylene (tetraPE‐Br) in a mixture of PhMe:EtOH:H_2_O (5:3:2, *v*/*v*/*v*), using Pd(PPh_3_)_4_/*^t^* Bu_3_P•HBF_4_ catalytic system at 85°C furnished the corresponding triPE‐tetraketal (**6a**) and tetraPE‐tetraketal (**6b**) in good yields (77% and 73%, respectively). The solid‐state structure of **6a** was further confirmed by single‐crystal x‐ray diffraction (SC‒XRD, Figures S72‒S73 and Table S4). Subsequent acid‐catalysed deprotection of triPE‐tetraketal (**6a**) and tetraPE‐tetraketal (**6b**) in TFA‐H_2_O mixture (9:1, *v*/*v*) gave the triPE tetraketone (**7a**) and tetraPE tetraketone (**7b**) in good yields (97% each). Subsequent cyclocondensation of tetraketones **7a** and **7b** with diamines **8a** and **8b** in CHCl_3_─AcOH mixture (5:1, *v*/*v*) at 70°C furnished the target TPE‐QPHAs **1**−**4** as yellow to pale‐yellow powders in excellent yields (87−92%). All intermediates and final compounds were fully characterised by IR, NMR, and HR−MS (see Figures S1‒S54). Compounds **1**−**4** displayed excellent solubility in common organic solvents such as benzene, toluene, chloroform, dichloromethane, ethyl acetate, and tetrahydrofuran, which is critical for solution‐processable technologies. Solubility studies were carried out in CHCl_3_ and CH_2_Cl_2_. The TIPS‐substituted derivatives tetraPE‐TIPS (**1**) and triPE‐TIPS (**2**) showed solubilities between 16 and 21 mg mL^−1^, whereas the TMS‐substituted analogues tetraPE‐TMS (**3**) and triPE‐TMS (**4**) showed relatively moderate solubility, with values between 9 and 14 mg mL^−^^1^. These results show that the TPE‐QPHAs retain good solubility in chlorinated organic solvents despite their extended π‐conjugated heteroacene cores. The electrochemical properties of compounds **1**−**4** were investigated using cyclic voltammetry (CV) in degassed CH_2_Cl_2_, with *n*‐Bu_4_NPF_6_ as the supporting electrolyte and ferrocene/ferrocenium (Fc/Fc^+^) as the internal standard. All four compounds **1**−**4** exhibit two reversible reduction processes with half‐wave potentials between −1.33 and −1.18 V, and *E* ^II^_1/2_ = −1.57 and −1.29 V, respectively, along with additional irreversible peak potentials (*E*_pc_) in the range of −1.93 to −1.48 V (Figures S68‒S71). Their values are consistent with those obtained by DFT calculations (Table S11) and are comparable to that of reported pyrene‐fused heteroacene derivatives [42, 48, 49].

No oxidation waves were observed on the oxidative scans, consistent with their deep‐lying HOMO levels. Overall, the redox potentials reveal subtle differences across the series, since alkynylsilane substitution at the pyrene and QPP phenyl positions involves structurally and electronically similar TPE groups, resulting in minor orbital tuning. This allows the solubility and luminescence properties to be tuned without substantially altering the intrinsic electronic properties of the hexacene scaffold. The redox potentials are summarised in Supporting Information. The HOMO levels of **1** (−6.20 eV) and **3** (−6.25 eV) are nearly identical, while **2** (−6.23 eV) lies slightly above **4** (−6.35 eV). LUMO levels, estimated from the onsets of the first reduction waves, highlight the minimal impact of the alkynylsilane substituents: **2** (−3.52 eV) and **4** (−3.62 eV) are only marginally lower than **1** (−3.47 eV) and **3** (−3.53 eV).

### 2.2 Single‐Crystal X‐ray Analysis

Crystals of compounds **2** and **3** suitable for SC−XRD were obtained by slow diffusion of ethanol vapour into toluene solutions of **2** and **3** (1–2 mM) at room temperature over two weeks. Compound **2** (triPE‐TIPS) crystallises in the triclinic space group *P–1* with six molecules of both **2** and toluene, per unit cell: a = 12.33 (2) Å, b = 15.31 (3) Å, c = 33.15 (6) Å, α = 99.42 (7)°, β = 96.65 (6)°, γ = 133.36 (7)° (Figure 3a and S74‒S75 and Table S5). The SC−XRD analysis reveals a twisted hexacene core (*θ* = 5.65°), arising from steric repulsion between the closely spaced TIPS and triPE substituents. Notably, the incorporation of these sterically demanding groups in **2** achieves its intended purpose: no direct intermolecular π–π interactions are observed between neighboring hexacene units, as the bulky triPE and TIPS substituents enforce a large centroid–centroid separation (*d* = 10.59 Å, Figure 3b). Therefore, the proximal steric tuning spatially isolates the molecules of **2** in the solid state by preventing these groups from relaxing to maximise crystal lattice energy. This restriction prevents π–π stacking of the hexacene scaffold, demonstrating the effectiveness of the proximal steric bulk strategy in controlling molecular packing and suppressing intermolecular interactions in the crystalline state. Compound **2** crystallises as a toluene solvate, which can influence the observed packing arrangement. However, the SC−XRD structure clearly shows that the hexaacene core is effectively shielded by proximal bulky substituents, preventing close cofacial π–π stacking. This steric blocking arises from the molecular design itself and is therefore expected to persist even in the absence of lattice solvent. While inclusion of toluene may affect secondary packing features, it is unlikely to alter this key suppression of π–π interactions. Accordingly, the crystal structure is used to explain intrinsic steric shielding and packing tendencies, while recognising that thin‐film morphologies may differ due to solvent loss and kinetic effects during film formation.

**FIGURE 3:**
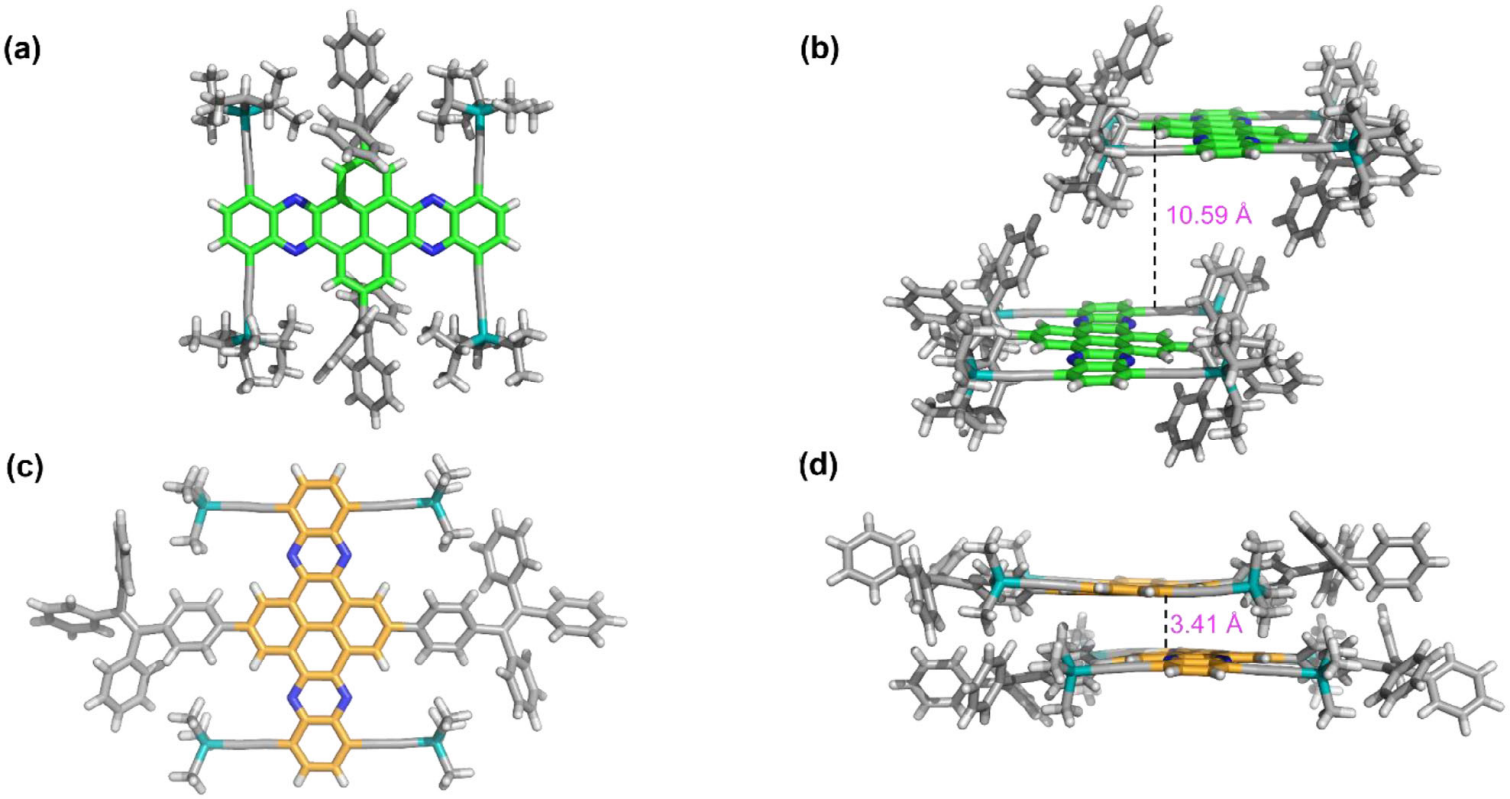
Crystal structures of compounds (a) **2** and (c) **3**, and intermolecular distances in the crystal structures of (b) **2** and (d) **3**.

Compound **3** (tetraPE‐TMS) adopts a distinctly different packing mode (Figures 3c,d and S76‒77). TetraPE‐TMS crystallises in the triclinic space group *P‒1*, but with two molecules of **3** and four toluene molecules per unit cell: a = 12.11 (15) Å, b = 16.27 (3) Å, c = 25.21 (4) Å, α = 72.89 (7)°, β = 80.52 (9)°, γ = 89.06 (11)° (Table S6). In **3**, the additional phenyl rings at the 2,7‐positions of the pyrene core and the smaller TMS substituents on the QPP scaffold, reduce proximal steric congestion, leading to outward bending of the hexacene backbone. This distortion allows slipped π–π stacked dimers with an interplanar distance of 3.41 Å (Figure 3d). In contrast, bulky TIPS and triPE substituents in **2** favour molecular isolation and effectively disrupt π–π interactions. Therefore, these characteristic packing arrangements of TPE‐QPHA derivatives, resulting from variations in proximal steric shielding, are expected to have a profound effect on their solid‐state emission. These distinct packing patterns provide an initial structural basis for understanding the different solid‐state emission behaviours of **2** and **3**, which are discussed further below.

### 2.3 Photophysical Properties of TPE‐QPHAs

The photophysical properties of compounds **1**–**4** were studied using UV−vis absorption and fluorescence emission spectroscopy in chloroform (*c* = 1 × 10^−5^ M, Figure 4). The UV−vis spectra of **1**−**4** display characteristic *β*  and *ρ* bands [42, 48, 50, 51, 52] typical of quinoxaline‐fused pyrene heteroacenes, spanning the UV and visible regions (Figure 4a). Although the overall absorption profiles of **1**−**4** are largely similar, triPE derivatives **2** and **4** display a bathochromic shift of ≈2 nm relative to their tetraPE analogues **1** and **3**. This modest red shift reflects subtle electronic differences between triPE‐ and tetraPE substituents, consistent with related pyrene derivatives [53], together with contributions from the alkynylsilane groups. The tetraPE acenes **1** and **3** exhibit an additional vibronic feature at 334 nm and stronger absorption around *λ*_max_ = 346 nm, reflecting extended conjugation relative to their triPE counterparts **2** and **4** (Figures S81 and S85). The optical band gaps of all TPE‐QPHAs fall within a narrow range (*E*_g_,_opt = _2.71–2.73 eV), in good agreement with the values obtained from density functional theory (DFT) calculations (Table 2 and Table S11) and electrochemical measurements.

**FIGURE 4:**
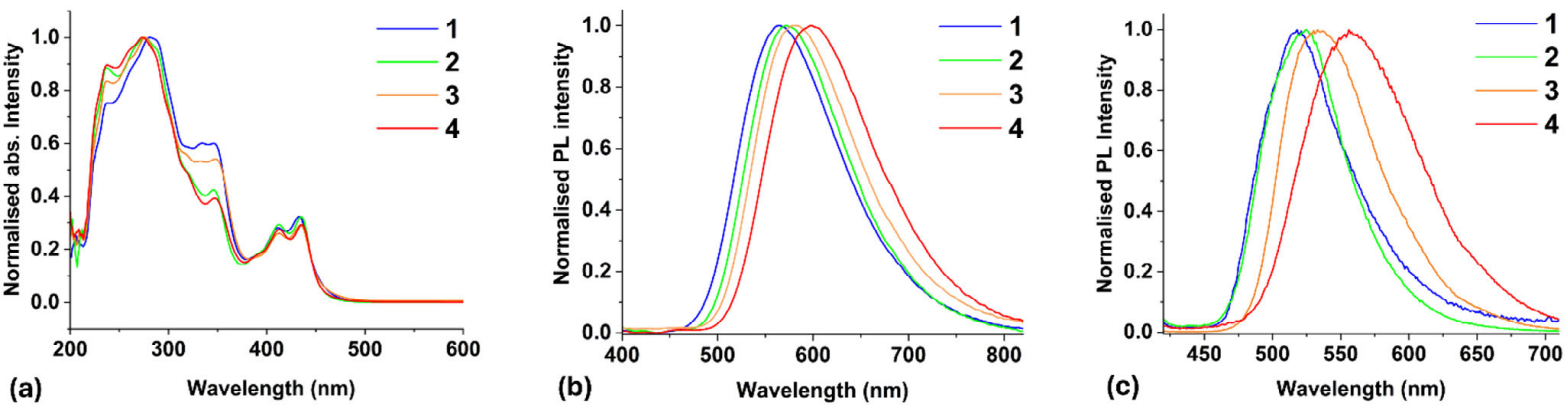
Normalised (a) UV‐vis spectra in CHCl_3_ (*c* = 1 × 10^−5^ M), (b) PL spectra in CHCl_3_ (*c* = 1 × 10^−6^ M), and (c) solid‐state (drop‐cast neat thin films) PL spectra of compounds TPE‐QPHAs **1**−**4** at 298 K.

**TABLE 2:** Optoelectronic properties of TPE‐QPHAs **1**−**4** in solution.

	*λ_ab_* (nm)^a^	*λ*_*em*_ (nm)^a^, ^b^	Φ_*sol*_ ^c^	τ*_sol_* (ns)	*k*_r_ [s^−1^]^d^	*k_n_*_r_ [s^−1^]^e^	*E_Stokes_* [cm^−1^]	*E_g, opt_* [eV]^f^	*E_g, DFT_* [eV]^g^	*E_g, CV_* [eV]^h^
**1**	433	564	0.14	9.2	1.5 × 10^7^	9.3 × 10^7^	5364	2.73	3.27	2.75
**2**	435	572	0.07	9.3	7.5 × 10^6^	1.0 × 10^8^	5506	2.73	3.27	2.76
**3**	435	581	0.10	11.9	8.4 × 10^6^	7.6 × 10^7^	5789	2.71	3.29	2.70
**4**	436	598	0.06	9.2	6.5 × 10^6^	1.0 × 10^8^	6213	2.72	3.28	2.77

^a^ Measurement in CHCl_3_ at 298 K at *c* = 1 × 10^−5^ M, absorption maximum at the longer wavelength.

^b^ Emission maximum in degassed CHCl_3_, *λ*_exc_ = 435 nm at 298 K, at *c* = 1 × 10^−6^ M.

^c^ Absolute quantum yields.

^d^ *k*_r_ = Φ/τ.

^e^ *k*_*nr*_ = (1−Φ)/τ.

^f^ Bandgap determined by the absorption onset.

^g^ Calculated by quantum‐chemical calculations.

^h^ Bandgap calculated using cyclic voltammetry in DCM.

To gain deeper insight into the geometric and electronic structures of azaacenes, DFT calculations were performed at the B3LYP/6‐31G(d) level of theory (Supporting Information, section DFT calculations). The calculated HOMO and LUMO energy levels of the TPE‐QPHAs are provided in the (Table S7–S10). For all pyrene‐fused azaacenes, the HOMOs are delocalised over the electron‐rich TPE units and extend along the *Z*‐axis of the pyrene core, whereas the LUMOs are primarily distributed over the QPP unit. The HOMO‐1 orbital of azaacenes is either entirely centred on the triPE or tetraPE units, with no density on the pyrene core. Particularly, HOMO–LUMO spatial overlap occurs in the central region of the pyrene ring. The electronic band gaps were determined to be *E_g, DFT_* = 3.27 eV for tetraPE‐TIPS (**1**), *E_g, DFT_* = 3.27 eV for triPE‐TIPS (**2**), *E_g, DFT_* = 3.29 eV for tetraPE‐TMS (**3**), and *E_g, DFT_* = 3.28 eV for triPE‐TMS (**4**). The theoretical calculations are in good agreement with the CV and optical data.

The photoluminescence (PL) spectra of **1**−**4** were recorded in degassed solution (CHCl_3_, *c* = 1 × 10^−6^ M, Figure 4b) and in drop‐cast thin films (Figure 4c). Their data are summarised in Tables 2 and 3. In dilute solution, all compounds exhibit broad, featureless emission bands extending into the near‐IR, *λ_em_* up to 820 nm, which is independent of excitation wavelength. The triPE derivatives **2** and **4** are red‐shifted compared to their tetraPE counterparts **1** and **3**. As expected, the quantum yields of all TPE‐QPHA derivatives in CHCl_3_ solution are markedly low. Specifically, tetraPE‐TIPS (**1**) shows a yellow‐green emission at *λ* ^sol^_em_ = 564 nm (τ_sol_ = 9.2 ns, sol: solution, Figure S60), with solution PLQY (Φ_sol_) of 0.14, while tetraPE‐TMS (**3**) is red‐shifted by 17 nm, (*λ* ^sol^_em_ = 581 nm, τ_sol_ = 11.9 ns, Figure S62) and exhibits a slightly lower quantum yield of 0.10. The red shift and reduced PLQY of **3** are consistent with greater conformational flexibility imparted by the smaller TMS substituents, compared to the bulky TIPS groups in **1**. Replacement of tetraPE with triPE changes the steric environment around the heteroacene core, which induces a further red shift in the emission maxima, reflecting the combined steric and electronic effects of the altered substitution pattern. In dilute CHCl_3_ solution, triPE‐TIPS (**2**) emits at *λ* ^sol^_em_ = 572 nm with a lifetime of τ_sol_ = 9.3 ns (Figure S61). Introduction of the more conformationally flexible TMS groups in **4** further redshifts the emission to 598 nm (τ_sol_ = 9.2 ns, Figure S63), indicating that increased molecular flexibility facilitates enhanced π‐conjugation and charge‐transfer. Despite these structural differences, both triPE derivatives **2** and **4** exhibit comparable PLQYs in solution with Φ_sol_ = 0.07 and Φ_sol_ = 0.06, respectively (Table S2).

**TABLE 3:** Solid‐state emission properties of TPE‐QPHAs **1**−**4** in neat thin films.

	*λ*_*film*_ (nm)^a^, ^b^	Φ_*film*_ ^c^	τ_*film*_ (ns)	*k*_*r*_ [s^−1^]^d^	*k_n_*_*r*_ [s^−1^]^e^
**1**	516	0.44	3.2	1.4 × 10^8^	1.8 × 10^8^
**2**	524	0.82	3.5	2.3 × 10^8^	5.1 × 10^7^
**3**	535	0.43	2.6	1.7 × 10^8^	2.2 × 10^8^
**4**	557	0.28	3.1	9.0 × 10^7^	2.3 × 10^8^

^a^ Measured emission spectra of neat thin films prepared by drop‐casting from CHCl_3_ solutions with a compound concentration of 1 mg/1 mL.

^b^ Emission maximum at the longer wavelength.

^c^ Absolute quantum yields measured using an integrating sphere from three independent samples.

^d^ *k*_r_ = Φ/τ.

^e^ *k*_*nr*_ = (1−Φ)/τ.

Compounds **1**–**4** display unusually large Stokes shifts, 5364, 5506, 5789, and 6213 cm^−1^, respectively, significantly greater than those of 3D counterparts, that is, triptycene‐fused pyrene‐fused *N*‐heteroacenes [54], and planar conjugated quinoxaline‐fused pyrene heteroacenes [35, 55, 56]. This effect results from the participation of the TPE molecular orbitals in the electronic transitions, giving rise to charge‐transfer character from the TPE moieties to the hexacene core. To investigate the influence of solvent polarity on their optical properties, the emission spectra were measured in six different solvents (Figures 5a and S55‒S59). While the absorption maxima remained relatively unchanged with increasing solvent polarity, the emission maxima substantially span from 514 to 622 nm (Table S1). In nonpolar solvents such as hexane and toluene, the shifts are markedly smaller, which supports the charge‐transfer character from the TPE to acene core. Upon increasing solvent polarity, the pronounced increase in Stokes shifts across all TPE‐QPHA compounds indicated stabilisation of the excited states in a polar environment, consistent with the predominant charge‐transfer nature of the emissions, as further supported by DFT calculations. Analysis of the natural transition orbitals (NTOs) for the S_0_ → S_1_ transition further validates the charge‐transfer character of the excitation: with the hole density predominantly localised on the TPE units and the electron density delocalised over the QPP moiety (Figures 5b and S78, S80, S82, S84). This spatial separation of frontier orbital contributions provides clear evidence for the intramolecular charge‐transfer nature of the lowest excited state, consistent with the pronounced solvatochromic response observed in the PL spectra of **1**–**4**.

**FIGURE 5:**
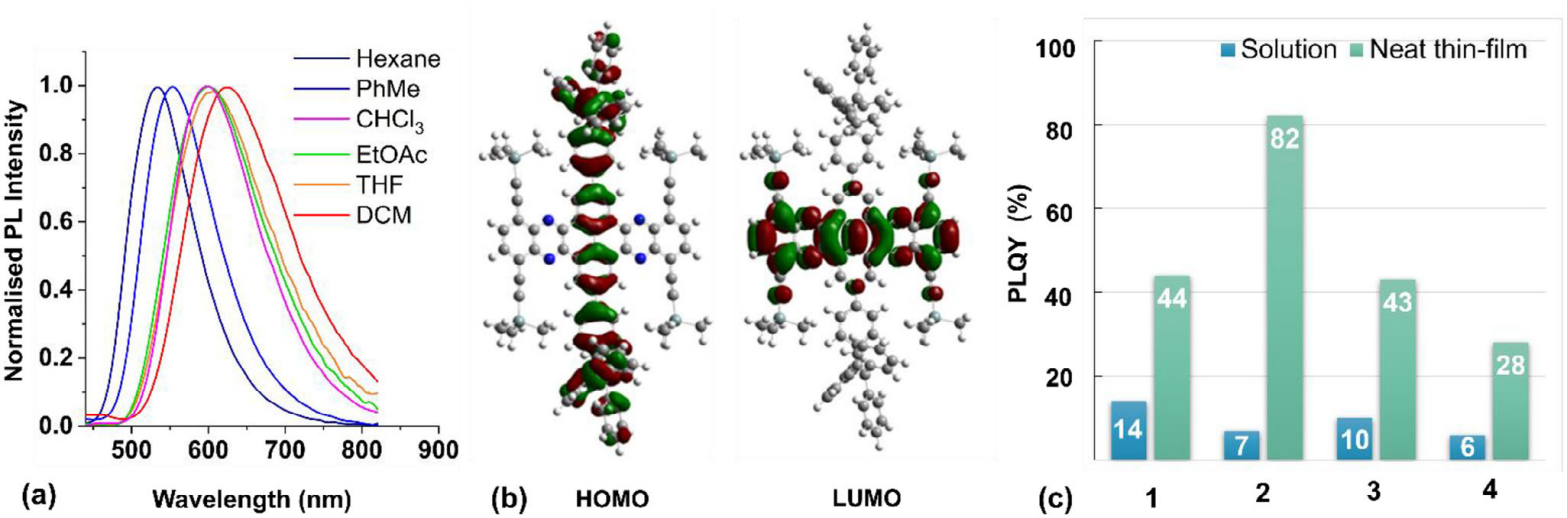
(a) Solvatochromism of **3** in different solvents at 298 K. (b) Natural transition orbitals (NTOs) and occupancies of the S_0_→S_1_ transition in **3**. (c) Comparison of solution, and solid‐state PLQYs of compounds **1**‒**4** in neat films.

### 2.4 Aggregation Behaviour of TPE‐QPHAs

AIE is a common strategy for enhancing the solid‐state emission of organic luminogens through restriction of intramolecular rotations and vibrations in the aggregated state. Given the presence of triPE‐ and tetraPE moieties in TPE‐QPHAs, we investigated whether the enhanced solid‐state emission observed for compounds **1**–**4** arises from a conventional AIE mechanism. Their photophysical properties were therefore examined in THF/water binary mixtures with water fractions ranging from 0% to 90% (Figures 6a–d and S86). The absorption spectra of TPE‐QPHAs show two characteristic structured bands: a lower‐energy band between 400 and 450 nm attributed to the S_0_→S_1_ transition and a higher‐energy band centred around 350 nm corresponding to the S_0_→S_2_ transition. Upon increasing the water content from 0% to 90%, only small bathochromic shifts were observed (≈10 nm for the tetraPE derivatives **1** and **3** (Figure S86a–b), and ≈3 nm for the triPE derivatives **2** and **4** (Figure S86c–d), indicating that aggregation has only a minor influence on the ground‐state electronic structures of these luminogens. In contrast, the emission behaviour shows a pronounced dependence on solvent composition. For the tetraPE derivatives **1** and **3**, the photoluminescence intensity decreases sharply as the water fraction increases from 0% to 30%, followed by a gradual recovery at higher water contents. The emission intensity reaches values comparable to those observed in pure THF at approximately 70–80% water fraction before decreasing slightly at 90% water content. A similar trend is observed for the triPE derivatives **2** and **4**, although the initial quenching persists up to approximately 50% water fraction before emission recovery occurs. Notably, for all four compounds, the recovered emission intensity remains comparable to that observed in pure THF solution (Figure 6) and does not exceed it substantially.

**FIGURE 6:**
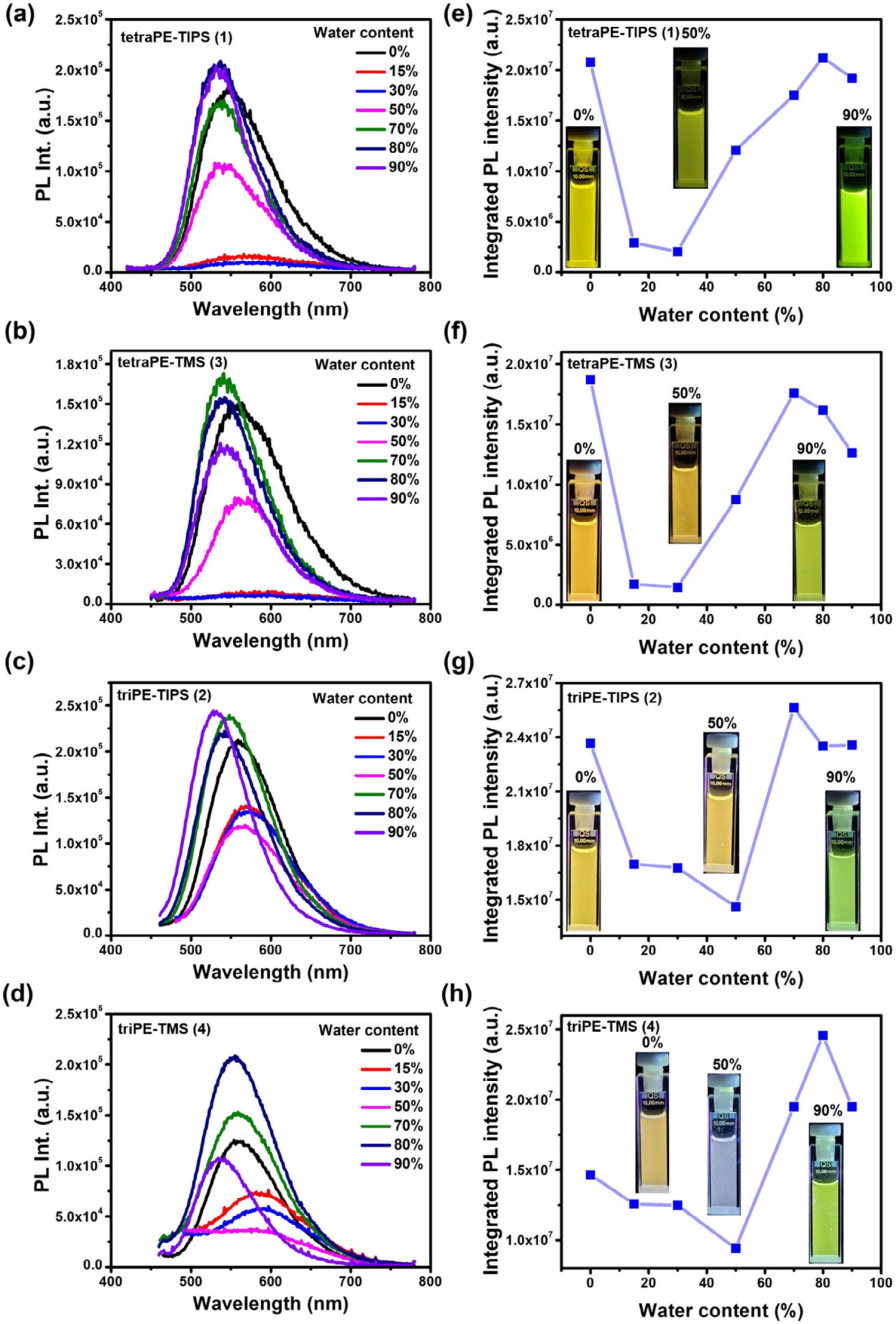
PL spectra of **1**–**4** in THF/water binary mixtures at 298 K and their variation of integrated PL intensity with water content. (a, e) tetraPE‐TIPS (**1**), (b,f) tetraPE‐TMS (**3**), (c,g) triPE‐TIPS (**2**), and (d,h) triPE‐TMS (**4**). Inset: Cuvette pictures of 0%, 50% and 90% THF/water mixtures, taken under 365 nm UV irradiation.

Time‐resolved fluorescence measurements further support this interpretation. In pure THF, these azaacenes exhibit relatively long fluorescence lifetimes of approximately 10–12 ns. Upon increasing the water content to 90%, the average lifetime decreases substantially to approximately 3–5 ns (Figure S86 e–h and Table S12). Such lifetime shortening indicates the emergence of additional non‐radiative deactivation pathways upon aggregation rather than the efficient excited‐state rigidification typically associated with classical AIE luminogens.

These observations confirm that the enhanced neat‐film emission of TPE‐QPHAs cannot be explained by conventional AIE behavior alone. In the THF/water mixtures, aggregation opens additional non‐radiative decay pathways, as reflected by the shortened fluorescence lifetimes. In contrast, the high PLQYs observed in neat films arise from a more favourable solid‐state arrangement, in which the proximal substituent arrangements suppress close π–π contacts and reduce non‐radiative decay.

Having established that the aggregation behaviour is not adequately described by a conventional AIE mechanism, we next analysed the neat‐film photophysical properties of TPE‐QPHAs. All TPE‐QPHAs show the expected, blue‐shifted emission in neat thin films, consistent with the lower dielectric environment of the solid state (Figure 4c). Among tetraPE derivatives, **1** emits at *λ* ^film^_em_ = 516 nm (τ_film_ = 3.2 ns, Figure S64), whereas **3** is red‐shifted by 19 nm to *λ* ^film^_em_ = 535 nm, with τ_film_ = 2.6 ns (Figure S66). Unlike in solution, the PLQYs of **1** and **3** increase markedly in thin films (Φ_film_ = 0.44 and Φ_film_ = 0.43), an improvement attributed to the restriction of intramolecular motions in the neat thin film that suppresses non‐radiative decay (Table 3).

The solid‐state PL spectra of triPE derivatives **2** and **4** follow trends analogous to those of **1** and **3**. Compound **2** displays an intense emission at *λ* ^film^_em_ = 524 nm with a lifetime of τ_film_ = 3.5 ns (Figure S65) and a solid‐state quantum yield of Φ_film_ = 0.82 (Figure 5c), placing it among the highest values reported for linearly fused azaacene derivatives. In contrast, compound **4** emits at *λ* ^film^_em_ = 557 nm with a comparable lifetime of τ_film_ = 3.1 ns (Figure S67) but substantially lower quantum yield of Φ_film_ = 0.28. This difference is primarily reflected in the non‐radiative decay rates. Compound **2** exhibits the lowest *k*_*nr*_ value among the series, whereas compound **4** displays the highest *k*_*nr*_ value. Compound **2** also shows a relatively high radiative rate constant, indicating that its superior solid‐state emission arises from both efficient radiative decay and suppression of non‐radiative relaxation enabled by its proximal steric environment. As shown by the crystal structure of **2**, the combined steric demand of the triPE and TIPS substituents effectively prevents close cofacial stacking of the hexacene backbones, leading to molecular isolation and a large centroid–centroid separation of 10.59 Å (Figure 3b). This packing arrangement provides a structural basis for the suppressed non‐radiative decay observed in neat films. Importantly, these results corroborate that the high PLQY of **2** is not governed by steric bulk alone, but by the precise proximal placement of the triPE and TIPS substituents around the emissive heteroacene core. In addition, several intermolecular C─H···π contacts involving the triPE and TIPS phenyl groups may contribute to local rigidification of the peripheral substituents, thereby limiting intramolecular motions that otherwise promote non‐radiative relaxation.

In contrast, the smaller TMS substituents in **4** provide reduced proximal shielding of the hexacene backbone, allowing greater conformational flexibility and more favourable intermolecular contacts between the hexacene moieties. These effects increase non‐radiative decay and result in a substantially lower PLQY (Φ_film_ = 0.28), despite the presence of similar chromophoric moieties. Kinetic analysis further supports this interpretation. Compound **4** exhibits the highest non‐radiative rate constant among the series, indicating that the decrease in PLQY is primarily governed by an increase in *k*_*nr*_, rather than by changes in *k*_*r*_. The moderately high PLQYs of **1** and **3** indicate that, despite the differences between the TIPS and TMS substituents, both derivatives retain partial conformational rigidity in their neat thin films. However, their lower efficiencies relative to triPE‐TIPS derivative **2** suggest that steric bulk alone is insufficient to maximise solid‐state emission. In particular, the reduced proximal steric hindrance in **1** could allow closer molecular packing, whereas **3** retains slipped π–π stacked dimers between neighboring hexacene backbones with an interplanar distance of 3.41 Å (Figures 3d and S76–S77). Compounds **1** and **3** show comparable decay behavior, consistent with their similar *k_r_* and *k_nr_* values (Table 3). Thus, their moderately high but lower PLQYs relative to **2** are consistent with only partial suppression of intermolecular interactions.

Although the single‐crystal structures reveal distinct packing arrangements and different degrees of π‐surface isolation, Hirshfeld surface analysis was used to quantify the relative contributions of close intermolecular contacts in **2** and **3**. The analysis was performed using CrystalExplorer [57, 58]. The C···C contact contribution decreases from 5.6% in tetraPE‐TMS derivative **3** to 2.7% in triPE‐TIPS derivative **2**. Although some C···C contacts in **2** may involve co‐crystallised toluene molecules, **2** does not display direct π–π stacking between neighboring hexacene backbones. In contrast, **3** forms slipped π–π stacked dimers with an interplanar separation of 3.41 Å. Thus, the lower C···C contribution in **2** can be interpreted as a reduced tendency for direct hexacene‐hexacene π‐stacking, rather than the complete absence of π–π interactions. This is consistent with more effective shielding of the hexacene core by the proximally placed triPE and TIPS substituents. Other non‐covalent interactions follow the same trend. The C···H contacts decrease from 25.3% in **3** to 21.2% in **2**, while C···N contacts decrease from 1.0% to 0.5%. Similarly, H···N/N···H contacts are reduced from 2.3% in **3** to 1.5% in **2** (Figures S87, S88). Together, these values show that the proximal steric environment in **2** limits not only π‐ π interactions but also other close contacts around the heteroacene framework. In contrast, the smaller TMS substituents in **3** provide less effective steric shielding of the hexacene core, allowing closer intermolecular interactions and residual π–π stacking. Thus, the Hirshfeld analysis provides quantitative support for the proposed proximal steric control mechanism. Although this analysis is based on single‐crystal structures rather than neat films, it provides molecular‐level insight into the intrinsic packing tendencies of **2** and **3**. When considered together with the crystallographic packing analysis, the Hirshfeld data show that aromatic interactions in **2** do not develop into direct hexacene‐hexacene stacking, whereas **3** forms slipped π–π stacked dimers. Therefore, the enhanced solid‐state emission of **2** is not governed simply by the number of bulky substituents, but by their spatial placement around the heteroacene core. This reduced close‐contact environment provides a structural rationale for the higher neat‐film PLQY of triPE‐TIPS derivative **2** compared with tetraPE‐TMS derivative **3**. Overall, the decrease in solid‐state PLQY from **2** to **1**/**3** and then to **4** primarily follows the increase in non‐radiative rate constants, with *k*_nr_ ​ increasing in the order **2 **< **1** ≈ **3 **< **4**. The observed non‐monotonic dependence of solid‐state PLQY on steric substitution demonstrates that suppression of non‐radiative decay correlates with steric proximity‐controlled molecular packing, rather than steric bulk alone.

## 3 Conclusion

In conclusion, we have introduced proximal steric shielding as a molecular design strategy for enhancing solid‐state emission in extended quinoxaline‐fused pyrene heteroacenes. By systematically varying both the identity and spatial placement of tri‐/tetraphenylethene and alkynylsilane substituents, we establish a clear structure–property relationship linking proximal steric arrangement, molecular packing, and neat‐film photoluminescence. The optimised triPE‐TIPS derivative exhibits a neat‐film PLQY of 82%, placing it among the most efficient linearly fused azaacene emitters reported to date. Aggregation studies and fluorescence lifetime measurements show that the enhanced neat‐film emission cannot be explained by conventional AIE behavior alone. Instead, the high PLQY arises from a favourable solid‐state arrangement in which proximal substituent geometry suppresses close π–π contacts and reduces non‐radiative decay. While the single‐crystal structures do not directly reproduce the morphology of drop‐cast neat films, they reveal how substituent geometry influences close‐contact formation and provide a useful molecular‐level rationale for the observed photophysical trends. Together, these results establish proximal steric shielding as a strategy for designing bright, soluble, and solution‐processable heteroacene‐based emitters.

## Author Contributions

SG designed the project and conceived the idea. RA and SP performed the synthesis and carried out spectroscopic investigations. SP and GKVS performed the scale‐up of compounds **5**‒**8** and crystallised compounds. MD carried out AIE studies and Hirshfeld surface analysis. Pyrene tetraketone was initially provided by AB. SF and TS performed DFT calculations. RKG collected and refined the single‐crystal x‐ray data of **2**, **3** and **6a**. All authors interpreted and discussed the data. SG and TS analysed the data, wrote the manuscript with input from all authors. All authors have approved the final version of the manuscript.

## Conflicts of Interest

There are no conflicts of interest to declare.

## Supporting information



The supporting data of this study can be found in the supplementary section of this article (general information, synthesis, detailed experimental procedures, analytical data, such as IR, ^1^H and ^13^C NMR spectra, HR‐MS spectra, UV‐vis, fluorescence, fluorescence lifetime, quantum yield calculations, single‐crystal x‐ray data, cyclic voltammetry and TD‐DFT calculations). The authors have cited additional references within the Supporting Information [59, 60, 61, 62]. The supplementary crystallographic data are available on CCDC and FIZ [63]. The authors have cited additional references within the Supporting Information. [59, 60, 61, 62, 63] **Supporting File**: chem71622‐sup‐0001‐SuppMat.pdf

## Data Availability

The experimental data that support the findings of this study are available from the corresponding author upon request.
