# Supplementary material for: Proximal Steric Shielding Unlocks High Solid‐State Luminescence in Quinoxaline‐Fused Pyrene Heteroacenes
Source: Chemistry. 2026 Aug 25;32(42):e71622. doi: 10.1002/chem.71622 (PMC13755133; doi:10.1002/chem.71622)
Supplement: Supplementary file 1 [file CHEM-32-e71622-s001.pdf]

Electronic Supplementary Information

Proximal Steric Shielding Unlocks High Solid-State Luminescence in Quinoxaline-Fused Pyrene Heteroacenes

Rafiq Ahamed,[a,#] Sonal A. Patil,[a,#] Madhusudan Dutta,[b] Godi Kiran Venkata Sai,[a] Sergey Fisher,[c] Anil Bhadke,[d] Rama Krishna Gamidi,[a] Susnata Pramanik,[e] Tomáš Šolomek[c] and Sudhakar Gaikwad*[a,f]

Table of Contents

1. Synthesis
   - 1.1 General information S2
   - 1.2 Synthesis of intermediates and final compounds S2
2. ^{1}H and ^{13}C NMR Spectra S16
3. FT–IR Spectra S31
4. MALDI–TOF Spectra S39
5. UV-visible and fluorescence spectroscopy
   - 5.1 Solution-state fluorescence studies S43
   - 5.2 Lifetime measurements (solution-state) S46
   - 5.3 Quantum yield measurements (solution-state) S48
   - 5.4 Lifetime measurements (solid-state) S48
   - 5.5 Quantum yield measurements (solid-state) S50
6. Cyclic voltammetry S51
7. Single–crystal X–ray diffraction data S53
8. DFT calculations S60
9. Aggregation investigation in THF-Water binary mixtures S68
10. Hirshfeld surface analysis S69
11. References S86

1. Synthesis

1.1. General information

All reagents and chemicals were obtained from commercial suppliers (Sigma-Aldrich, TCI Chemical Industry, BLDpharm, Acros Organics, and Spectrochem). They were used without further purification. 4 Å Molecular sieves were dried at 350 °C for 3 hours and stored under an inert atmosphere. Dioxane and ethylene glycol were dried over 4 Å molecular sieves and stored under nitrogen atmosphere. Technical-grade solvents were distilled before use. Chloroform ($CHCl_3$), triethylamine (Et_3N), and dichloromethane (DCM) were distilled over calcium hydride. Tetrahydrofuran (THF) was pre-dried over basic alumina prior to distilling over sodium metal. All air- and moisture-sensitive reactions were performed under an inert atmosphere using standard Schlenk line techniques. Compounds were purified on silica gel (60−120, 100−200, or 230−400 mesh) or neutral alumina (0.05–0.15 mm, Brockmann Activity 1). Thin-layer chromatography (TLC) was performed using Merck silica gel 60 (F_{254}) or Al_2O_3 150 (F_{254}) sheets. The melting points of intermediates and final compounds were measured on a Stuart SMP10 apparatus and reported without corrections. IR spectra were recorded on a Bruker IR ATR and a Bruker ALPHA FT-IR spectrometer. Deuterated solvents were purchased from Eurisotop. ^{1}H, ^{13}C, and ^{1}H−^{1}H COSY NMR spectra were recorded on a Bruker Avance 400 MHz or Bruker 500 MHz spectrometer, using the deuterated solvent as the lock. The chemical shifts (*d*) refer to the residual protiated fraction of deuterated solvents ($CDCl_3$: δ_H = 7.26 ppm, δ_C = 77.00 ppm, CD_2Cl_2: δ_H = 5.32 ppm, δ_C = 53.80 ppm) at 298 K. In ^{1}H NMR assignments, the following abbreviations were used to describe splitting patterns: s: singlet, d: doublet, t: triplet, dd: doublet of doublet, ddd: doublet of doublets of doublet, bs: broad singlet, dt: doublet of triplets, td: triplet of doublets, m: multiplet, quin: quintet, and sext: sextet. The value of coupling constant(s) is reported in Hertz (Hz). The number of protons is implied, and interpretations of protons are mentioned wherever possible. The numbering of the carbon atoms is not in agreement with IUPAC nomenclature. HR−MS Matrix-assisted laser desorption/ionisation-time-of-flight mass (MALDI-TOF) spectra were recorded on a Bruker spectrometer using 2, 5-dihydroxybenzoic acid (DHB) as a matrix material. UV−vis absorption spectra were measured on Analytik Jena SPECORD 210 PLUS spectrometer (25 °C) using 117.100F-QS cuvettes from Hellma Analytics (10 mm path length). Excitation and emission spectra were recorded at 25 °C on the FS5 Spectrofluorometer from Edinburgh Instruments. The PL lifetimes were recorded on the FS5 Spectrofluorometer. Absolute PLQYs were measured on a Fluoromax-4 spectrofluorometer (Horiba Jobin Yvon). The solid-state PL

spectra were measured using the drop-casting method, with a compound concentration of 1 mg/1 mL in chloroform. The PL spectra were measured in the wavelength range spanning up to 825 nm (solution state) and 725 nm (solid state). Cyclic voltammetry (CV) experiments were carried out on a Metrohm Autolab multi-channel electrochemical workstation (Multi Autolab M204) equipped with a standard three-electrode setup: a Pt counter electrode, a glassy carbon working electrode, and a silver wire as the pseudoreference electrode. The compounds were dissolved in freshly distilled and degassed (dichloromethane) CH_2Cl_2. All experiments were carried out in a solution containing 0.1 M tetra-*n*-butylammonium hexafluorophosphate as a supporting electrolyte. The values are referenced against SCE using ferrocene (Fc) as an internal reference ($E_{1/2}$ vs. SCE = + 0.48 V). Single crystals were measured on a Bruker D8 VENTURE Kappa Duo PHOTON II CPAD diffractometer equipped with Incoatech multilayer mirror optics. The intensity measurements were carried out with a micro-focus sealed tube diffraction source (MoK = 0.71073 Å) at 100 (2) K.

1.2: Synthesis of intermediates and final compounds

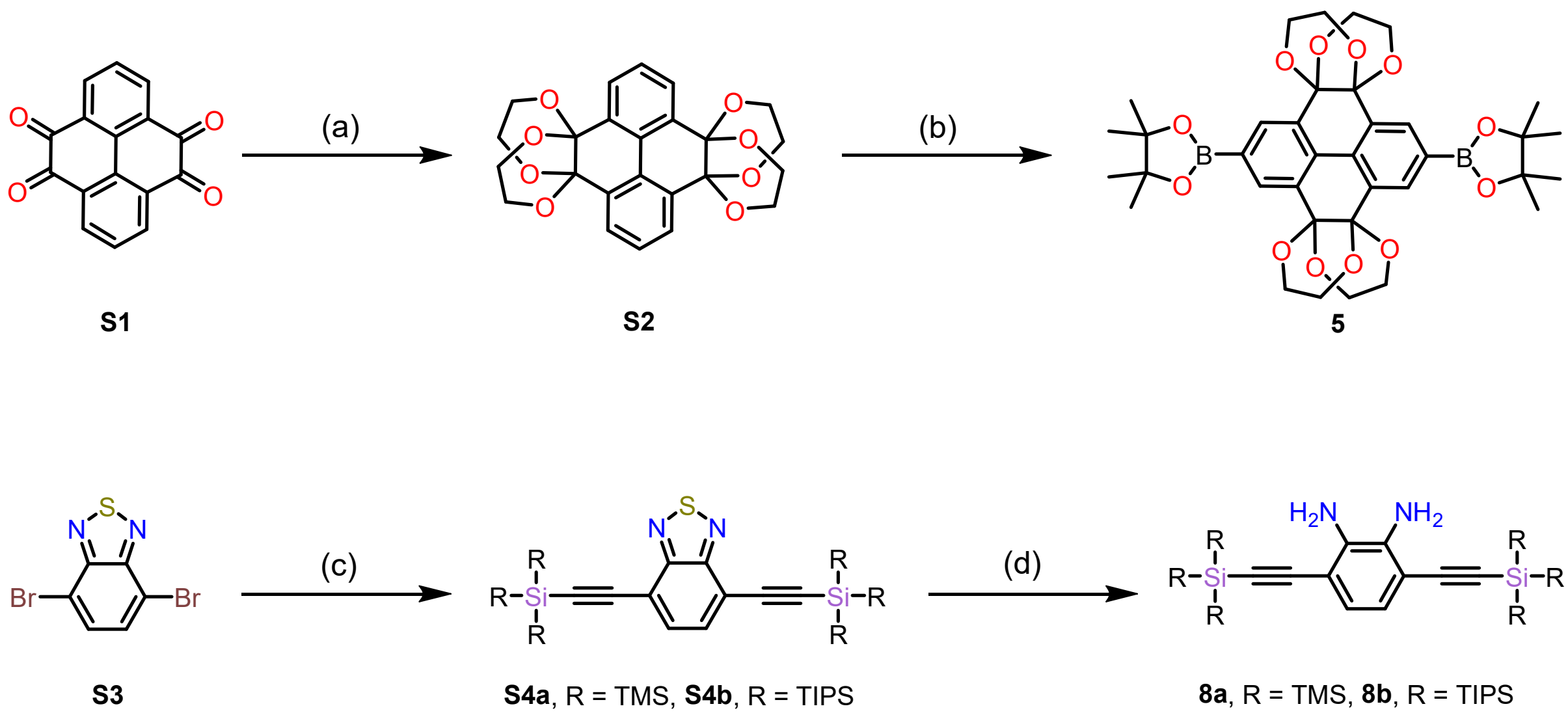

Scheme S1. (a) Ethylene glycol, (*R*)-(−)CSA, MeOH, 140 °C, 24 h, 87%; (b) B_2Pin_2, $[Ir(COD)(OMe)]_2$, di-*tert*-butyl-4,4'-dipyridine (dtbpy), anhydrous dioxane, 120 °C, 48 h, 88%; (c) TMS-acetylene and TIPS-acetylene, $PdCl_2(PPh_3)_2$, CuI, THF−Et_3N (1:1, *v*/*v*), 80 °C, 12 h, 84% (**S4a**), 88% (**S4b**); (d) $LiAlH_4$, dry THF, 0 °C, then 25 °C, 12 h, 81% (**8a**), 88% (**8b**).

Synthesis of pyrene-4,5,9,10-tetraone (S1)[1]

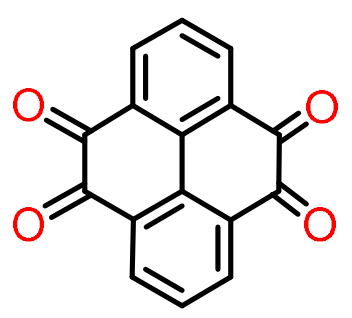

A suspension of pyrene (5.00 g, 24.72 mmol, 1.00 equiv.) in a mixture CH_2Cl_2:MeCN:H_2O (350 mL, 1.0:1.0:1.5, *v*/*v*/*v*) was cooled to 5 °C and $RuCl_3$•$x$$H_2O$ (0.50 g, 2.41 mmol, 10 mol%) was carefully added portion-wise,

over 15 minutes. After the addition of $NaIO_4$ (21.20 g, 99.11 mmol, 4.01 equiv.) in small batches over 30 minutes, the resulting dark brown mixture was allowed to stir at 35 °C for 18 hours (monitored by TLC). The reaction mixture was allowed to cool to room temperature, diluted with deionised H_2O (500 mL) and filtered through celite (~200 g). The crude residue was extracted with CH_2Cl_2 (100 mL × 2). The organic phase was sequentially washed with H_2O (200 mL) and a saturated brine solution (100 mL). The organic layers were combined, dried over anhydrous Na_2SO_4, and filtered through a cotton plug. The solvent was evaporated under reduced pressure. The crude product was purified by column chromatography on silica gel 100−200 mesh (⌀ = 5.0 cm, *l* = 25 cm) using a gradient of 50−75−100% CH_2Cl_2 in hexane (SiO_2, R_f = 0.20, 100% CH_2Cl_2), yielding compound **S1** as a bright orange solid (2.14 g, 8.16 mmol, 33%).

M.p. > 250 °C.

IR (ATR): $\tilde{\nu}$ = 3067 (*w*), 1672 (*s*), 1559 (*s*), 1450 (*m*), 1421 (*s*), 1335 (*m*), 1273 (*s*), 1174 (*m*), 1103 (*w*), 1054 (*m*), 1000 (*w*), 961 (*w*), 908 (*s*), 806 (*s*), 708 (*s*), 642 (*w*), 545 (*w*), 479 (*s*), 418 (*s*) cm^{-1}.

^{1}H NMR (DMSO-d_6, 400 MHz, 298 K): δ = 8.32 (d, ^3J = 6.5 Hz, 4H), 7.74 (t, ^3J = 6.5 Hz, 2H) ppm.

^{13}C NMR (DMSO-d_6, 100 MHz, 298 K): δ = 177.17, 134.25, 133.78, 131.55, 130.28 ppm.

Synthesis of compound S2[2]

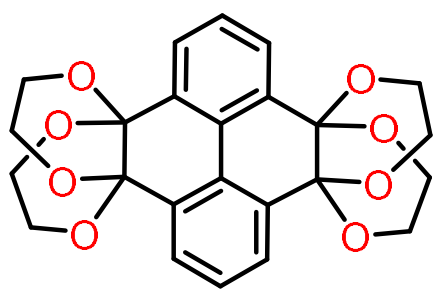

Pyrene-4,5,9,10-tetraone (**S1**, 1.00 g, 3.81 mmol, 1.00 equiv.) and (1*R*)-(–)-CSA (0.13 g, 0.56 mmol, 15 mol%) were placed in an oven-dried 100 mL sealed tube. The tube was evacuated and filled with argon (3×). Freshly degassed anhydrous MeOH (8 mL) and anhydrous ethylene glycol (4.72 g, 4.30 mL, 76.00 mmol, 20.0 equiv.) were then added to the above mixture under an inert atmosphere. The reaction mixture was further degassed for 20 minutes and heated to 120 °C for 24 h (TLC check). After cooling to room temperature, the mixture was filtered off over a sintered funnel. The residue was purified by successive washing with MeOH (10 × 2 mL) and Et_2O (20 mL × 2). The residue was dried under high vacuum to furnish compound **S2** as a pale-yellow solid (1.46 g, 3.33 mmol, 87%). R_f = 0.20 in 100% DCM.

M.p.: > 250 °C.

IR (ATR): $\tilde{\nu}$ = 2927 (*w*), 2873 (*w*), 1437 (*w*), 1282 (*w*), 1267 (*m*), 1244 (*w*), 1181 (*s*), 1080 (*s*), 1011 (*s*), 955 (*s*), 942 (*s*), 901 (*m*), 888 (*w*), 861 (*w*), 815 (*s*), 744 (*s*), 728 (*m*), 585 (*m*), 542 (*m*), 520 (*m*), 497 (*w*), 432 (*m*) cm^{-1}.

^{1}H NMR ($CDCl_3$, 400 MHz, 298 K): δ = 7.77 (d, ^3J = 7.8 Hz, 4H), 7.50 (t, ^3J = 7.8 Hz, 2H), 4.20 (bs, 8H), 3.66 (bs, 8H), ppm.

^{13}C NMR ($CDCl_3$, 100 MHz, 298 K): δ = 132.90, 129.39, 129.17, 126.97, 92.55, 61.33 ppm.

Synthesis of compound 5[3]

A mixture of pyrene tetraketal (**S2**, 1.27 g, 2.90 mmol, 1.00 equiv.) and bis(pinacolato)diboron (B_2Pin_2, 2.94 g, 11.58 mmol, 4.00 equiv.) was placed in an oven-dried sealed tube. The tube was evacuated and filled with argon (3×).

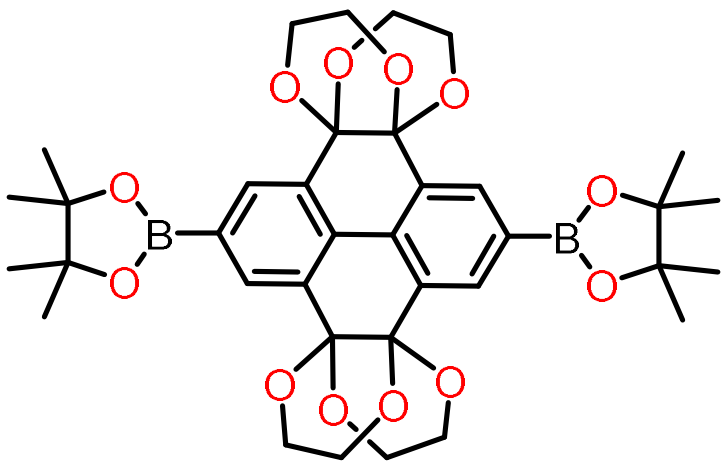

(1,5-cyclooctadiene)(methoxy)iridium(I) dimer ([Ir(OMe)(1,5-cod)]$_2$, 95 mg, 143 µmol, 5 mol%) and 4,4'-di-*tert*-butyl-2,2'-bipyridyl (77 mg, 287 µmol, 10 mol %) were added under an inert atmosphere. After the addition of pre-degassed anhydrous dioxane (30 mL), the reaction mixture was further degassed for 20 minutes and heated to 120 °C in the dark for 48 h (monitored by TLC). The mixture was then cooled to 10 °C and filtered over a sintered funnel. The residue was washed sequentially with pre-cooled MeOH (20 mL × 2) and Et_2O (30 mL × 2). The residue was dried under vacuum to afford the title compound **5**, which was an off-white powder (1.75 g, 2.55 mmol, 88%). R_f = 0.40, SiO_2, 50%EtOAc/petroleum ether (PE).

M.p.: > 250 °C.

IR (ATR): $\tilde{\nu}$ = 2975 (*w*), 2879 (*w*), 1615 (*m*), 1555 (*w*), 1470 (*m*), 1372 (*s*), 1330 (*m*), 1289 (*m*), 1248 (*m*), 1185 (*m*), 1140 (*s*), 1117 (*m*), 1095 (*s*), 1024 (*m*), 1012 (*s*), 958 (*s*), 899 (*m*), 851 (*s*), 766 (*w*), 731 (*w*), 690 (*s*), 586 (*m*), 518 (*m*) cm^{-1}.

^{1}H NMR ($CDCl_3$, 400 MHz, 298 K): δ = 8.21 (s, 4H), 4.22 (bs, 8H), 3.67 (bs, 8H), 1.32 (s, 24H) ppm. **^{13}C NMR** ($CDCl_3$, 100 MHz, 298 K): δ = 133.27, 132.38, 131.52, 126.96, 92.61, 83.88, 67.08, 24.87 ppm.

Synthesis of compound 6a

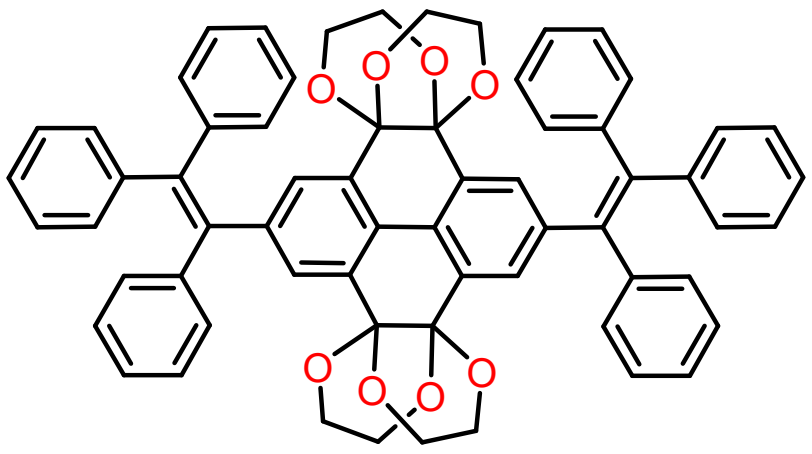

A mixture of compound **5** (100 mg, 145 µmol, 1.00 equiv.), 2-bromo-1,1,2-triphenylethylene (triPE-Br, 146 mg, 435 µmol, 3.00 equiv.) and Na_2CO_3 (154 mg, 1.45 mmol, 10.00 equiv.) were placed in a Schlenk tube. The tube was evacuated and filled with argon (3×). Then, pre-degassed PhMe (5 mL), EtOH (3 mL), and deionised H_2O (2 mL) were added. After the addition of $Pd(PPh_3)_4$ (34 mg, 29.0 µmol, 0.20 equiv.) and tri-*tert*-butylphosphonium tetrafluoroborate ($^t Bu_3HBF_4$, 8.5 mg, 29.0 µmol, 0.20 equiv.) under an inert atmosphere, the resultant mixture was stirred at 85 °C for 18 h (TLC check). The reaction mixture was diluted with deionised water (20 mL), and the crude product was extracted in CH_2Cl_2 (25 mL × 3). The combined organic layer was washed with deionised water (50 mL), followed by a saturated solution of brine (25 mL). The organic layer was separated, combined, and dried over anhydrous Na_2SO_4. The solvent was evaporated by rotary evaporation. Column chromatographic purification of crude residue on silica gel (100−200 mesh, ⌀ = 3.0 cm, l = 20 cm) using 60% CH_2Cl_2 in petroleum ether (PE) on SiO_2 (R_f = 0.5, 80% CH_2Cl_2 in PE) yielded the title compound **6a** as a light yellow solid (106 mg, 112 µmol, 77%). R_f = 0.5, 80% CH_2Cl_2 in PE.

M.p. > 250 °C.

IR (ATR): $\tilde{\nu}$ = 3021 (*w*), 2942 (*w*), 2870 (*w*), 1598 (*w*), 1575 (*w*), 1491 (*w*), 1442 (*m*), 1251 (*m*), 1179 (*s*), 1118 (*m*), 1103 (*m*), 1080 (*s*), 1062 (*s*), 1019 (*s*), 963 (*m*), 901 (*s*), 754 (*m*), 695 (*s*), 593 (*m*), 579 (*m*), 546 (*m*), 534 (*m*), 504 (*m*), 457 (*m*) cm^{-1}.

^{1}H NMR ($CDCl_3$, 400 MHz, 298 K): δ = 7.41 (s, 4H), 7.12−7.16 (m, 8H), 6.97−7.10 (m, 12H), 6.97−7.04 (m, 10H), 4.18 (bs, 4H), 3.33 (bs, 4H), 3.13 (bs, 8H) ppm.

^{13}C NMR ($CDCl_3$, 100 MHz, 298 K): δ = 145.05, 144.09, 143.79, 143.54, 141.65, 140.23, 132.10, 131.80, 131.62, 131.28, 130.37, 128.18, 127.68, 127.57, 127.26, 126.59, 126.56, 126.27, 92.50, 63.02, 58.99 ppm.

MALDI-TOF HRMS: Calcd for $C_{64}H_{50}O_8$: 946.3506; Found: 946.3840.

Synthesis of compound 6b

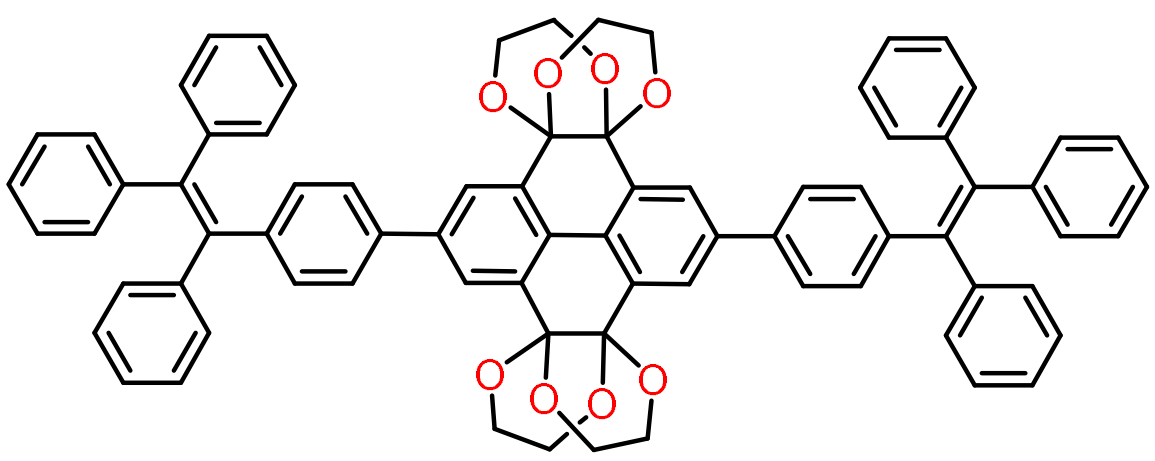

A mixture of borylated-pyrenetetraketal (**5**, 100 mg, 145 µmol, 1.00 equiv.), 1-bromo-4-(1,2,2-triphenylethenyl)benzene (tetraPE-Br, 179 mg, 435 µmol, 3.0 equiv.), and Na_2CO_3 (154 mg, 1.45 mmol, 10.00 equiv.) were placed in a

Schlenk tube. The tube was evacuated and filled with argon (3×). $Pd(PPh_3)_4$ (34 mg, 29.0 µmol, 0.20 equiv.) and tri-*tert*-butylphosphonium tetrafluoroborate ($^tBu_3HBF_4$, 8.5 mg, 29.0 µmol, 0.20 equiv.) were transferred under an inert atmosphere. After the addition of pre-degassed PhMe (5 mL), EtOH (3 mL) and deionised H_2O (2 mL), the resultant mixture was further degassed for 10 minutes and heated to 85 °C for 17 h (checked by TLC). The mixture was cooled to room temperature, diluted with deionised water (25 mL), and extracted with CH_2Cl_2 (25 mL × 3). The organic layer was successively washed with deionised water (25 mL × 3) and a saturated solution of brine (25 mL). The organic phase was combined, dried over anhydrous Na_2SO_4, and the solvent was removed in *vacuo*. The residue was subjected to column chromatographic purification on silica gel (100−200 mesh, ⌀ = 3.0 cm, l = 20 cm) using 80% CH_2Cl_2 in PE (R_f = 0.3, 80% CH_2Cl_2 in PE) to furnish **6b** as a pale yellow solid (116 mg, 106 µmol, 73%).

M.p.: > 250 °C.

IR (ATR): $\tilde{\nu}$ = 2965 (*w*), 2933 (*w*), 2871 (*w*), 1597 (*w*), 1491 (*w*), 1451 (*m*), 1262 (*m*), 1178 (*m*), 1117 (*m*), 1098 (*s*), 1014 (*m*), 960 (*s*), 914 (*m*), 852 (*m*), 829 (*w*), 750 (*m*), 698 (*s*), 622 (*w*), 593 (*w*), 517 (*w*) cm^{-1}.

^{1}H NMR ($CDCl_3$, 400 MHz, 298 K): δ = 7.96 (s, 4H), 7.45 (d, ^3J = 8.4 Hz, 4H), 7.03−7.15 (m, 34H), 4.22 (bs, 8H), 3.71 (bs, 8H) ppm.

^{13}C NMR ($CDCl_3$, 100 MHz, 298 K): δ = 143.71, 143.66, 143.60, 143.27, 141.73, 141.30, 140.40, 137.82, 133.33, 131.78, 131.40, 131.32, 131.31, 127.84, 127.78, 127.69, 126.62, 126.59, 126.51, 126.18, 125.14, 92.74, 61.14 ppm.

MALDI-TOF HRMS: Calcd for $C_{76}H_{58}O_8$: 1098.4132; Found: 1098.4260.

Synthesis of compound 7a

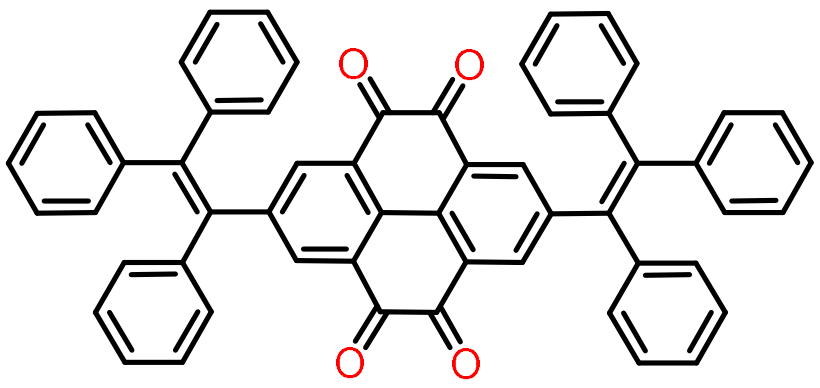

TriPE-tetraketal (**6a**, 90 mg, 95 µmol) was placed in a 50 mL single-neck round-bottomed flask. Trifluoroacetic acid (TFA, 9 mL) and deionised H_2O (1 mL) were added, and the resultant suspension was allowed to stir at 70 °C for 12 h (monitored by NMR). The reaction mixture was allowed to cool to room temperature and then diluted with deionised H_2O (50 mL). The product was extracted in CH_2Cl_2 (25 mL × 3). The organic phases were combined, dried over anhydrous Na_2SO_4, and filtered. The solvent was removed by rotary evaporation. The resultant solid was washed with MeOH (10 mL) and

vacuum dried to yield the desired triPE-tetraketone (**7a**) as a dark red solid (71 mg, 92 µmol, 97%). R_f = 0.30 in 60% DCM/PE.

M.p.: > 250 °C.

IR (ATR): $\tilde{\nu}$ = 3055 (*w*), 2924 (*w*), 2851 (*w*), 1785 (*w*), 1675 (*m*), 1600 (*w*), 1490 (*w*), 1441 (*w*), 1395 (*w*), 1345 (*w*), 1256 (*w*), 1208 (*w*), 1152 (*m*), 1073 (*w*), 1027 (*w*), 947 (*w*), 916 (*w*), 840 (*w*), 754 (*m*), 698 (*s*), 644 (*w*), 595 (*w*), 576 (*w*) cm^{-1}.

^{1}H NMR ($CDCl_3$, 400 MHz, 298 K): δ = 8.05 (s, 4H), 7.12−7.16 (m, 18H), 7.06−7.09 (m, 4H), 6.98−7.02 (m, 8H) ppm.

^{13}C NMR ($CDCl_3$, 100 MHz, 298 K): δ = 177.80, 147.36, 144.85, 142.16, 142.10, 141.25, 139.10, 137.40, 132.05, 131.14, 131.08, 130.99, 130.35, 128.38, 128.36, 127.86, 127.70, 127.54, 127.33 ppm.

MALDI-TOF HRMS: Calcd for $C_{56}H_{34}O_4{\bullet}Na^+$: 793.2349; Found: 793.2638.

Synthesis of compound 7b

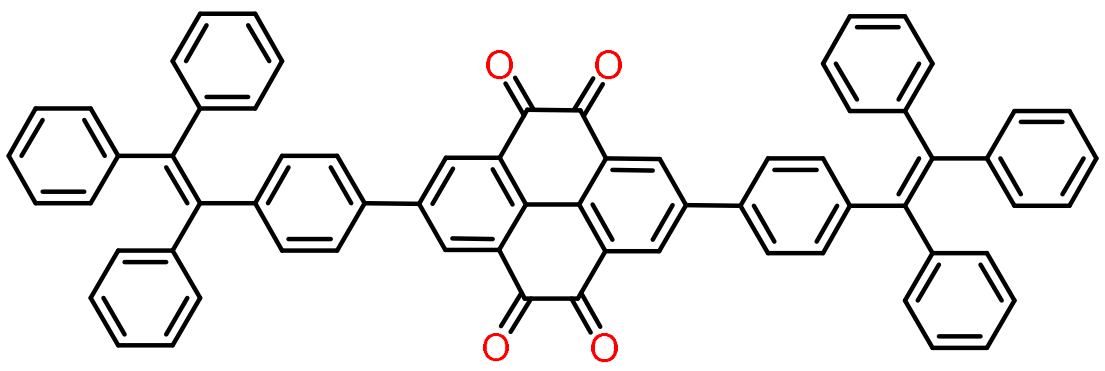

To a suspension of tetraPE-tetraketal (**6b**, 90 mg, 95 µmol) in TFA (9 mL), deionised H_2O (1 mL) was slowly added. The resultant mixture was heated at 100 °C for 48 h (checked by NMR). The reaction was allowed to cool to room temperature and diluted with deionised H_2O (50 mL). The crude product was extracted in CH_2Cl_2 (25 mL × 3). The organic layers were combined, dried over anhydrous Na_2SO_4, and filtered. The solvent was removed under reduced pressure, and the residue obtained was washed with MeOH (10 mL). The obtained residue was dried under vacuum to give the title tetraPE-tetraketone derivative (**7b**) as a red solid (79 mg, 86 µmol, 98%). R_f = 0.30 in 60% DCM/PE.

M.p.: > 250 °C.

IR (ATR): $\tilde{\nu}$ = 2922 (*w*), 1679 (*m*), 1599 (*w*), 1531 (*w*), 1491 (*w*), 1438 (*w*), 1324 (*w*), 1259 (*w*), 1205 (*w*), 1090 (*w*), 1075 (*w*), 1030 (*w*), 975 (*w*), 925 (*w*), 853 (*w*), 826 (*w*), 742 (*m*), 697 (*s*), 612 (*w*), 573 (*w*), 425 (*m*) cm^{-1}.

^{1}H NMR ($CDCl_3$, 400 MHz, 298 K): δ = 8.65 (s, 4H), 7.48 (d, ^3J = 8.5 Hz, 4H), 7.20 (d, ^3J = 8.5 Hz, 4H), 7.11−7.16 (m, 18H), 7.03−7.11 (m, 12H) ppm.

^{13}C NMR ($CDCl_3$, 100 MHz, 298 K): δ = 178.03, 145.45, 143.42, 143.36, 143.25, 142.15, 139.81, 134.49, 132.74, 132.36, 131.33, 131.30, 131.27, 131.15, 127.92, 127.84, 127.69, 126.87, 126.72, 126.68, 126.07 ppm.

MALDI-TOF HRMS: Calcd for $C_{68}H_{42}O_4$: 922.3083; Found: 922.3290.

Synthesis of compound S4a[4]

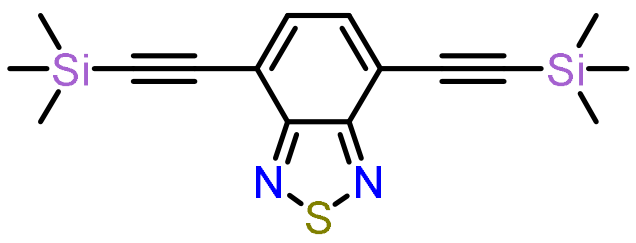

4,7-Dibromobenzo[*c*][1,2,5]thiadiazole (**S3**, 1.40 g, 4.76 mmol, 1.00 equiv.), $PdCl_2(PPh_3)_2$ (34 mg, 48 μmol, 10 mol%), and CuI (9 mg, 48 μmol, 10 mol%) were placed in a Schlenk tube. The tube was evacuated and filled with argon (3×). Then, a mixture of pre-degassed dry THF-Et_3N (1:1, *v*/*v*, 40 mL) was added. After the addition of trimethylsilylacetylene (TMSA, 1.45 g, 2.10 mL, 15 mmol, 3.10 equiv.) under an inert atmosphere, the reaction was heated to 80 °C for 12 h (TLC monitoring). The mixture was cooled to room temperature and filtered over a short pad of celite (~50 g). The solvents were removed by rotary evaporation. The residue was extracted with CH_2Cl_2 (25 mL × 3) and washed with deionised water (25 mL × 2). The organic phases were combined, dried over anhydrous Na_2SO_4 and filtered. The solvent was removed in *vacuo* and the crude product was purified by column chromatography on silica gel (100−200 mesh, ⌀ = 3.0 cm, *l* = 25 cm) using 20% DCM in PE (SiO_2, R_f = 0.30 in 10% DCM/PE) to provide the title compound **S4a** as a bright yellow solid (1.39 g, 4.23 mmol, 88%).

M.p.: 122−124 °C.

IR (ATR): $\tilde{\nu}$ = 2959 (*w*), 2901 (*w*), 2152 (*m*), 1558 (*w*), 1538 (*w*), 1490 (*w*), 1412 (*w*), 1358 (*w*), 1339 (*m*), 1247 (*m*), 1168 (*w*), 1063 (*w*), 1027 (*m*), 888 (*m*), 833 (*s*), 757 (*s*), 700 (*m*), 638 (*s*), 512 (*m*) cm^{-1}.

^{1}H NMR ($CDCl_3$, 400 MHz, 298 K): δ = 7.70 (s, 2H), 0.33 (s, 18H) ppm.

^{13}C NMR ($CDCl_3$, 100 MHz, 298 K): δ = 154.21, 133.12, 117.26, 103.63, 99.98, −0.12 ppm.

Synthesis of compound S4b[4]

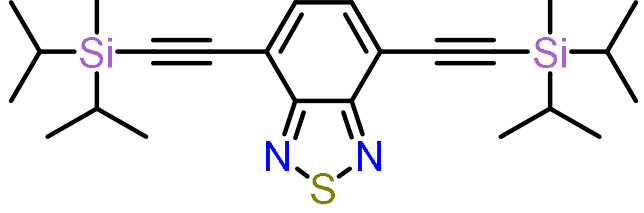

4,7-Dibromobenzo[*c*][1,2,5]thiadiazole (**S3**, 0.92 g, 3.13 mmol, 1.00 equiv.), $PdCl_2(PPh_3)_2$ (22 mg, 31 μmol, 10 mol%) and CuI (6 mg, 31 μmol, 10 mol%) were transferred into an oven-dried Schleck tube. The tube was evacuated and flushed with argon (3×). Then, a mixture of pre-degassed dry THF-Et_3N (1:1, *v*/*v*, 30 mL) and triiisopropylsilylacetylene (TIPSA, 1.77 g, 2.20 mL, 10 mmol, 3.10 equiv.) was added under argon atmosphere. The tube was sealed and stirred at

80 °C. After 12 h, the mixture was cooled to room temperature (checked by TLC) and filtered over a short pad of celite (~50 g). The residue was extracted with CH_2Cl_2 (25 mL × 3), and the organic layer was consecutively washed with deionised water (25 mL × 2) and a saturated brine solution (50 mL). The organic layers were combined, dried over anhydrous Na_2SO_4, and the solvent was removed in *vacuo*. The residue obtained was subjected to column chromatographic purification on silica gel (100−200 mesh, ⌀ = 3.0 cm, *l* = 25 cm) using 20% DCM in PE (SiO_2, R_f = 0.50 in 10% DCM/PE) to afford the desired compound **S4b** as a green solid (1.30 g, 2.62 mmol, 84%).

M.p.:106−108 °C.

IR (ATR): $\tilde{\nu}$ = 2944 (*m*), 2865 (*m*), 2152 (*w*), 1882 (*w*), 1692 (*w*), 1559 (*w*), 1539 (*w*), 1488 (*w*), 1462 (*m*), 1383 (*w*), 1359 (*w*), 1338 (*w*), 1261 (*w*), 1169 (*w*), 1073 (*w*), 1059 (*w*), 1029 (*m*), 996 (*m*), 882 (*s*), 845 (*s*), 780 (*s*), 659 (*s*), 635 (*s*), 586 (*m*), 547 (*w*), 500 (*m*) cm^{-1}.

^{1}H NMR ($CDCl_3$, 400 MHz, 298 K): δ = 7.67 (s, 2H), 1.19 (d, 36H), 1.18 (m, 6H) ppm.

^{13}C NMR ($CDCl_3$, 100 MHz, 298 K): δ = 154.64, 132.66, 117.47, 102.26, 100.26, 18.70, 11.35 ppm.

Synthesis of compound 8a[5]

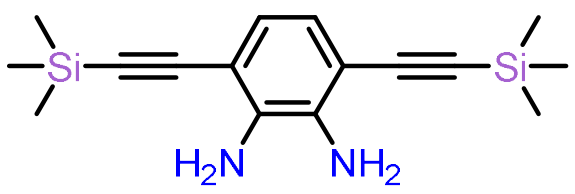

Compound **S4a** (210 mg, 0.64 mmol, 1.00 equiv.) was placed in an oven-dried round-bottomed flask. The flask was evacuated and filled with argon (3×), and freshly distilled anhydrous THF (10 mL) was added. The resultant solution was cooled to −5 °C for 10 minutes. $LiAlH_4$ (194 mg, 5.11 mmol, 8.0 equiv.) was added over 20 minutes in small portions, and the reaction mixture was stirred at −5 °C for 10 minutes. The resultant suspension was allowed to warm to room temperature and stirred at 25 °C for 12 h (TLC monitoring). The mixture was then cooled to 0 °C and cautiously quenched with a saturated solution of NH_4Cl (20 mL). The reaction was diluted with Et_2O (20 mL), subsequently washed with deionised H_2O (50 mL) and saturated brine solution (30 mL). The organic layers were combined and dried over anhydrous Na_2SO_4. After removal of solvent on rotavapor, the residue was purified by quick column chromatographic purification on silica gel (100−200 mesh, ⌀ = 3.0 cm, *l* = 15 cm) using 10% Et_2O in PE as an eluent (SiO_2, R_f = 0.30 in 10% Et_2O/PE) to furnish the desired diamine **8a** as an air-sensitive pale-yellow solid (156 mg, 519 µmol, 81%). The compound **8a** was stored at – 20 °C under an inert atmosphere.

M.p.: 139−141 °C.

IR ($CHCl_3$): $\tilde{\nu}$ = 3685 (*w*), 3429 (*w*), 3352 (*m*), 2963 (*m*), 2902 (*w*), 2404 (*w*), 2358 (*w*), 2142 (*m*), 1613 (*m*), 1526 (*w*), 1448 (*m*), 1215 (*w*), 1119 (*s*), 920 (*w*), 852 (*s*), 792 (s), 672 (m), 631 (*m*) cm^{-1}.

^{1}H NMR ($CDCl_3$, 400 MHz, 298 K): δ = 6.77 (s, 2H), 3.59 (bs, 4H), 0.27 (s, 18H) ppm.

^{13}C NMR ($CDCl_3$, 100 MHz, 298 K): δ = 136.49, 122.05, 109.59, 101.69, 100.92, 0.08 ppm.

Synthesis of compound 8b[5]

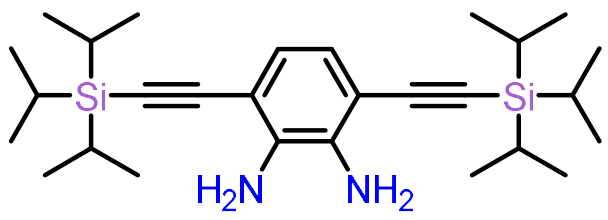

Compound **S4b** (900 mg, 1.81 mmol, 1.00 equiv.) was placed in an oven-dried round-bottomed flask. The flask was evacuated and filled with argon (3×). After the addition of freshly distilled anhydrous THF (40 mL), the solution was cooled to −5 °C for 10 minutes. Thereafter, $LiAlH_4$ (550 mg, 14.50 mmol, 8.0 equiv.) was added over 20 minutes in small portions, and the mixture was allowed to stir at −5 °C for a further 10 minutes.

The reaction mixture was warmed to 25 °C and stirred for 12 h (checked by TLC). The mixture was cooled to 0 °C using an ice-water mixture and cautiously quenched with a saturated solution of NH_4Cl (30 mL). The reaction mixture was diluted with Et_2O (40 mL). The organic phase was washed with deionised H_2O (50 mL) followed by a saturated brine solution (50 mL). The layers were separated, the combined organic phase was dried over anhydrous Na_2SO_4, filtered, and the solvent was removed in *vacuo*. The crude residue was subjected to quick column chromatographic purification on silica gel (100−200 mesh, ⌀ = 3.0 cm, *l* = 15 cm) using 10% Et_2O in PE (SiO_2, R_f = 0.30 in 10% Et_2O/PE) to yield the desired diamine **8b** as an air-sensitive pale-yellow solid (740 mg, 1.58 mmol, 87%). The compound **8b** was stored at –20 °C under an inert atmosphere.

M.p.: 134−136 °C.

IR ($CHCl_3$): $\tilde{\nu}$ = 3686 (*w*), 3427 (*w*), 3350 (*w*), 3320 (*w*), 2947 (*s*), 2869 (*s*), 2725 (*w*), 2403 (*w*), 2141 (*m*), 1611 (*m*), 1525 (*w*), 1458 (*m*), 1216 (*m*), 1120 (*w*), 1071 (*w*), 1004 (*m*), 922 (*m*), 884 (*m*), 769 (*s*), 671 (*s*) cm^{-1}.

^{1}H NMR ($CDCl_3$, 400 MHz, 298 K): δ = 6.80 (s, 2H), 3.38 (bs, 4H), 1.14 (d, 36H), 1.13 (m, 6H) ppm.

^{13}C NMR ($CDCl_3$, 100 MHz, 298 K): δ = 136.52, 122.15, 109.97, 103.67, 97.13, 18.71, 11.27 ppm.

Synthesis of compound 1 = tetraPE-TIPS:

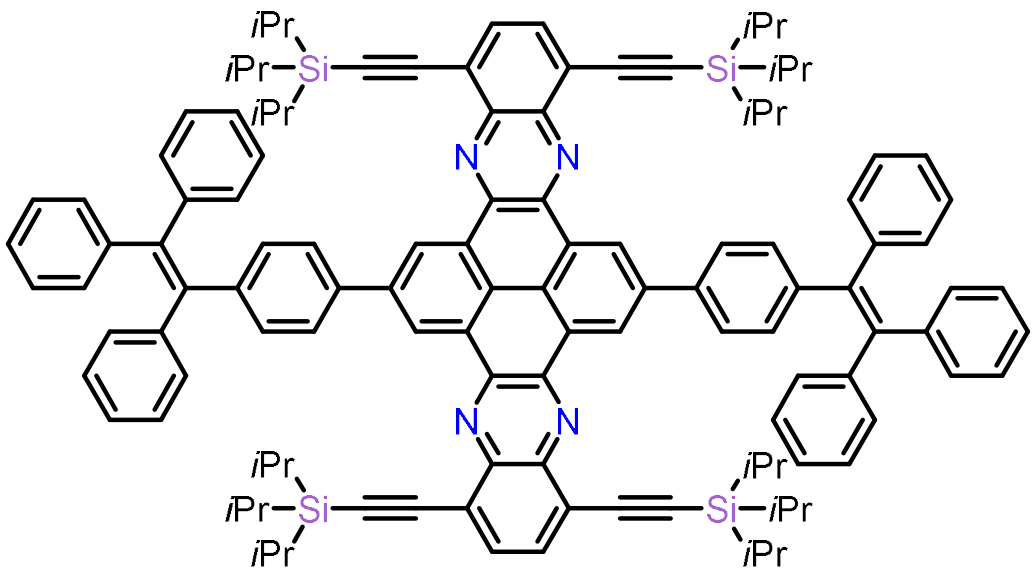

A mixture of tetraPE-tetraketone (**9b**, 43 mg, 47 µmol, 1.00 equiv.) and 3,6-bis((triisopropylsilyl)ethynyl)benzene-1,2-diamine (**12b**, 48 mg, 103 µmol, 2.20 equiv.) was placed in a 25 mL round-bottomed flask. The flask was evacuated and filled with argon (1×). Thereafter, freshly degassed $CHCl_3$ (2.5 mL) and degassed AcOH (0.5 mL) were added, and the reaction mixture was heated to 70 °C for 17 h (monitored on TLC). The solvents were removed by rotary evaporation, and the residue was co-evaporated with MeOH to remove residual AcOH. The mixture was triturated in MeOH (3 mL), filtered off over a sintered funnel, and washed with MeOH (2.5 mL). The solid was washed with MeOH (2.5 mL). The crude residue was purified by column chromatography on silica gel (230−400 mesh, ⌀ = 1.5 cm, *l* = 12 cm) using 0−30% DCM in PE (SiO_2, R_f = 0.40 in 10% DCM/PE) to give the title compound **1** as a bright yellow solid (75 mg, 42 µmol, 90%). **M.p.**: > 250 °C.

IR (ATR): $\tilde{\nu}$ = 2938 (*w*), 2862 (*w*), 2147 (*w*), 1606 (*w*), 1489 (*w*), 1461 (*m*), 1367 (*w*), 1322 (*w*), 1222 (*w*), 1134 (*m*), 1061 (*m*), 994 (*m*), 915 (*w*), 881 (*m*), 844 (*m*), 822 (*w*), 799 (*s*), 778 (*w*), 765 (*m*), 750 (*m*), 701 (*s*), 676 (*s*), 664 (*s*), 627 (*w*) cm^{-1}.

^{1}H NMR ($CDCl_3$, 400 MHz, 298 K): δ = 9.97 (s, 4H), 8.02 (s, 4H), 7.77 (d, ^3J = 8.4 Hz, 4H), 7.23 (d, ^3J = 8.4 Hz, 4H), 7.08−7.19 (m, 30H), 1.26 (m, 12H) 1.22 (d, 72H) ppm.

^{13}C NMR ($CDCl_3$, 100 MHz, 298 K): δ = 143.88, 143.83, 143.61, 143.37, 143.02, 142.30, 141.37, 140.90, 140.68, 138.69, 134.61, 131.92, 131.40, 131.36, 130.11, 127.85, 127.86, 127.65, 127.48, 127.10, 126.95, 126.71, 126.52, 126.46, 124.16, 103.92, 100.09, 18.88, 11.49 ppm.

MALDI-TOF HRMS: Calcd for $C_{124}H_{130}N_4Si_4$: 1786.9373; Found: 1786.8945.

Synthesis of compound 2 = triPE-TIPS:

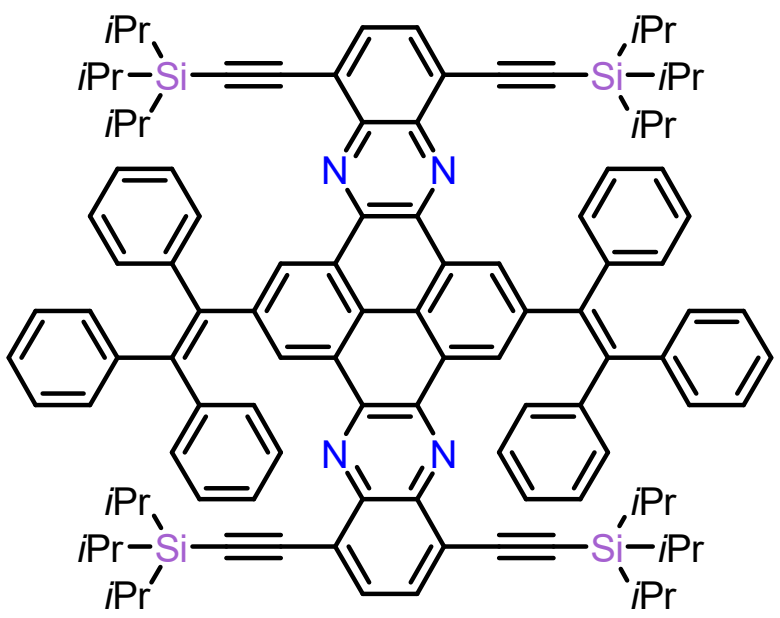

A mixture of TriPE-tetraketone (**9a**, 45 mg, 58 µmol, 1.00 equiv.) and 3,6-bis((triisopropylsilyl)ethynyl)benzene-1,2-diamine (**12b**, 60 mg, 128 µmol, 2.20 equiv.) was placed in a 25 mL round-bottomed flask. The flask was evacuated and filled with argon (1×). Freshly degassed $CHCl_3$ (2.5 mL) and degassed AcOH (0.5 mL) were added, and the reaction mixture was allowed to stir at 70 °C for 17 h (checked by TLC). The solvents were removed

in *vacuo* and the residue was co-evaporated with MeOH to remove traces of AcOH. The mixture was then triturated in MeOH (3 mL), filtered off over a sintered funnel. The solid was washed with MeOH (2.5 mL). The crude mixture was purified by column chromatography on silica gel (100−200 mesh, ⌀ = 1.5 cm, *l* = 12 cm) using 0−20% DCM in PE (SiO_2, R_f = 0.60 in 30% DCM/PE) to furnish the title compound **2** as a bright yellow solid (83 mg, 51 μmol, 87%). **M.p.**: > 250 °C.

IR (ATR): $\tilde{\nu}$ = 2941 (*w*), 2862 (*w*), 2365 (*w*), 2342 (*w*), 2138 (*w*), 1698 (*w*), 1593 (*w*), 1489 (*w*), 1460 (*w*), 1365 (*w*), 1320 (*w*), 1260 (*m*), 1131 (*m*), 1076 (*m*), 1017 (*m*), 919 (*m*), 881 (*w*), 847 (*m*), 797 (*s*), 758 (*w*), 726 (*w*), 697 (*s*), 675 (*s*), 663 (*s*), 639 (*m*), 614 (*w*) cm^{-1}.

^{1}H NMR ($CDCl_3$, 400 MHz, 298 K): δ = 9.48 (s, 4H), 7.94, (s, 4H), 7.07−7.24 (m, 24H), 6.71−6.92 (m, 6H), 1.23−1.26 (m, 84H) ppm.

^{13}C NMR ($CDCl_3$, 100 MHz, 298 K): δ = 144.10, 142.90, 142.87, 142.83, 142.76, 142.73, 142.17, 142.14, 142.11, 141.91, 141.77, 140.18, 134.31, 134.28, 131.23, 131.22, 131.21, 131.14, 130.54, 129.61, 129.59, 127.71, 127.70, 127.67, 127.54, 127.50, 126.76, 126.75, 126.63 (2C), 126.57, 126.20, 124.02, 123.99, 104.08, 99.72, 99.71, 19.00, 18.97, 18.96, 11.46, 11.44 ppm.

MALDI-TOF HRMS: Calcd for $C_{112}H_{122}N_4Si_4$•H^+: 1635.8825; Found: 1636.1059.

Synthesis of compound 3 = tetraPE-TMS:

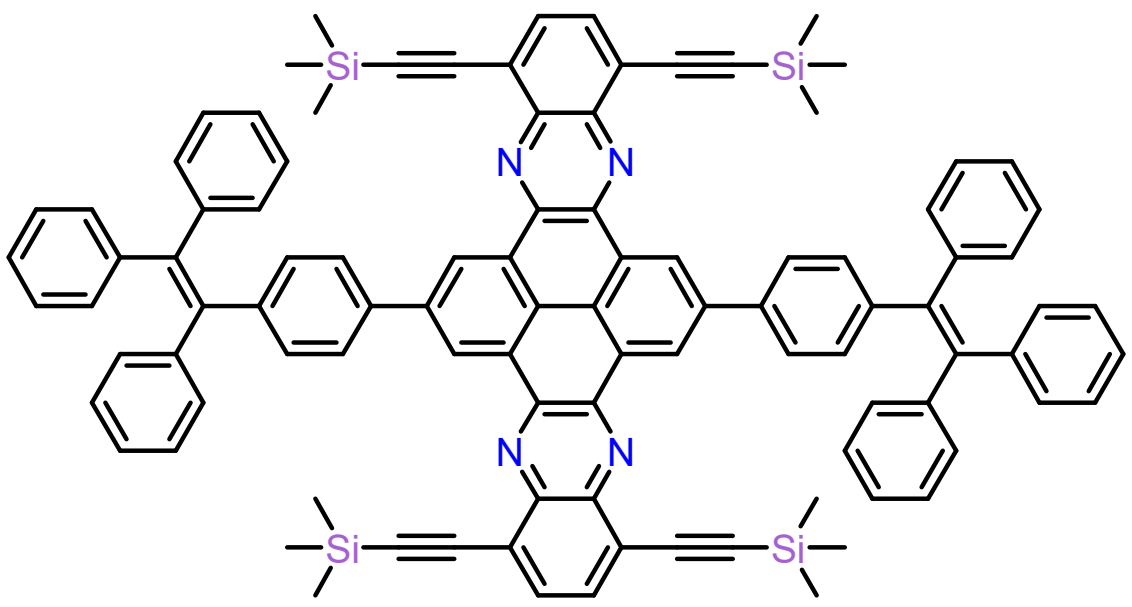

A mixture of tetraPE-tetraketone (**9b**, 55 mg, 60 μmol, 1.00 equiv.) and 3,6-bis((trimethylsilyl)ethynyl)benzene-1,2-diamine (**12a**, 40 mg, 131 μmol, 2.20 equiv.) was placed in a 25 mL round-bottomed flask. The flask was evacuated and filled with argon (1×). After the addition of freshly degassed $CHCl_3$ (2.5 mL) and degassed AcOH (0.5 mL), the reaction mixture was heated to 70 °C for 17 h (monitored on TLC). The solvents were removed under reduced pressure, and the residue was co-evaporated with MeOH to remove residual AcOH. The resultant mixture was triturated in MeOH (3 mL), filtered off over a sintered funnel, and washed with MeOH (2.5 mL). The crude product was purified by column chromatography on silica gel (230−400 mesh, ⌀ = 1.5 cm, *l* = 12 cm) using 0−30−50% DCM in PE (SiO_2, R_f = 0.30 in 30% DCM/PE) to provide the target compound **3** as a bright yellow solid (77 mg, 53 μmol, 89%).

M.p.: > 250 °C.

IR (ATR): $\tilde{\nu}$ = 2958 (*w*), 2149 (*w*), 1606 (*w*), 1489 (*w*), 1462 (*w*), 1444 (*w*), 1367 (*w*), 1326 (*w*), 1249 (*m*), 1223 (*w*), 1134 (*w*), 1061 (*w*), 940 (*w*), 914 (*w*), 842 (*s*), 778 (*w*), 754 (*m*), 701 (*s*), 661 (*w*), 646 (*w*), 626 (*w*) cm^{-1}.

^{1}H NMR ($CDCl_3$, 400 MHz, 298 K): δ = 10.11 (s, 4H), 8.03 (s, 4H), 7.90 (d, ^3J = 8.4 Hz, 4H), 7.26 (d, ^3J = 8.4 Hz, 4H), 7.07−7.17 (m, 30H), 0.33 (s, 36H) ppm.

^{13}C NMR ($CDCl_3$, 100 MHz, 298 K): δ = 143.89, 143.76, 143.51, 143.05, 142.28, 141.13, 140.53, 140.42, 140.35, 138.14, 134.03, 132.12, 131.42, 131.41, 131.29, 129.98, 127.88, 127.75, 127.66, 127.26, 127.15, 126.89, 126.79, 126.50, 126.36, 123.87, 103.78, 101.39, 0.20 ppm.

MALDI-TOF HRMS: Calcd for $C_{100}H_{74}N_4Si_4$: 1450.5617; Found: 1450.5195.

Synthesis of compound 4 = triPE-TMS:

A mixture of triPE-tetraketone (**9a**, 70 mg, 91 µmol, 1.00 equiv.) and 3,6-bis((trimethylsilyl)ethynyl)benzene-1,2-diamine (**12a**, 60 mg, 200 µmol, 2.20 equiv.) was

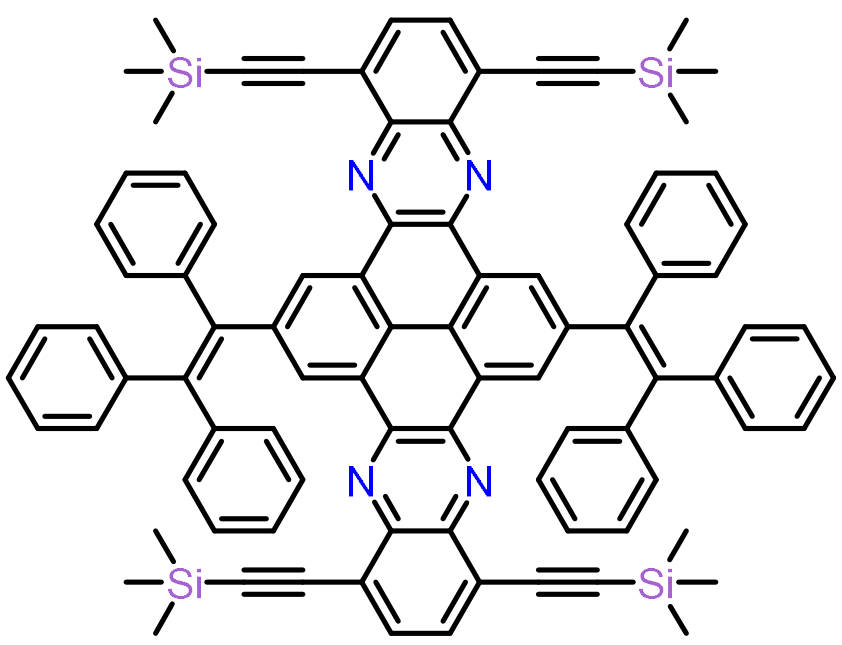

added to a 25 mL round-bottomed flask. The flask was evacuated and flushed with argon (1×). After the addition of degassed $CHCl_3$ (3.7 mL) and degassed AcOH (0.8 mL), the reaction mixture was stirred at 70 °C for 17 h (checked by TLC).

The solvents were evaporated, and the mixture was co-evaporated with MeOH to remove traces of AcOH. The crude residue was triturated in MeOH (3 mL), filtered off over a sintered funnel, and washed with MeOH (2.5 mL). The crude residue was subjected to column chromatography on silica gel (100−200 mesh, ⌀ = 1.5 cm, *l* = 12 cm) using 0−30% DCM in PE (SiO_2, R_f = 0.50 in 30% DCM/PE) to afford the title compound **4** as a bright yellow solid (104 mg, 80 µmol, 88%).

M.p.: > 250 °C.

IR (ATR): $\tilde{\nu}$ = 2958 (*w*), 2154 (*w*), 1491 (*w*), 1463 (*w*), 1367 (*w*), 1322 (*w*), 1248 (*m*), 1131 (*m*), 1078 (*w*), 844 (*w*), 843 (*s*), 761 (*m*), 739 (*w*), 698 (*w*), 647 (*w*) cm^{-1}.

^{1}H NMR ($CDCl_3$, 400 MHz, 298 K): δ = 9.55 (s, 4H), 7.96 (s, 4H), 7.10−7.22 (m, 24H), 6.75−7.00 (m, 6H), 0.40 (s, 36H) ppm.

^{13}C NMR ($CDCl_3$, 100 MHz, 298 K): δ = 144.09, 144.05, 143.10, 142.96, 142.91, 142.81, 142.79, 142.76, 142.65, 142.14, 142.11, 140.56, 140.51, 133.81, 131.38, 131.28, 131.24, 131.20, 131.17, 130.80, 129.53, 127.95, 127.74, 127.71, 126.76, 126.69, 126.64, 126.39, 126.37, 123.80, 103.44, 101.66, 0.30 ppm.

MALDI-TOF HRMS: Calcd for $C_{88}H_{122}N_4Si_4{\bullet}H^+$: 1299.5063; Found: 1299.5784.

2. ^{1}H and ^{13}C NMR Spectra

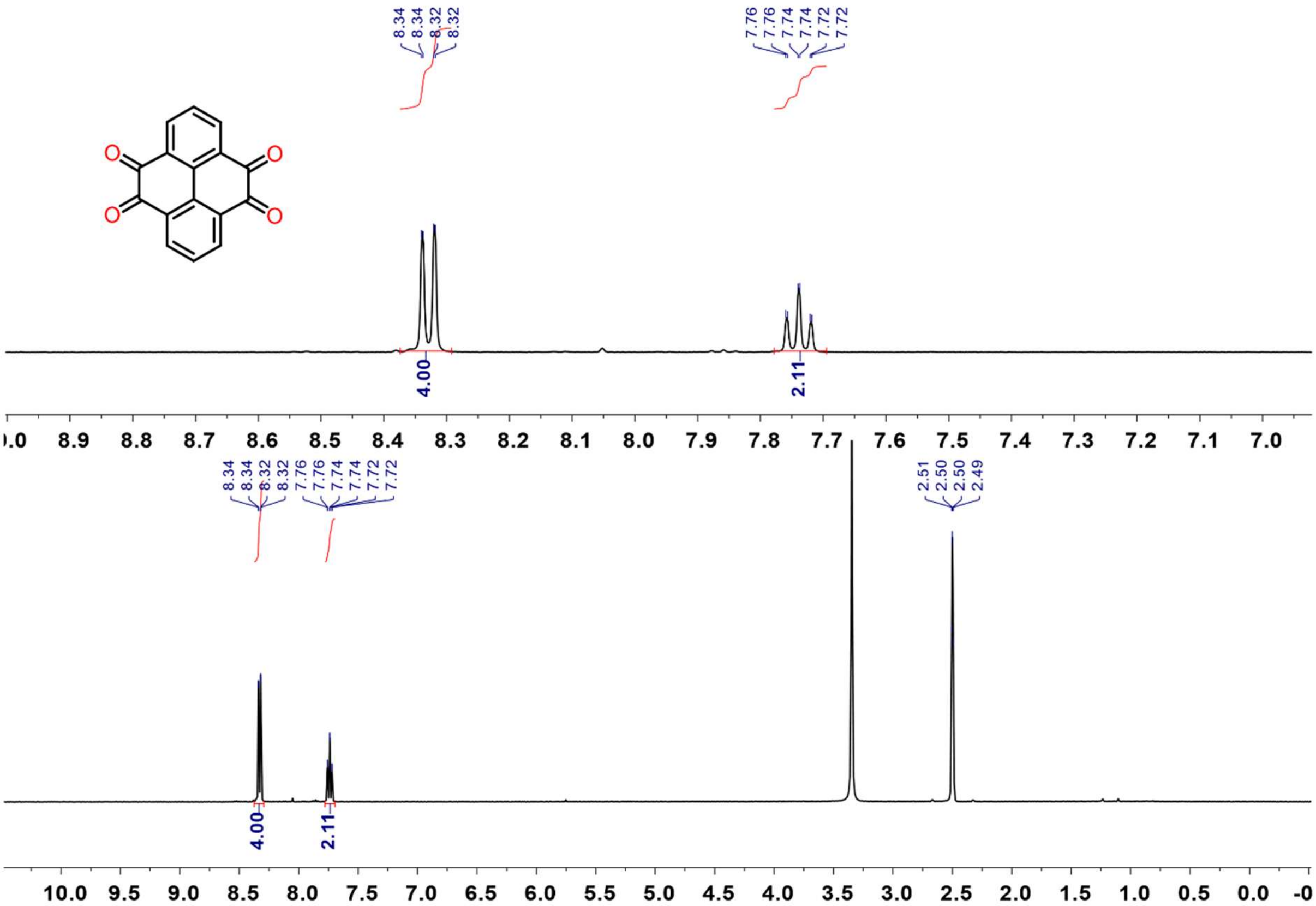

Figure S1. ^{1}H NMR spectrum (DMSO-d_6, 400 MHz, 298 K) of compound **S1**.

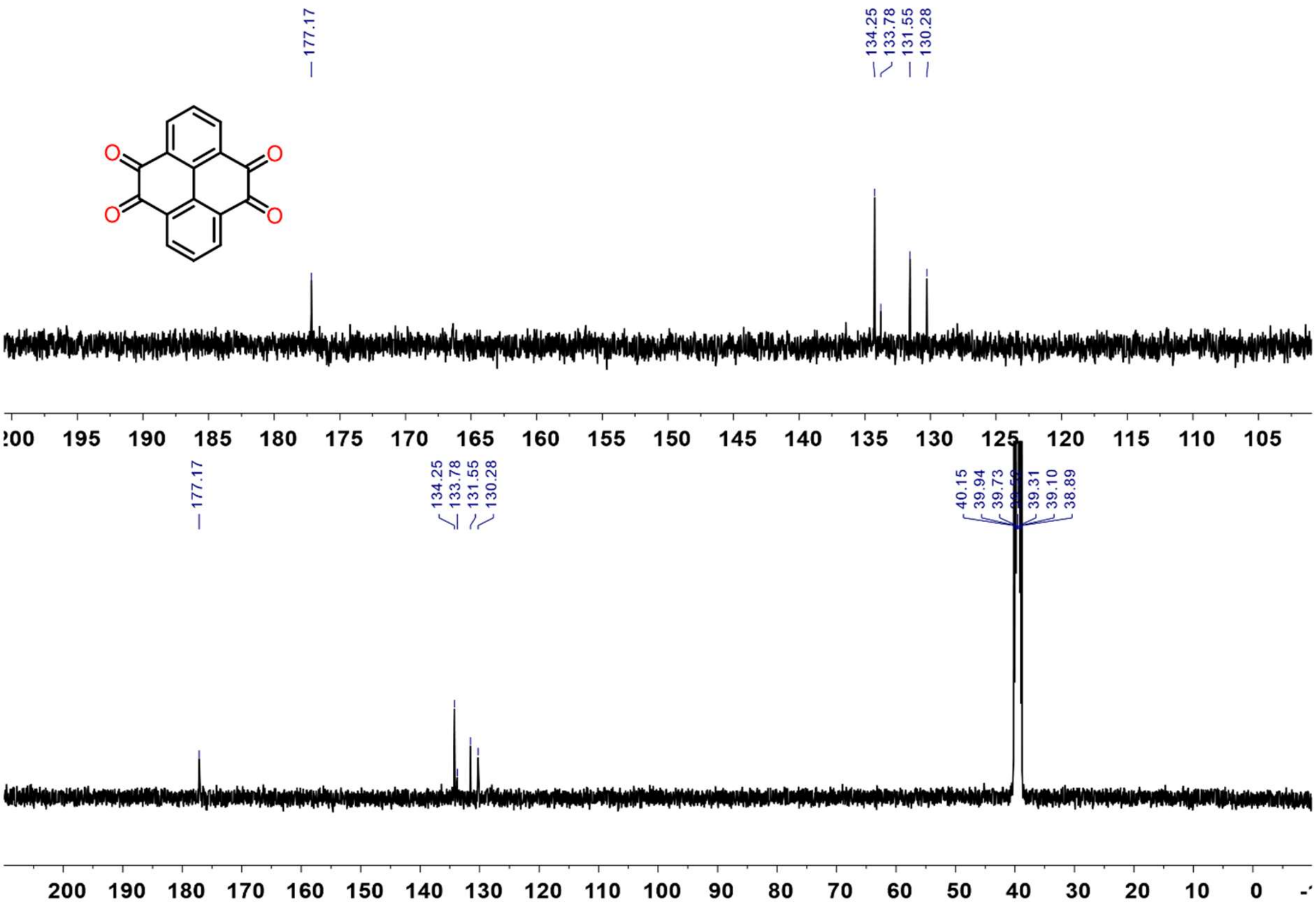

Figure S2. ^{13}C NMR spectrum (DMSO-d_6, 100 MHz, 298 K) of compound **S1**.

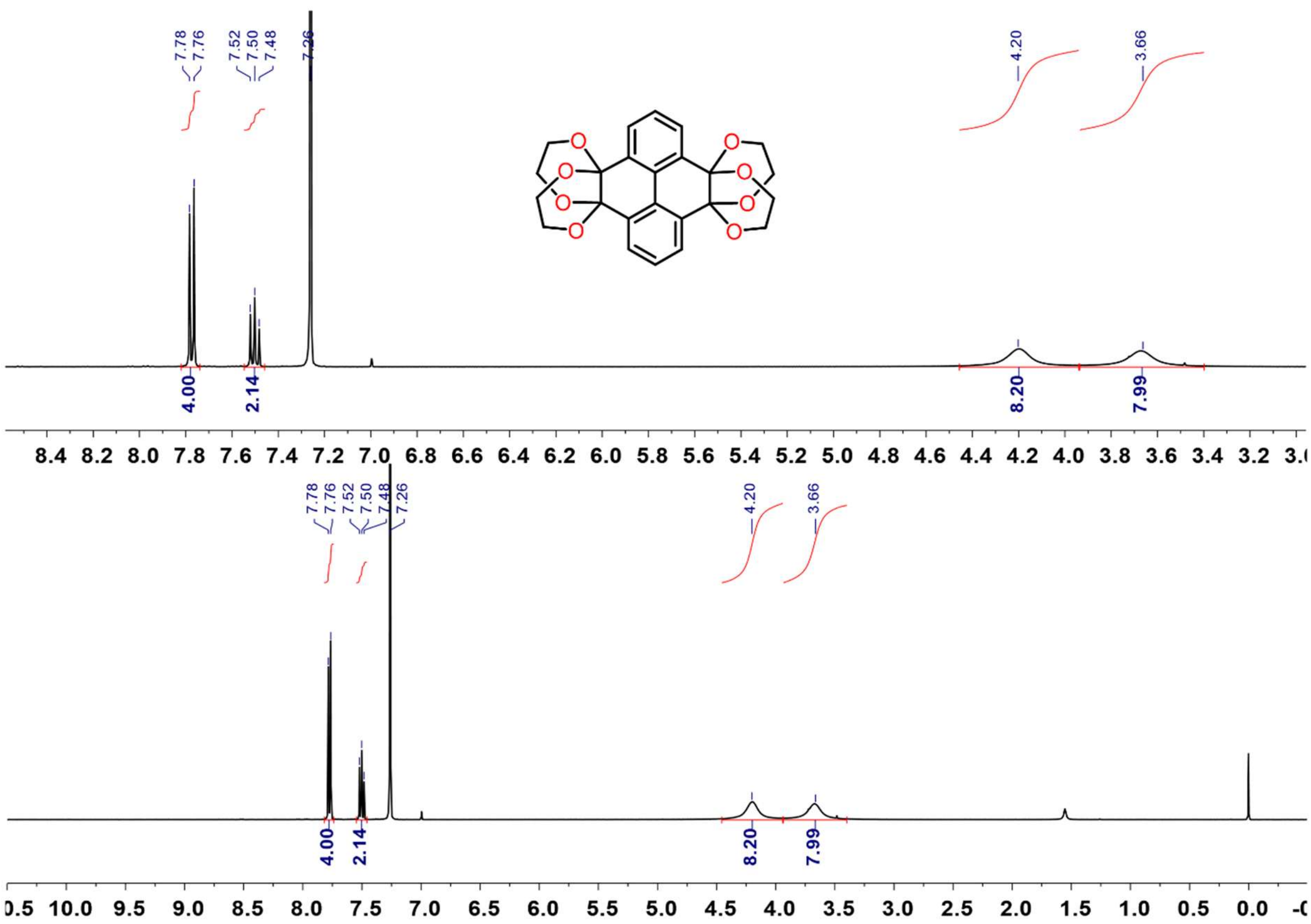

Figure S3. ^{1}H NMR spectrum ($CDCl_3$, 400 MHz, 298 K) of compound **S2**.

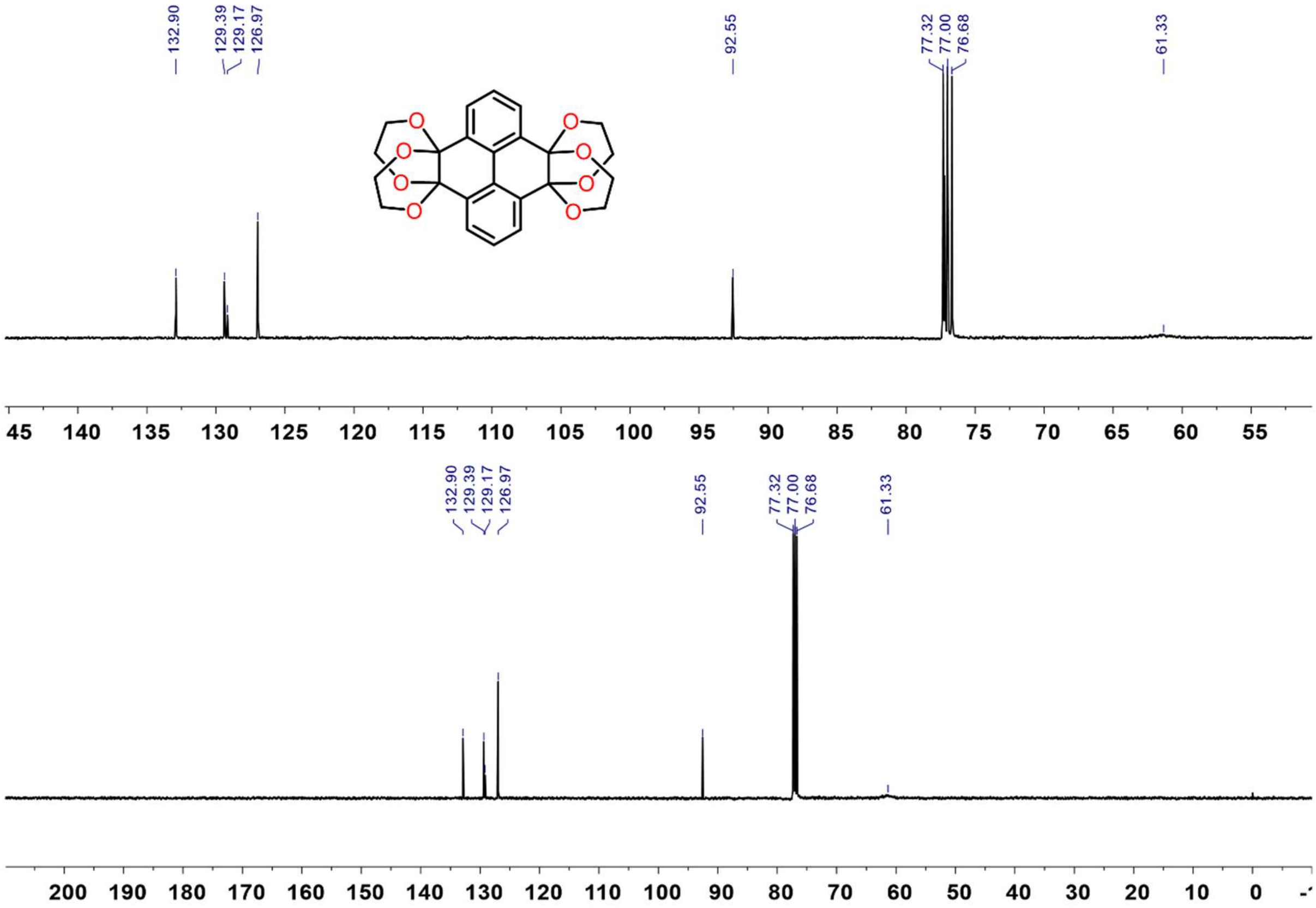

Figure S4. ^{13}C NMR spectrum ($CDCl_3$, 100 MHz, 298 K) of compound **S2**.

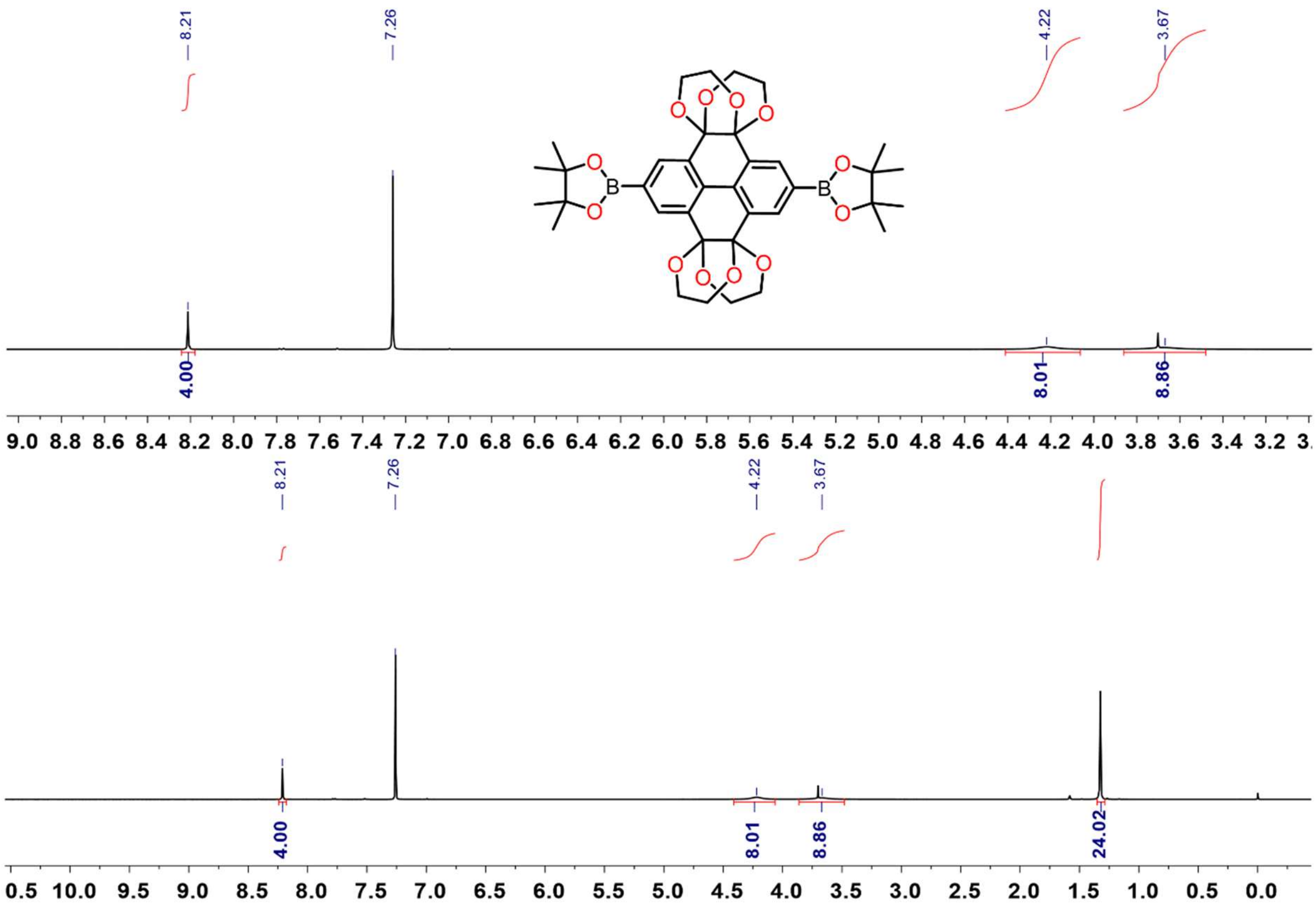

Figure S5. ^{1}H NMR spectrum ($CDCl_3$, 400 MHz, 298 K) of compound **5**.

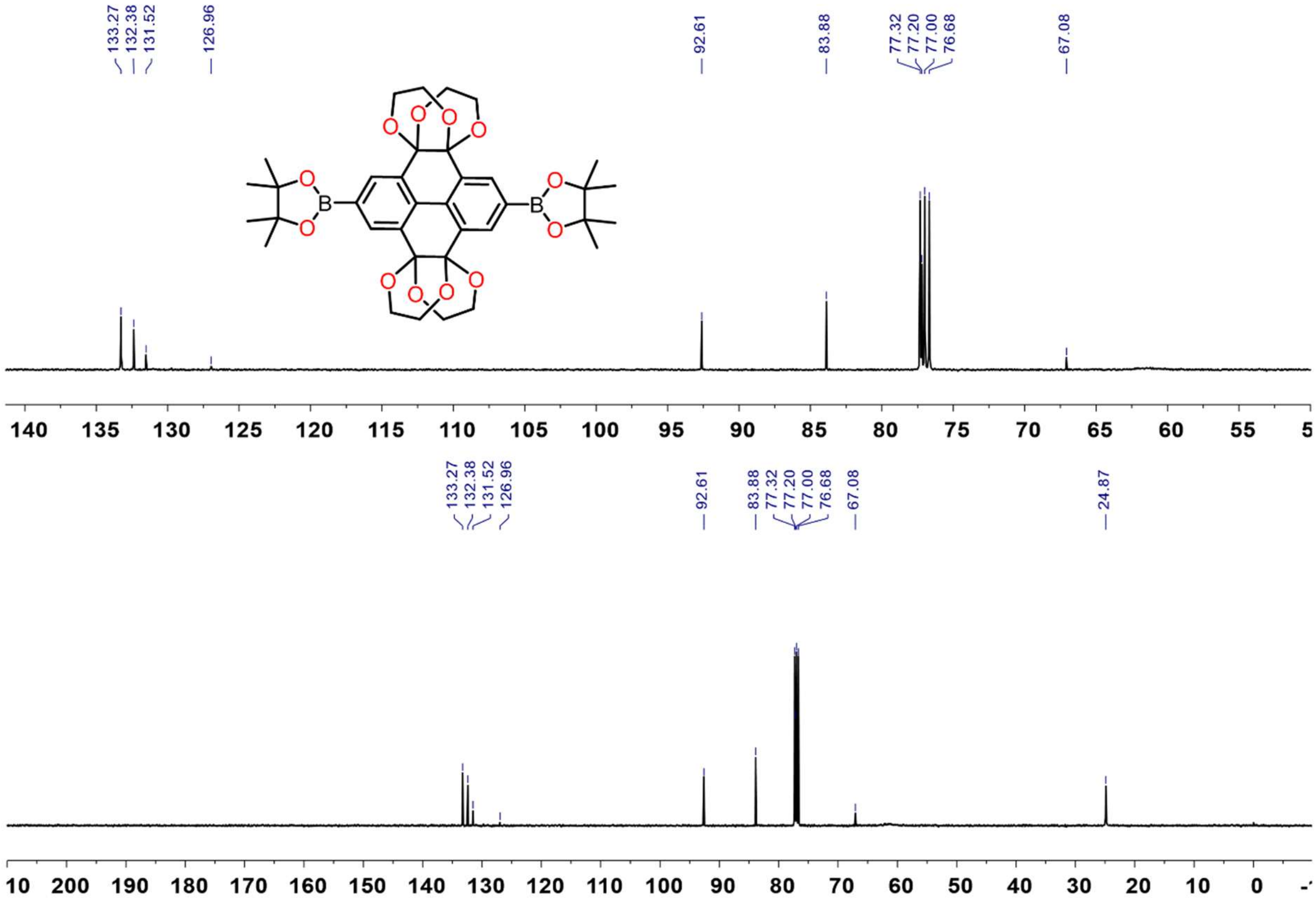

Figure S6. ^{13}C NMR spectrum ($CDCl_3$, 100 MHz, 298 K) of compound **5**.

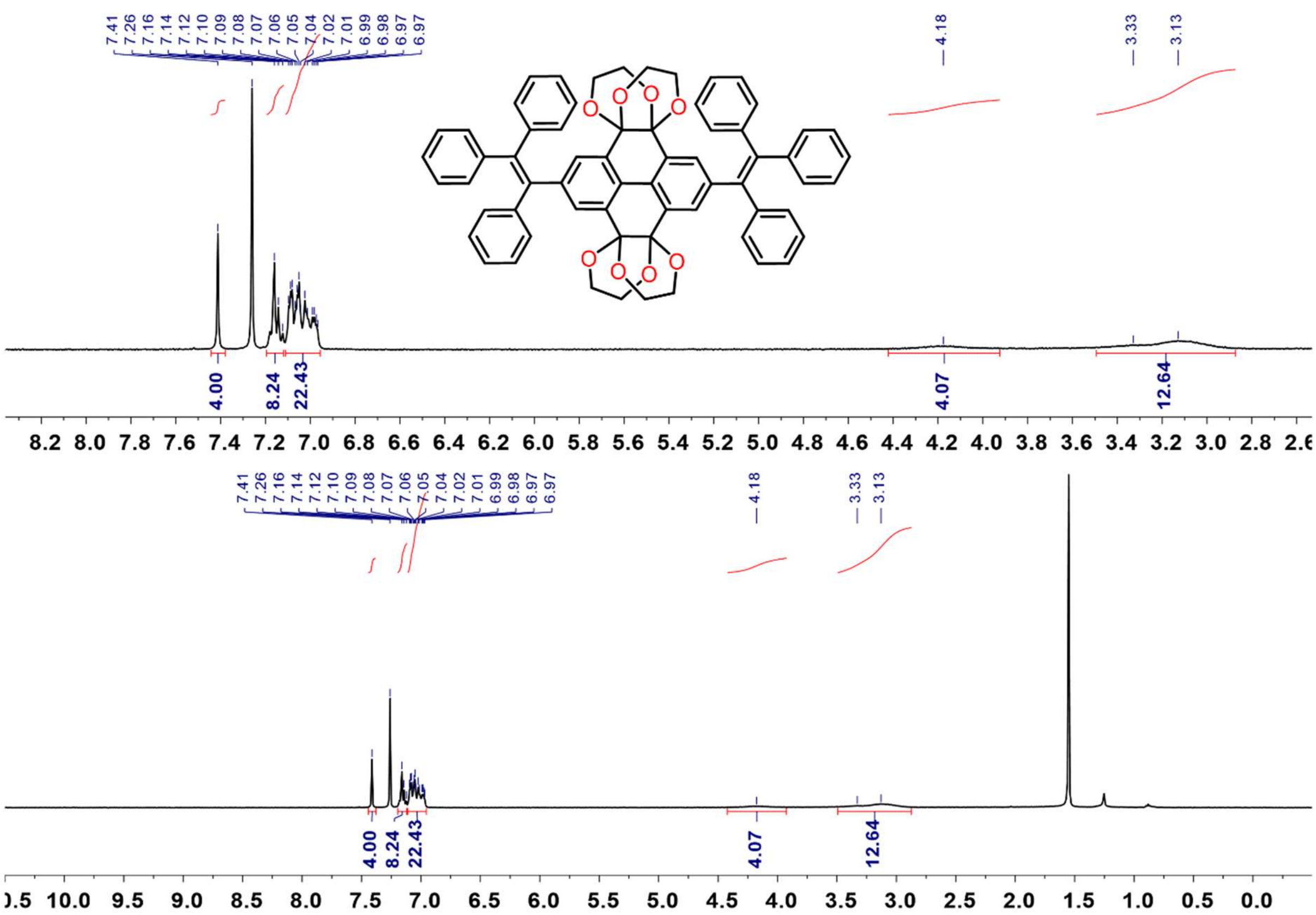

Figure S7. ^{1}H NMR spectrum ($CDCl_3$, 400 MHz, 298 K) of compound **6a**.

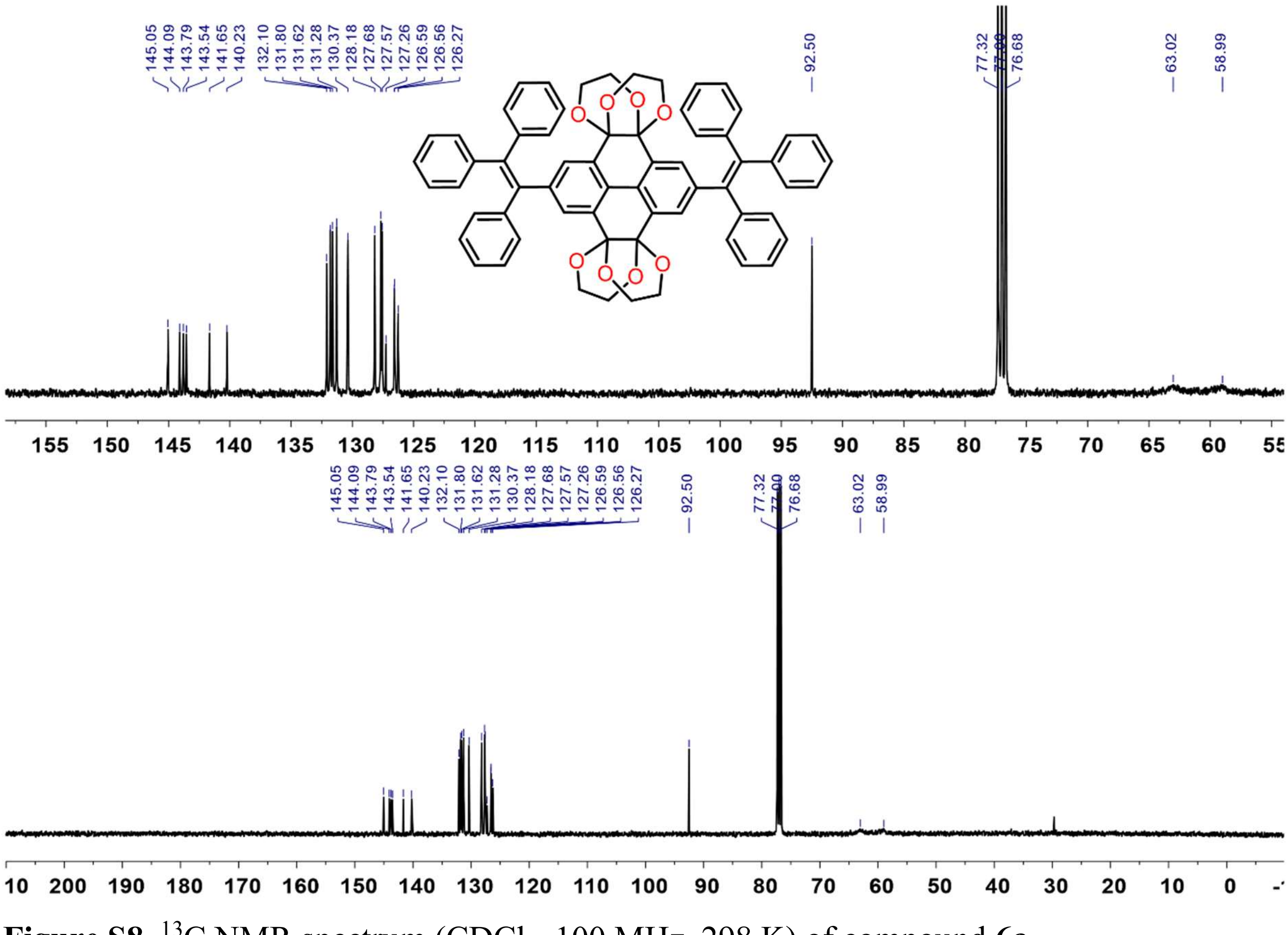

Figure S8. ^{13}C NMR spectrum ($CDCl_3$, 100 MHz, 298 K) of compound **6a**.

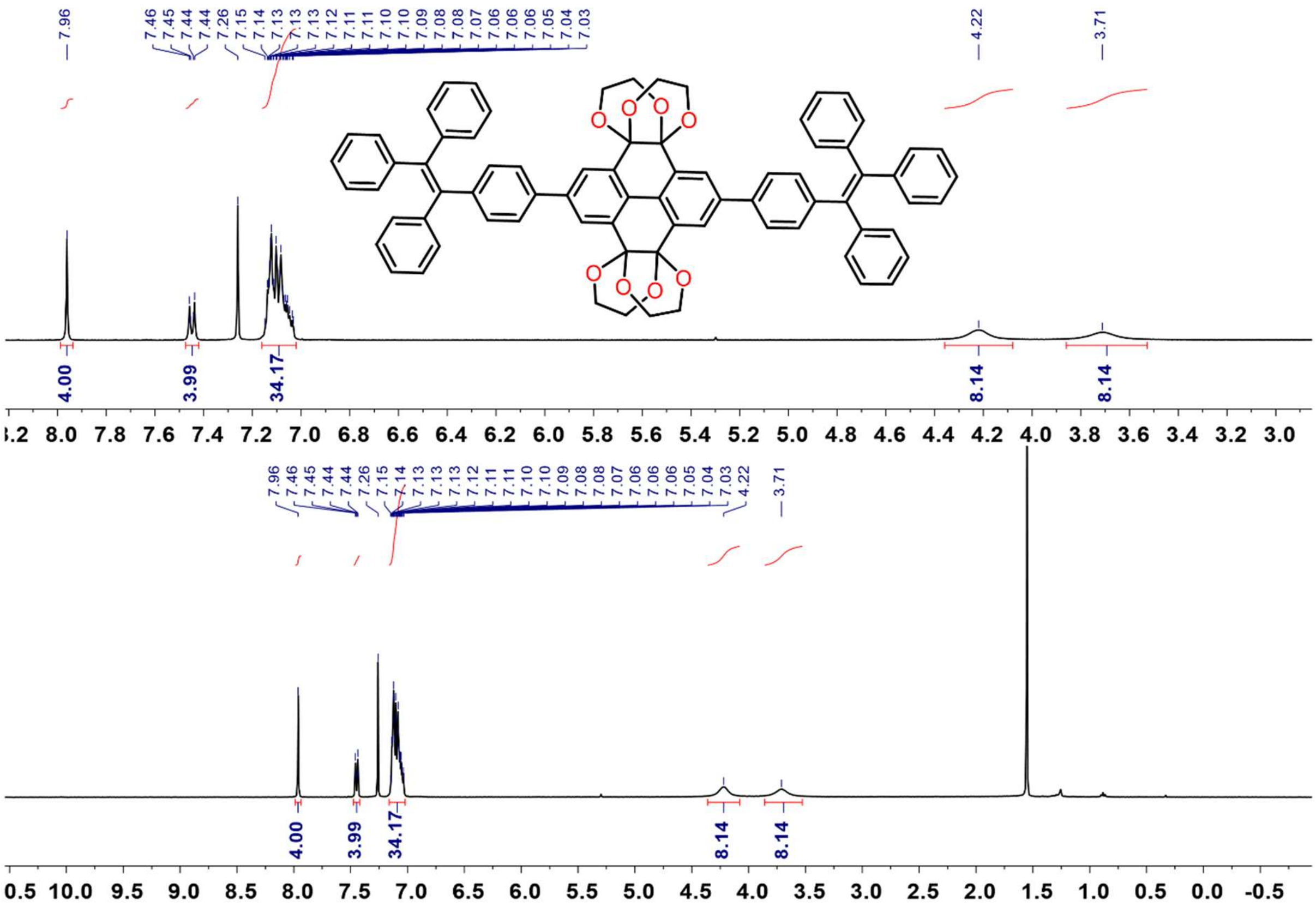

Figure S9. ^{1}H NMR spectrum ($CDCl_3$, 400 MHz, 298 K) of compound **6b**.

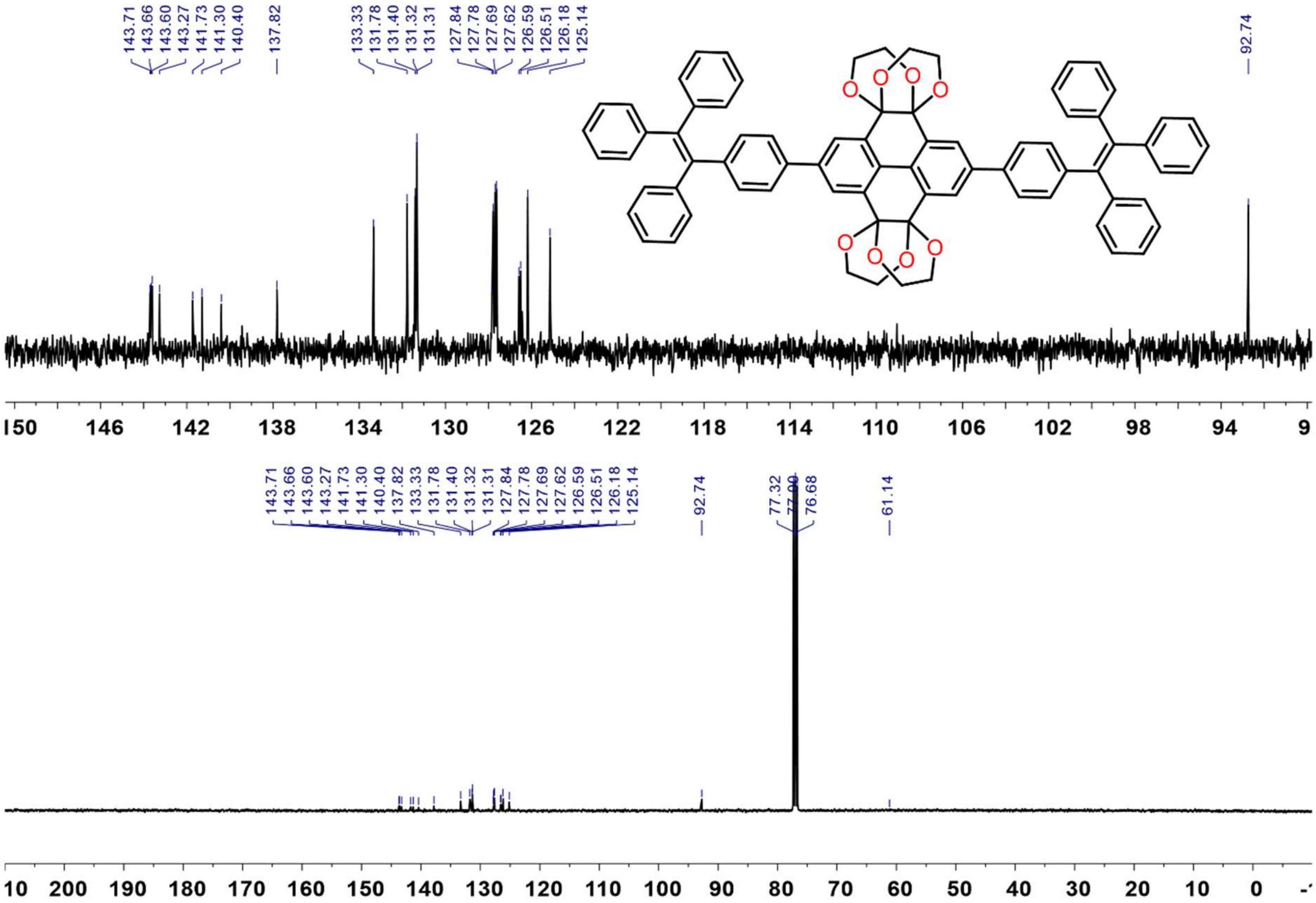

Figure S10. ^{13}C NMR spectrum ($CDCl_3$, 100 MHz, 298 K) of compound **6b**.

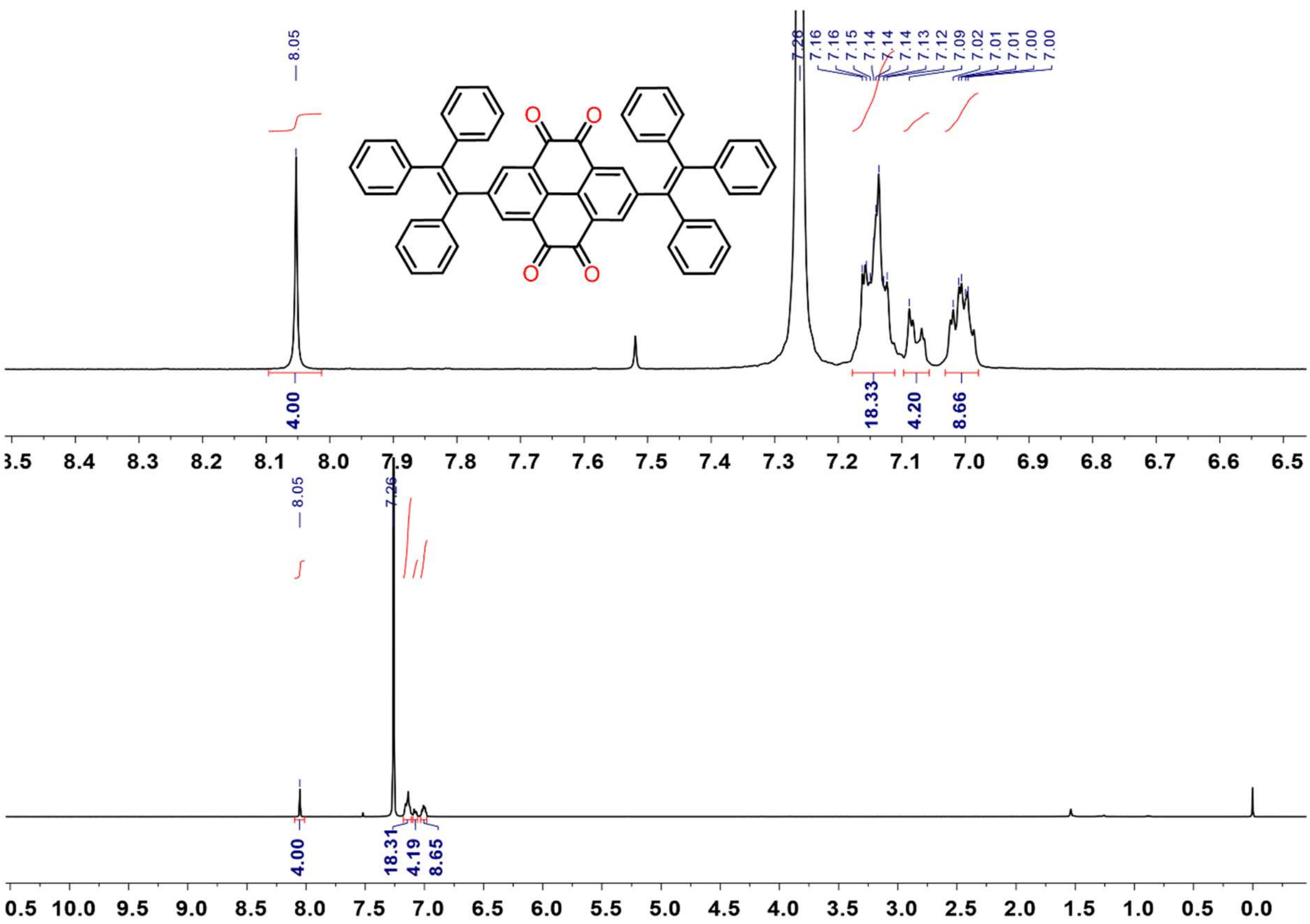

Figure S11. ^{1}H NMR spectrum ($CDCl_3$, 400 MHz, 298 K) of compound **7a**.

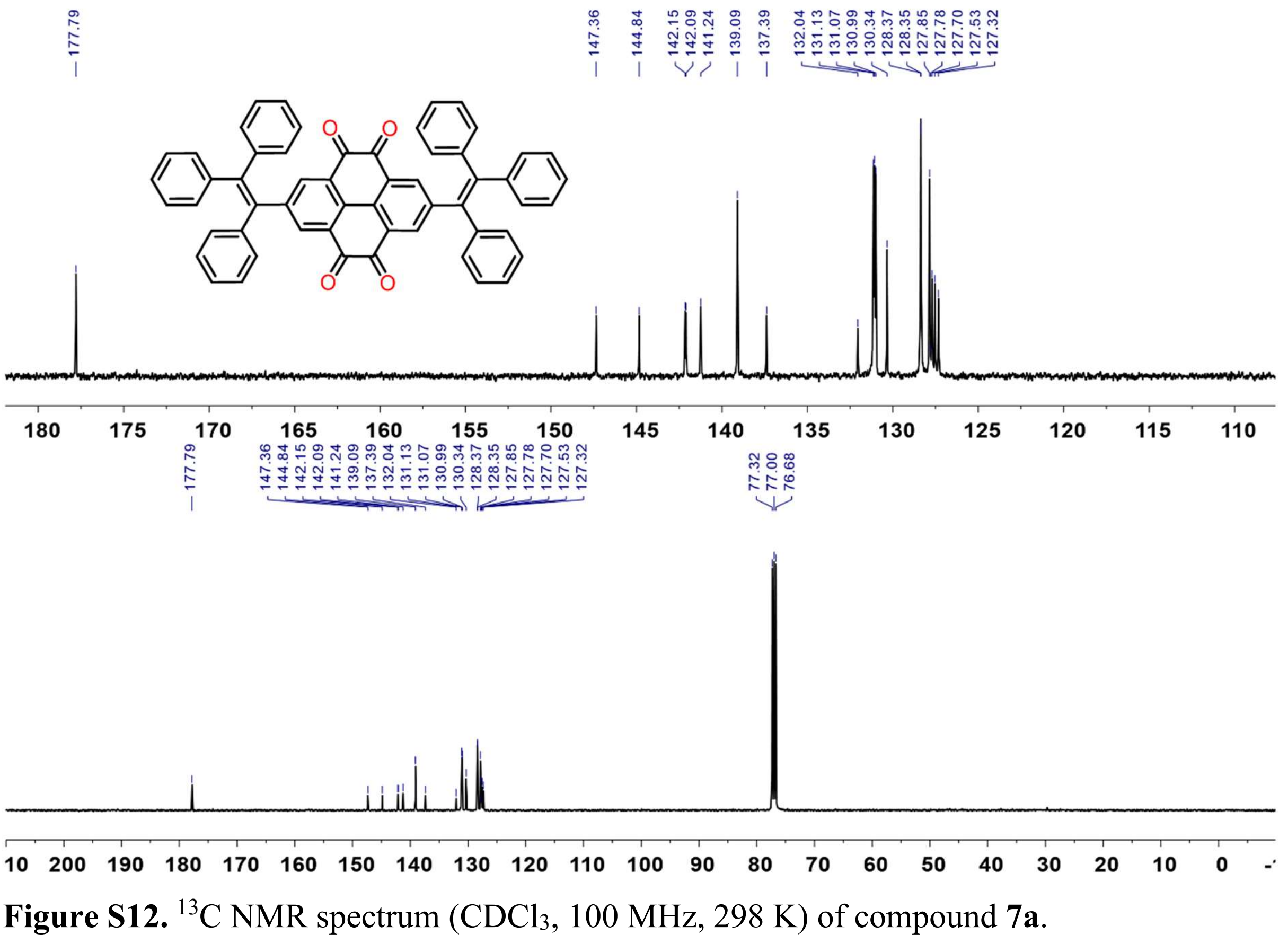

Figure S12. ^{13}C NMR spectrum ($CDCl_3$, 100 MHz, 298 K) of compound **7a**.

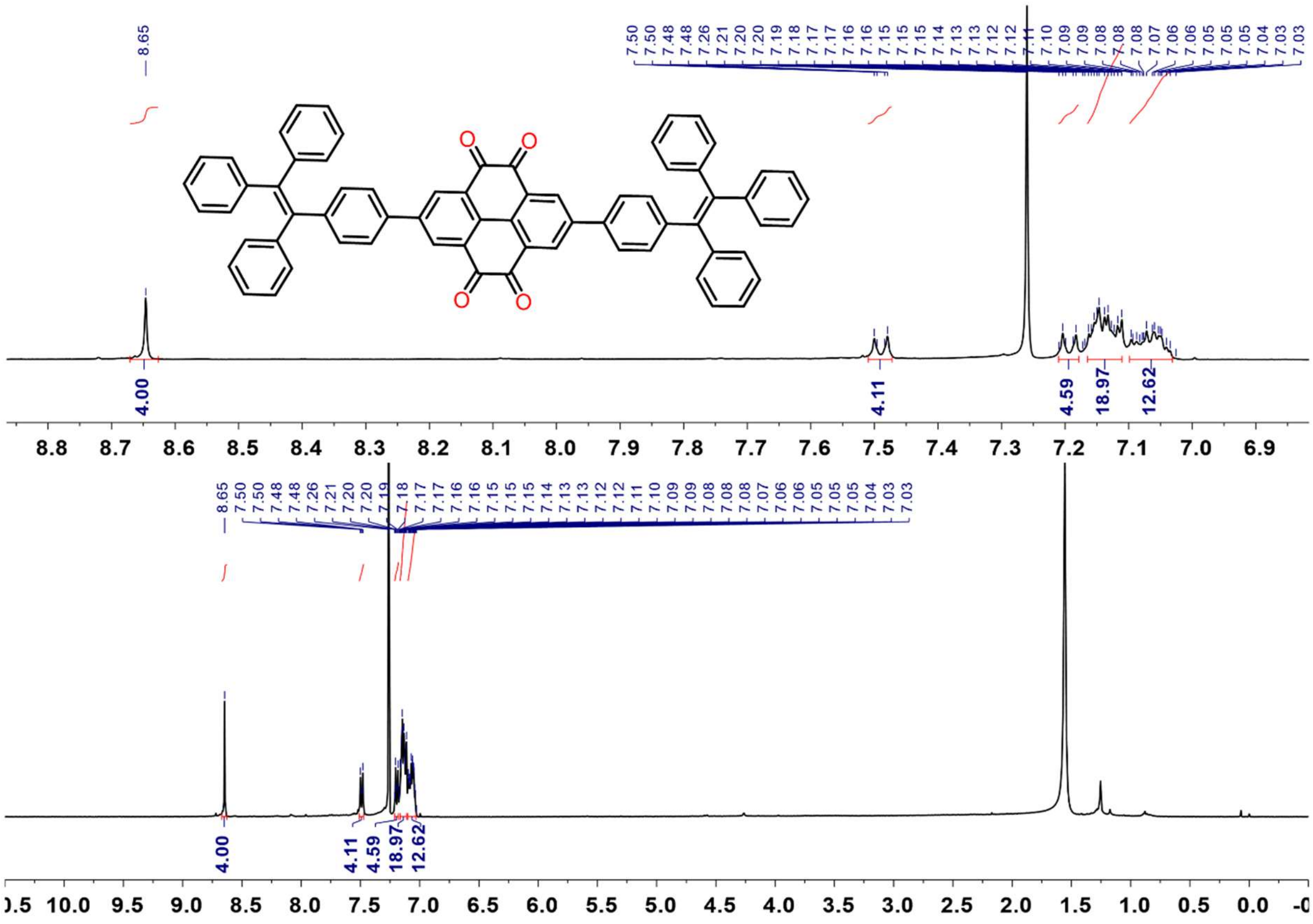

Figure S13. ^{1}H NMR spectrum ($CDCl_3$, 400 MHz, 298 K) of compound **7b**.

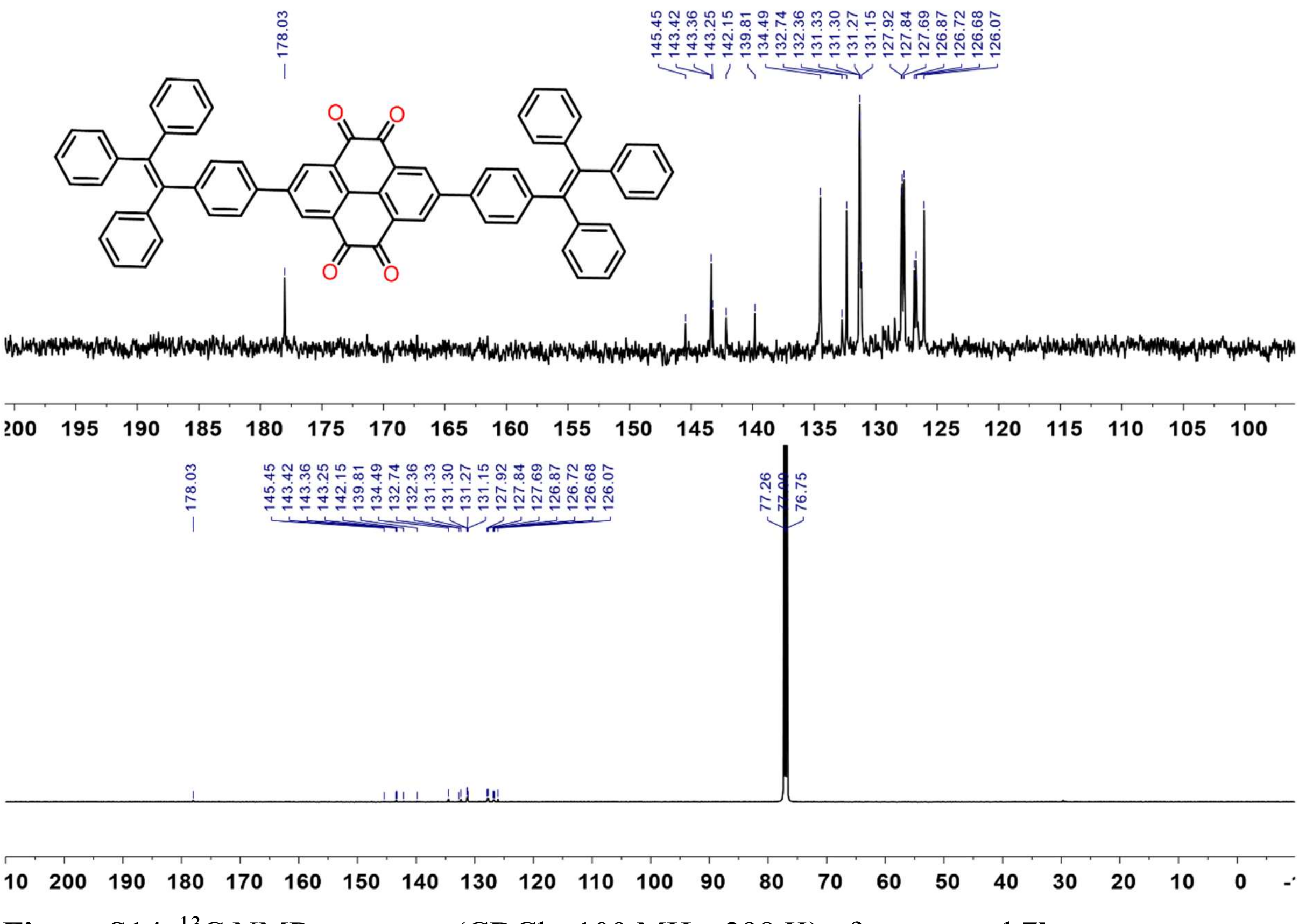

Figure S14. ^{13}C NMR spectrum ($CDCl_3$, 100 MHz, 298 K) of compound **7b**.

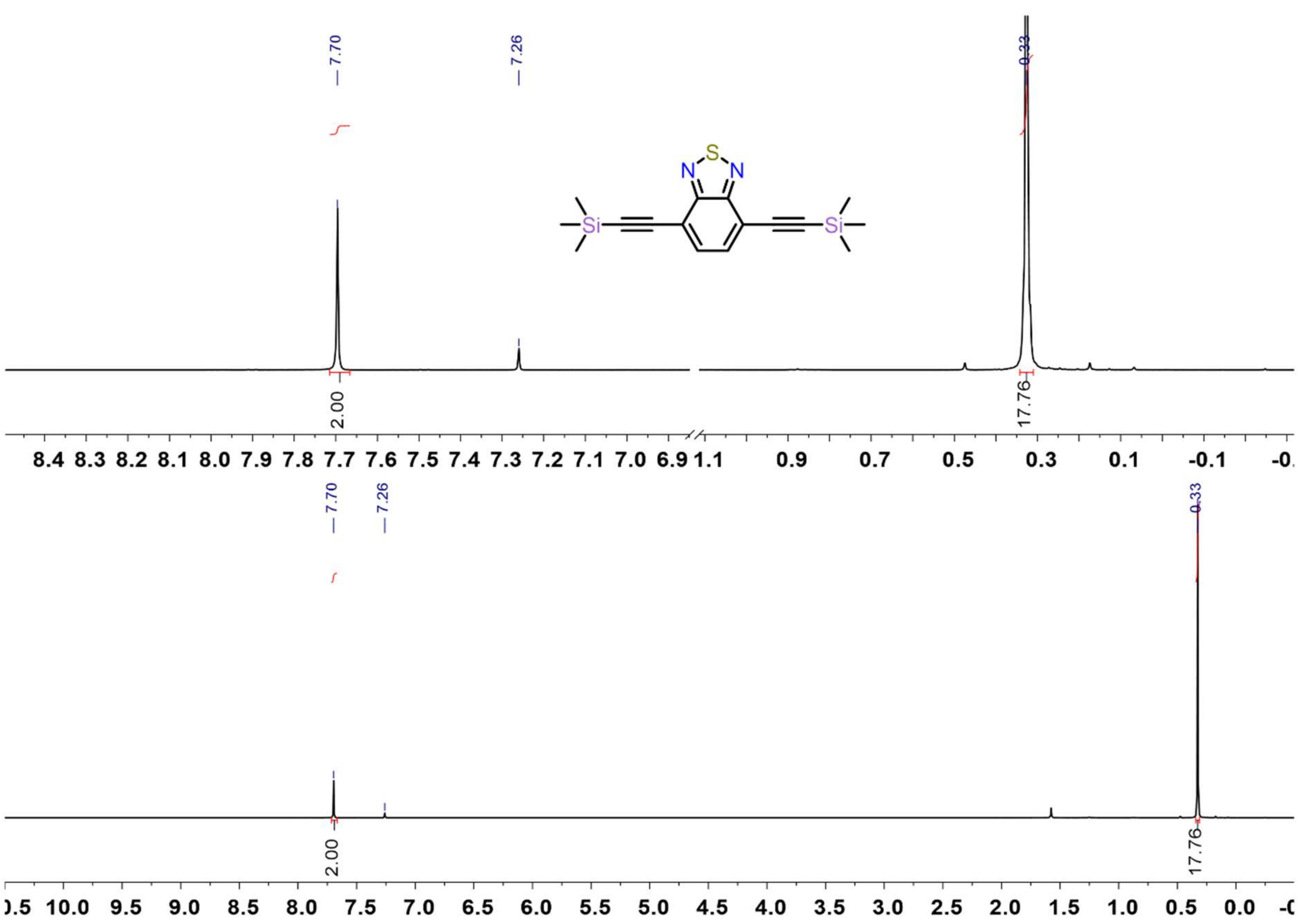

Figure S15. ^{1}H NMR spectrum ($CDCl_3$, 400 MHz, 298 K) of compound **S4a**.

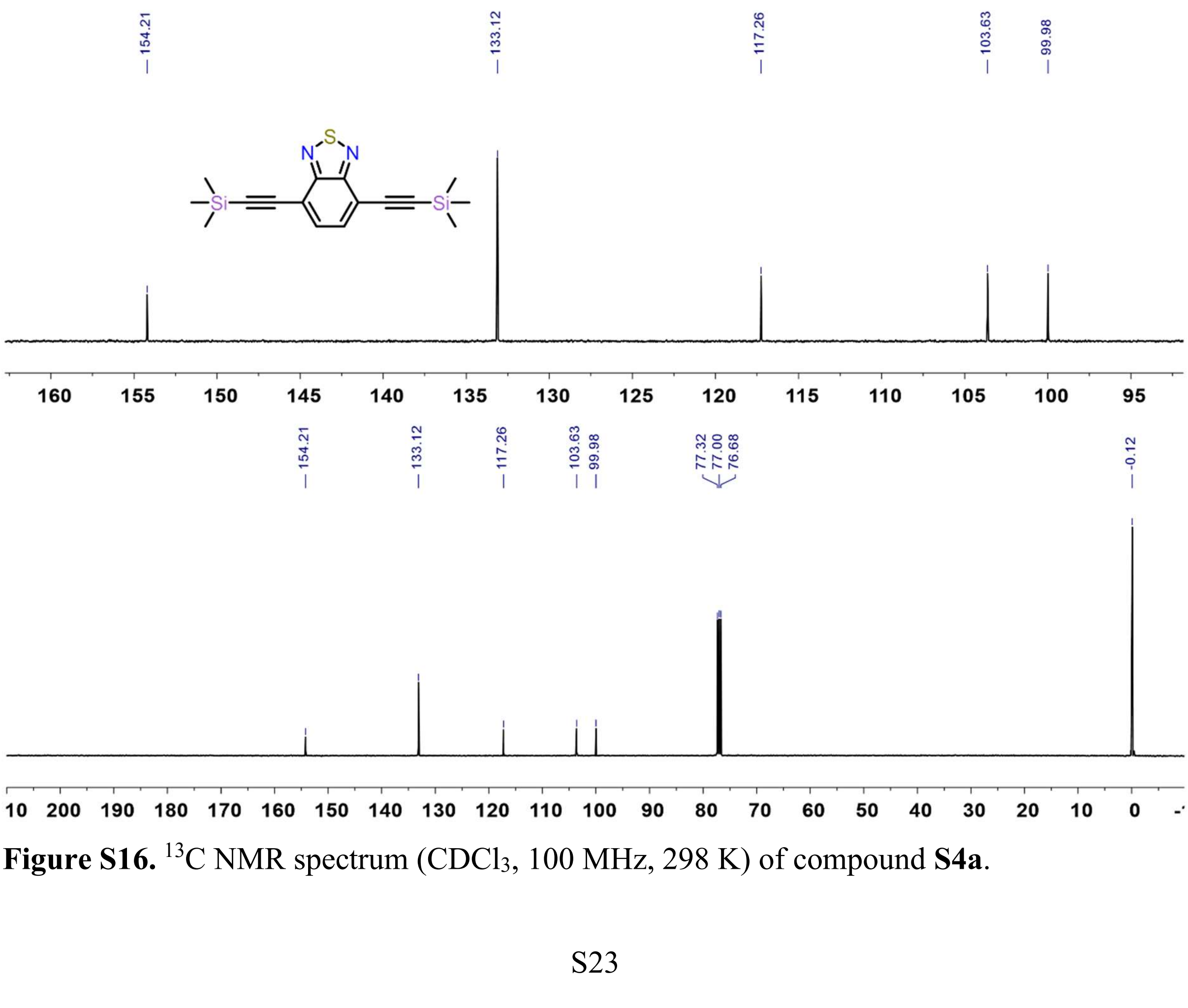

Figure S16. ^{13}C NMR spectrum ($CDCl_3$, 100 MHz, 298 K) of compound **S4a**.

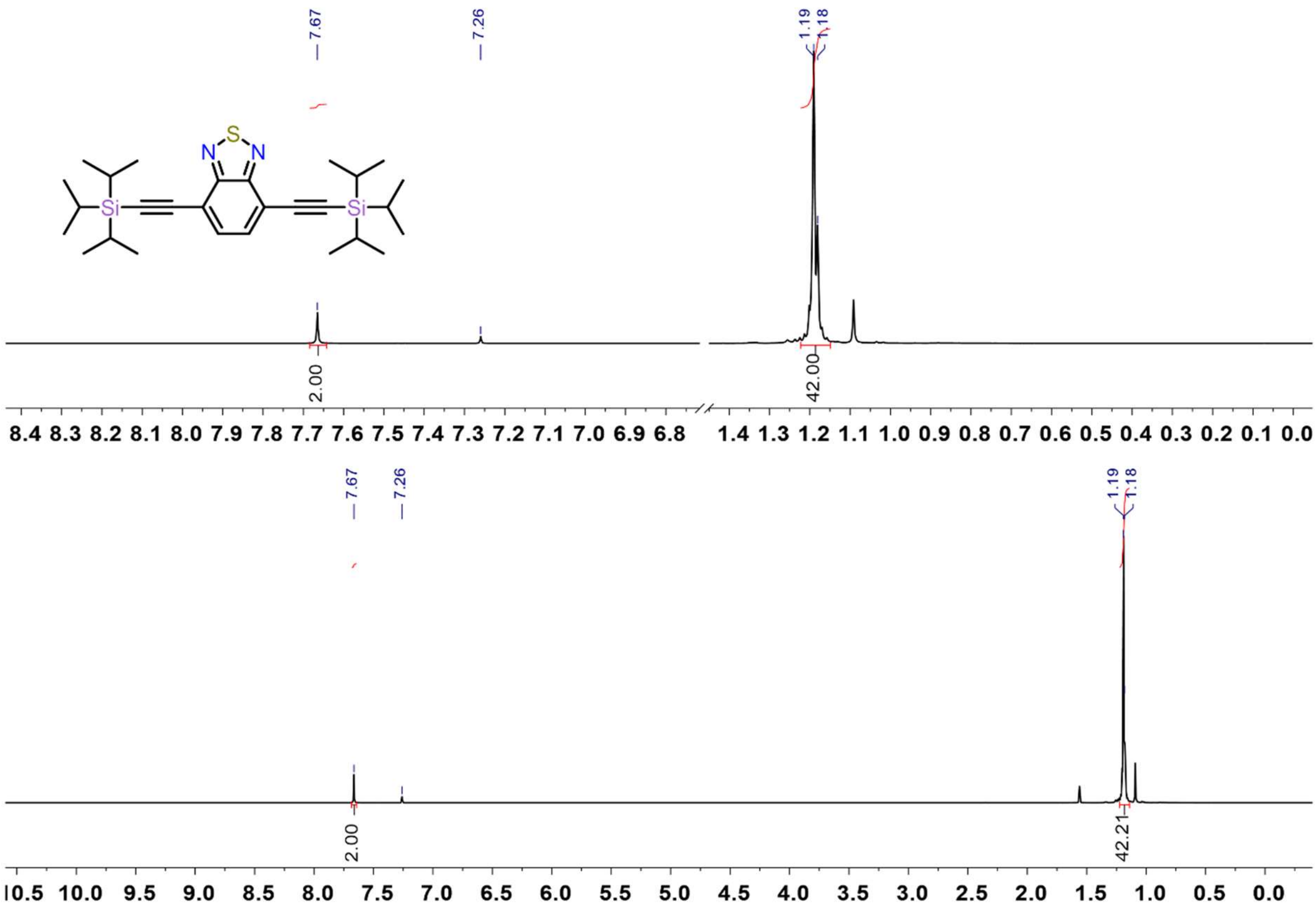

Figure S17. ^{1}H NMR spectrum ($CDCl_3$, 400 MHz, 298 K) of compound **S4b**.

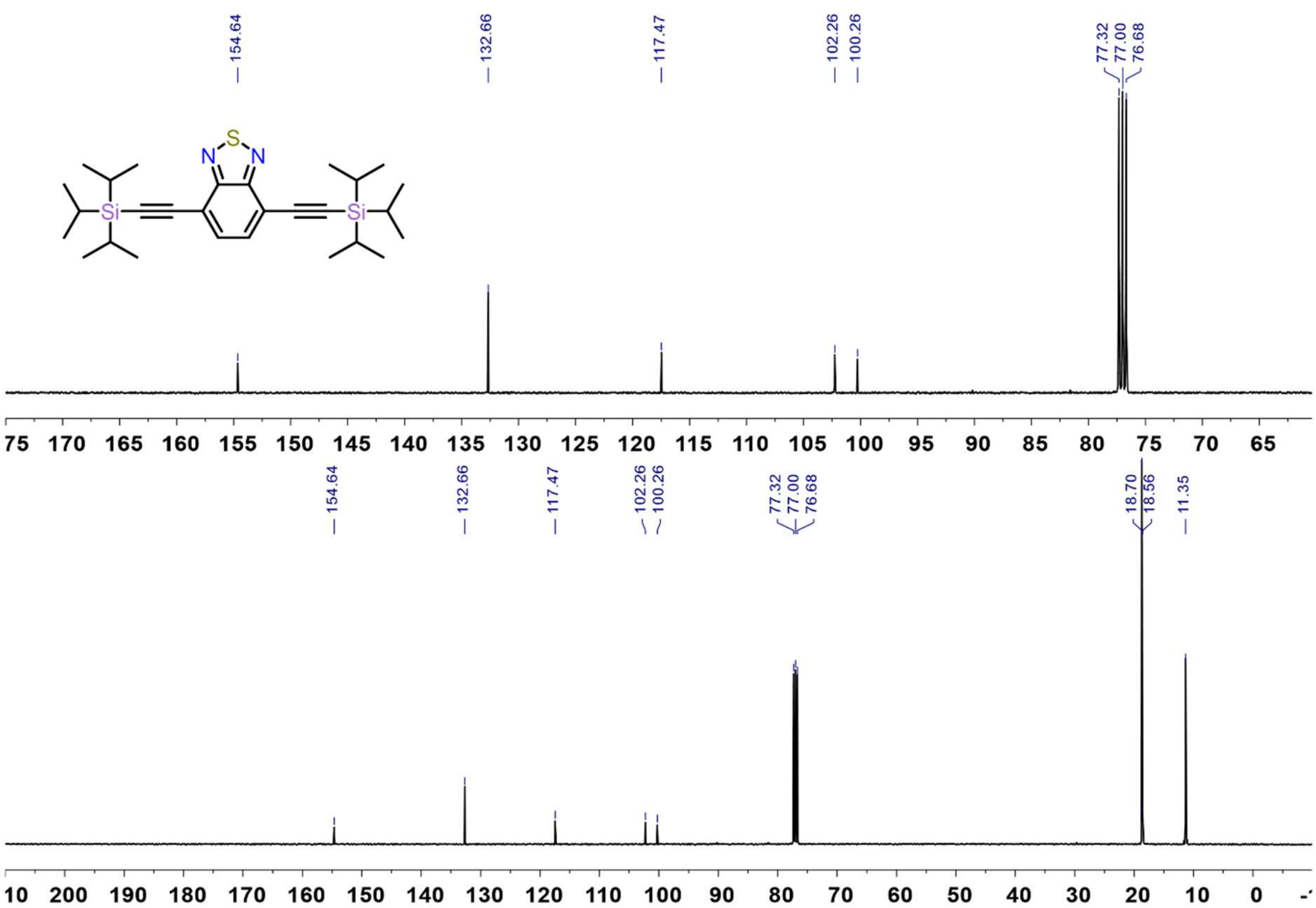

Figure S18. ^{13}C NMR spectrum ($CDCl_3$, 100 MHz, 298 K) of compound **S4b**.

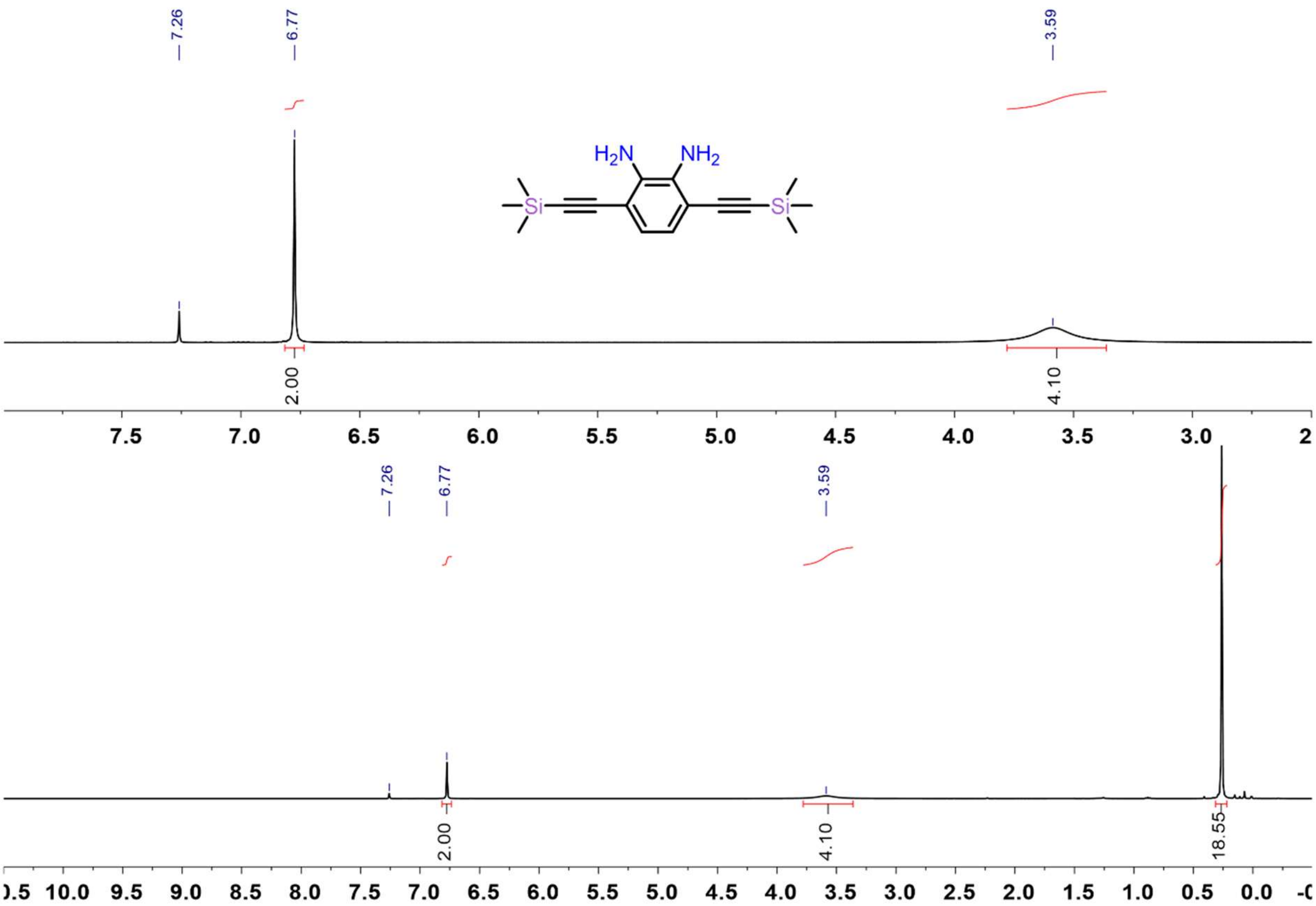

Figure S19. ^{1}H NMR spectrum ($CDCl_3$, 400 MHz, 298 K) of compound **8a**.

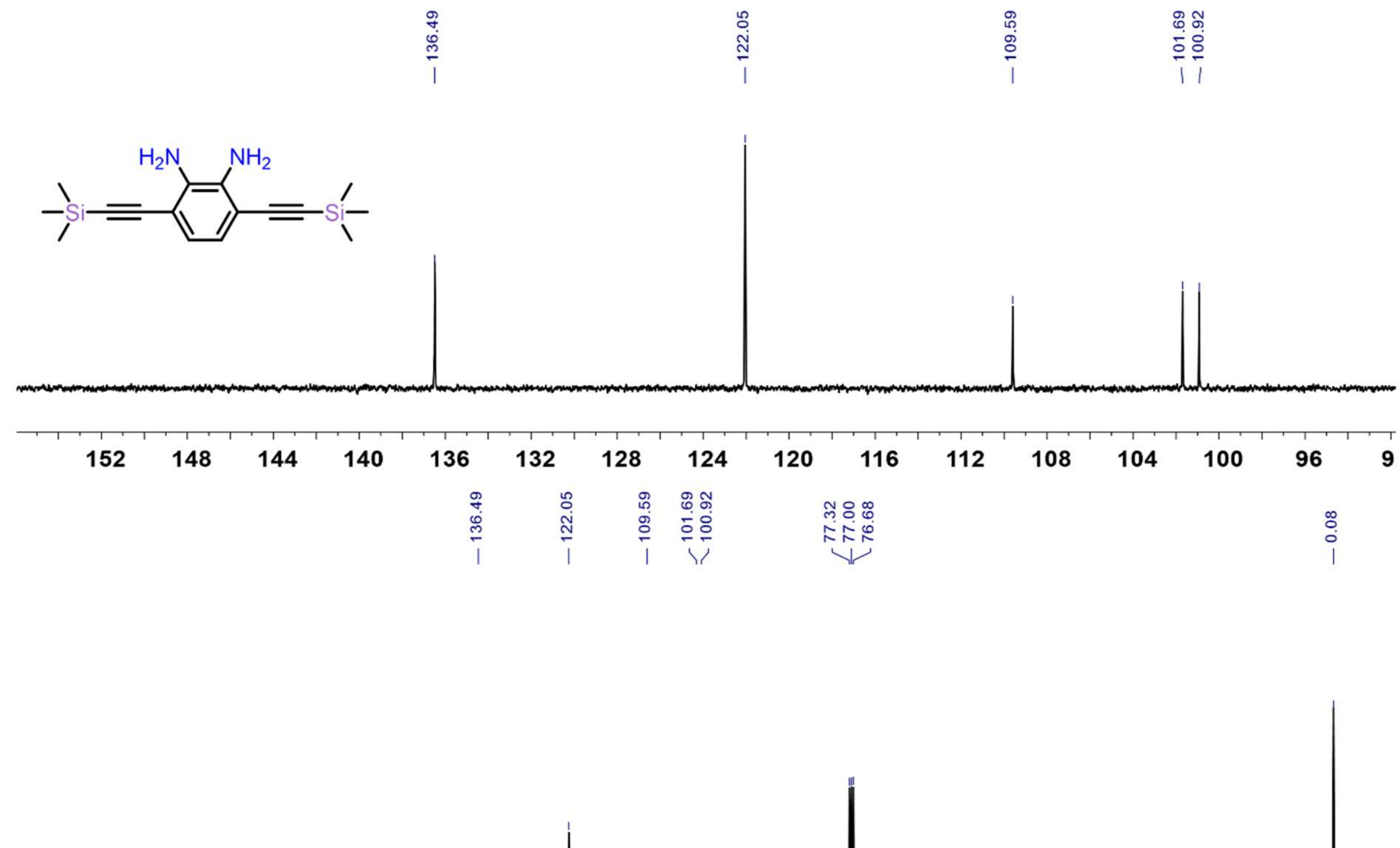

Figure S20. ^{13}C NMR spectrum ($CDCl_3$, 100 MHz, 298 K) of compound **8a**.

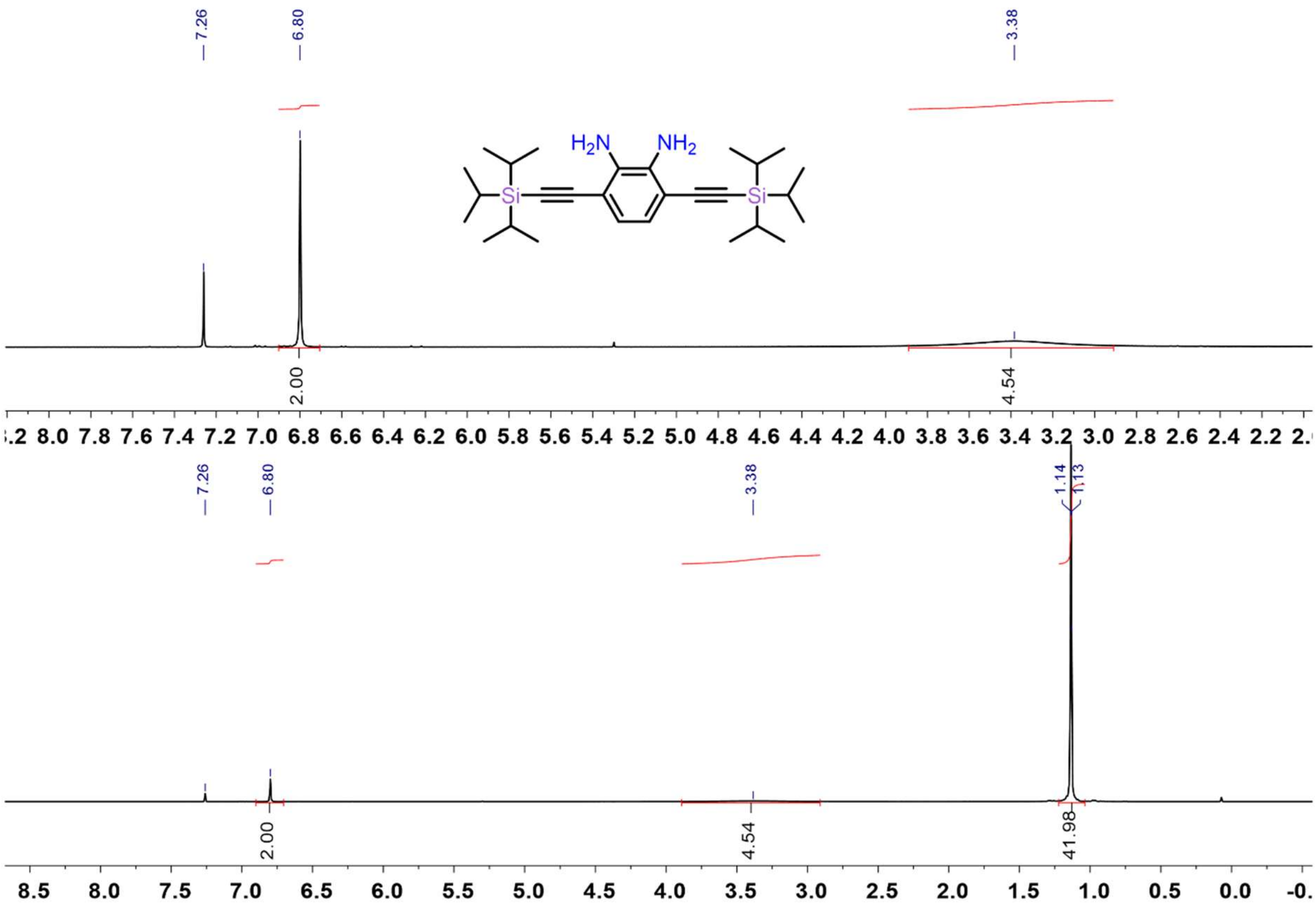

Figure S21. ^{1}H NMR spectrum ($CDCl_3$, 400 MHz, 298 K) of compound **8b**.

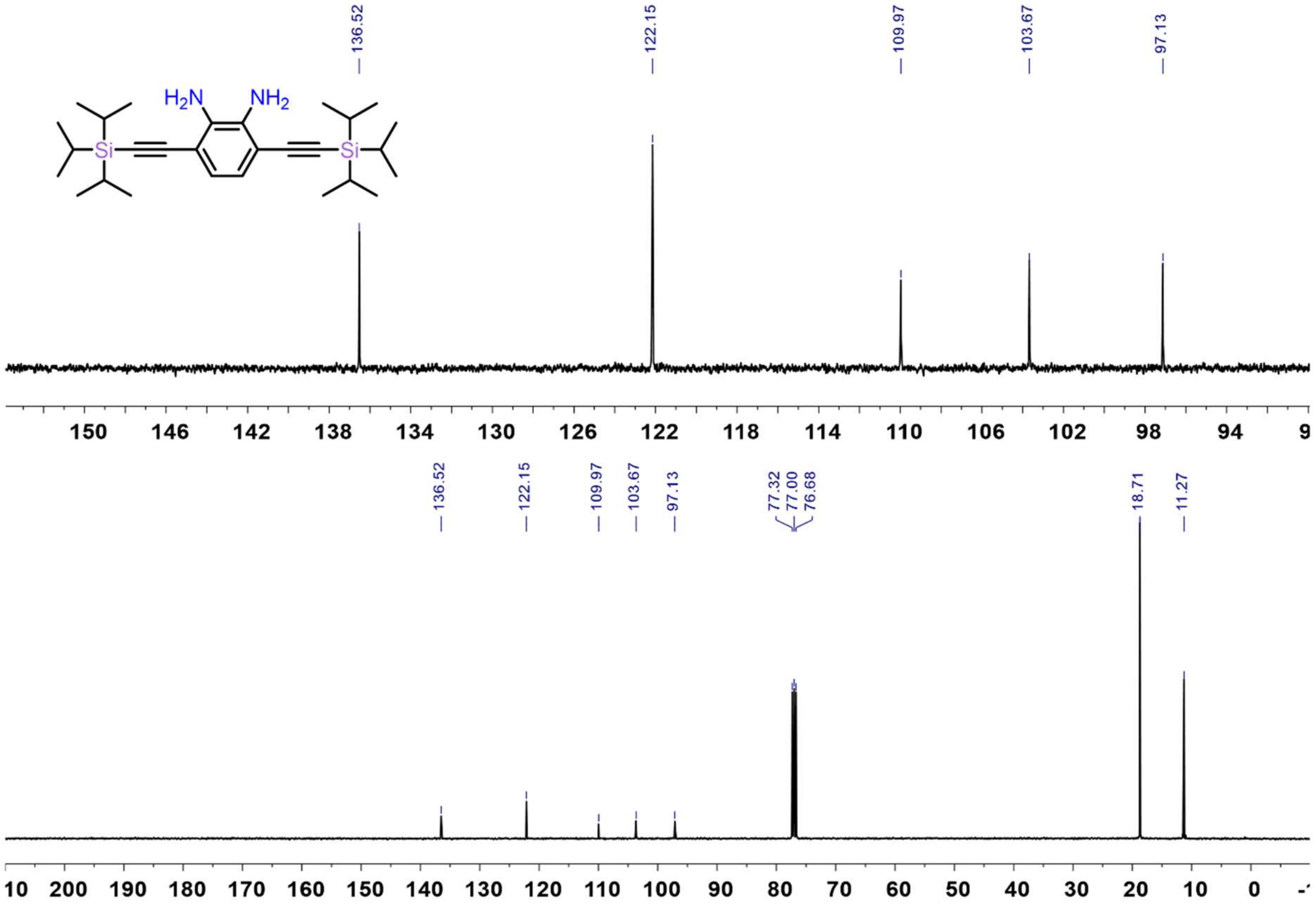

Figure S22. ^{13}C NMR spectrum ($CDCl_3$, 100 MHz, 298 K) of compound **8b**.

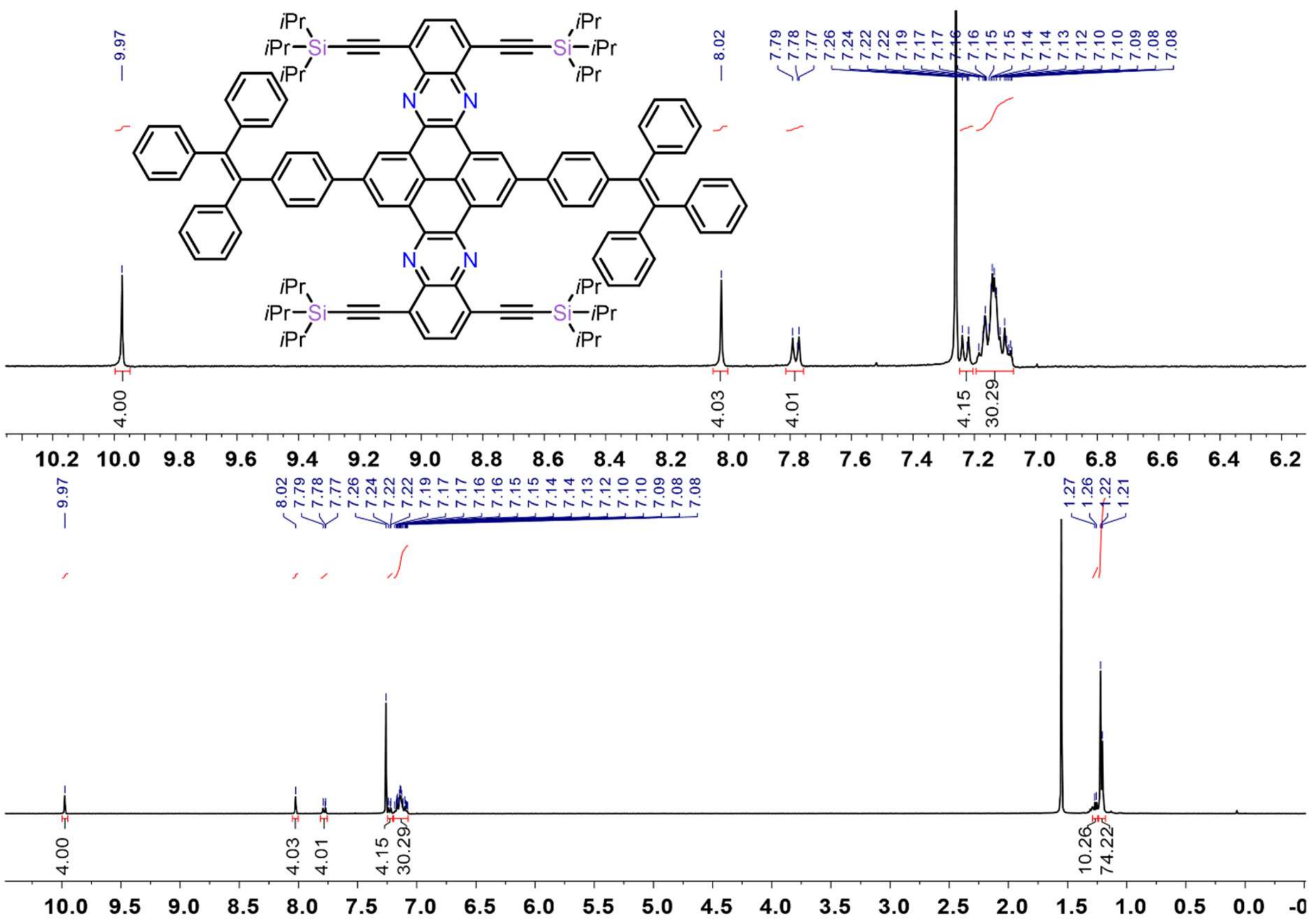

Figure S23. ^{1}H NMR spectrum ($CDCl_3$, 400 MHz, 298 K) of compound **1**.

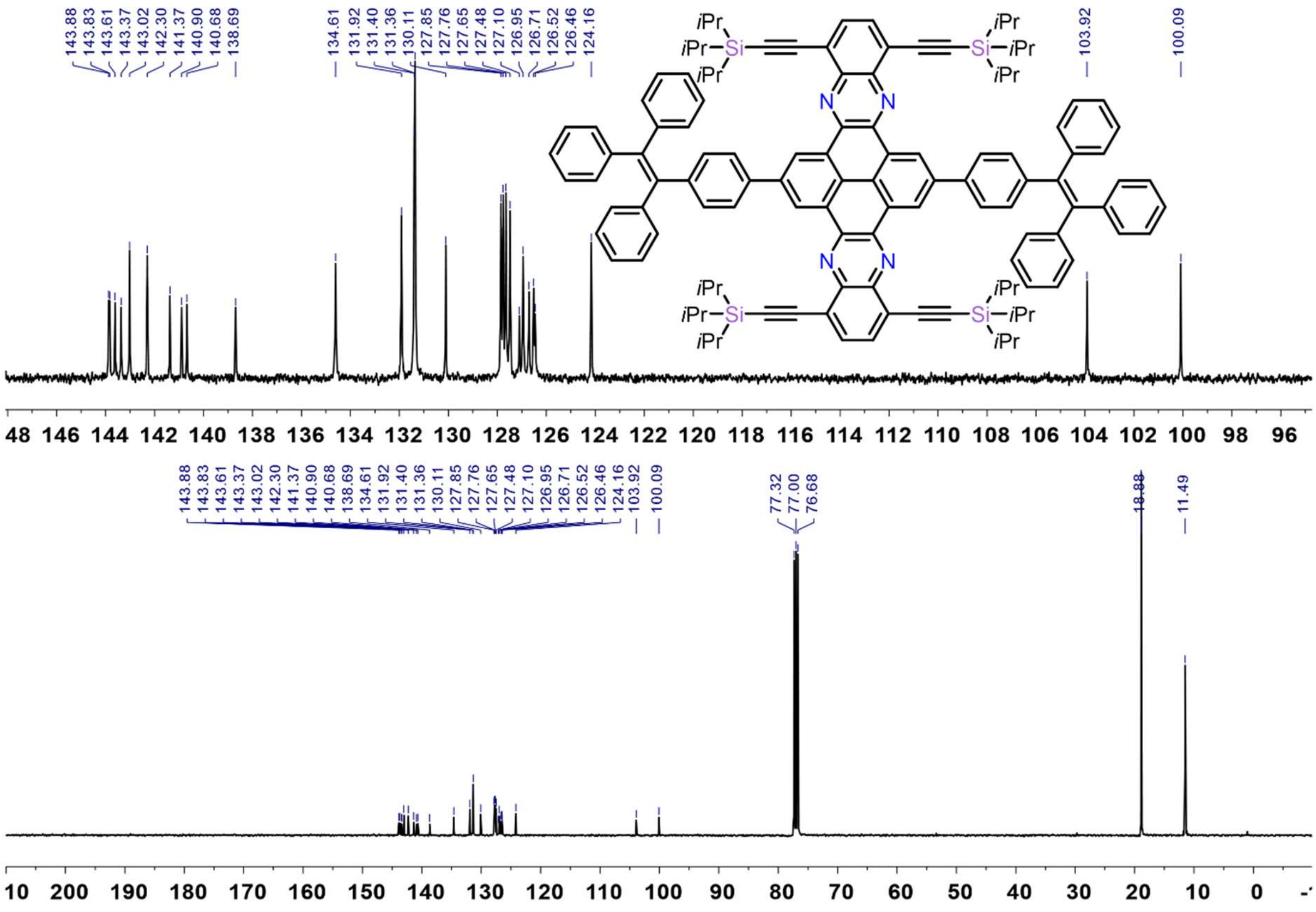

Figure S24. ^{13}C NMR spectrum ($CDCl_3$, 100 MHz, 298 K) of compound **1**.

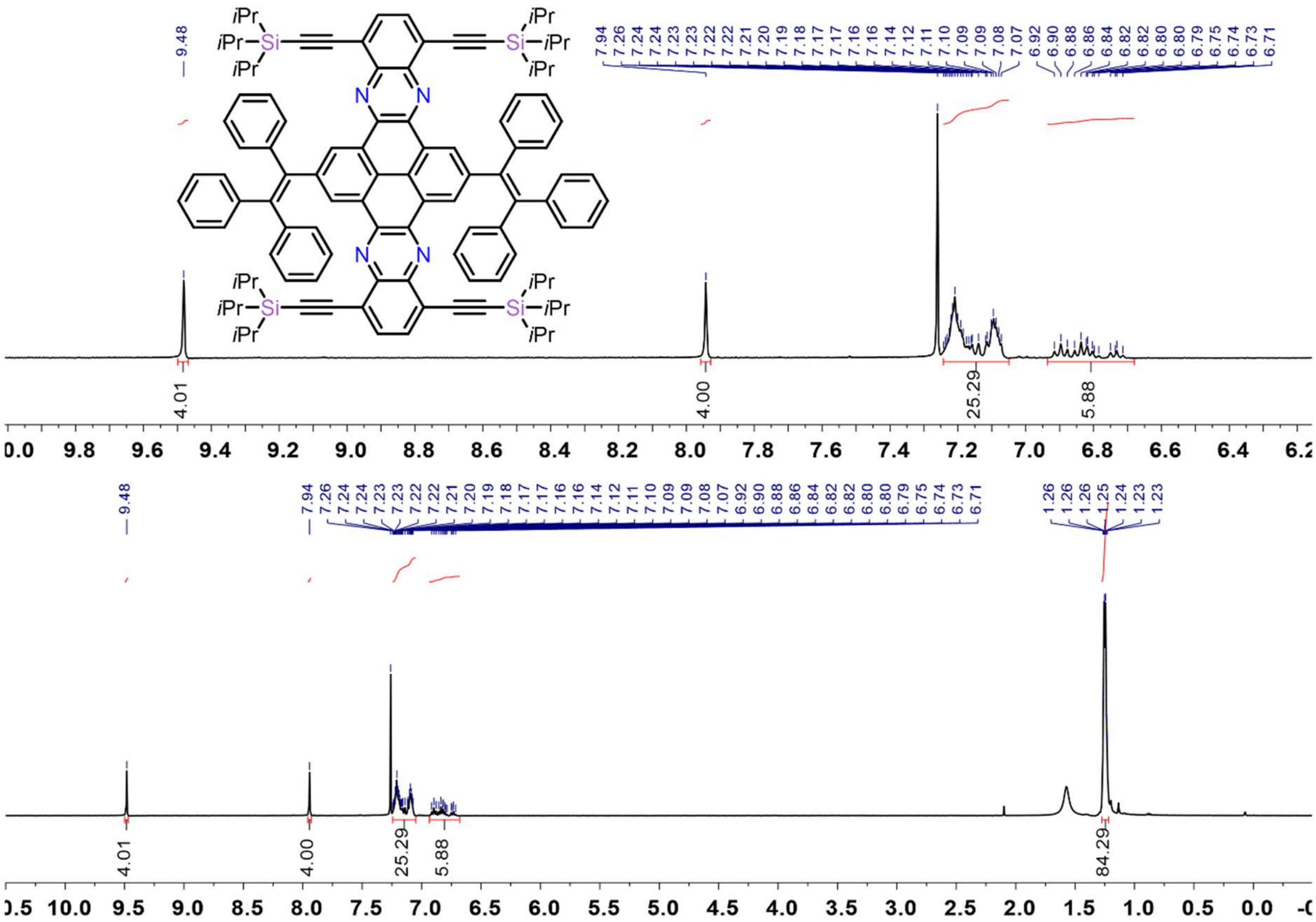

Figure S25. ^{1}H NMR spectrum ($CDCl_3$, 400 MHz, 298 K) of compound **2**.

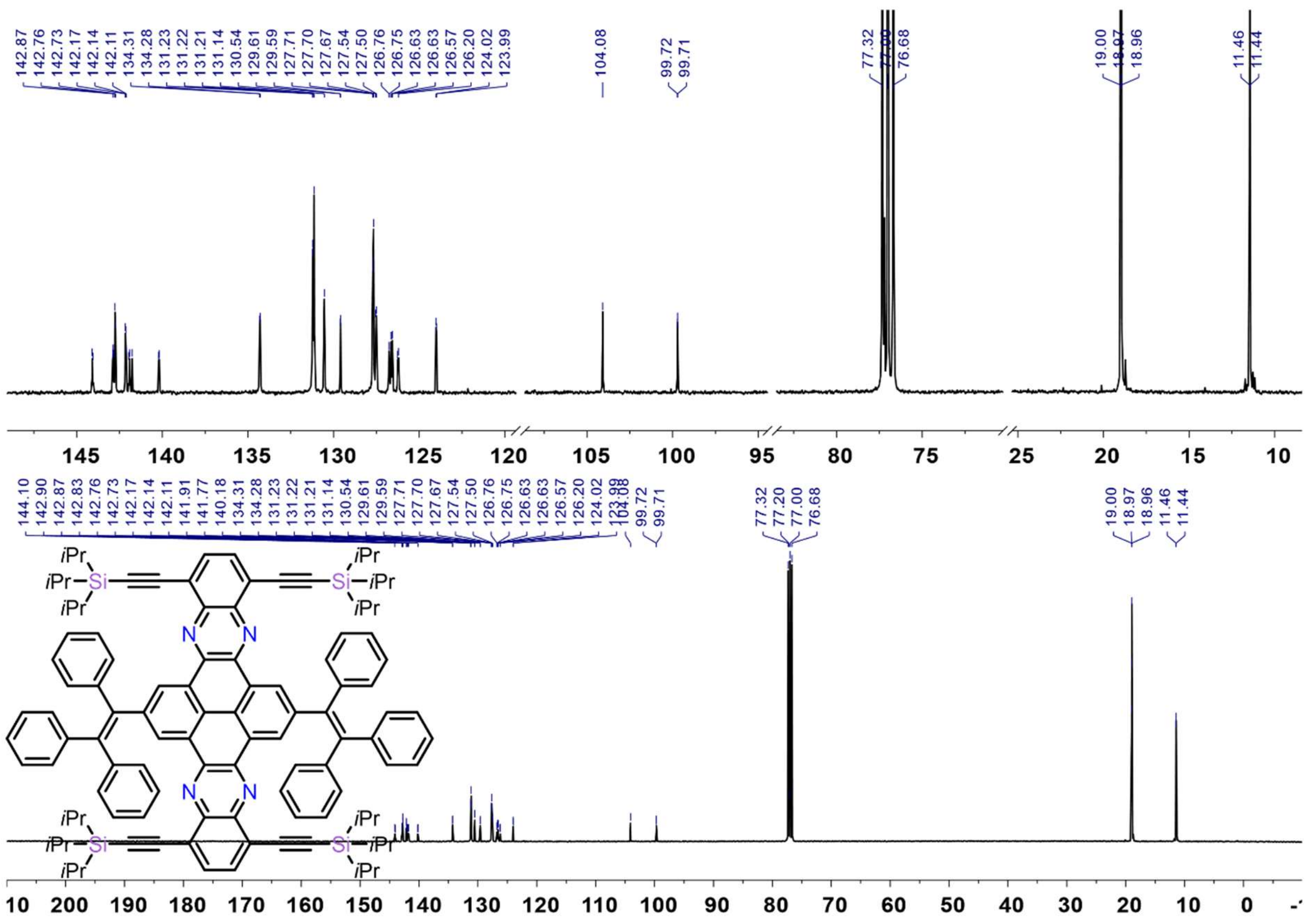

Figure S26. ^{13}C NMR spectrum ($CDCl_3$, 100 MHz, 298 K) of compound **2**.

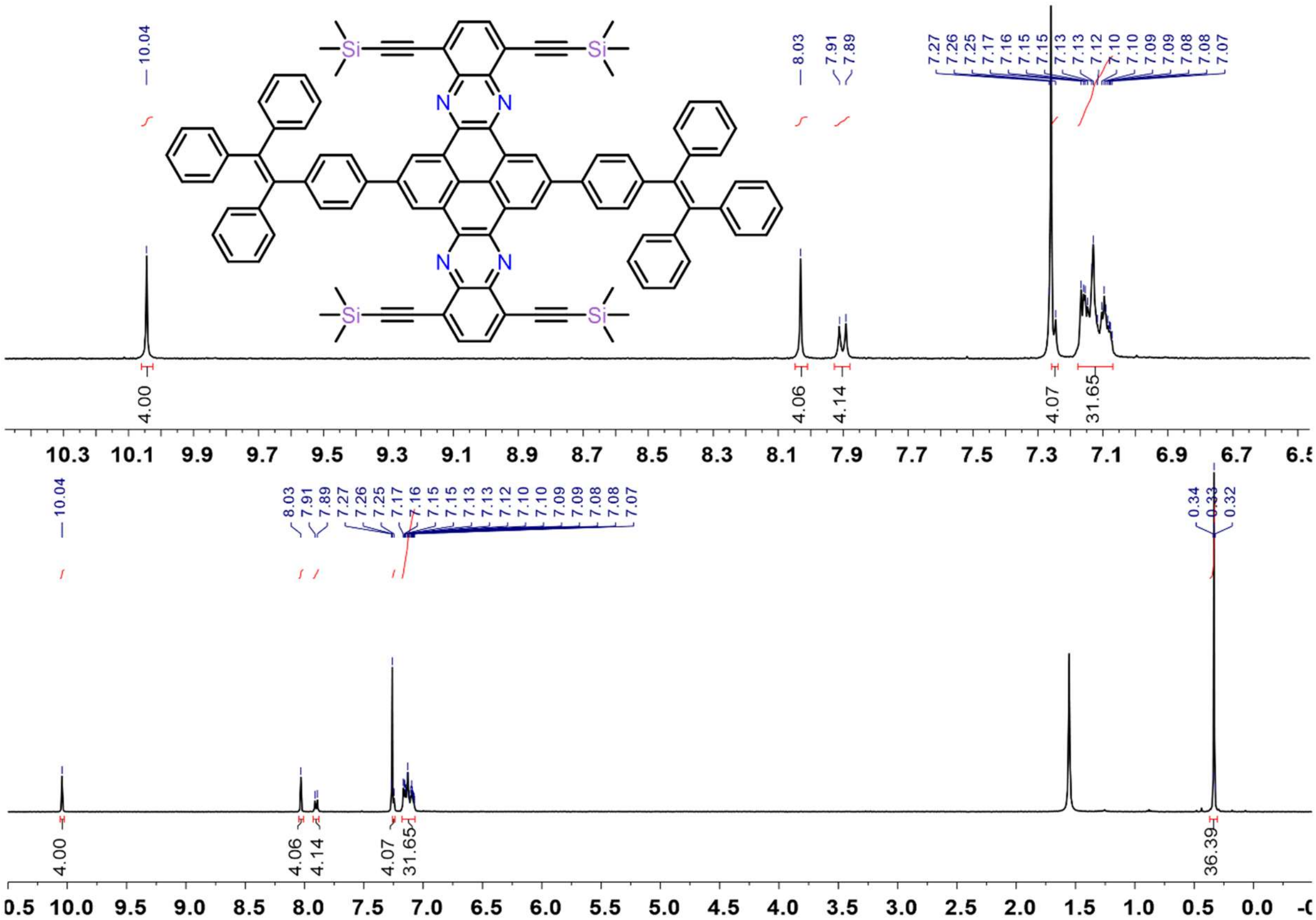

Figure S27. ^{1}H NMR spectrum ($CDCl_3$, 400 MHz, 298 K) of compound **3**.

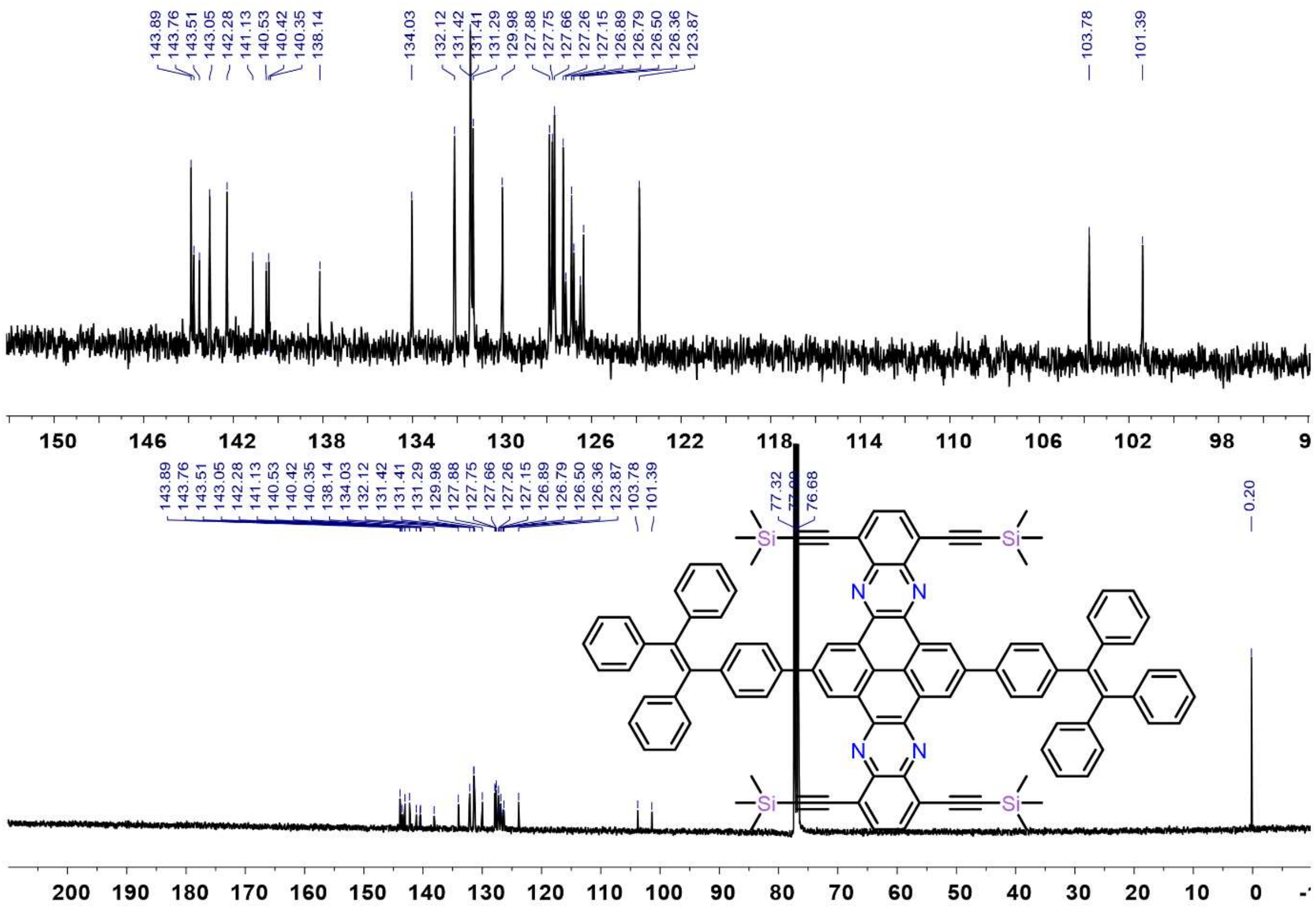

Figure S28. ^{13}C NMR spectrum ($CDCl_3$, 100 MHz, 298 K) of compound **3**.

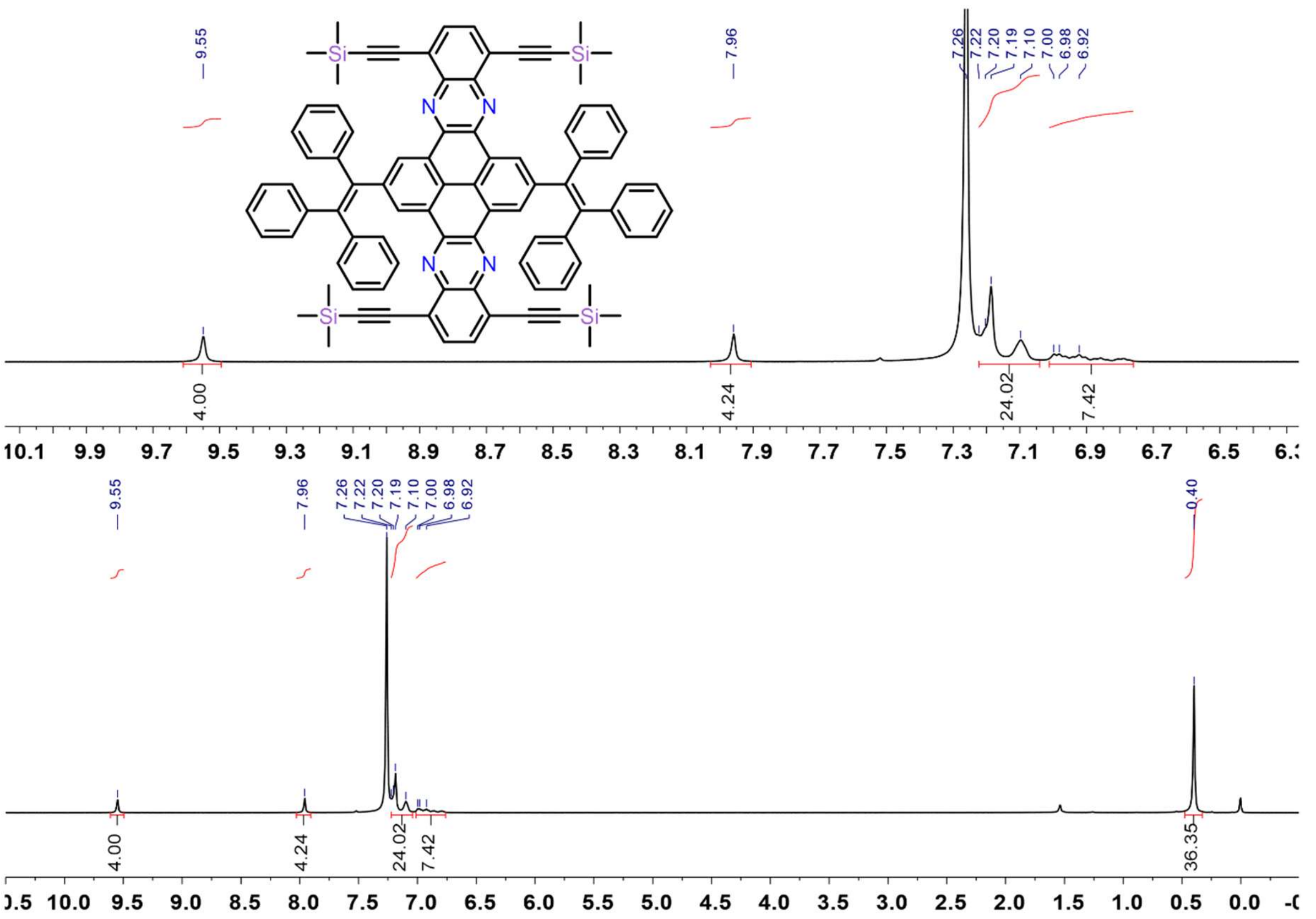

Figure S29. ^{1}H NMR spectrum ($CDCl_3$, 400 MHz, 298 K) of compound **4**.

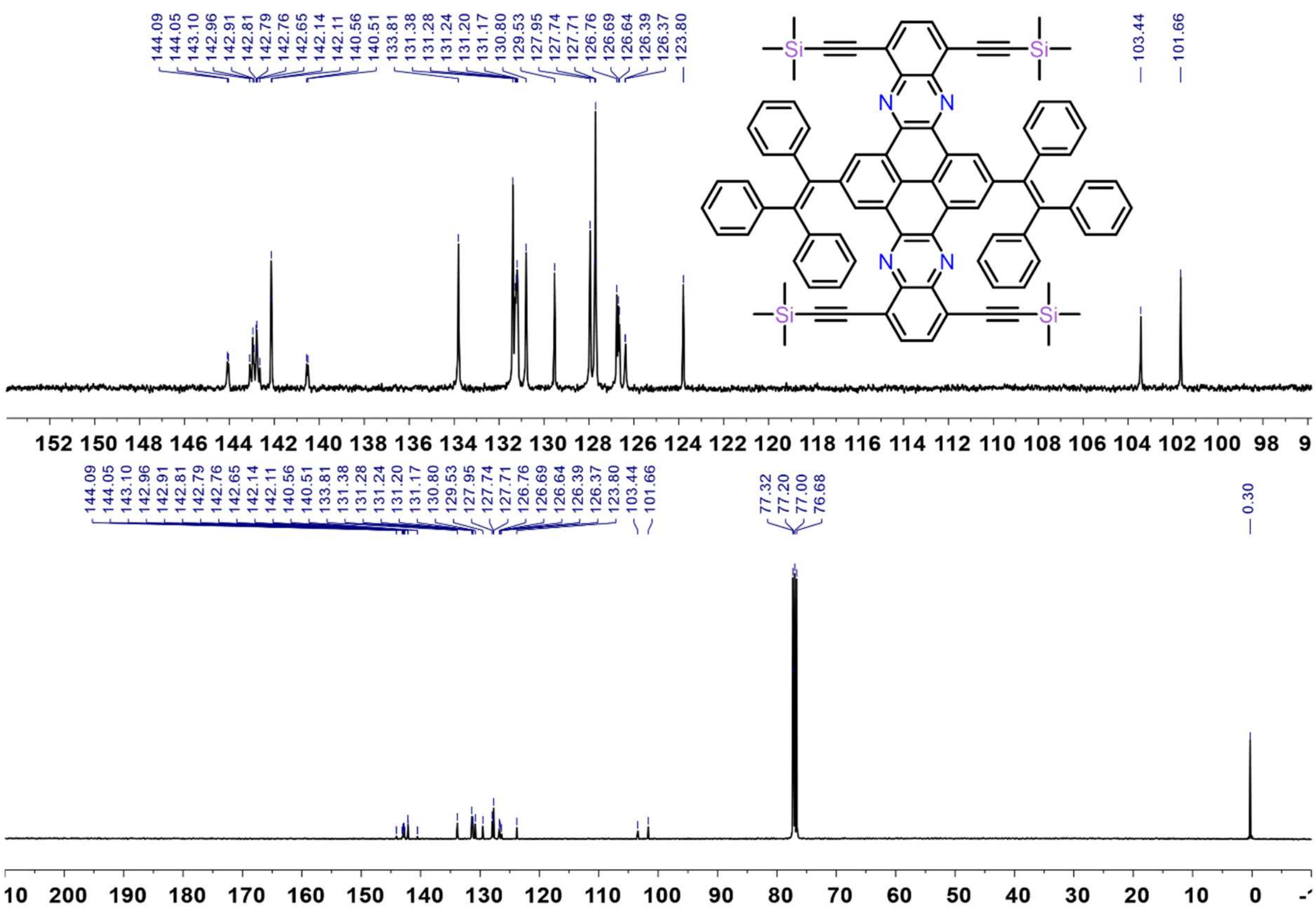

Figure S30. ^{13}C NMR spectrum ($CDCl_3$, 100 MHz, 298 K) of compound **4**.

3. IR Spectra

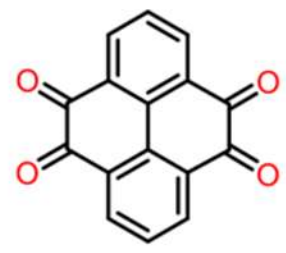

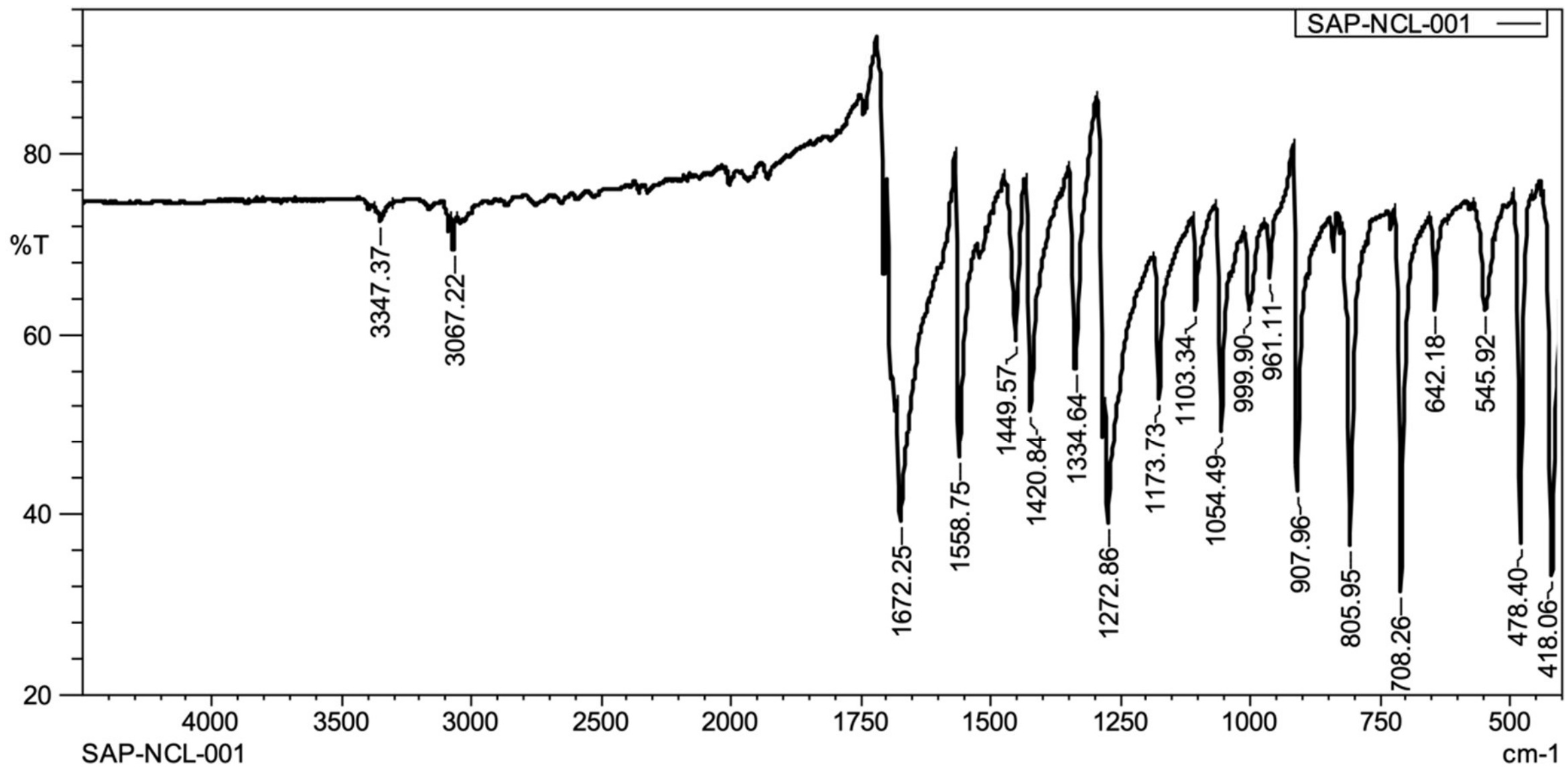

Figure S31. FT-IR spectra (ATR) of compound **S1**.

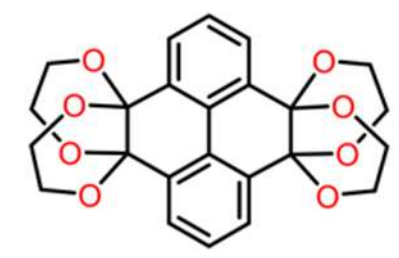

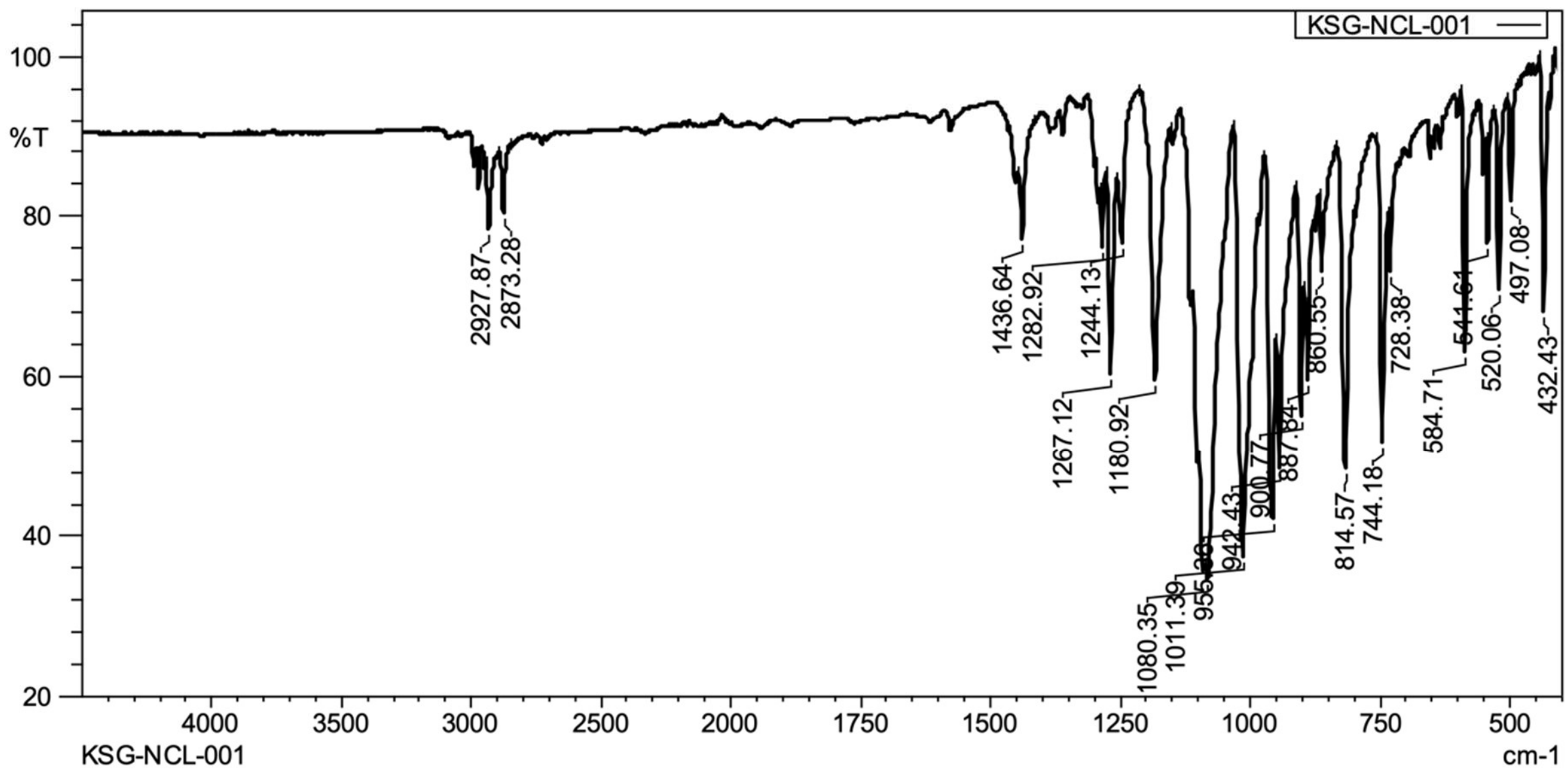

Figure S32. FT-IR spectra (ATR) of compound **S2**.

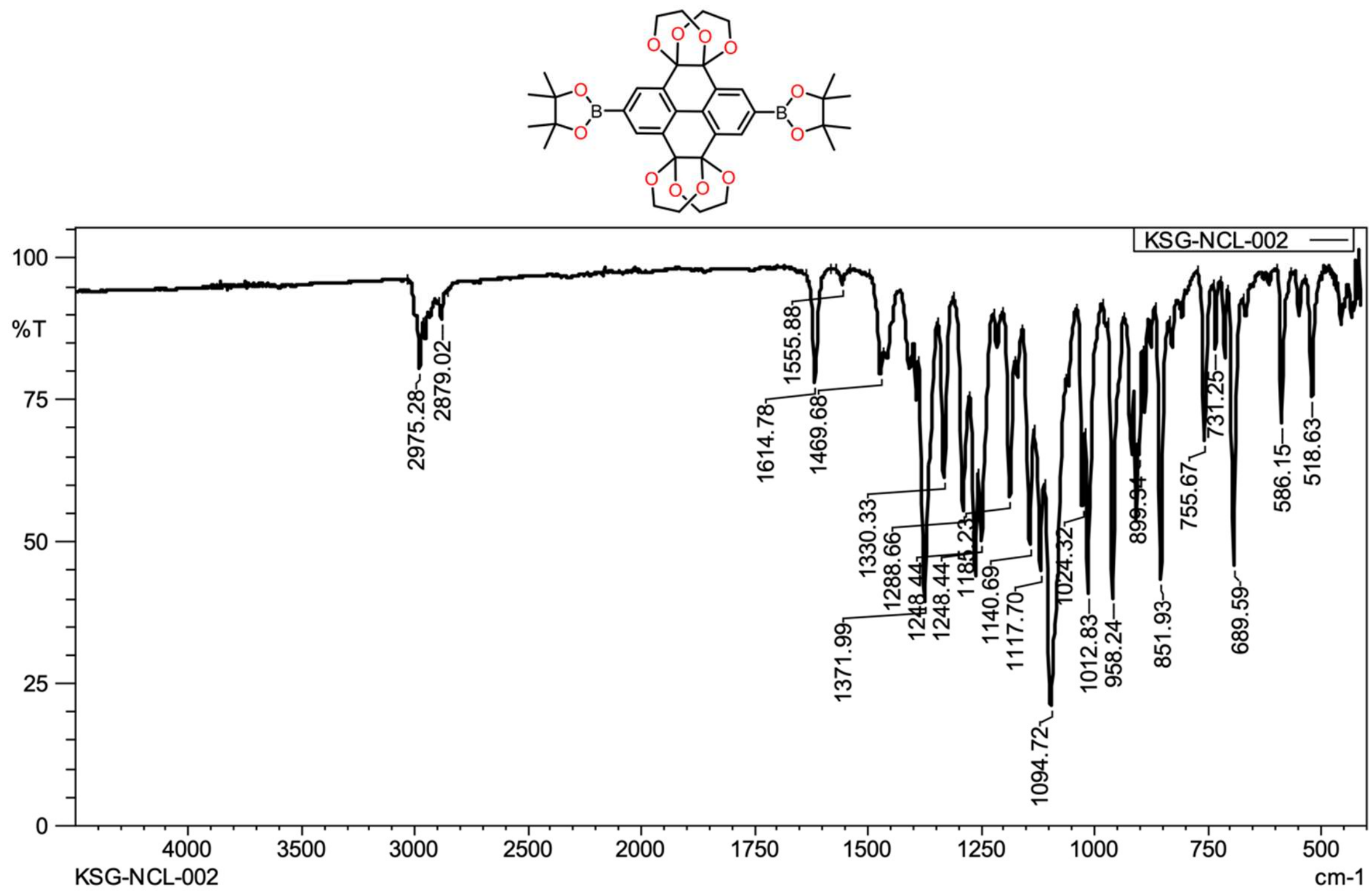

Figure S33. FT-IR spectra (ATR) of compound **5**.

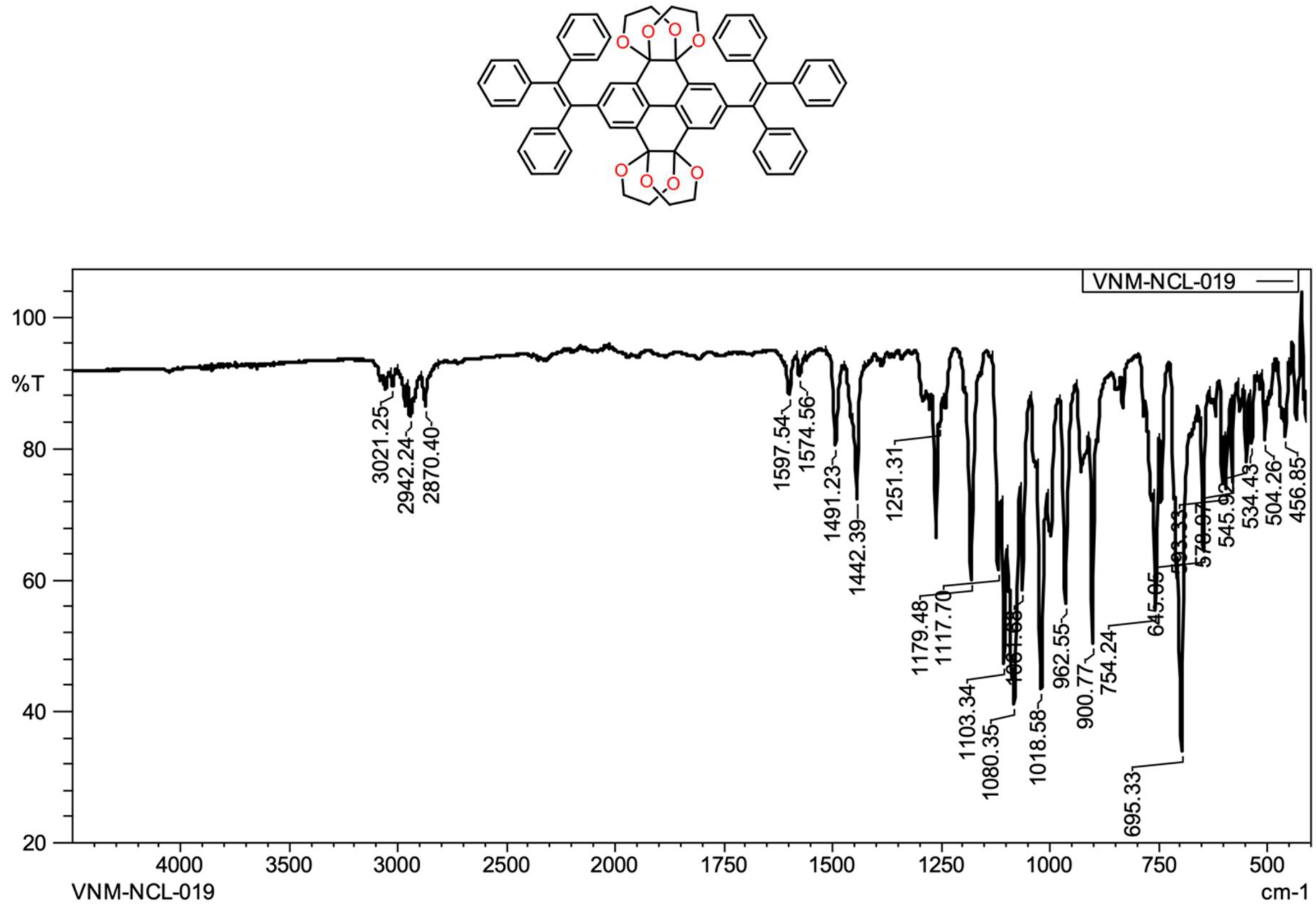

Figure S34. FT-IR spectra (ATR) of compound **6a**.

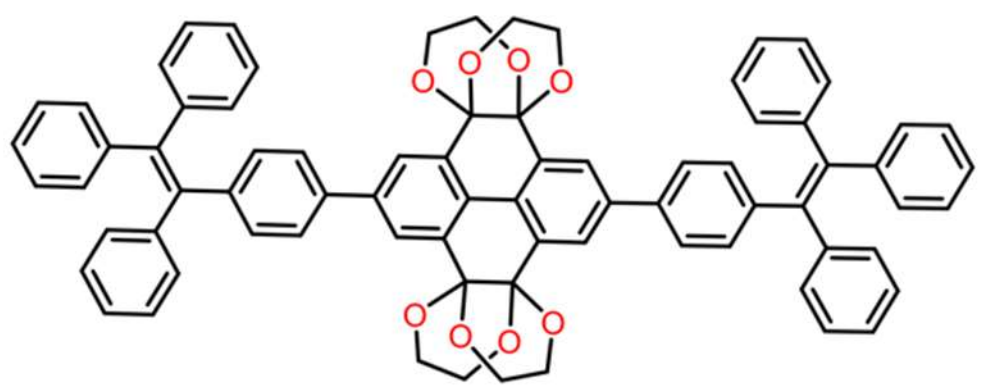

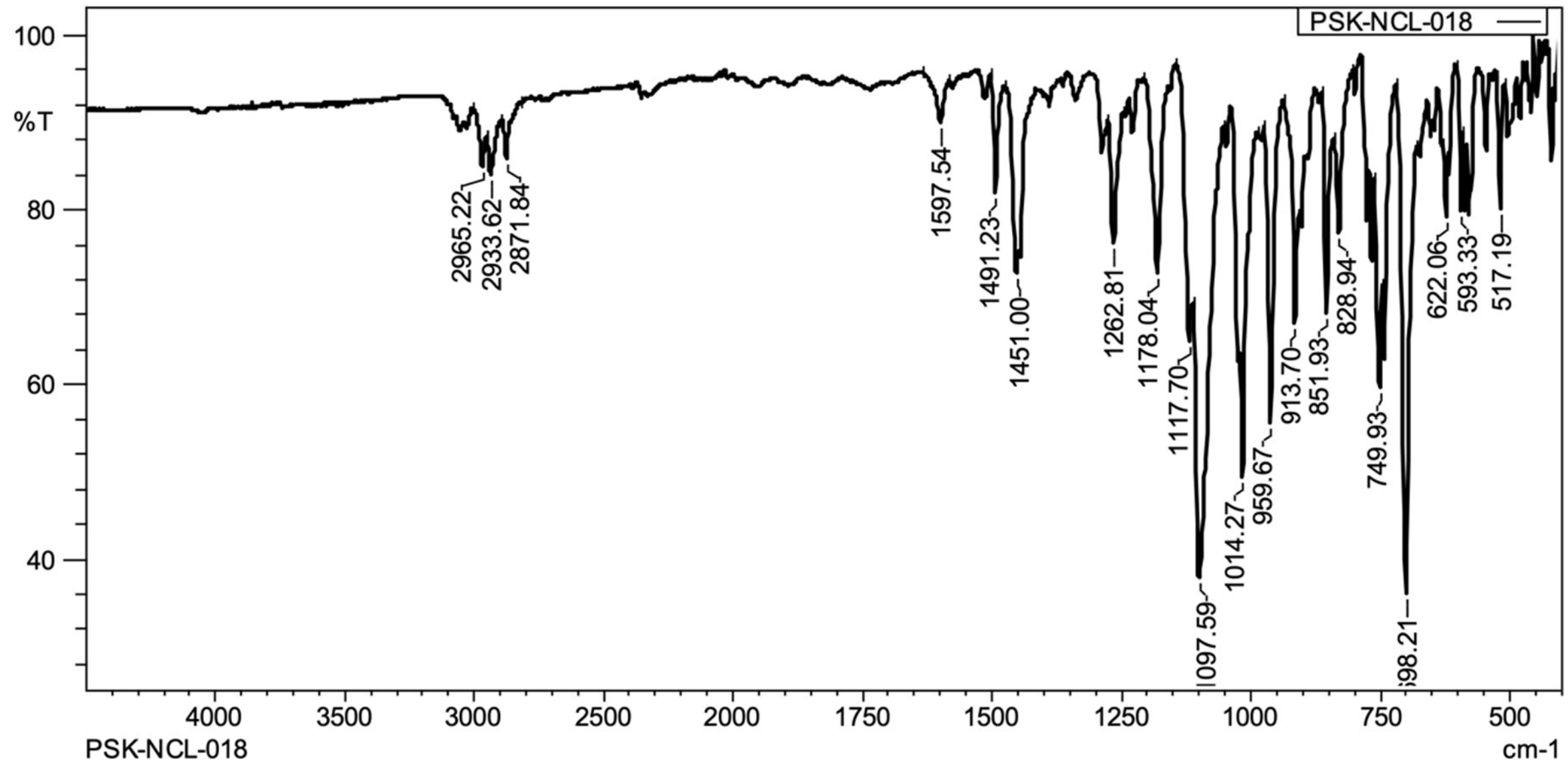

Figure S35. FT-IR spectra (ATR) of compound **6b**.

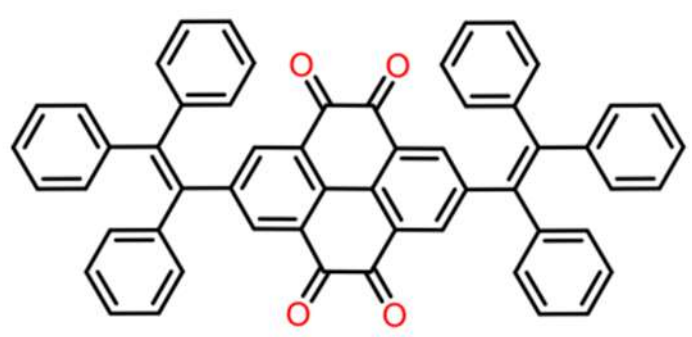

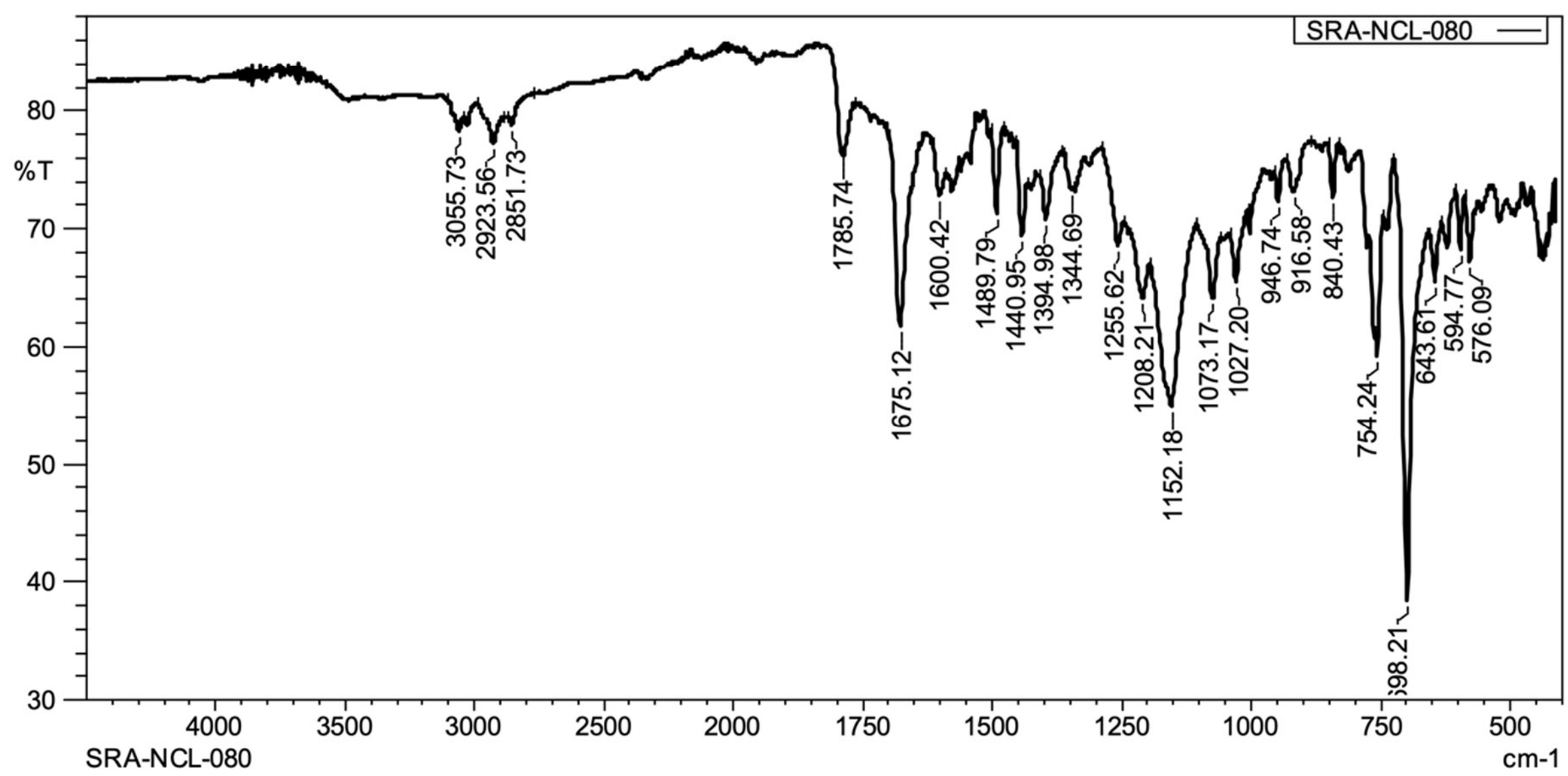

Figure S36. FT-IR spectra (ATR) of compound **7a**.

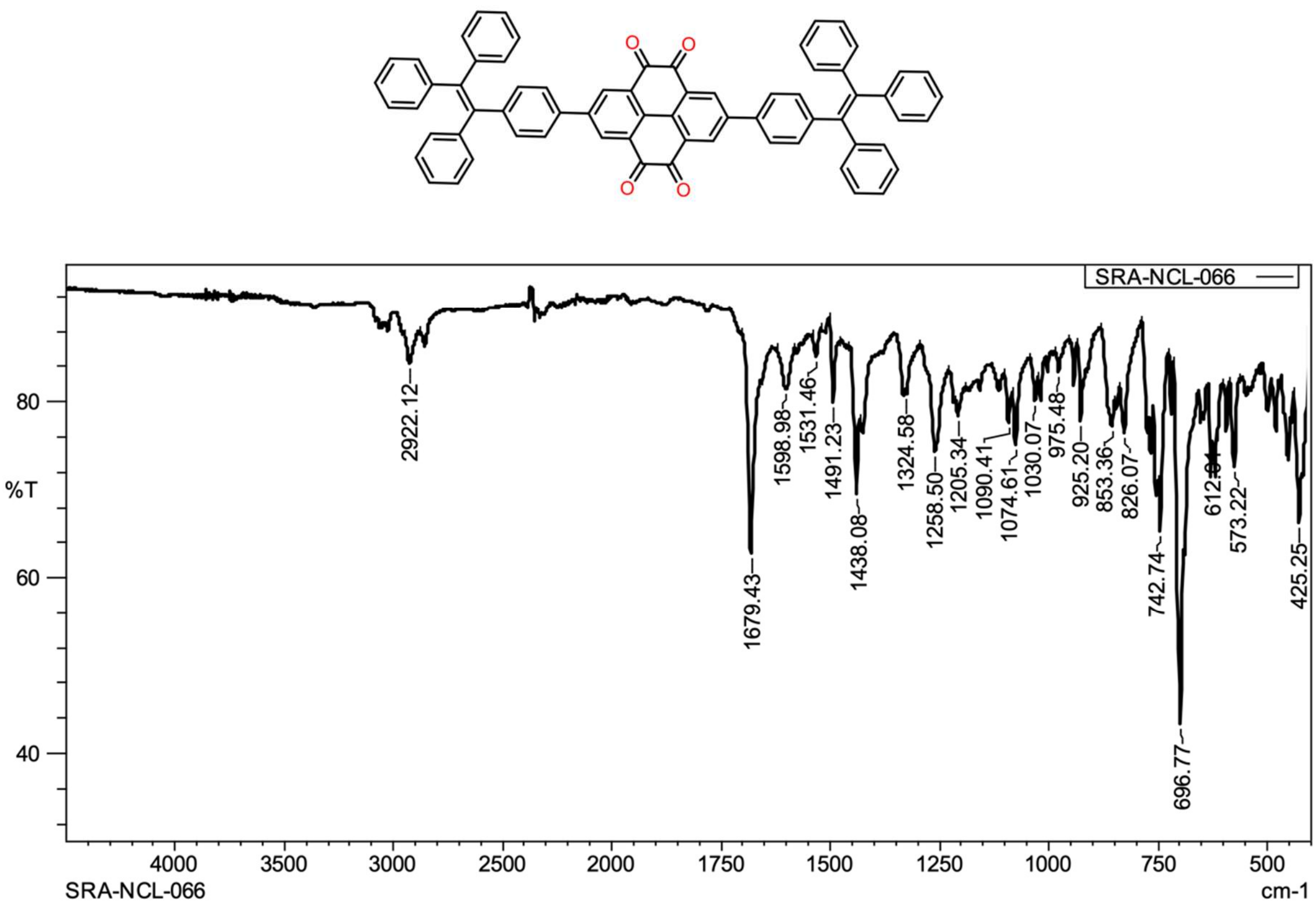

Figure S37. FT-IR spectra (ATR) of compound **7b**.

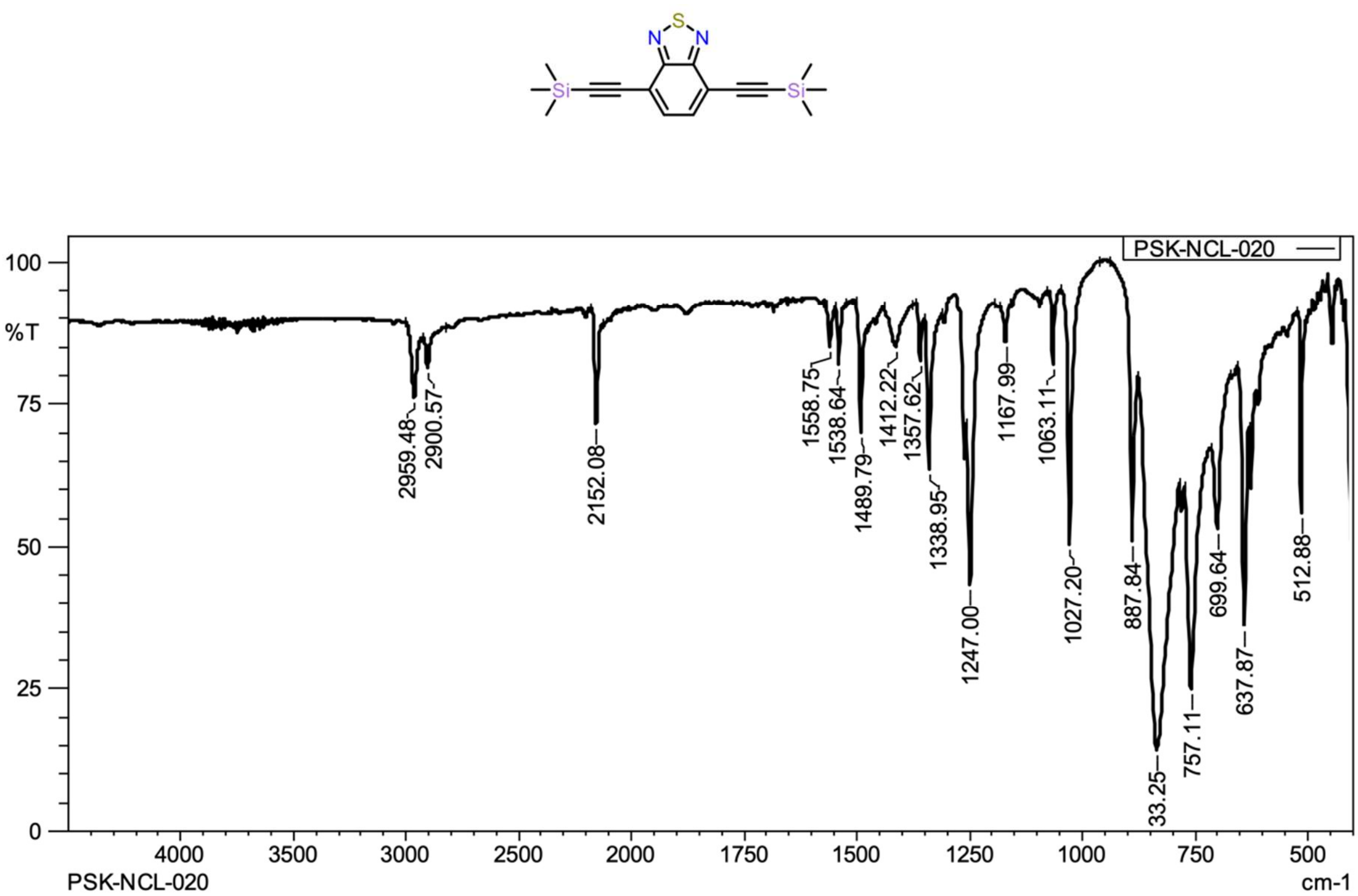

Figure S38. FT-IR spectra (ATR) of compound **S4a**.

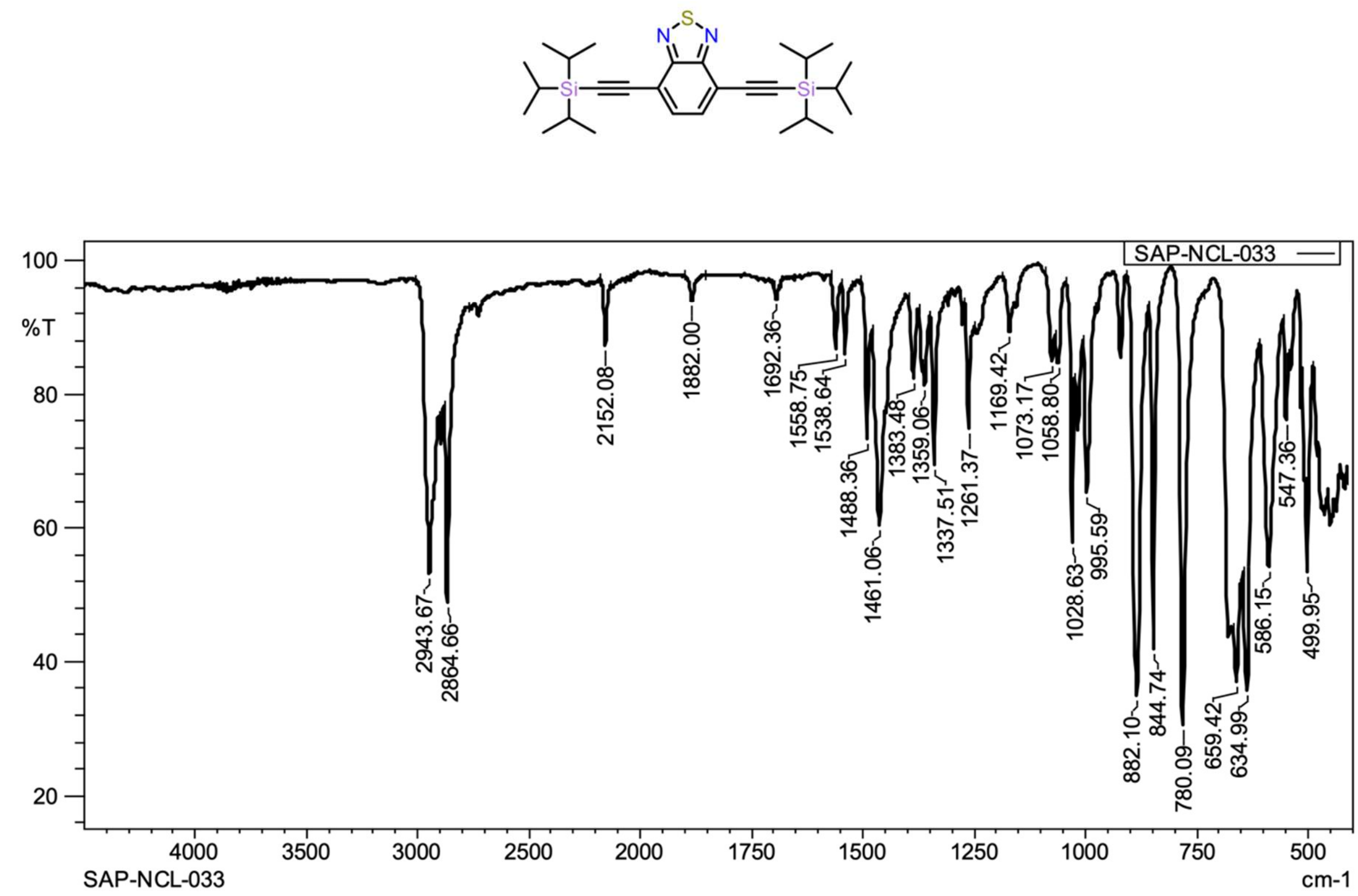

Figure S39. FT-IR spectra (ATR) of compound **S4b** in $CHCl_3$.

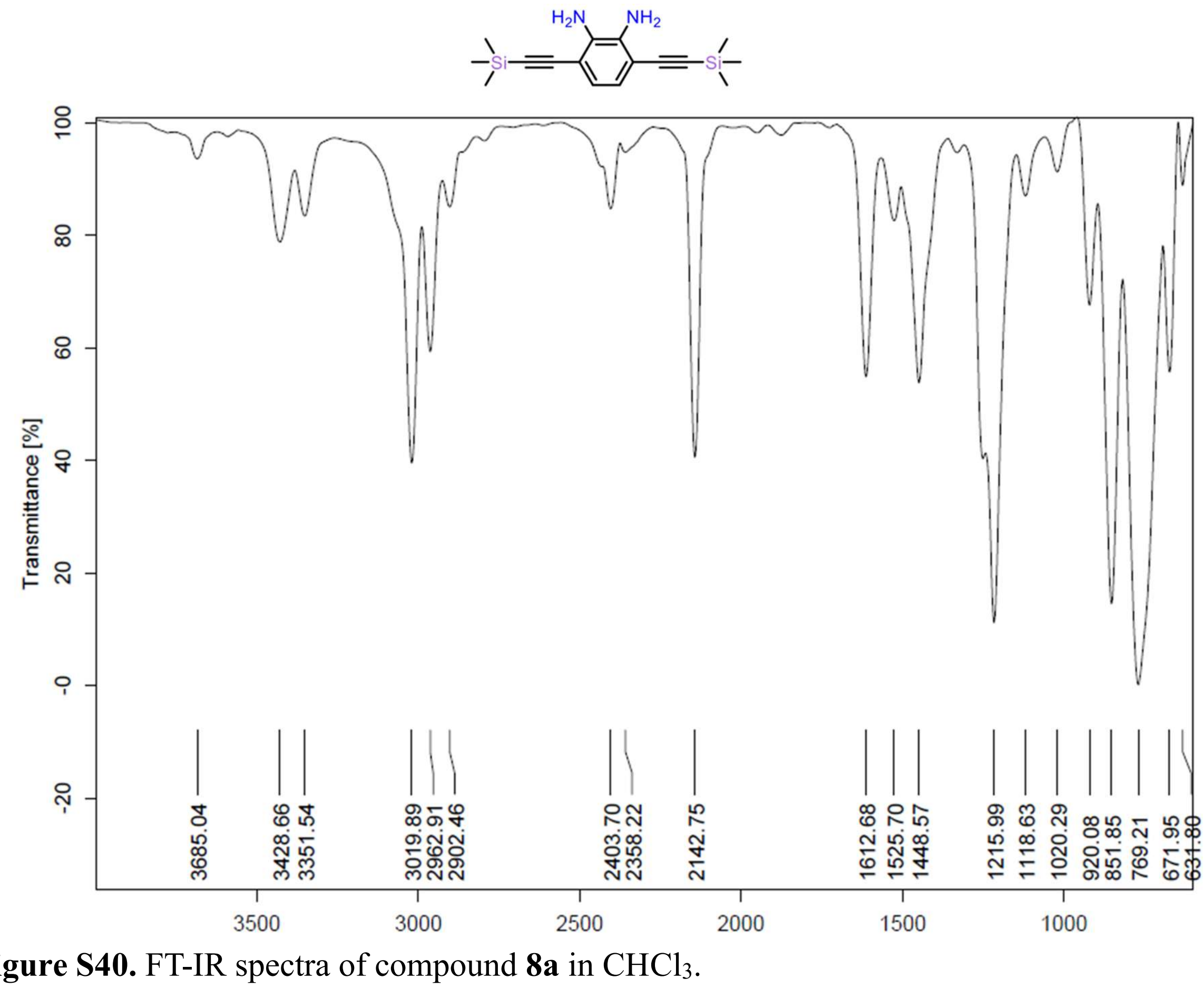

Figure S40. FT-IR spectra of compound **8a** in $CHCl_3$.

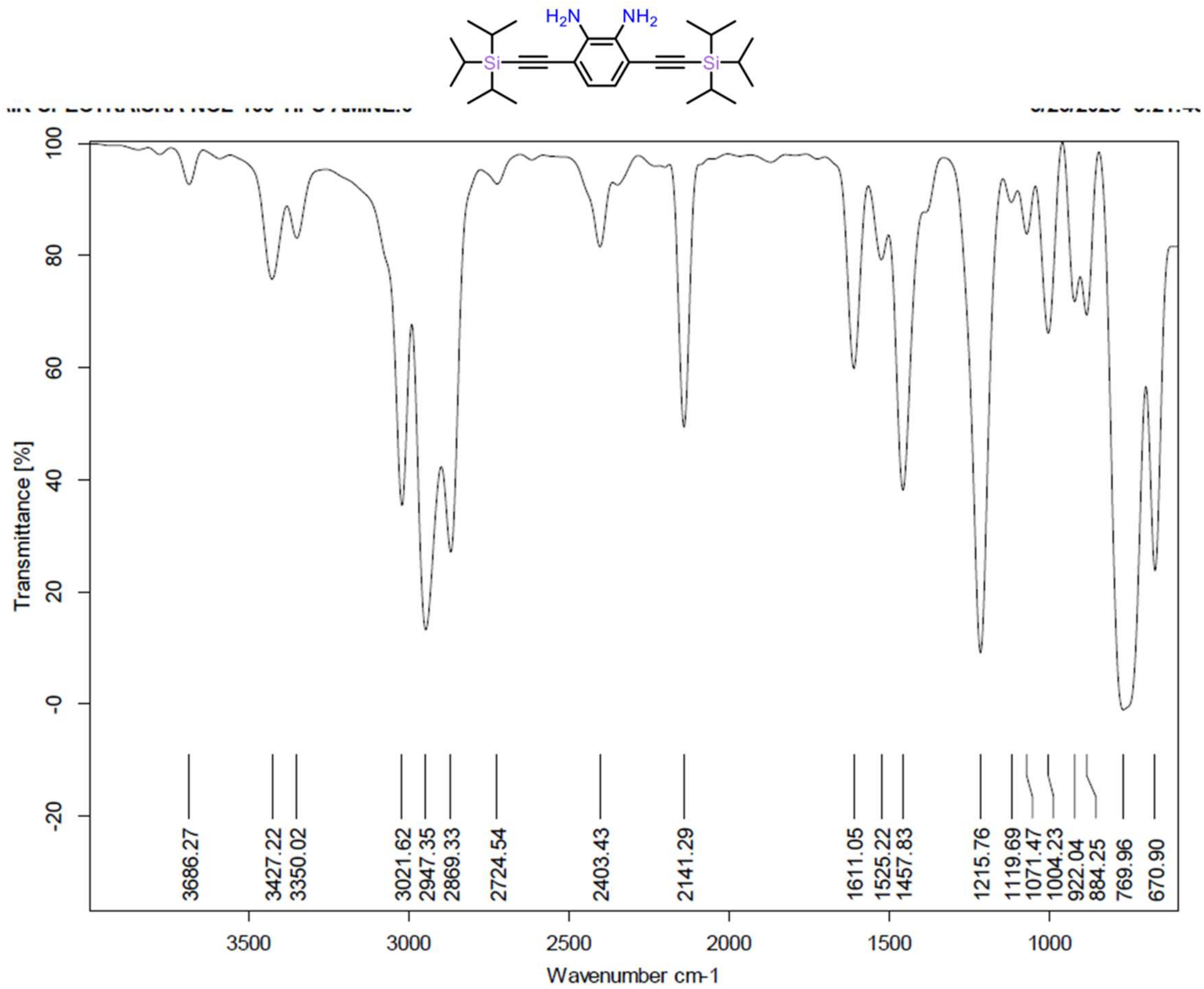

Figure S41. FT-IR spectra of compound **8b** in $CHCl_3$.

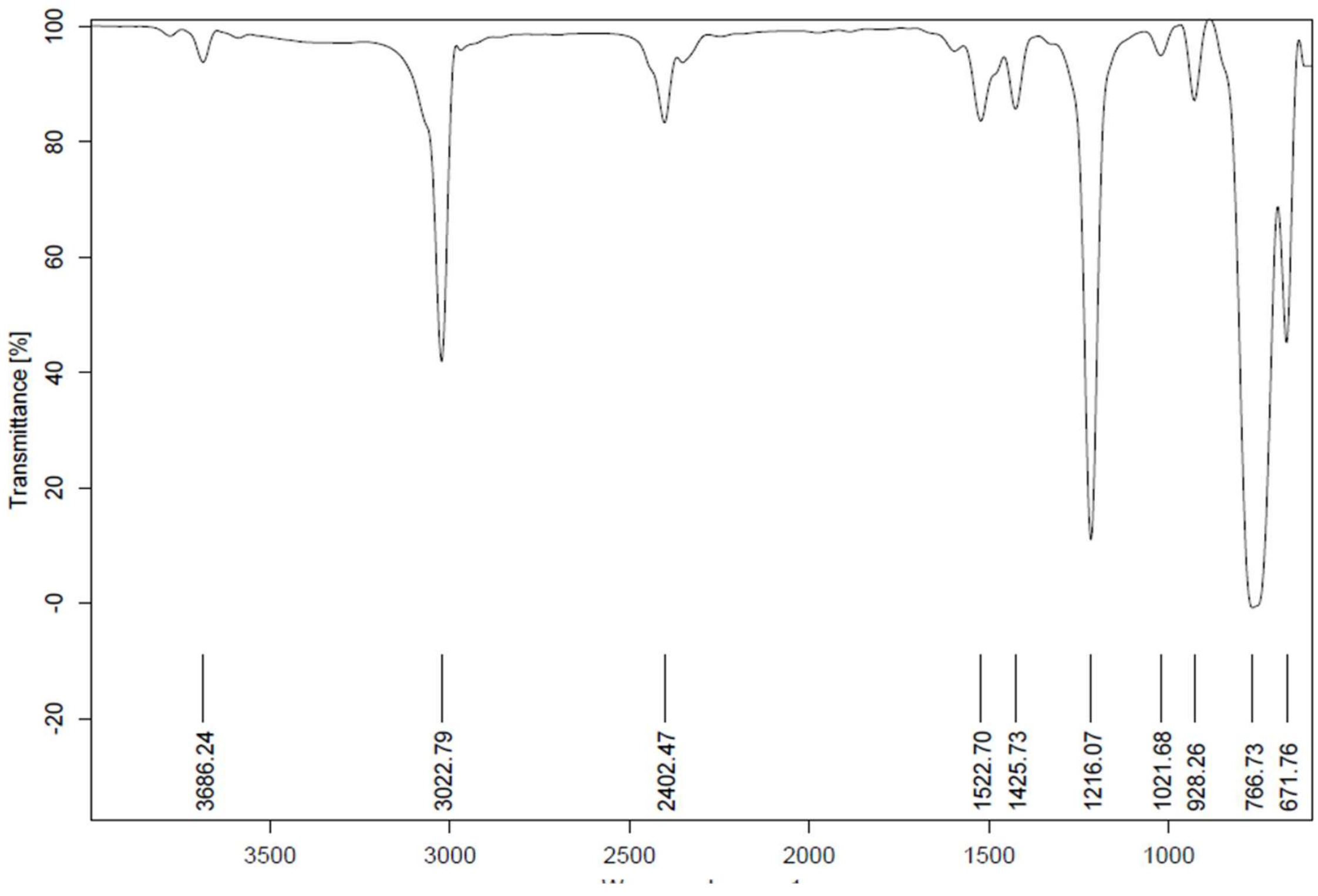

Figure S42. FT-IR spectra of blank $CHCl_3$ measured for **8a** and **8b**.

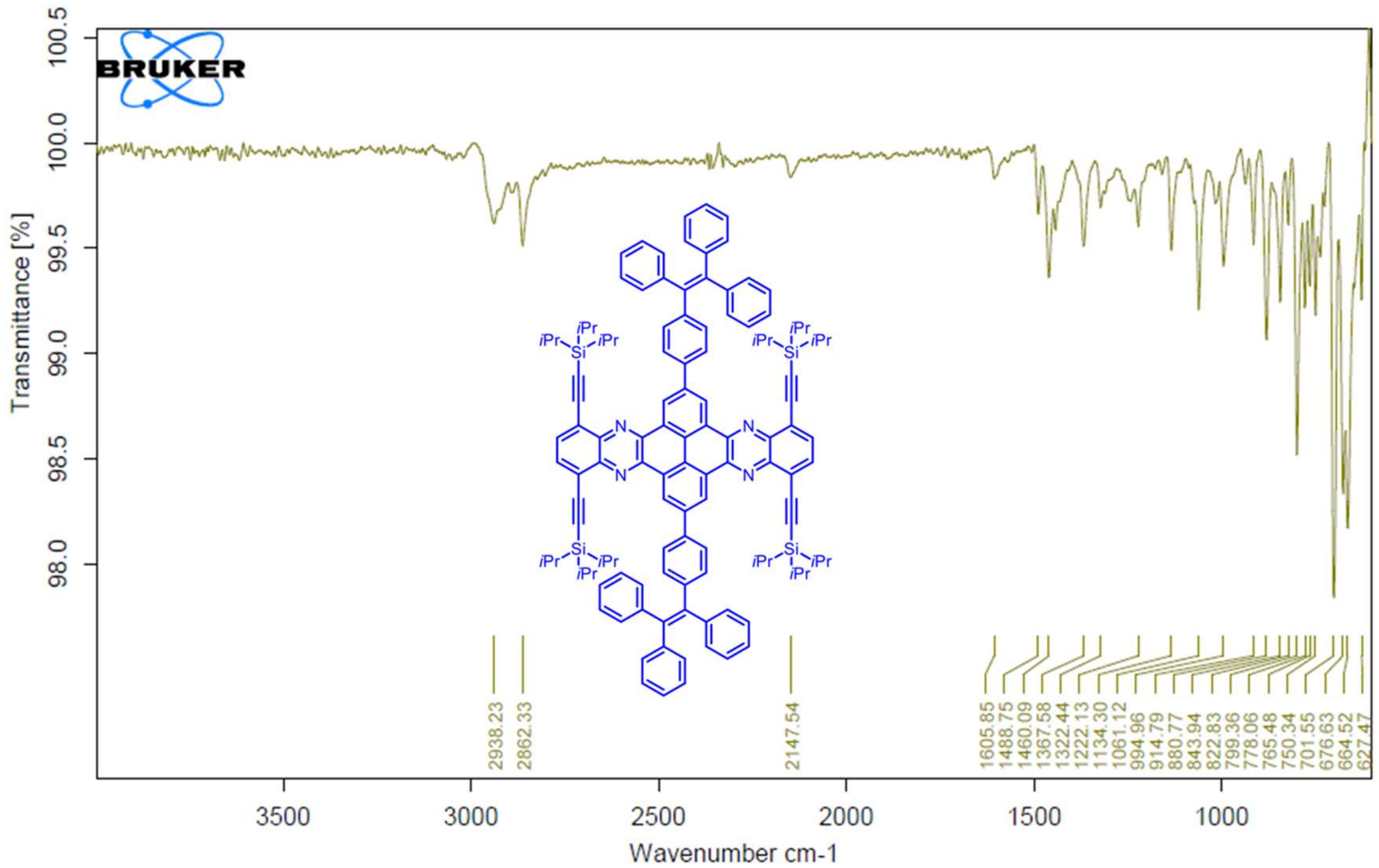

Figure S43. FT-IR spectra (ATR) of compound **1**.

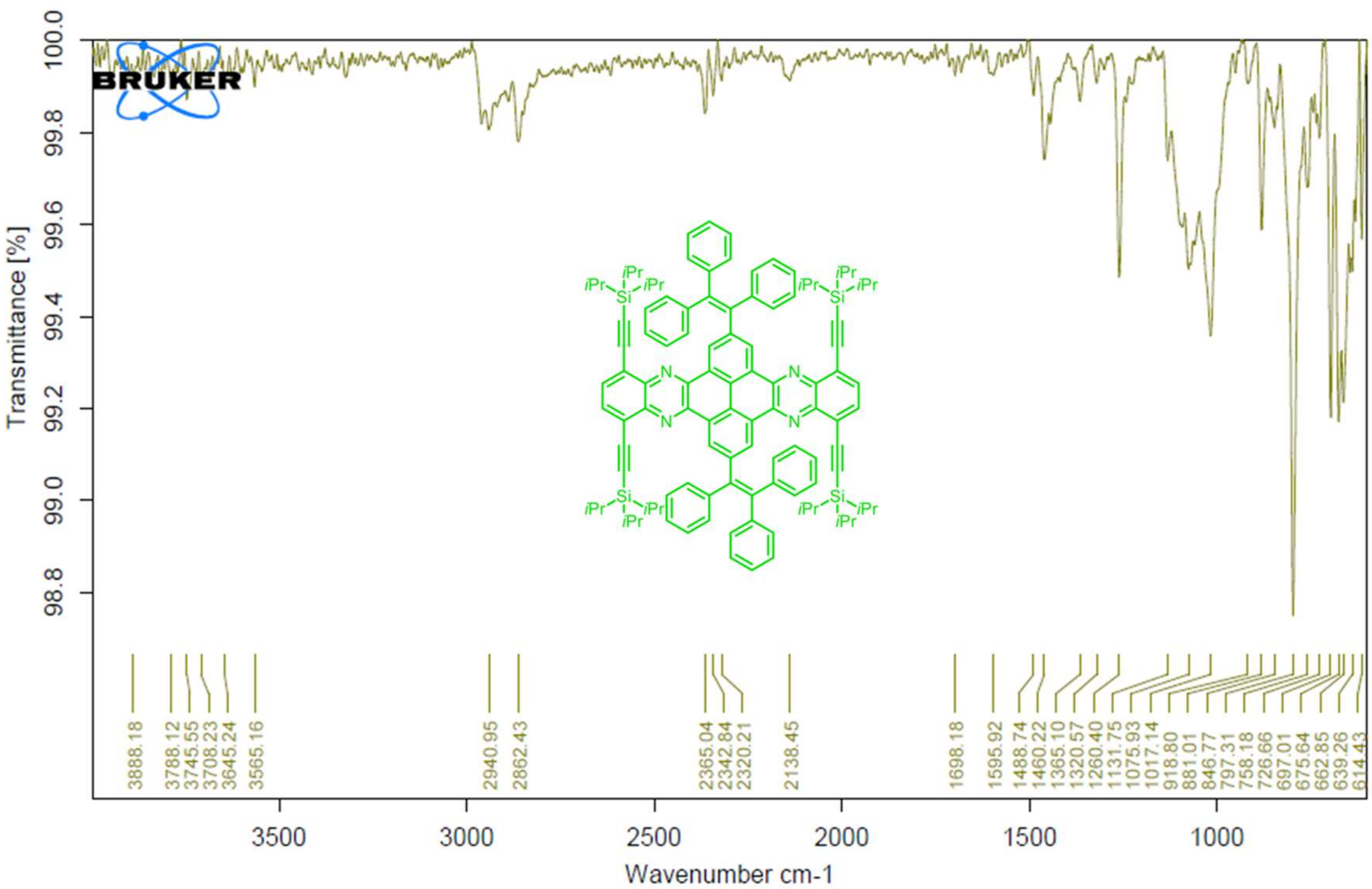

Figure S44. FT-IR spectra (ATR) of compound **2**.

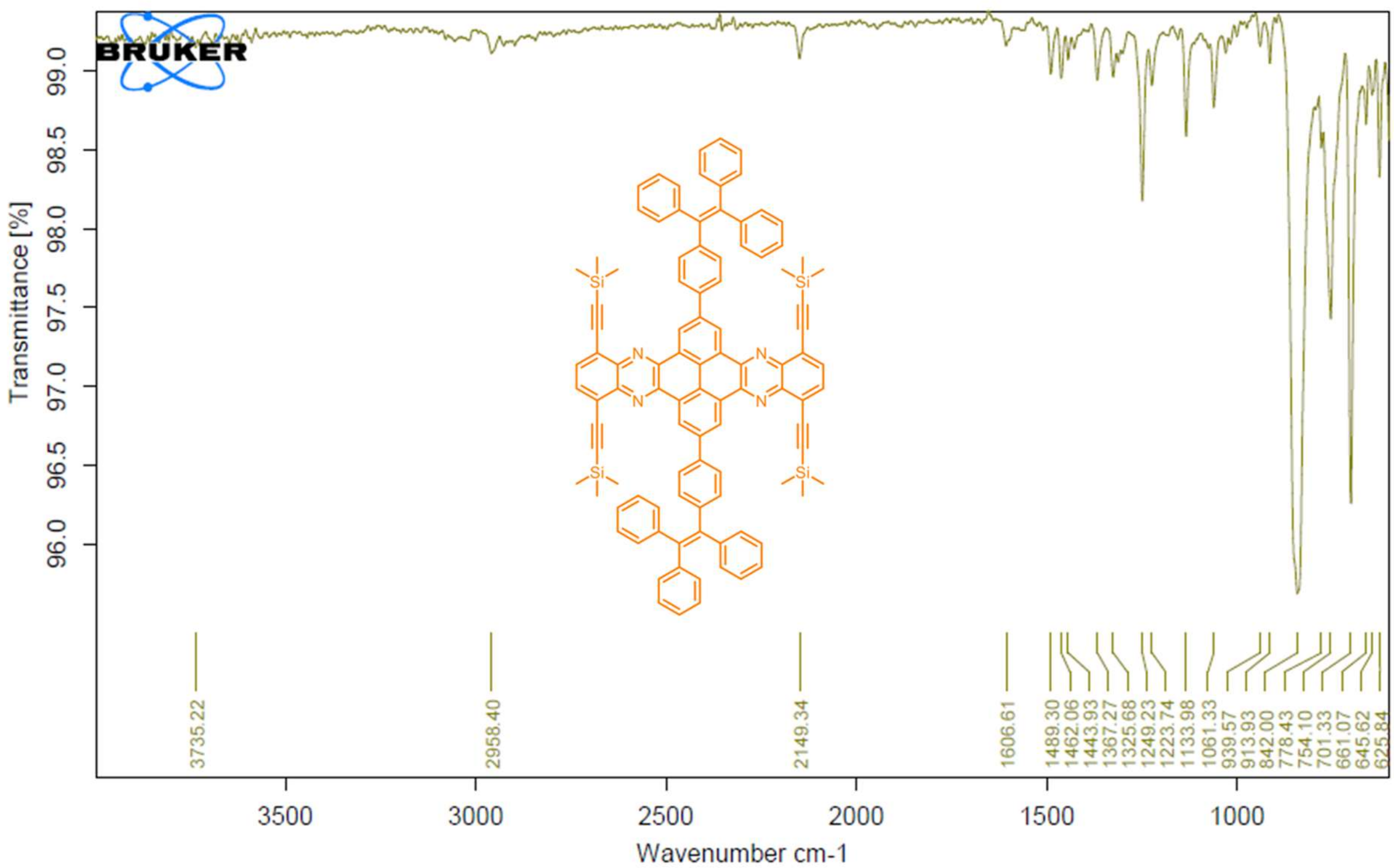

Figure S45. FT-IR spectra (ATR) of compound **3**.

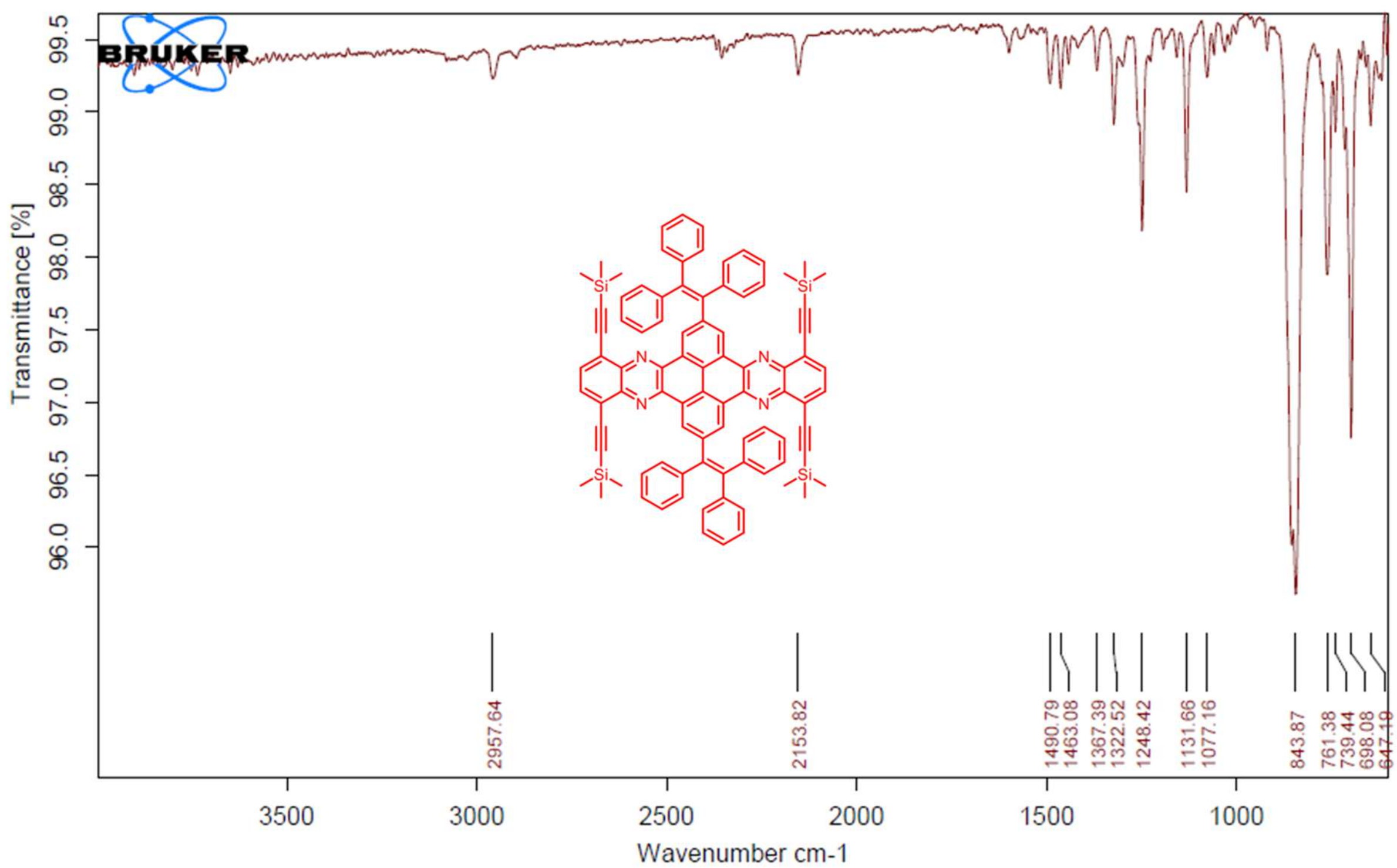

Figure S46. FT-IR spectra (ATR) of compound **4**.

4. MALDI-TOF HR-MS Spectra

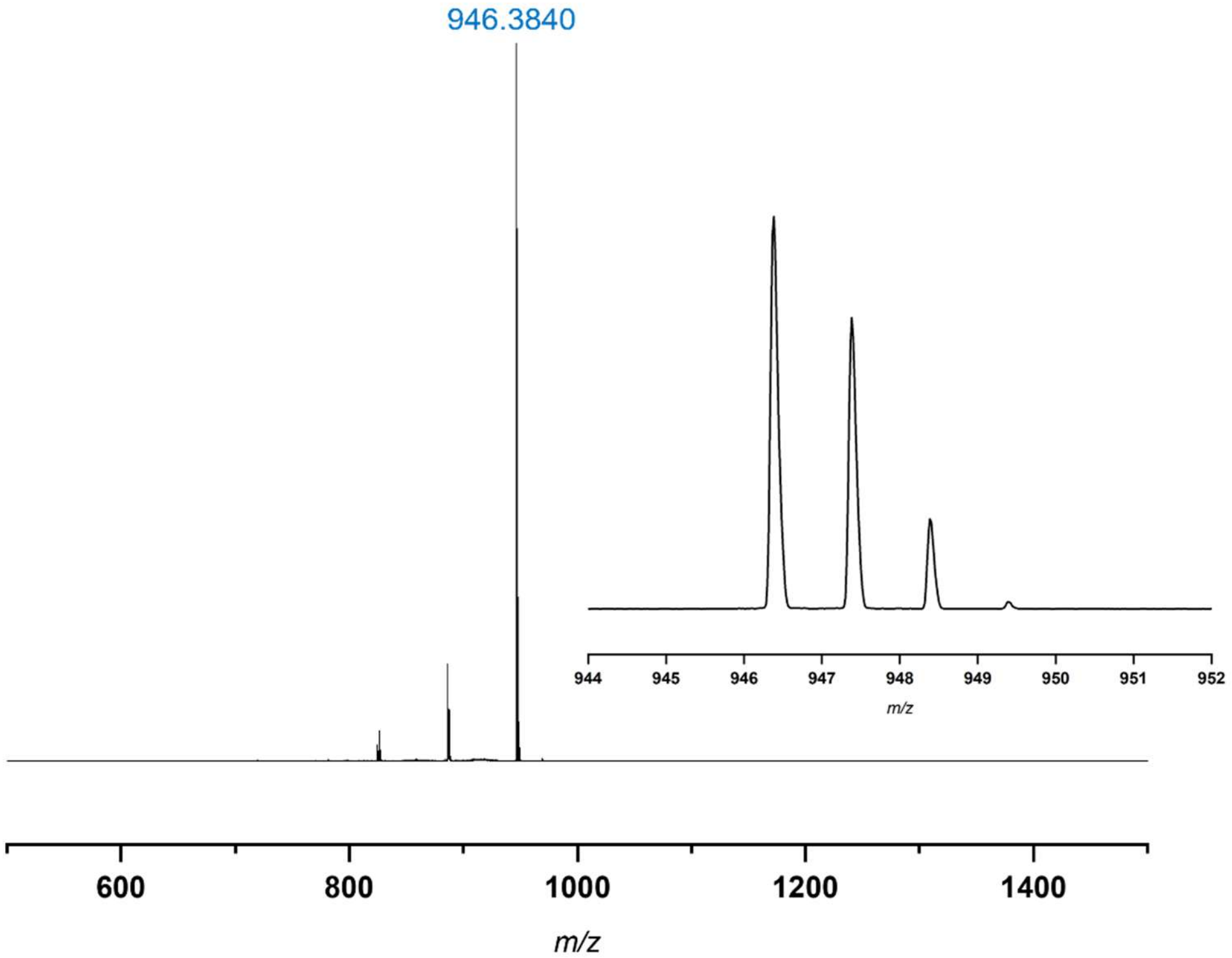

Figure S47. MALDI-TOF spectrum of compound **6a**.

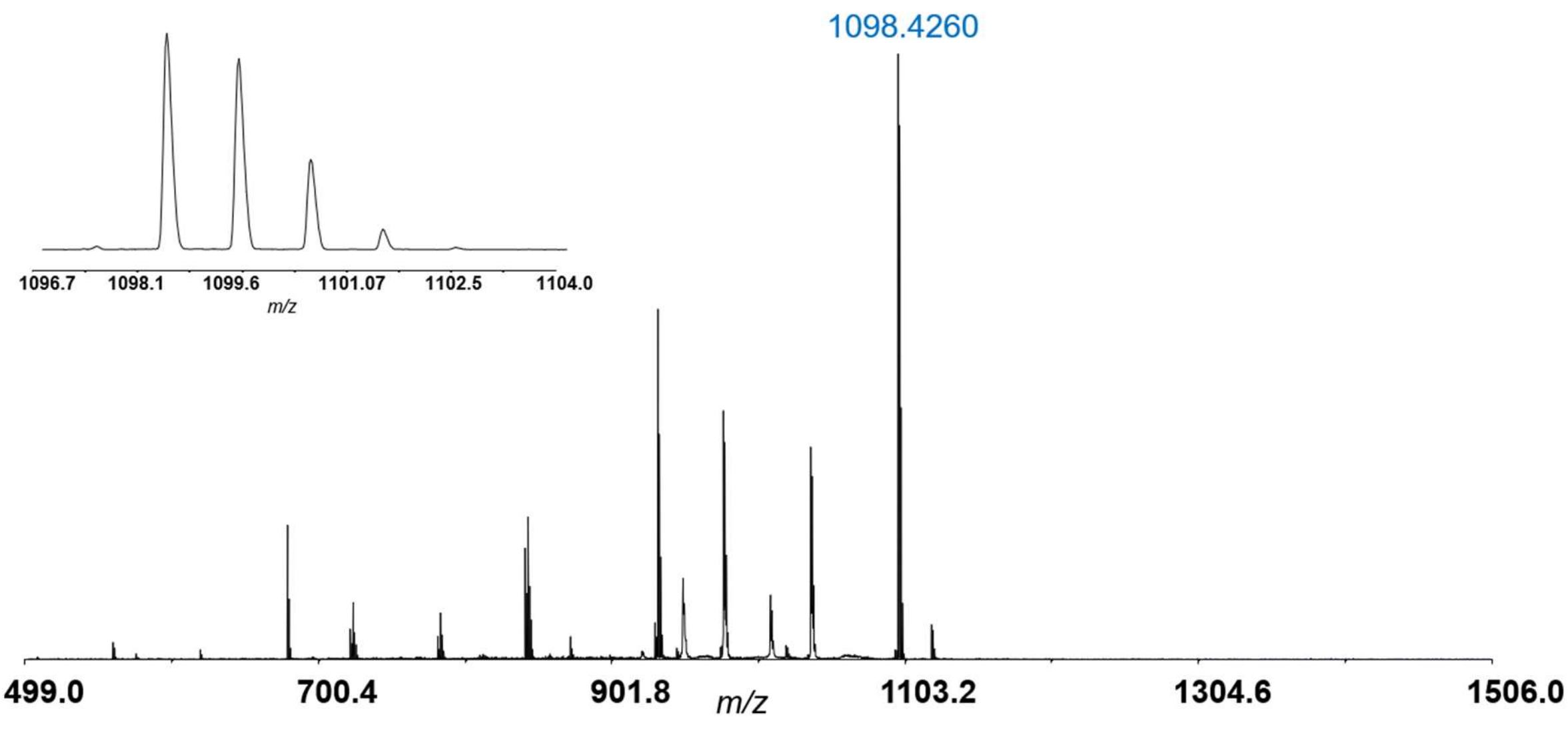

Figure S48. MALDI-TOF spectrum of compound **6b**.

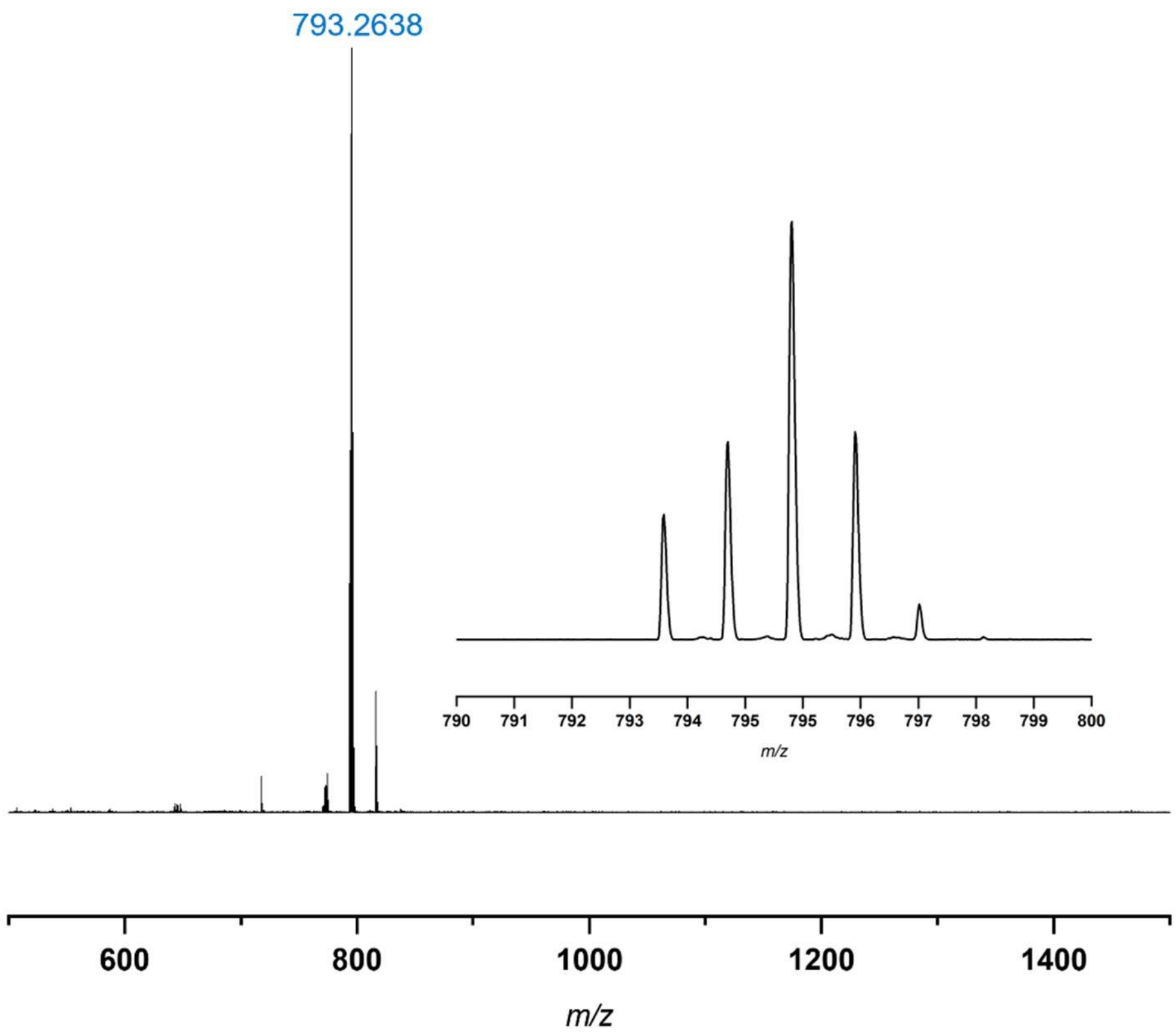

Figure S49. MALDI-TOF spectrum of compound **7a**.

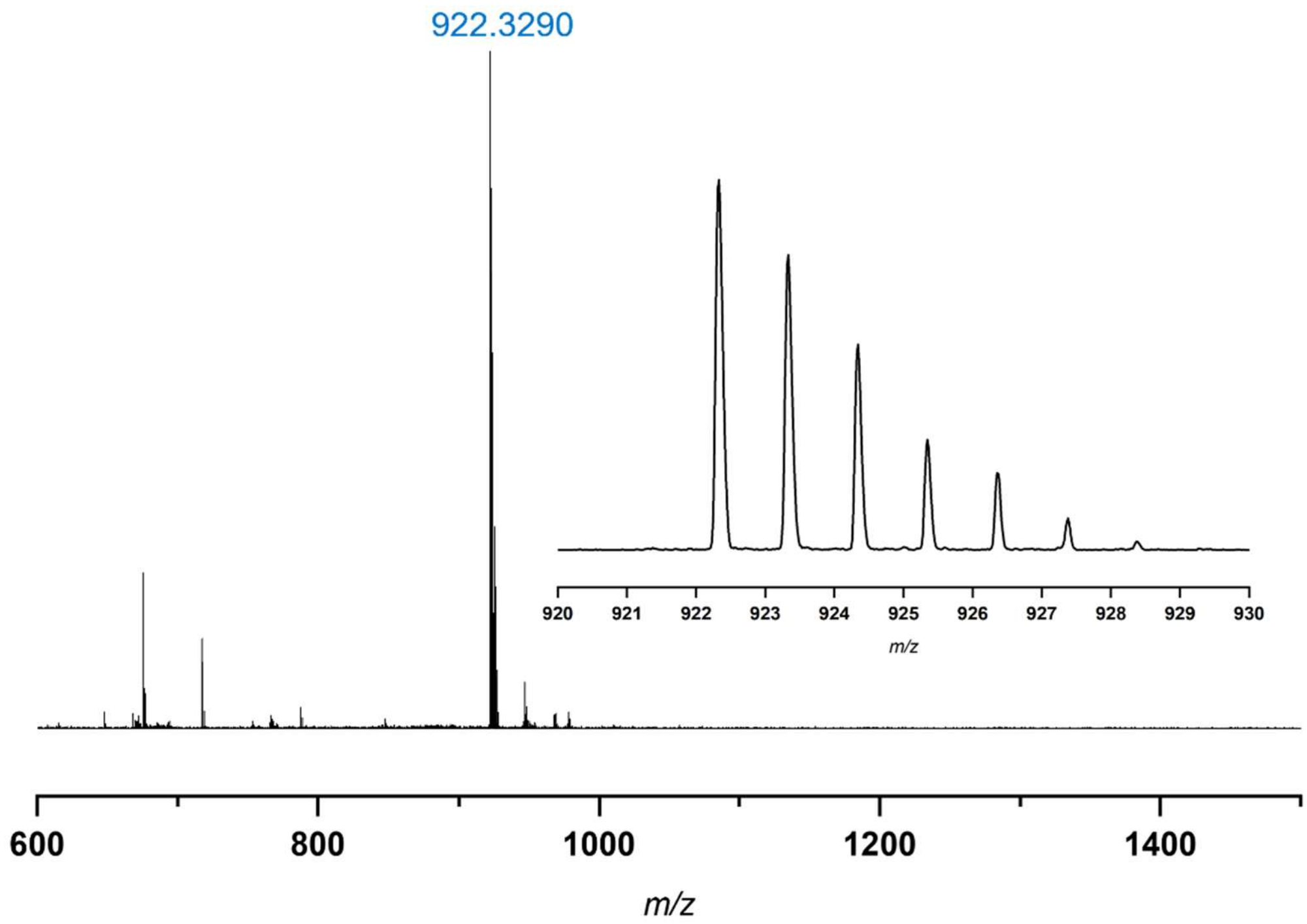

Figure S50. MALDI-TOF spectrum of compound **7b**.

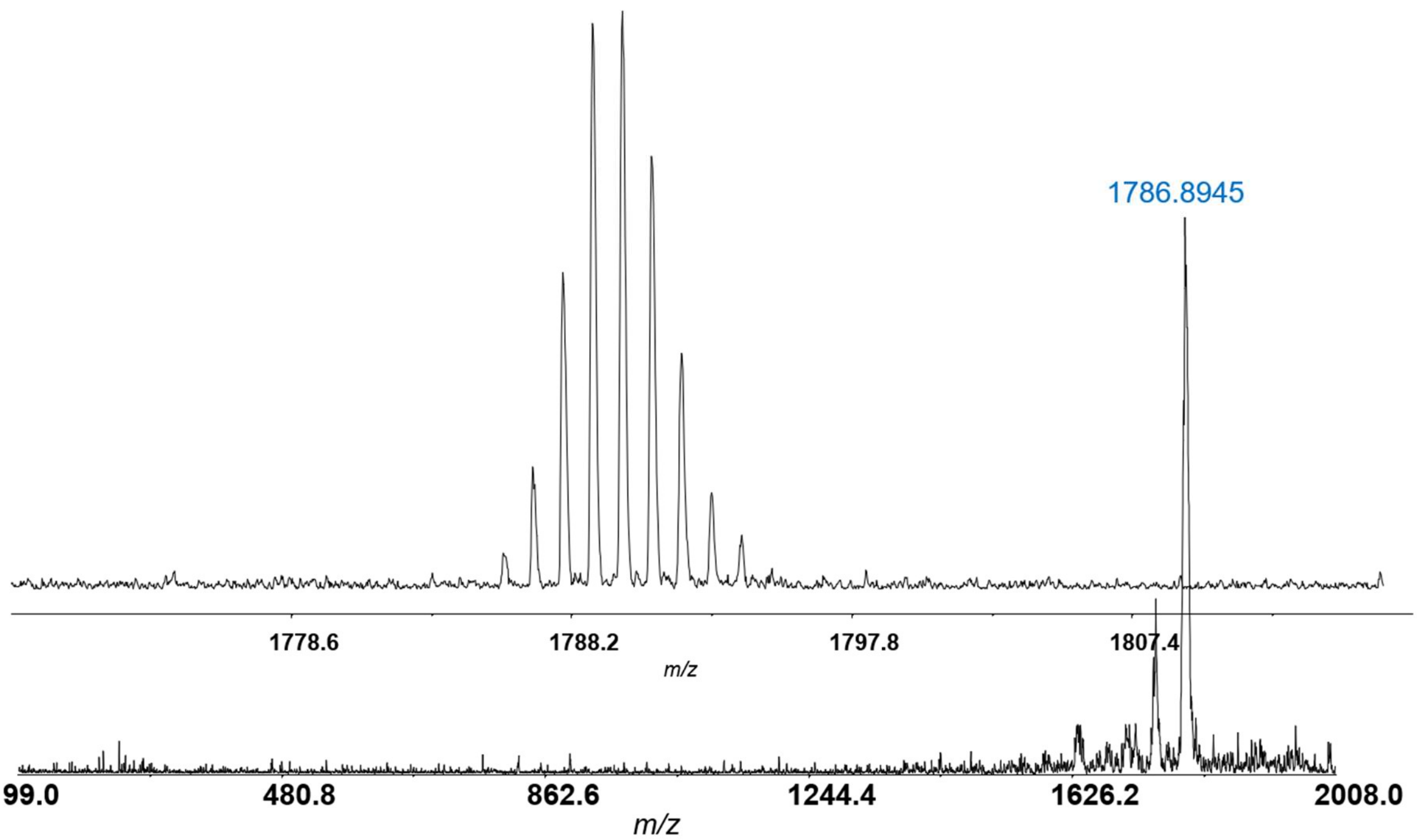

Figure S51. MALDI-TOF spectrum of compound **1**.

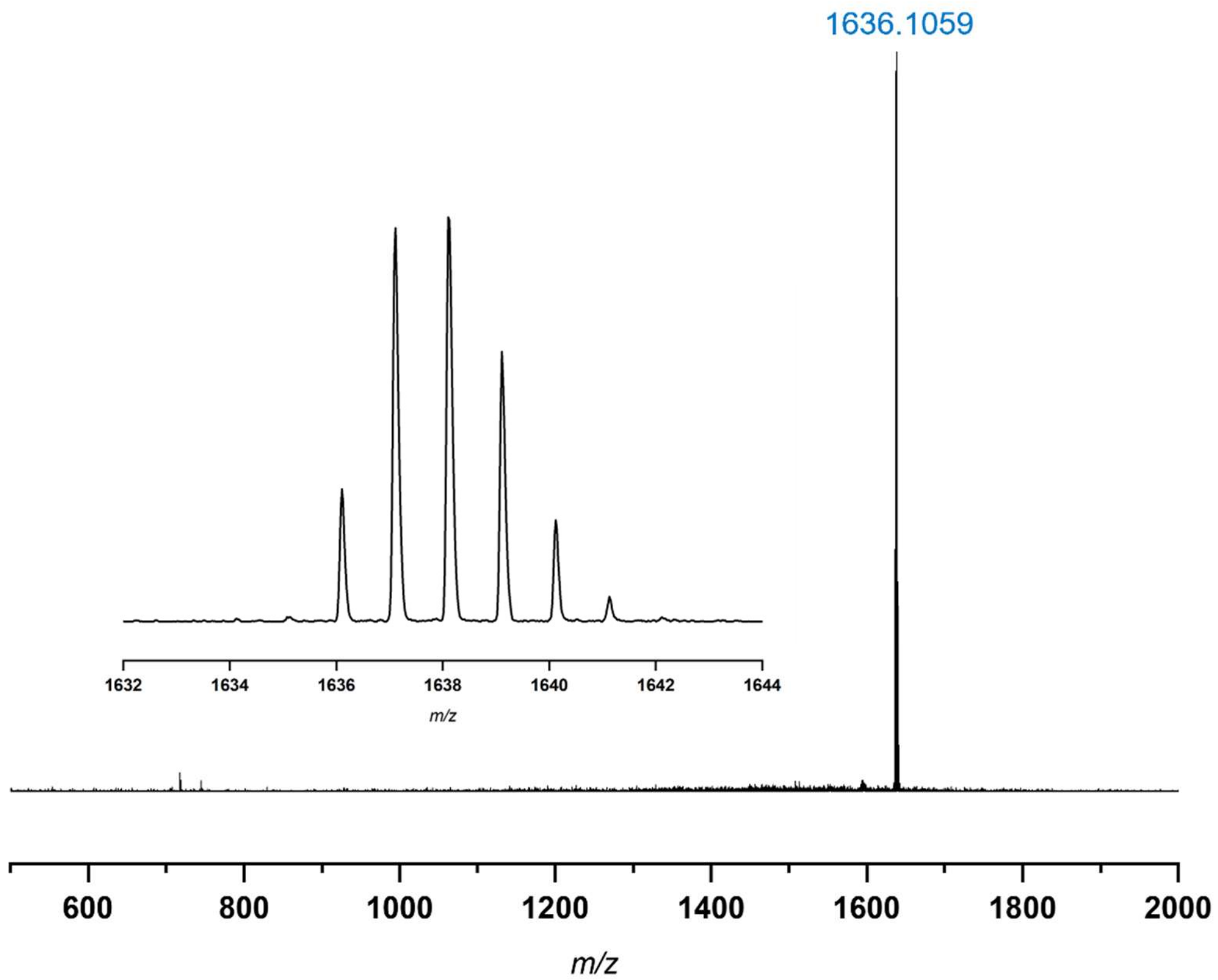

Figure S52. MALDI-TOF spectrum of compound **2**.

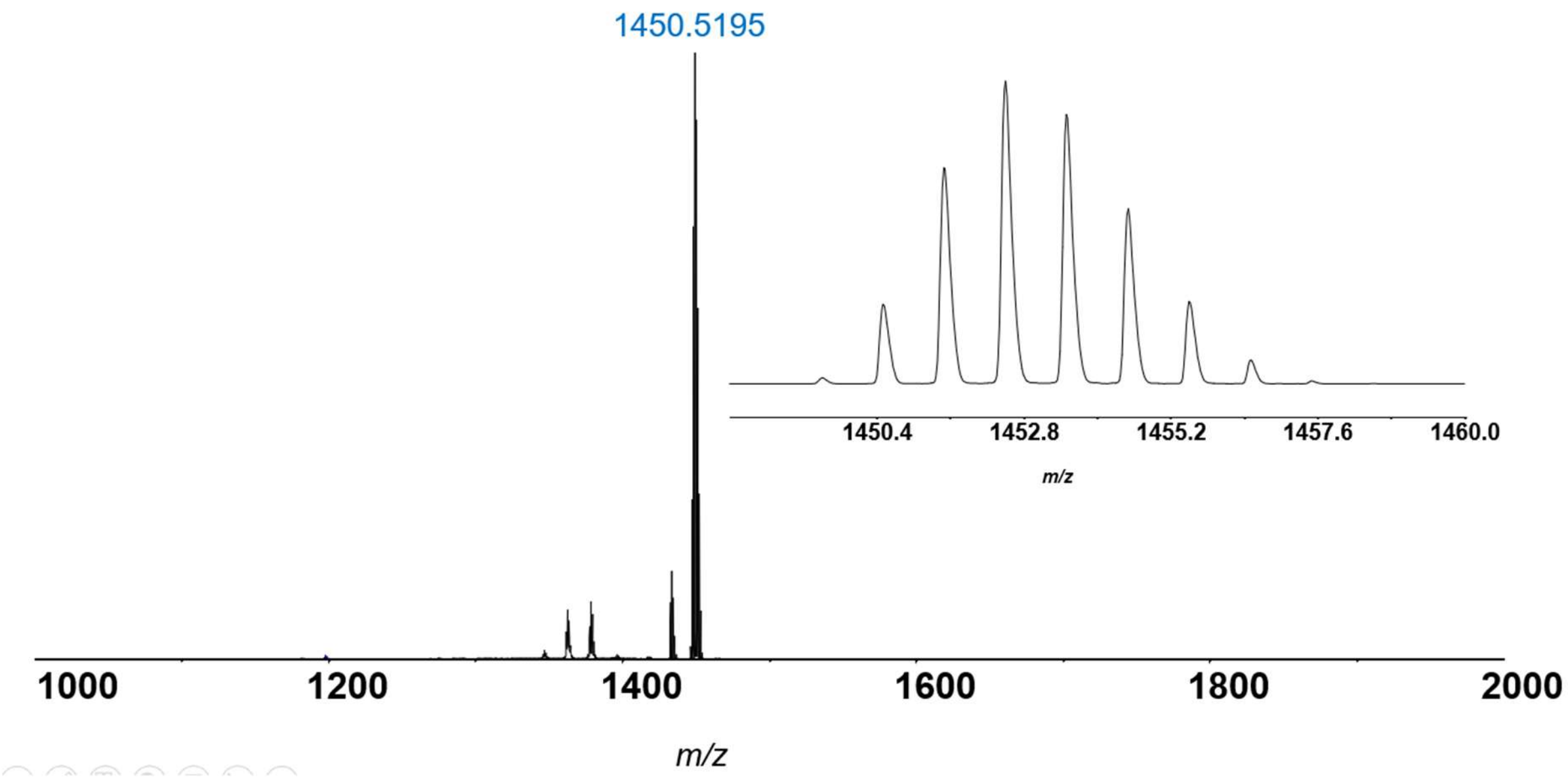

Figure S53. MALDI-TOF spectrum of compound **3**.

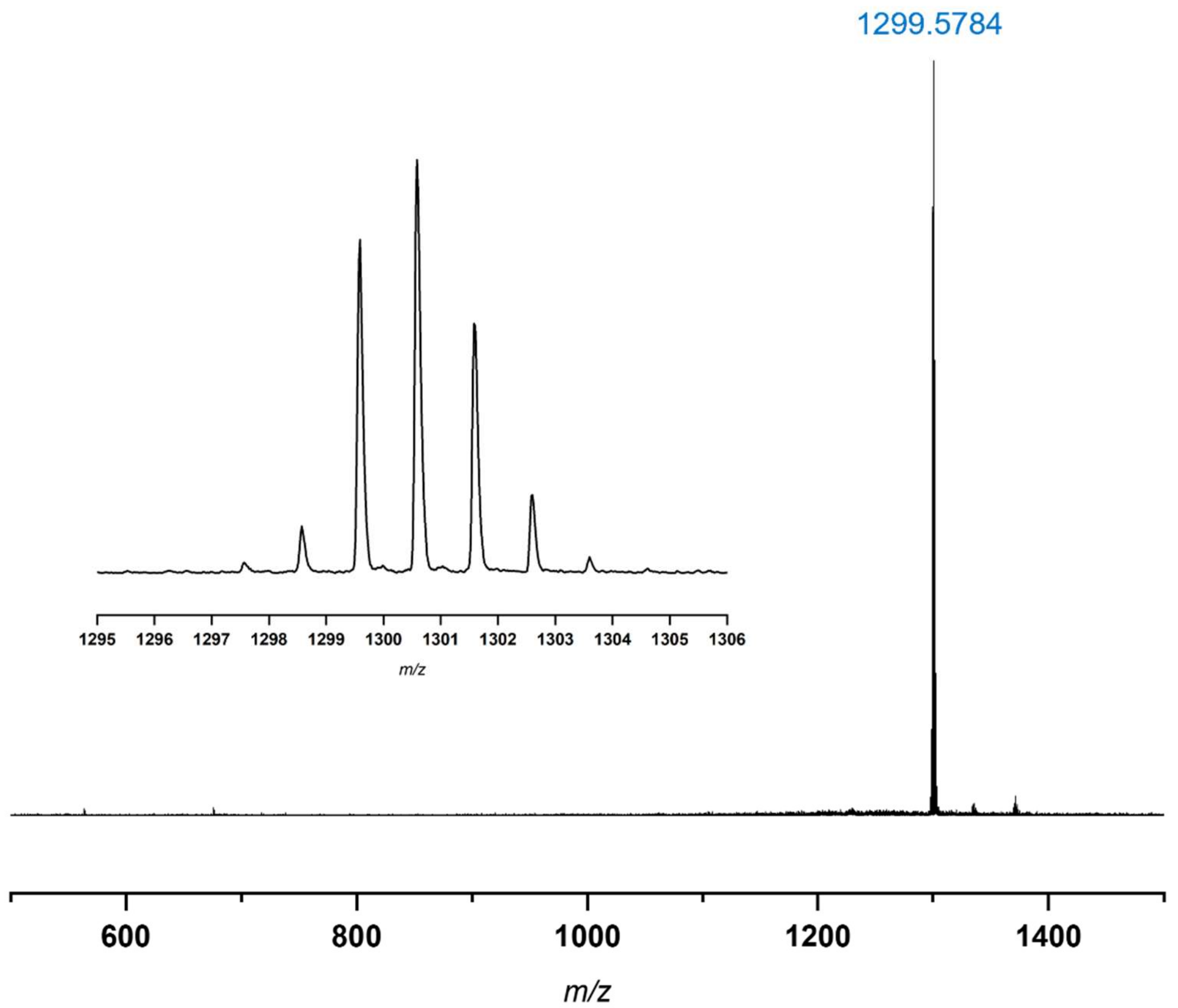

Figure S54. MALDI-TOF spectrum of compound **4**.

5. UV-visible and fluorescence spectroscopy

5.1 Solution-state fluorescence studies and lifetime measurements

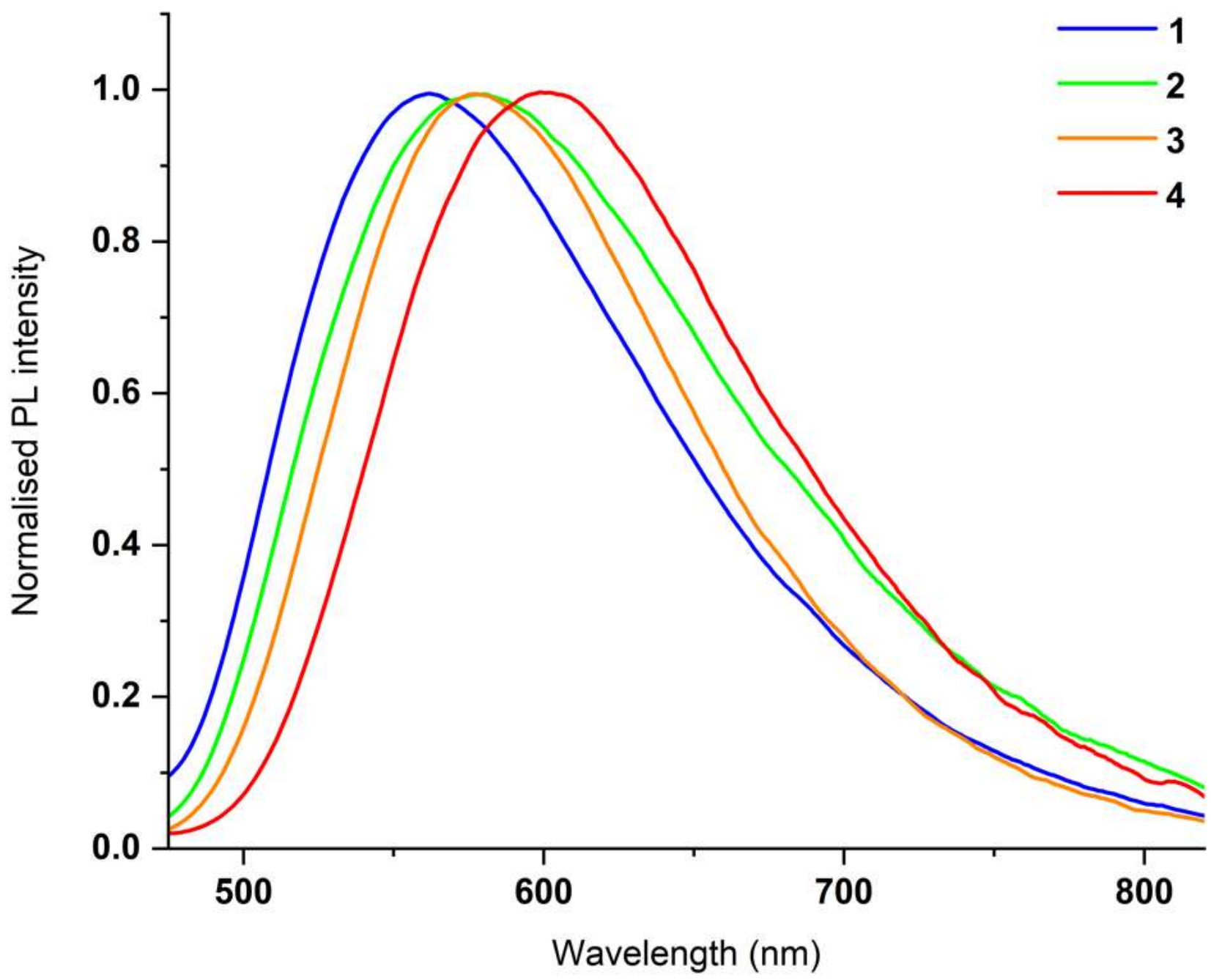

Figure S55. Normalised PL spectra of compounds **1-4** in DCM at 298 K ($c = 1 \times 10^{-5}$ M).

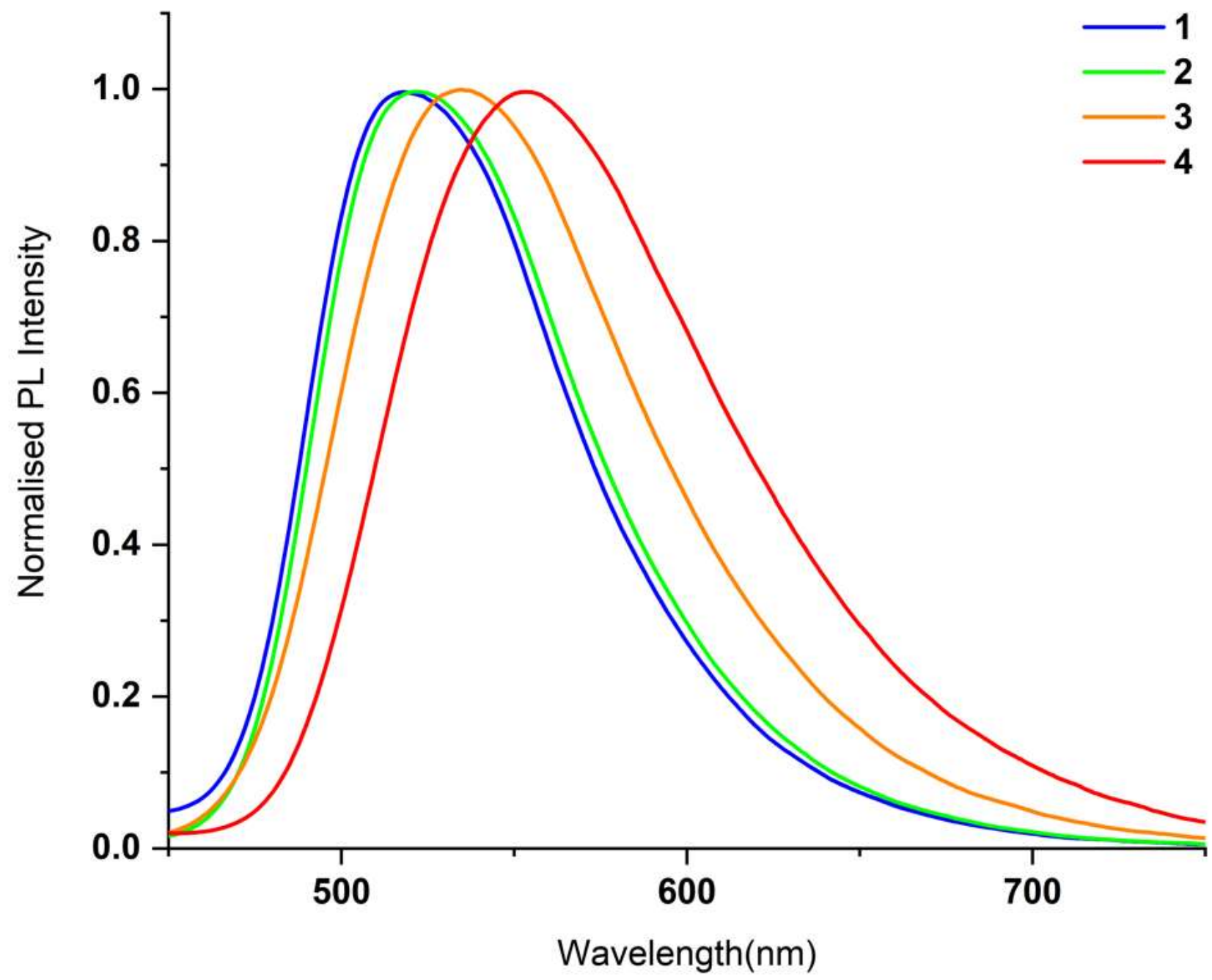

Figure S56. Normalised PL spectra of compounds **1-4** in PhMe at 298 K ($c = 1 \times 10^{-5}$ M).

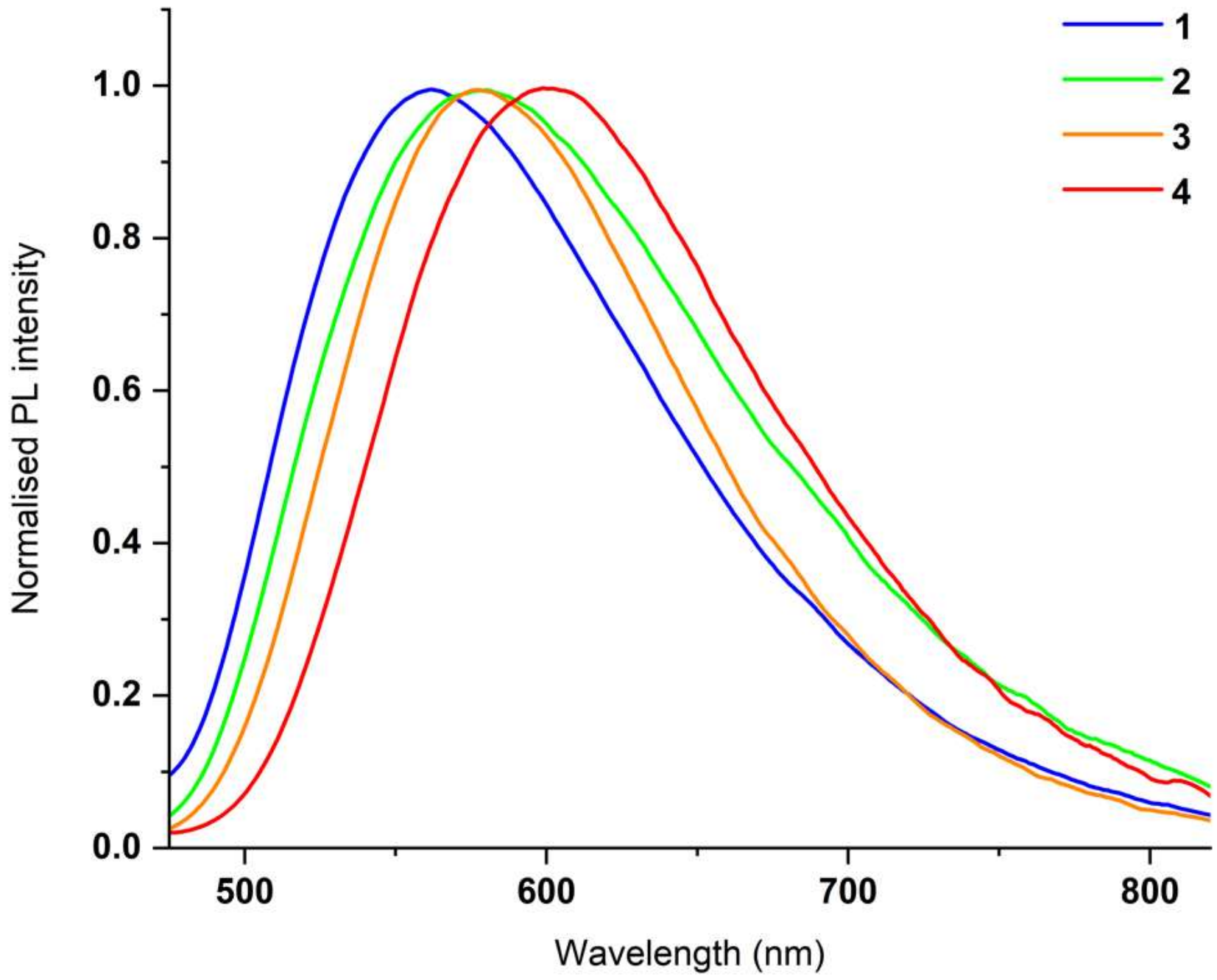

Figure S57. Normalised PL spectra of compounds **1-4** in EtOAc at 298 K ($c = 1 \times 10^{-5}$ M).

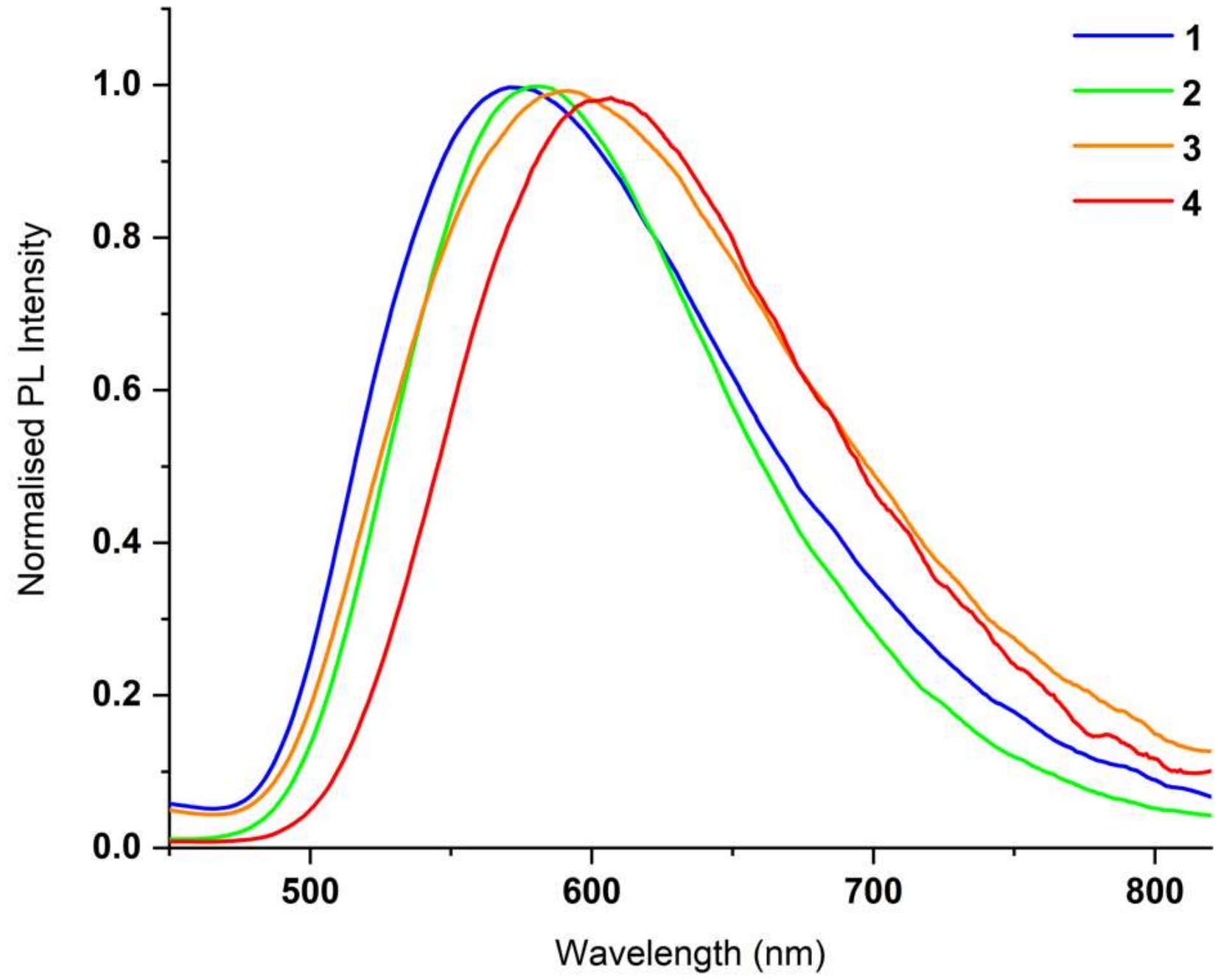

Figure S58. Normalised PL spectra of compounds **1-4** in THF at 298 K ($c = 1 \times 10^{-5}$ M).

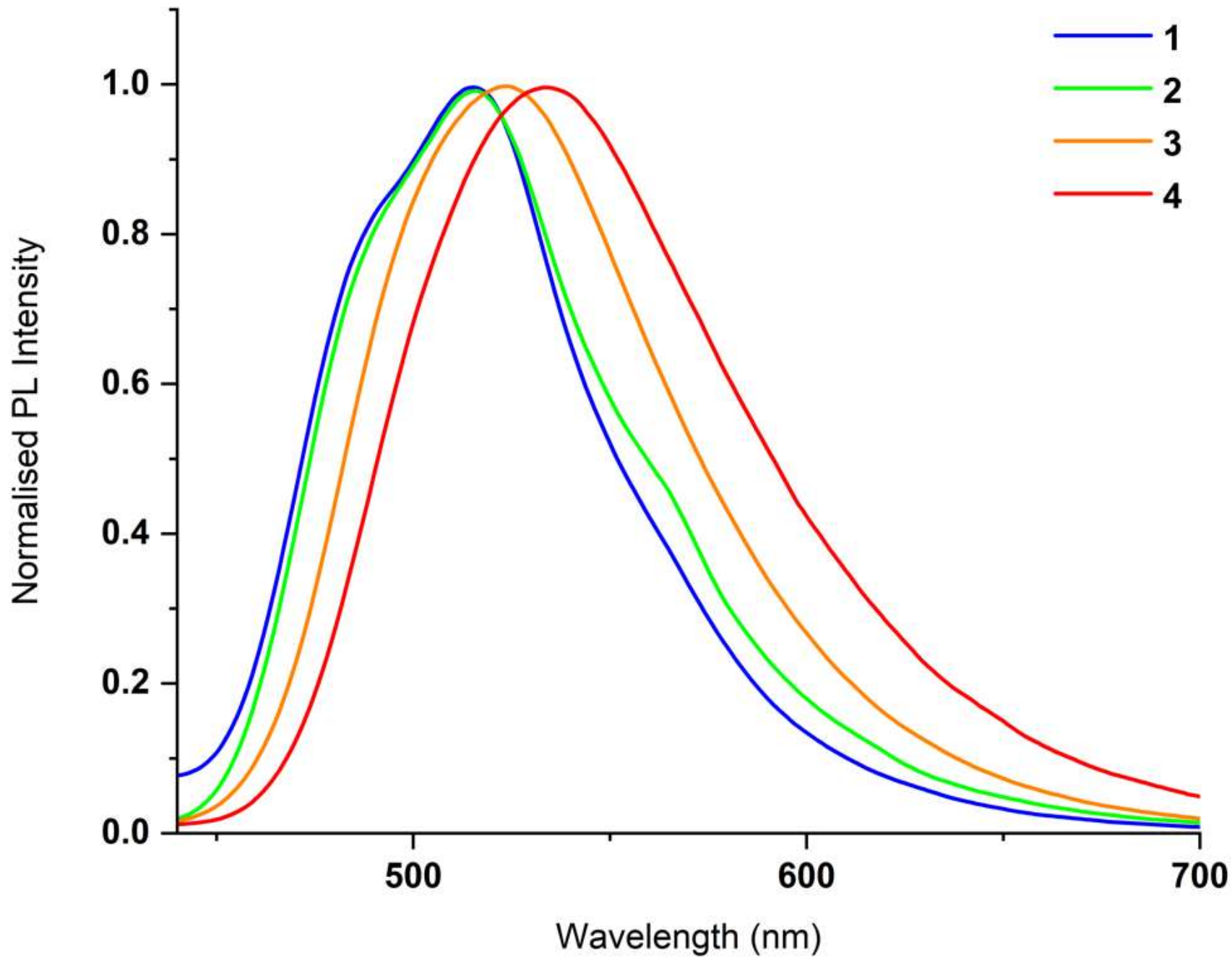

Figure S59. Normalised PL spectra of compounds **1**–**4** in Hexane at 298 K ($c = 1 \times 10^{-5}$ M).

Table S1. Emission of compounds **1**–**4** in different solvents

**Solvent**	**Dielectric constant**	**Emission λ_{max} (nm) of compounds**			
		**1**	**2**	**3**	**4**
Hexane	1.88	514	515	522	535
PhMe	2.38	519	524	536	555
THF	7.58	573	579	590	602
EtOAc	6.02	561	581	580	601
DCM	8.93	564	574	607	622
$CHCl_3$	4.71	564	572	581	598

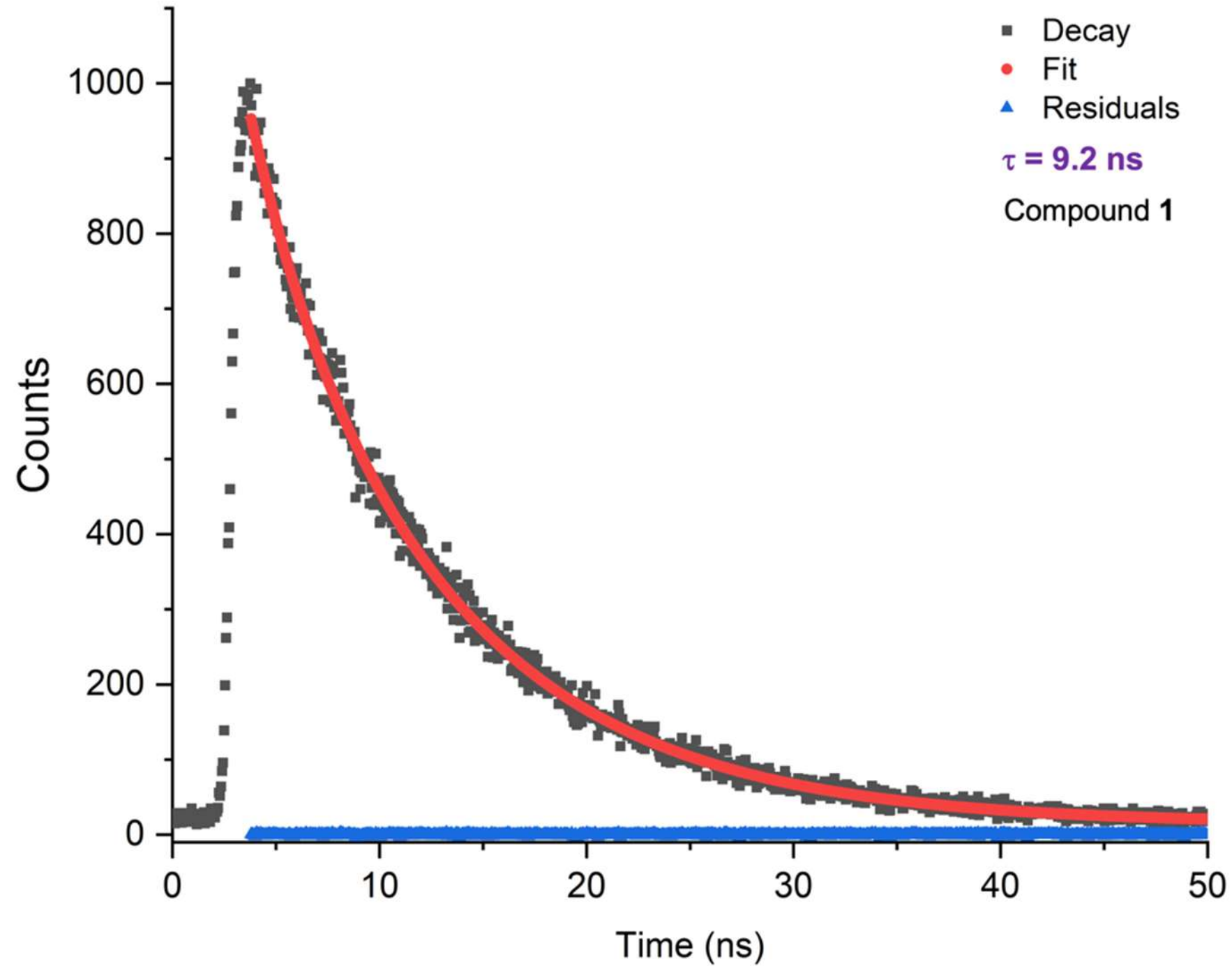

Figure S60. Fluorescence lifetime of azaacene **1** in $CHCl_3$ at 298 K ($c = 1 \times 10^{-5}$ M).

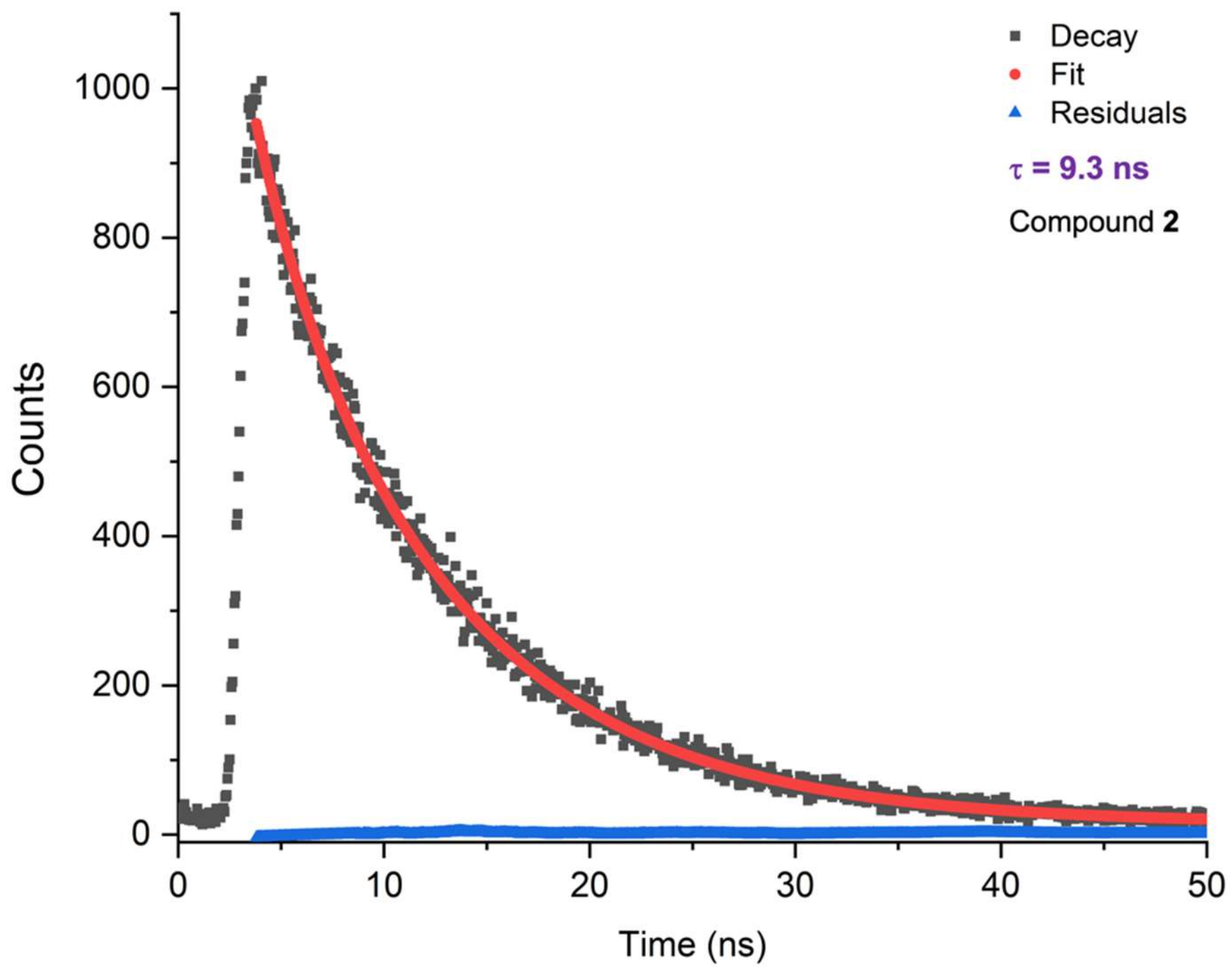

Figure S61. Fluorescence lifetime of azaacene **2** in $CHCl_3$ at 298 K ($c = 1 \times 10^{-5}$ M).

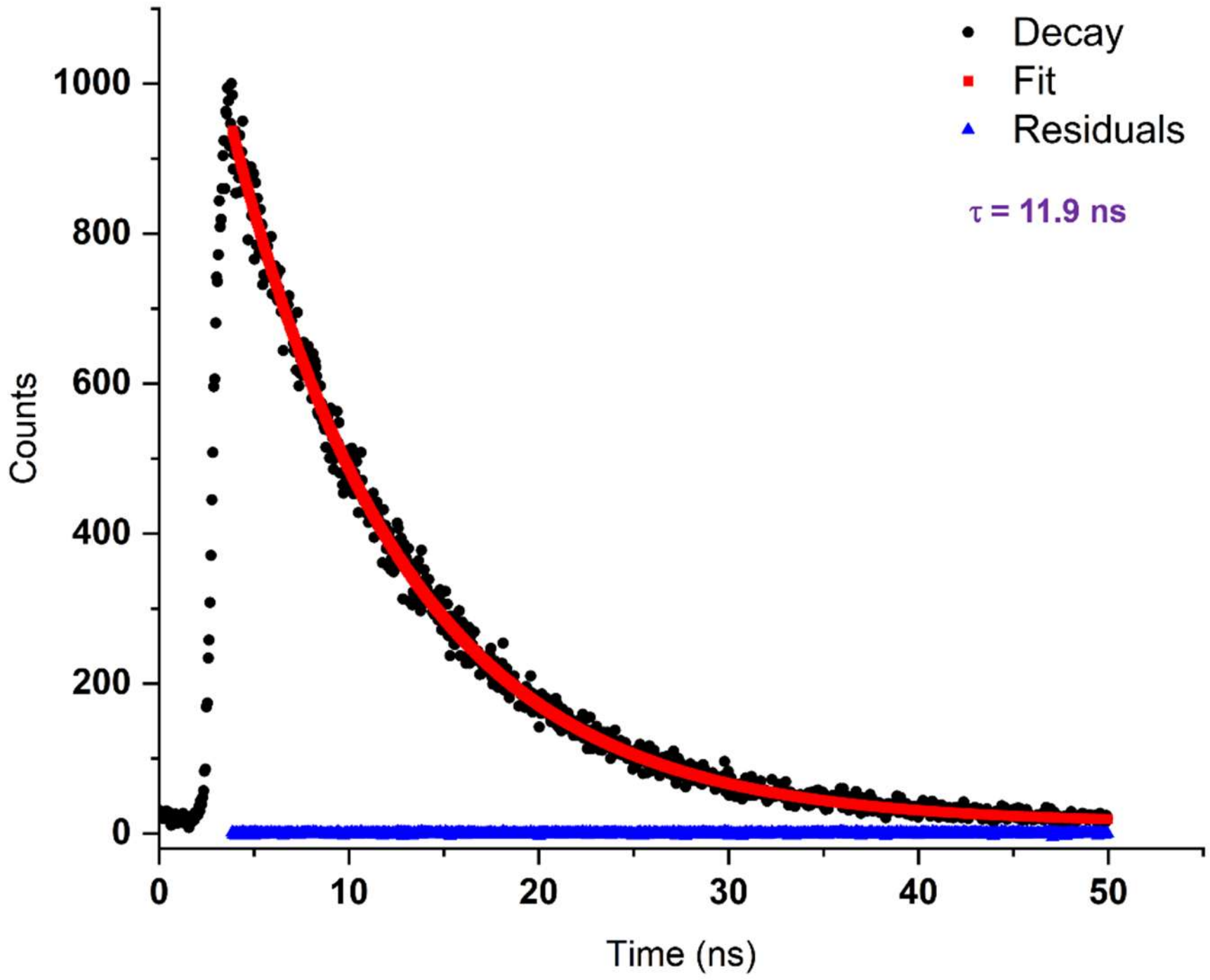

Figure S62. Fluorescence lifetime of azaacene **3** in $CHCl_3$ at 298 K ($c = 1 \times 10^{-5}$ M).

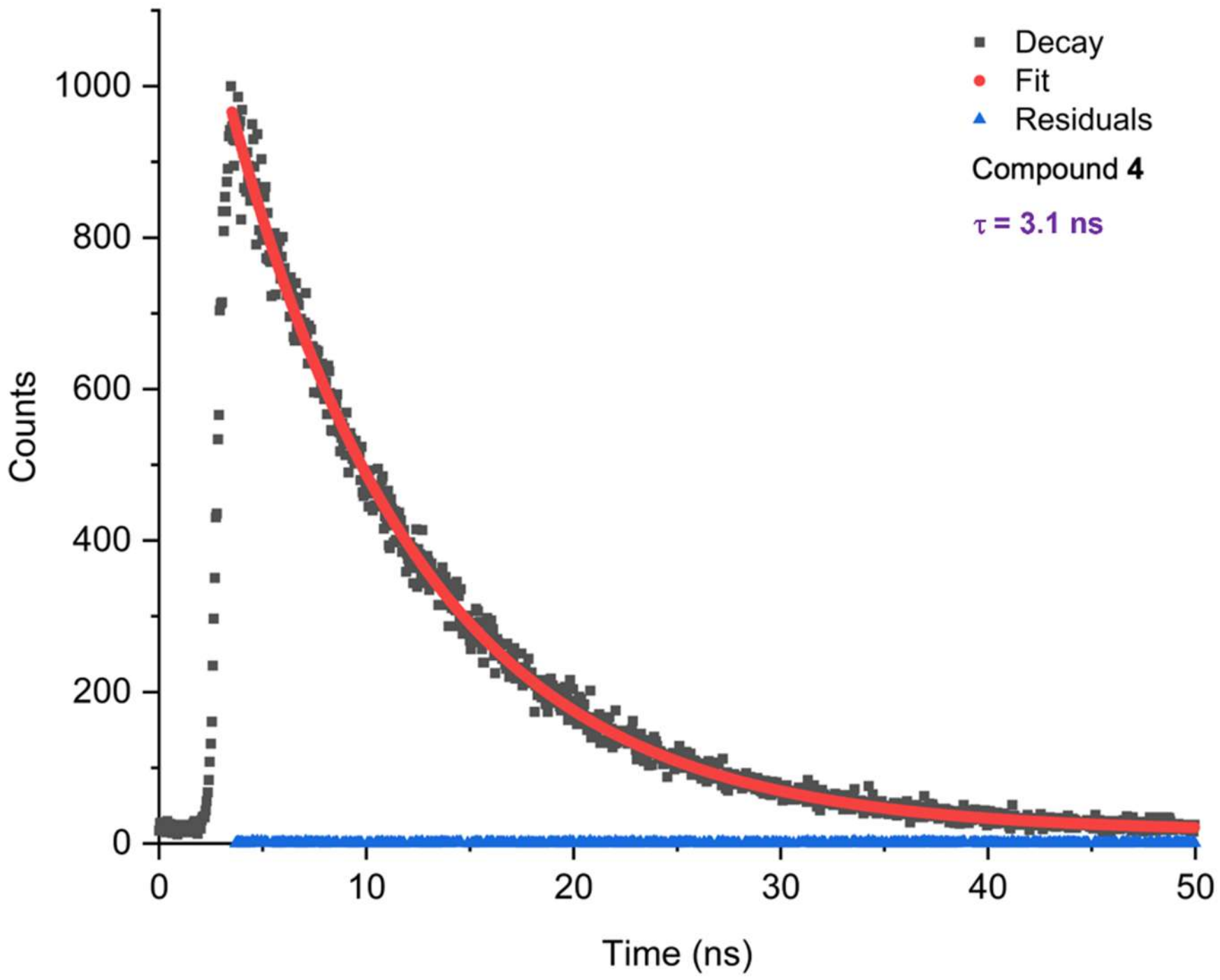

Figure S63. Fluorescence lifetime of azaacene **4** in $CHCl_3$ at 298 K ($c = 1 \times 10^{-5}$ M).

Time-correlated single photon counting (TCSPC) was used to measure fluorescence lifetime using FS5 Spectrofluorometer. The samples are excited at their λ_{max}.

Table S2. Absolute quantum yield of azacenes **1-4** in $CHCl_3$ ($c = 1 \times 10^{-5}$ M). Three different samples of each compound were prepared, and average values PLQYs are reported.

Compound	Quantum Yield Φ (%)
**1**	13.85
	13.17
	13.55
**2**	7.22
	7.26
	7.91
**3**	9.98
	9.86
	9.71
**4**	5.85
	6.01
	5.91

The absolute quantum yields were measured using the integrated sphere method employing Xenon lamp.

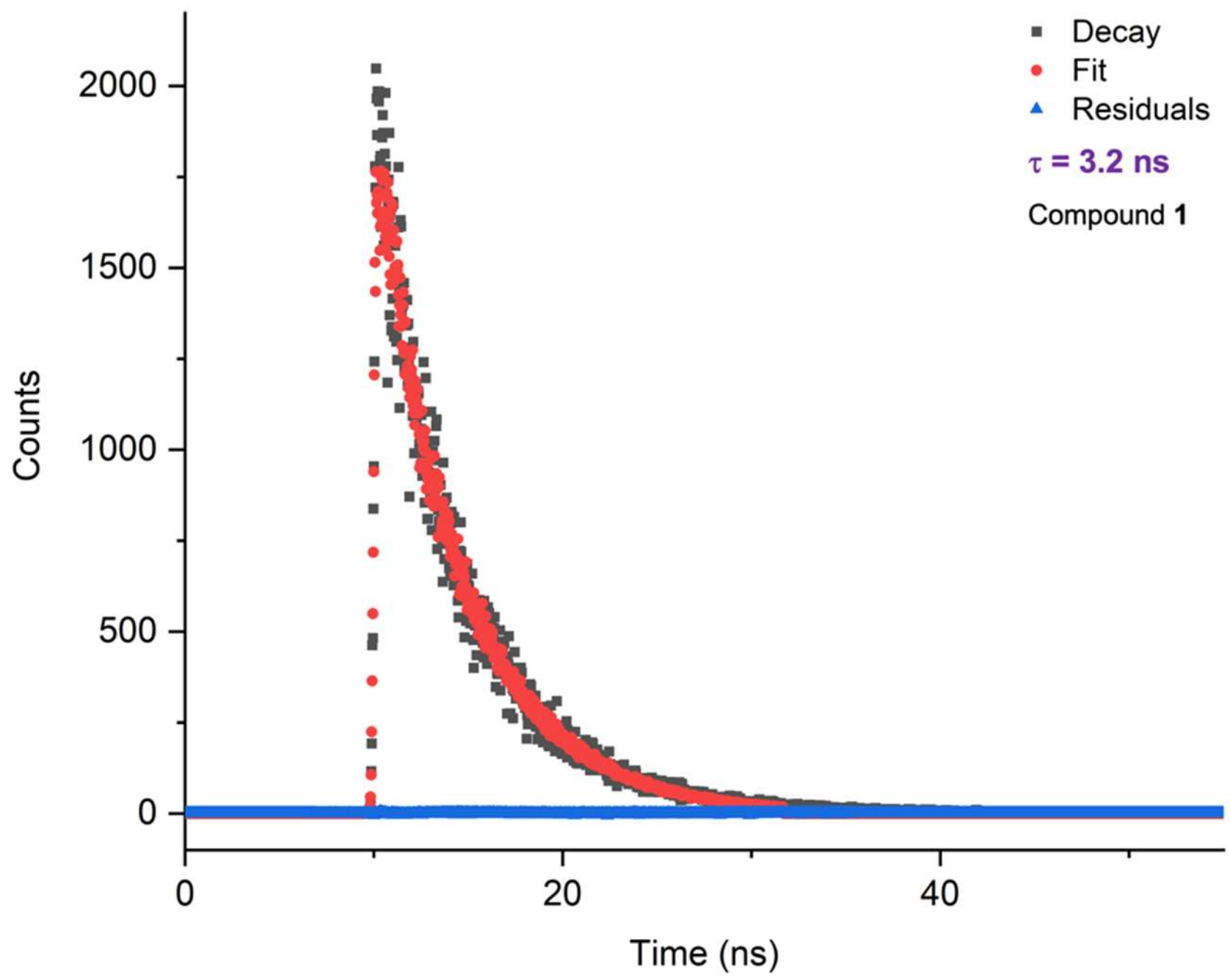

Figure S64. Fluorescence lifetime of azaacene **1** in thin-film at 298 K.

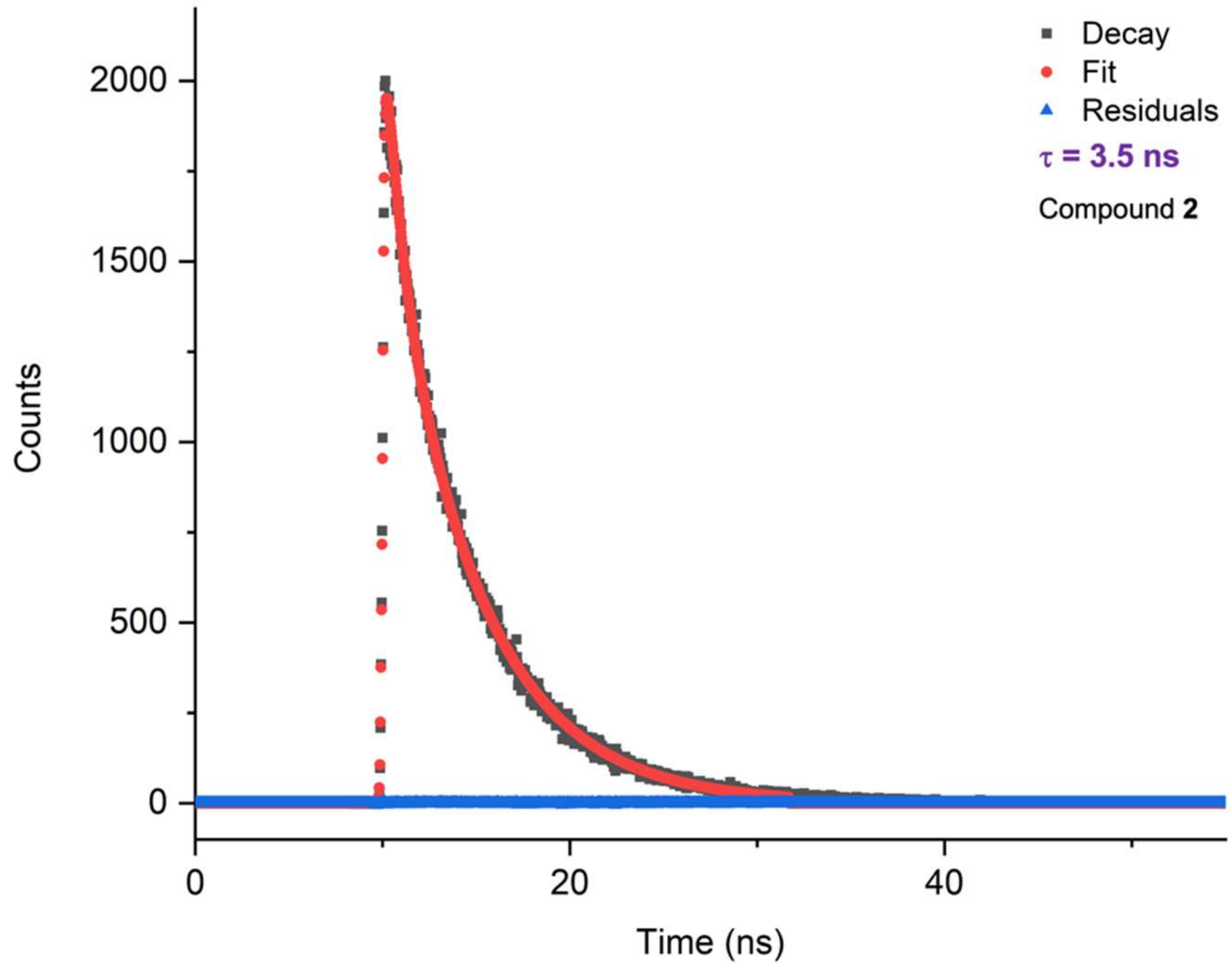

Figure S65. Fluorescence lifetime of azaacene **2** in thin-film at 298 K.

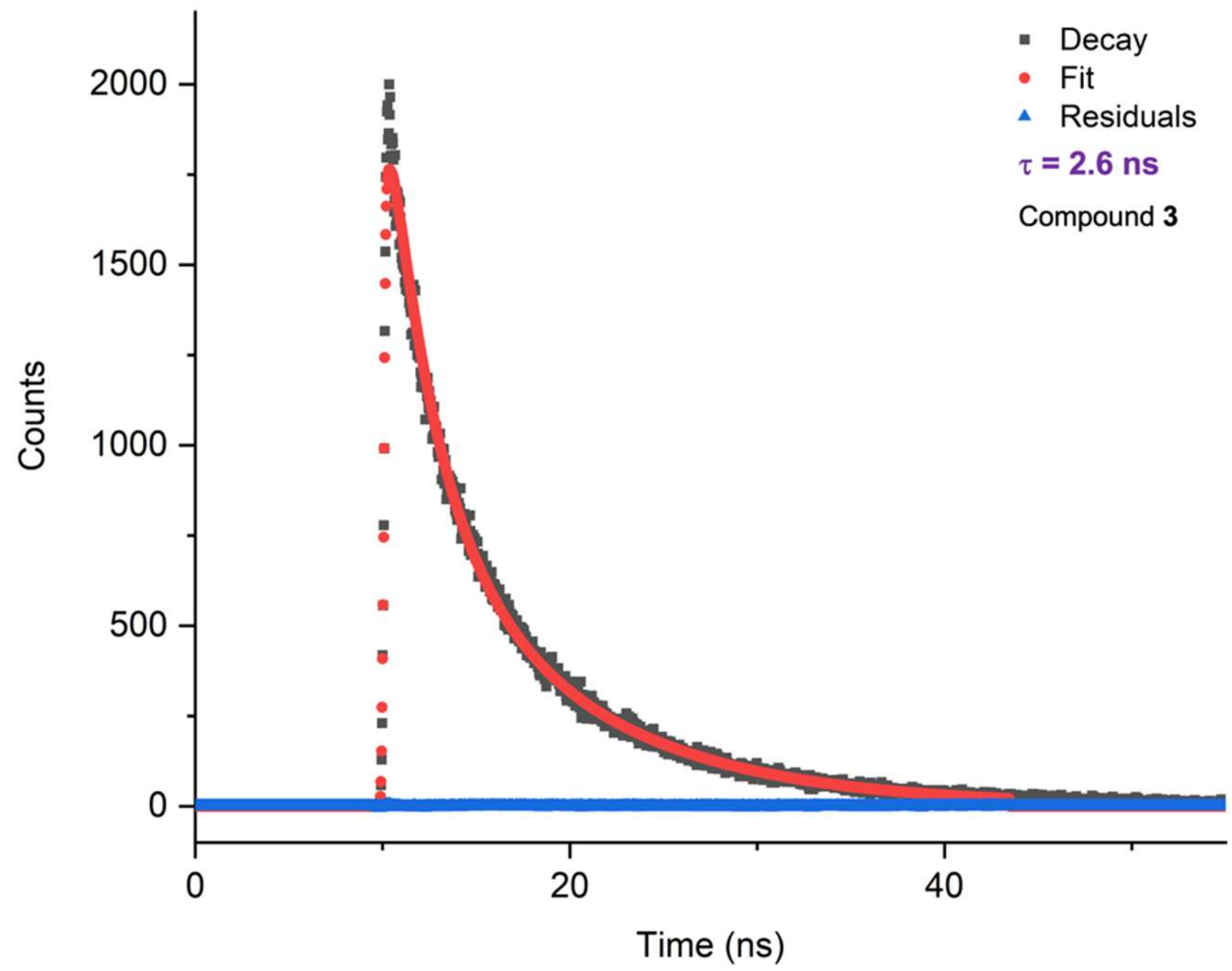

Figure S66. Fluorescence lifetime of azaacene **3** in thin-film at 298 K.

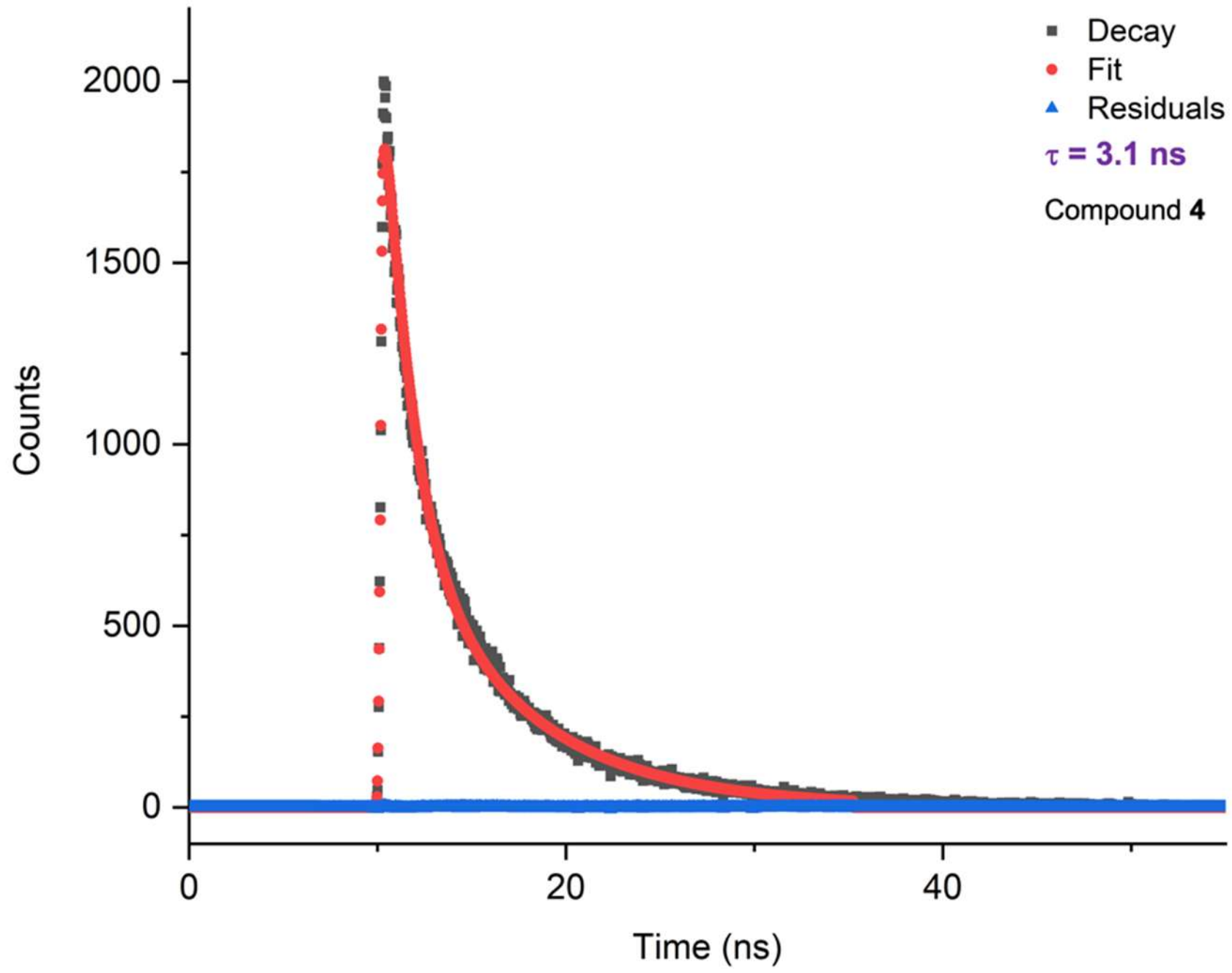

Figure S67. Fluorescence lifetime of azaacene **4** in thin-film at 298 K.

Table S3. Absolute quantum yield of compounds **1-4** in drop-cast thin-films. Three different samples of each compound were prepared, and average values of PLQYs are reported.

**Compound**	**Quantum Yield Φ (%)**
**1**	41.06
	45.54
	46.61
**2**	81.25
	80.91
	82.45
**3**	41.63
	44.20
	43.36
**4**	26.22
	29.39
	28.99

The absolute quantum yields solid states were measured using the integrated sphere method employing Xenon lamp.

6. Cyclic Voltammetry

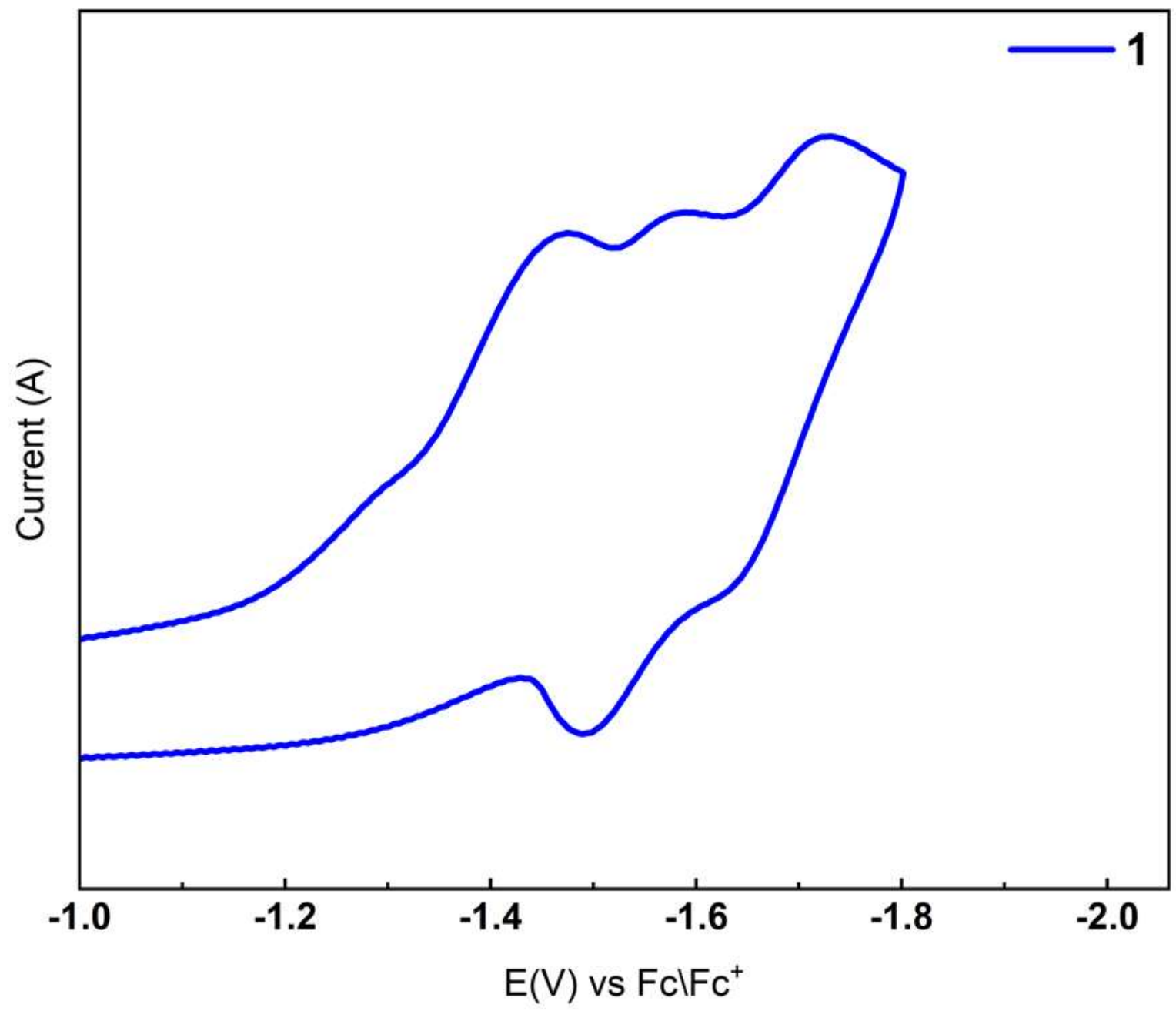

Figure S68. Normalised cyclic voltammograms of **1** in *n*-Bu_4NPF_6 (0.1 M) in DCM.

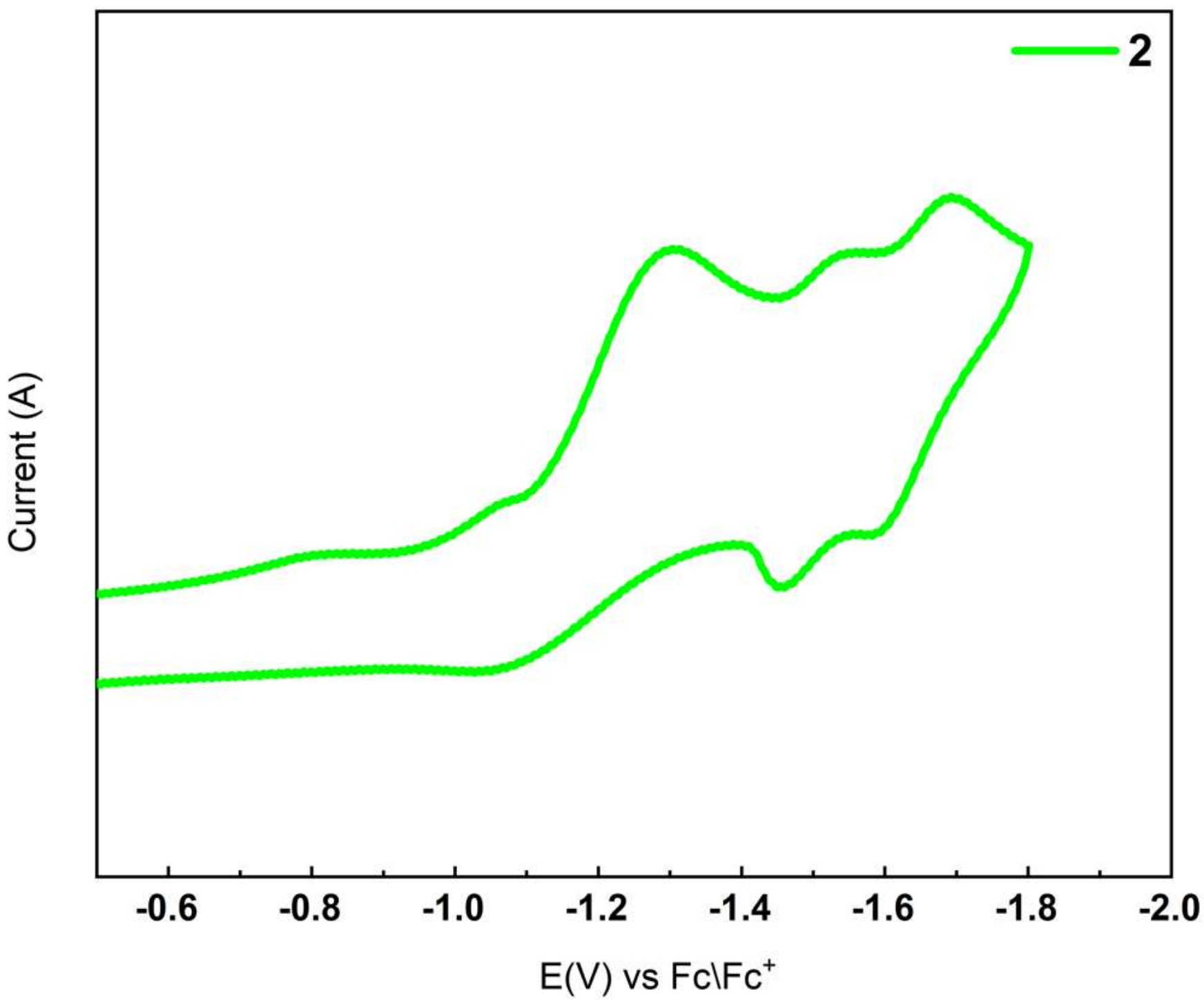

Figure S69. Normalised cyclic voltammograms of **2** in *n*-Bu_4NPF_6 (0.1 M) in DCM.

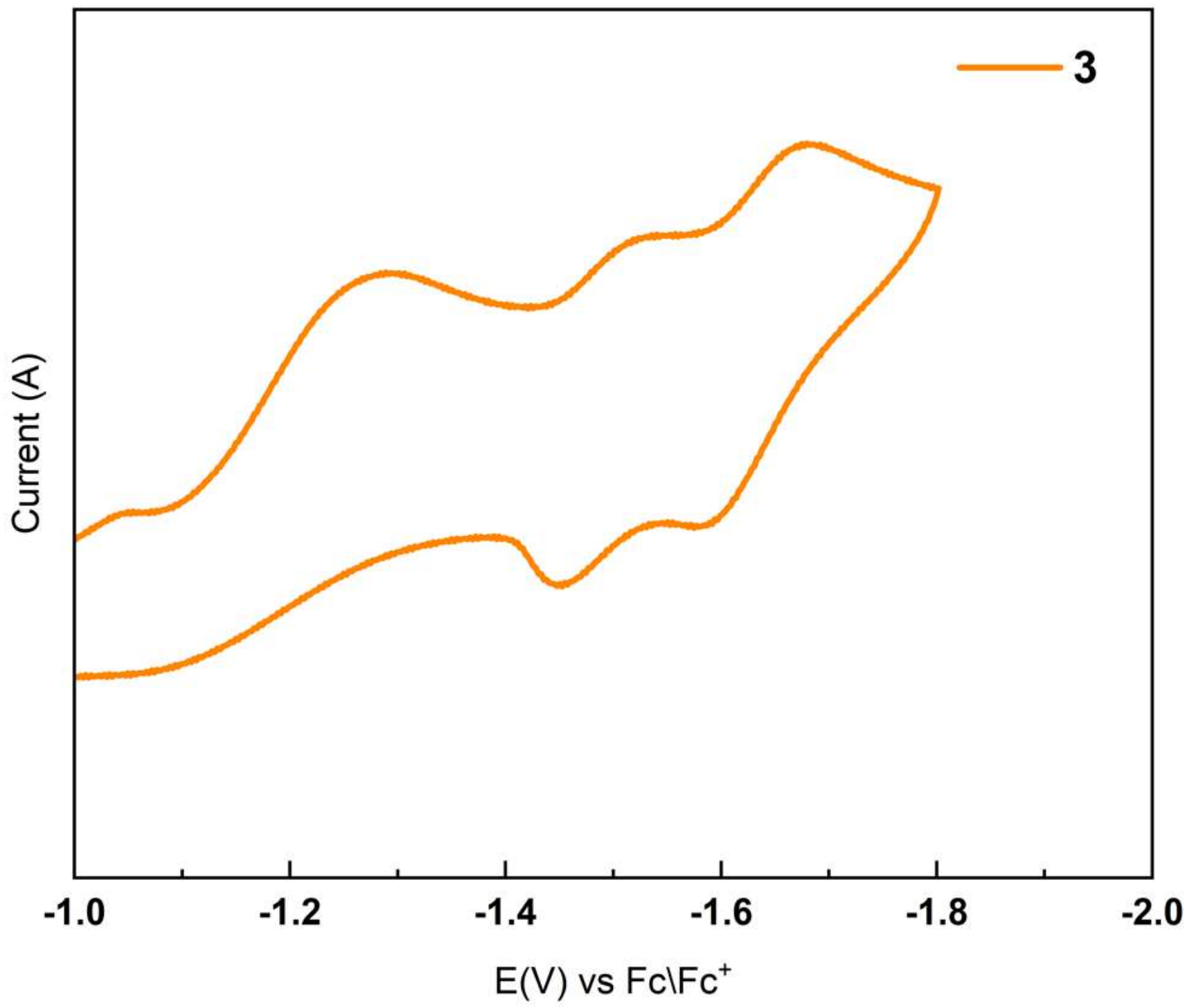

Figure S70. Normalised cyclic voltammograms of **3** in *n*-Bu_4NPF_6 (0.1 M) in DCM.

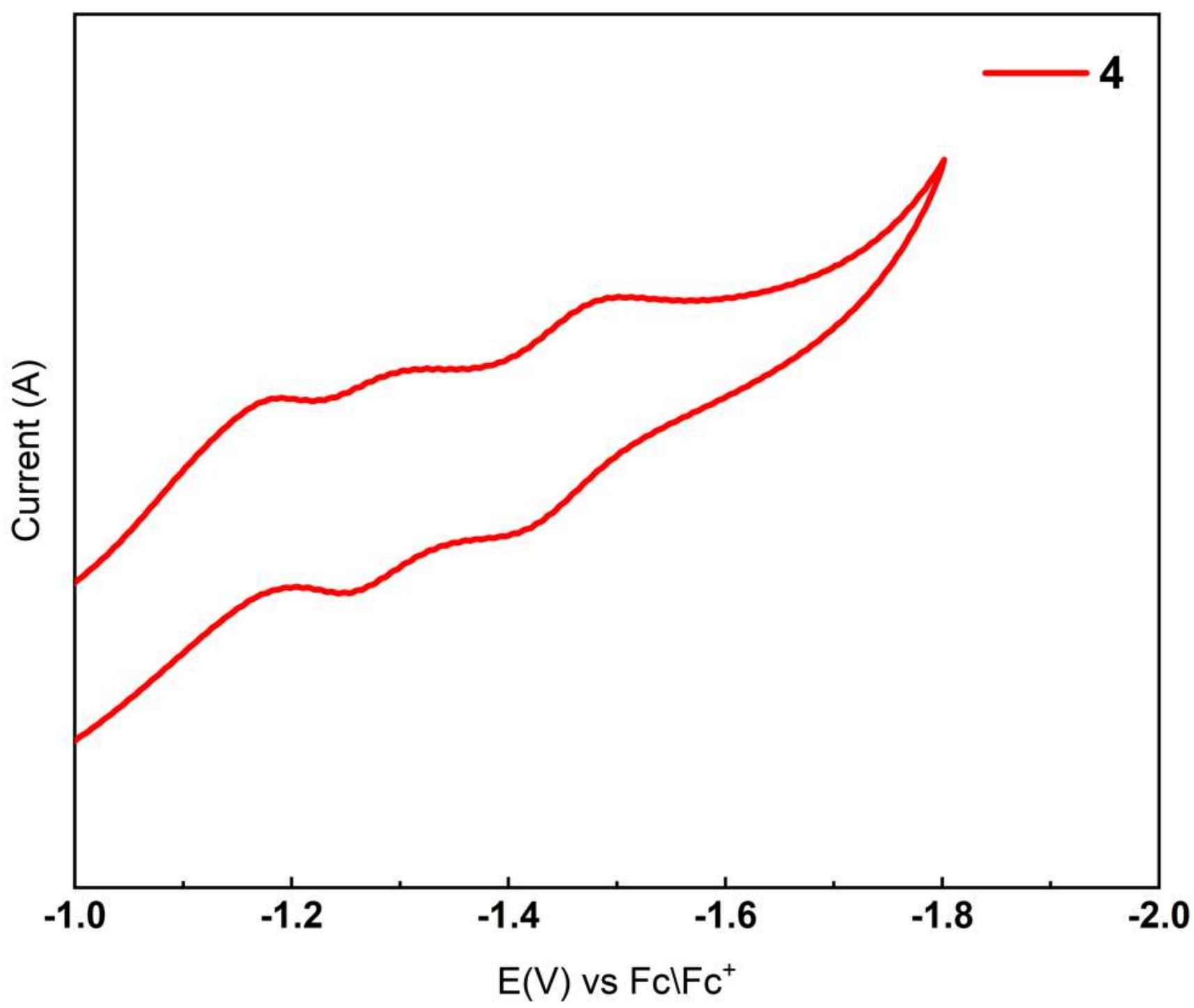

Figure S71. Normalised cyclic voltammograms of **4** in *n*-Bu_4NPF_6 (0.1 M) in DCM.

7. Single-crystal X-ray diffraction data

SC-XRD: The single crystal X-ray diffraction measurements were performed to determine the crystal structure of compounds **6a**, **2** and **3** at 100 K using APEX3 (Bruker, 2016; Bruker D8 VENTURE Kappa Duo PHOTON II CPAD) diffractometer having graphite monochromatized (MoKα (0.71073)). The X-ray generator was operated at 50 kV and 30 mA. A preliminary set of unit cell parameters and an orientation matrix were calculated from 36 frames, and the cell refinement was performed by SAINT-Plus (Bruker, 2016). An optimised strategy used for data collection consisted of different sets of φ and ω scans with 0.5° steps φ/ω. The data were collected with a time frame of 10 seconds by setting the sample to detector distance fixed at 40 cm. All the data points were corrected for Lorentzian, polarisation, and absorption effects using SAINT-Plus and SADABS programs (Bruker, 2016). SHELXS-97 (Sheldrick, 2008) was used for structure solution, and full-matrix least squares refinement on F2.[6,7] The molecular graphics of ORTEP diagrams were performed using Mercury software. The crystal symmetry of the components was cross-checked by running the CIF files through PLATON (Spek, 2020) software, and no additional symmetry was observed. The Encifer software was used to correct the CIF files.

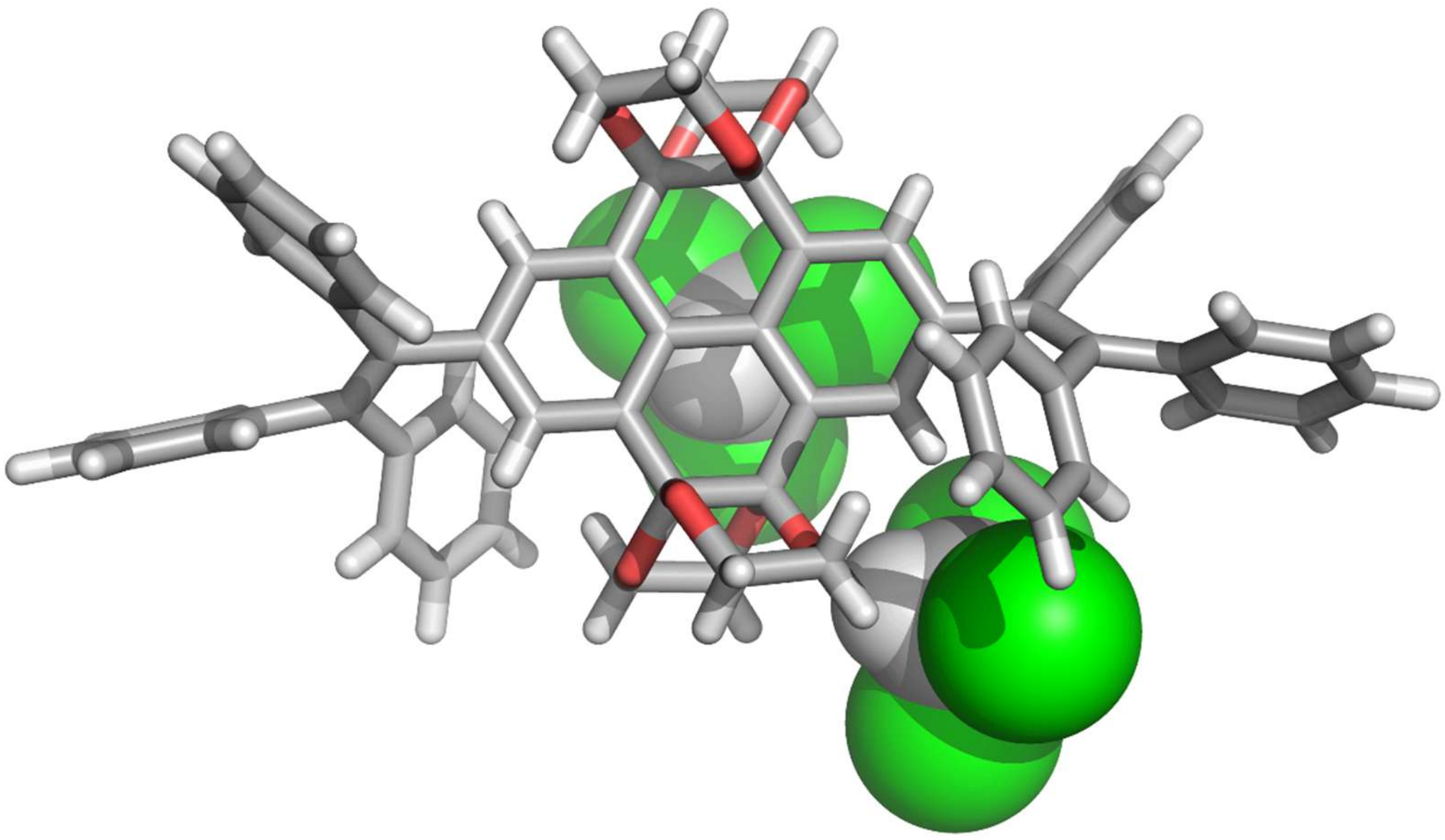

Figure S72. ORTEP diagram of triPE-tetraketal **6a**. Thermal ellipsoids are shown at 50% probability. The single crystals were grown by slow diffusion of ethanol vapour to a solution of **6a** in chloroform at room temperature.

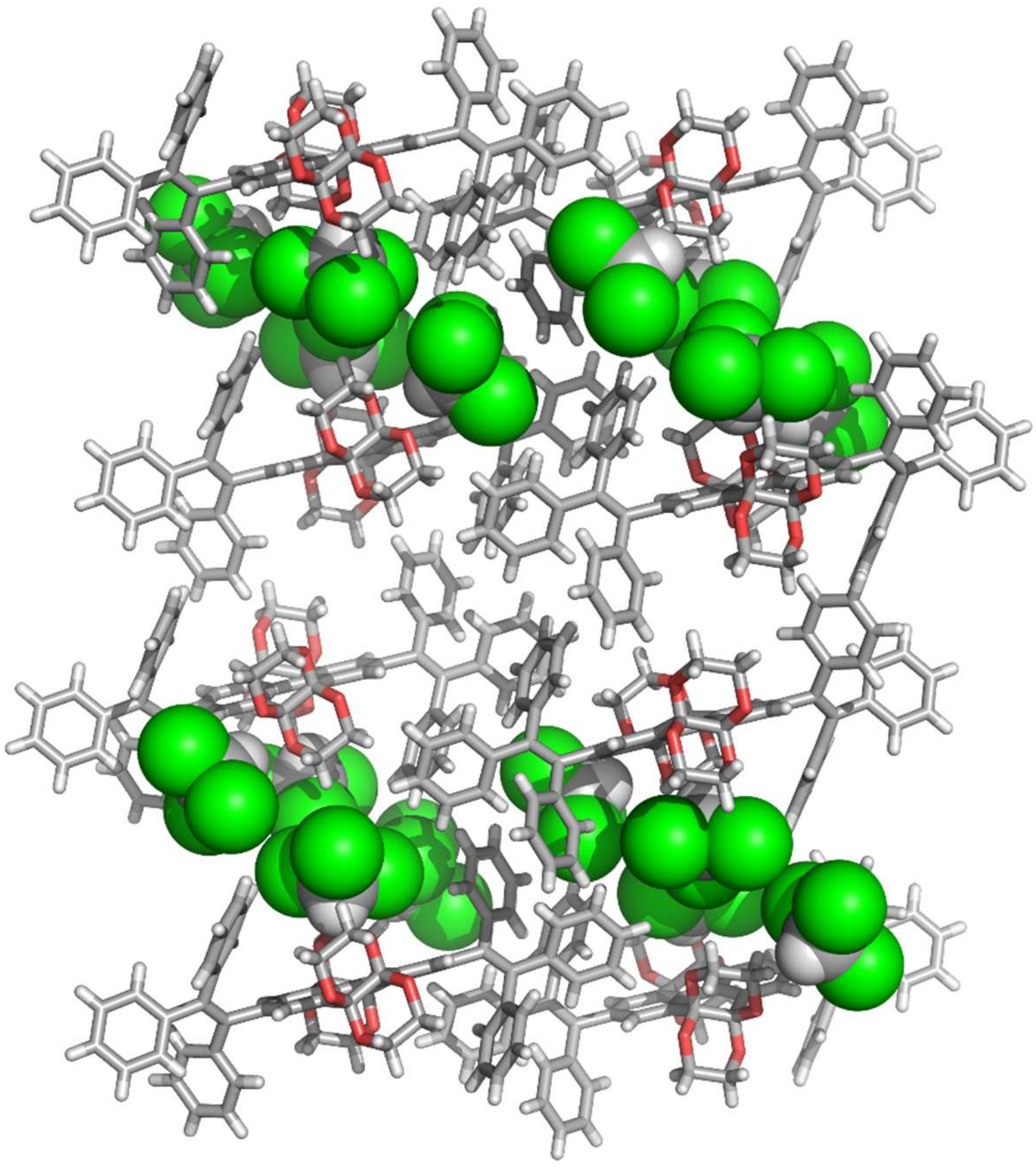

Figure S73. Packings observed in the single crystal X-ray structure of **6a**.

Table S4. Crystallographic data of compound **6a**:

Colour, crystal shape & size in mm	Colourless, plate, 0.23 × 0.10 × 0.08	
CCDC number	2496421	
Chemical formula & Formula weight	$C_{68}H_{42}O_4$, 923.06	
Temperature	100 (2)	
Wavelength	0.71073	
Radiation source	MoKα	
Crystal system	Monoclinic	
Space group	$C_{12}C_{1}$	
Z	8	
Unit cell dimensions	a = 39.754(6) Å	α = 90 °
	b = 11.251 (18) Å	β = 92.317 (5) °
	c = 27.834 (4) Å	γ = 90 °
Volume and density	12439(3) $Å^3$, 1.266	
Absorption coefficient	0.329 mm^{-1}	

D*calc* (mgm^{-3})	1.266
μ (mm^{-1})	0.329
Reflections collected	13503
Theta range for collection	2.40–27.22
**Data collection -** Diffractometer	Bruker D8 VENTURE Kappa Duo PHOTON II CPAD
Absorption correction	SADABS-2014/5
T_{min}, T_{max}	0.6879, 0.7456
No. of measured, independent & observed [I > 2σ(I)] reflections	299447, 13503, 11164
Theta range (°)	2.40–27.22
Rint	0.115
**Refinement**	
R[F2 > 2σ (F^2)], wR(F^2)	0.137, 0.146
GOF on F^2	1.07
No. of independent reflections	13503
No. of parameters	749
F_000	4912
No. of restraints	3
H-atom treatment	Constr

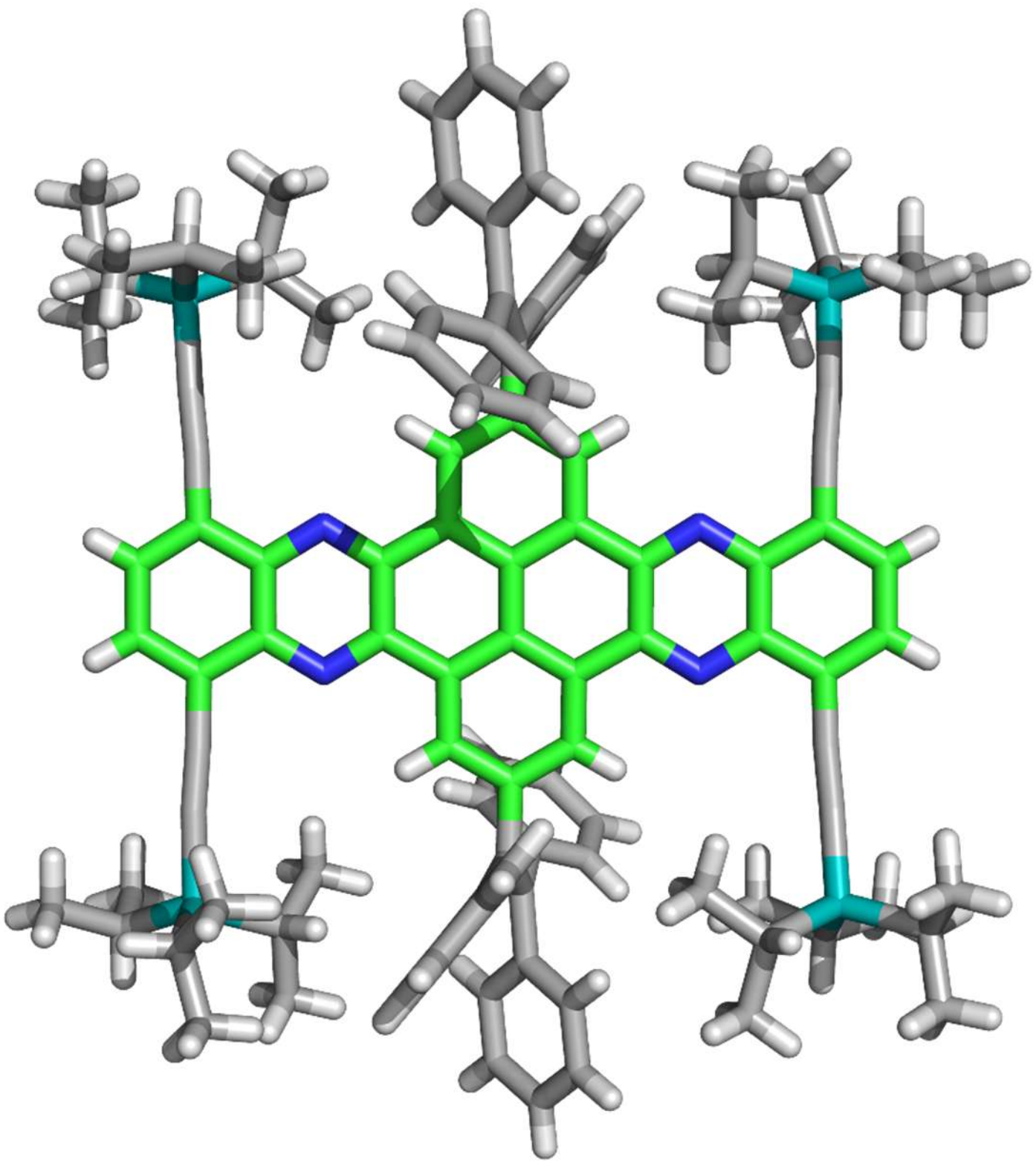

Figure S74. ORTEP diagram of **2**. Thermal ellipsoids are shown at 50% probability. The single crystals of **2** were grown by slow diffusion of ethanol vapours to a solution of **2** in toluene at room temperature.

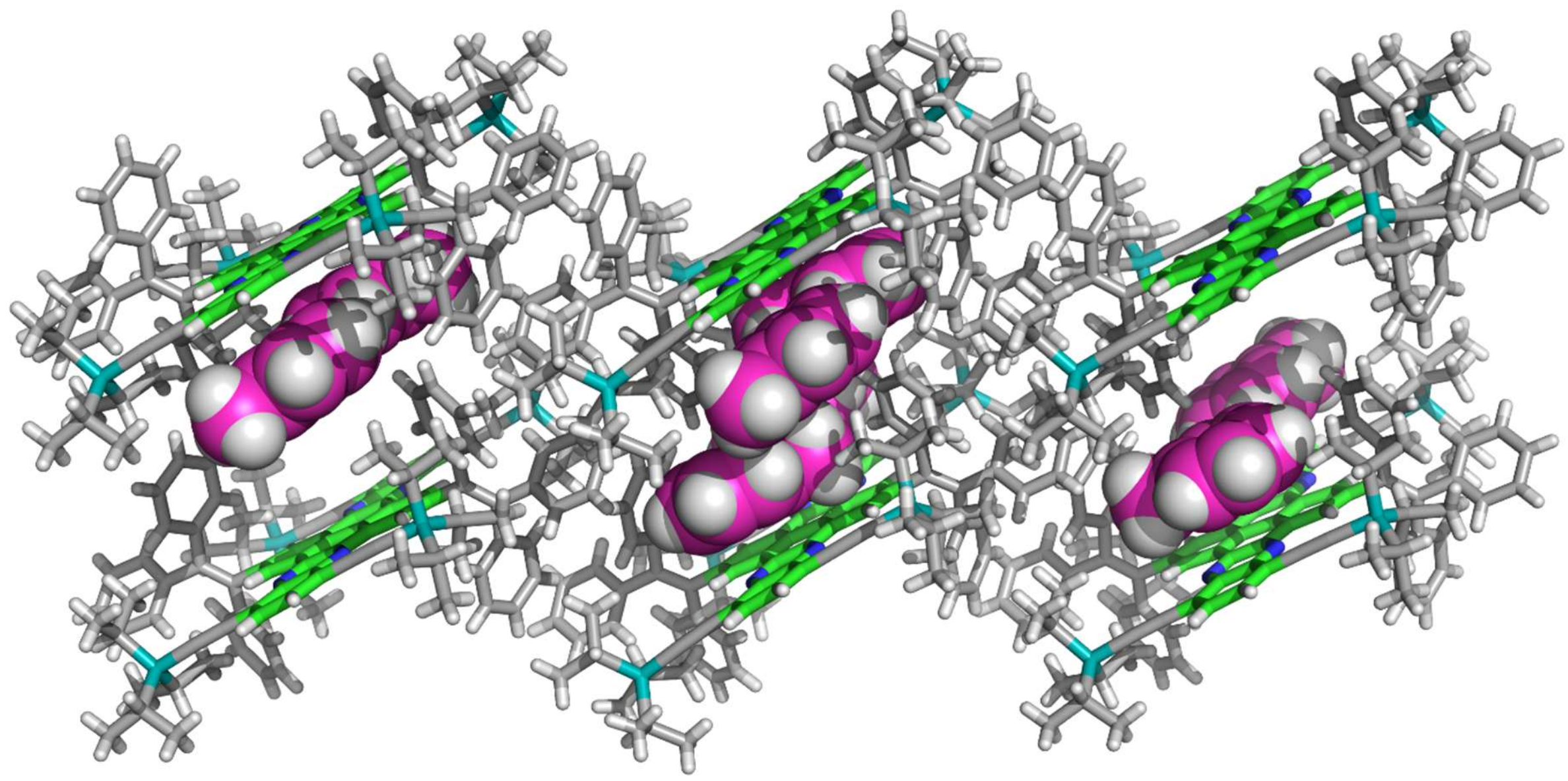

Figure S75. Packings observed in the single crystal X-ray structure of azaacene **2** with toluene molecules (magenta) in the crystal lattice. The single crystals were grown by slow diffusion of ethanol vapour to a solution of **2** in toluene at room temperature.

Table S5. Crystallographic data of compound **2**:

Colour, crystal shape & size in mm	Colourless, block, 0.21 × 0.11 × 0.08	
CCDC number	2496420	
Chemical formula & Formula weight	$C_{112}H_{122}N_4Si_4$, 1636.53	
Temperature	100 (2)	
Wavelength	0.71073	
Radiation source	MoKα	
Crystal system	Triclinic	
Space group	P–1	
Z	1	
Unit cell dimensions	a = 12.3310 (17) Å	α = 99.469 (5) °
	b = 15.282 (2) Å	β = 96.612 (5) °
	c = 33.097 (5) Å	γ = 103.281(5) °
Volume and density	5910.9 (14) $Å^3$, 1.126	
Absorption coefficient	0.102 mm^{-1}	
D*calc* (mgm^{-3})	1.126	
μ (mm^{-1})	0.102	
Reflections collected	25755	
Theta range for collection	1.896–26.999	
**Data collection -** Diffractometer	Bruker D8 VENTURE Kappa Duo PHOTON II CPAD	
Absorption correction	SADABS-2014/5	
T_{min}, T_{max}	0.7099, 0.7456	
No. of measured, independent & observed [I > 2σ(I)] reflections	272583, 25755, 21403	
Theta range (°)	1.90–27.00	
Rint	0.082	
**Refinement**		
R[F2 > 2σ (F^2)], wR(F^2)	0.05, 0.128	
GOF on F^2	1.06	
No. of independent reflections	25755	
No. of parameters	1365	
F_000	2154	
No. of restraints	0	
H-atom treatment	Mixed	

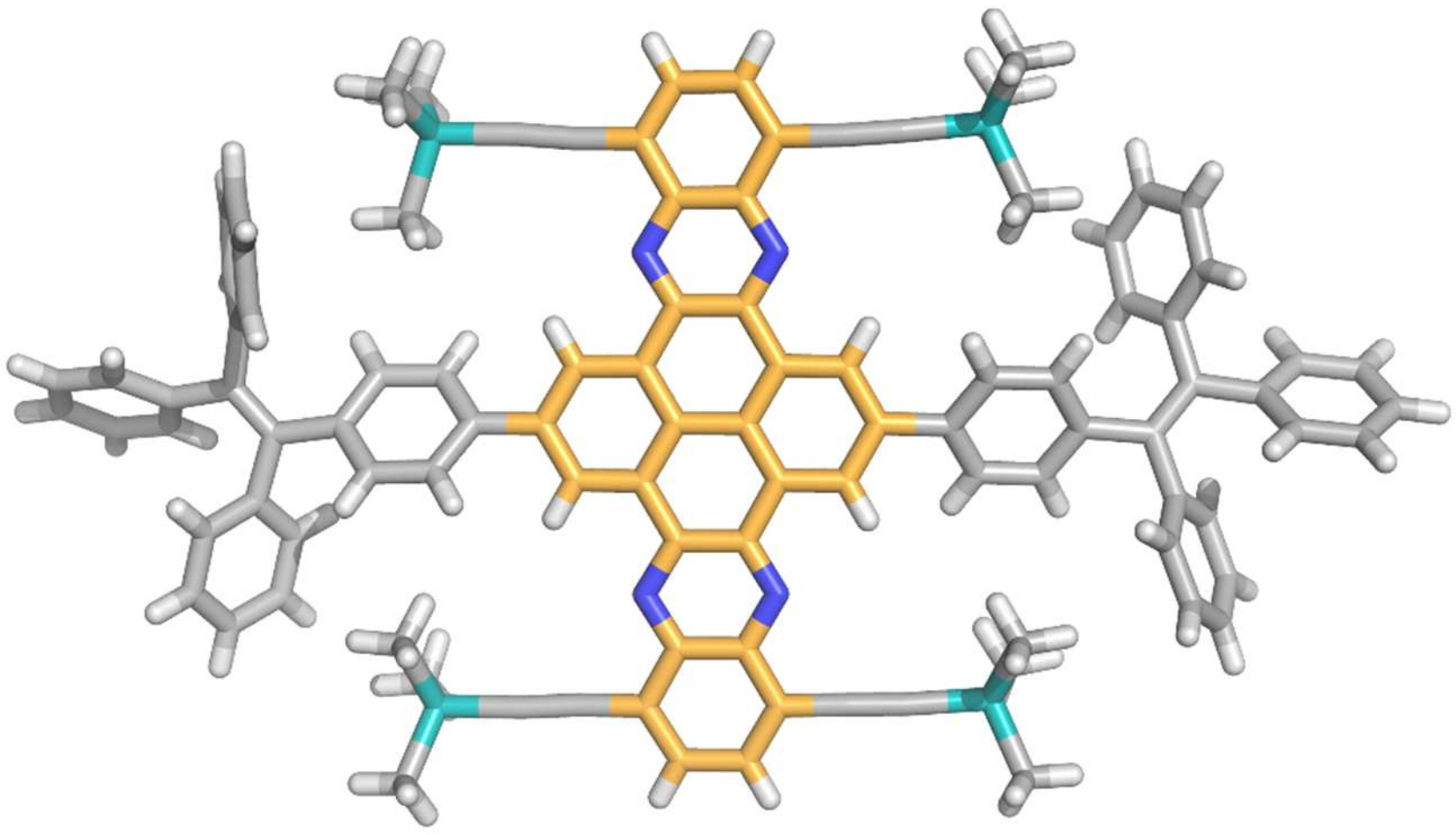

Figure S76. ORTEP diagram of **3**. Thermal ellipsoids are shown at 50% probability. The single crystals of **3** were grown by slow diffusion of ethanol vapours to a solution of **3** in toluene at room temperature.

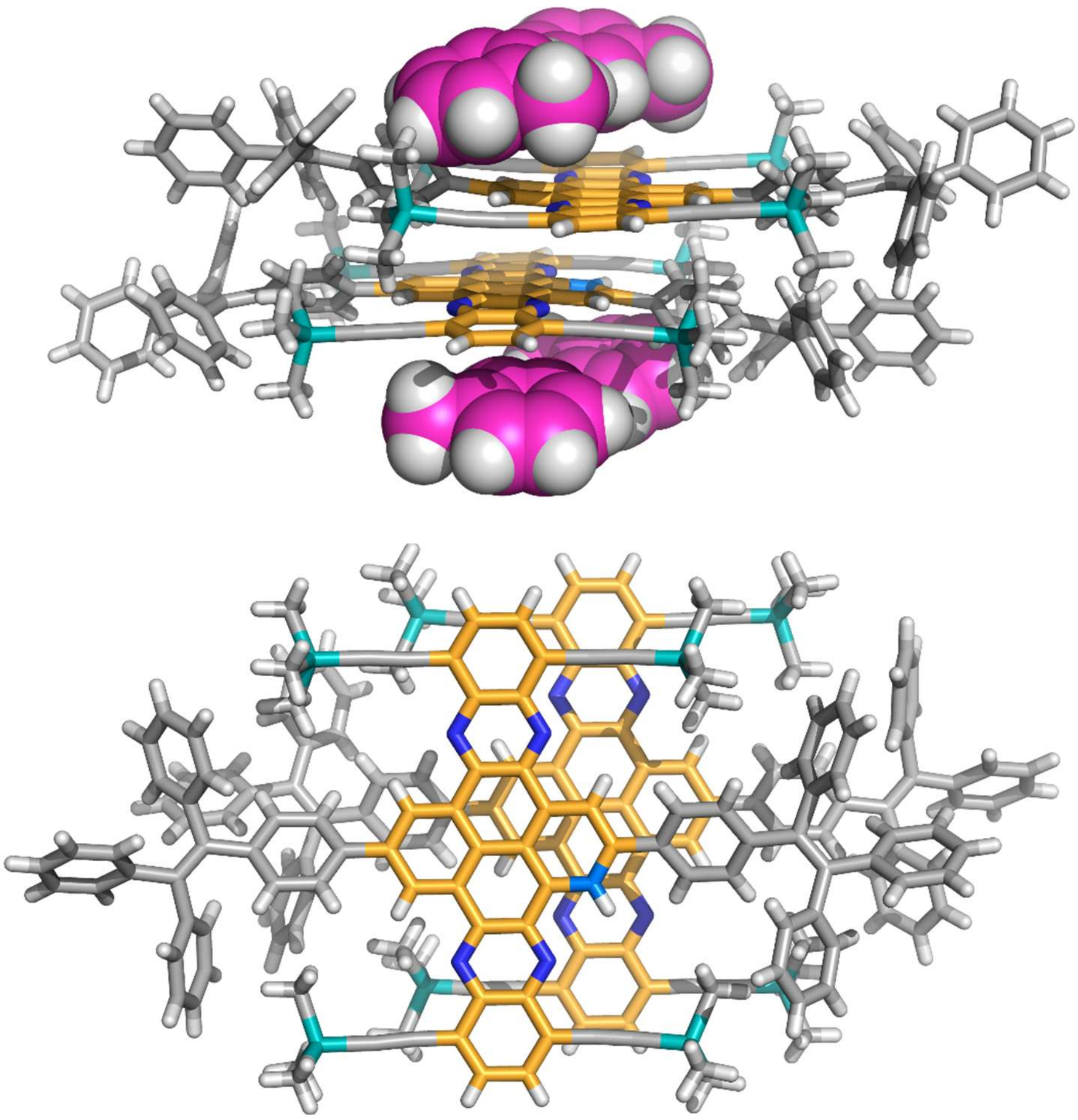

Figure S77. Packings observed in the single-crystal X-ray structure of azaacene **3** (side view and top view). The single crystals were grown by slow diffusion of ethanol vapour to a solution of **3** in toluene at room temperature.

Table S6. Crystallographic data of compound **3**:

Colour, crystal shape & size in mm	Colourless, needle, 0.24 × 0.11 × 0.08	
CCDC number	2496419	
Chemical formula & Formula weight	$C_{100}H_{74}N_4Si_4$, 1444.02	
Temperature	100 (2)	
Wavelength	0.71073	
Radiation source	MoKα	
Crystal system	Triclinic	
Space group	*P*–1	
Z	2	
Unit cell dimensions	a = 12.114 (15) Å	α = 72.89 (7) °
	b = 16.27 (3) Å	β = 80.52 (9) °
	c = 25.21 (4) Å	γ = 89.06 (11) °
Volume and density	4682 (13) $Å^3$, 1.161	
Absorption coefficient	0.115 mm^{-1}	
D*calc* (mgm^{-3})	1.161	
μ (mm^{-1})	0.115	
Reflections collected	20424	
Theta range for collection	2.53–27.26	
**Data collection -** Diffractometer	Bruker D8 VENTURE Kappa Duo PHOTON II CPAD	
Absorption correction	SADABS-2014/5	
T_{min}, T_{max}	0.6202, 0.7456	
No. of measured, independent & observed [I > 2σ(I)] reflections	209288, 20424, 9879	
Theta range (°)	2.09–27.00	
Rint	0.517	
**Refinement**		
R[F2 > 2σ (F^2)], wR(F^2)	0.177, 0.353	
GOF on F^2	1.152	
No. of independent reflections	20424	
No. of parameters	1011	
F_000	1732	
No. of restraints	15	
H-atom treatment	Constr	

8. DFT Calculations

All calculations were performed with Gaussian16 rev. C.02 suite of electronic structure programs.[8] The geometries of the potential energy minima or transition states were optimised at B3LYP/6-31G(d) level of theory. The nature of all stationary points was verified by frequency calculations. The frontier molecular orbitals were obtained on B3LYP/6-31G(d) level of theory. The absorption spectra were obtained using the TD-CAM-B3LYP method with 6-31G(d) basis set, calculating the first 10 singlet excitations.

Table S7. HOMO–3, HOMO–2, HOMO–1, HOMO, LUMO, and LUMO+1 representation of **1** (level of theory: CAM-B3LYP/6-31G(d)).

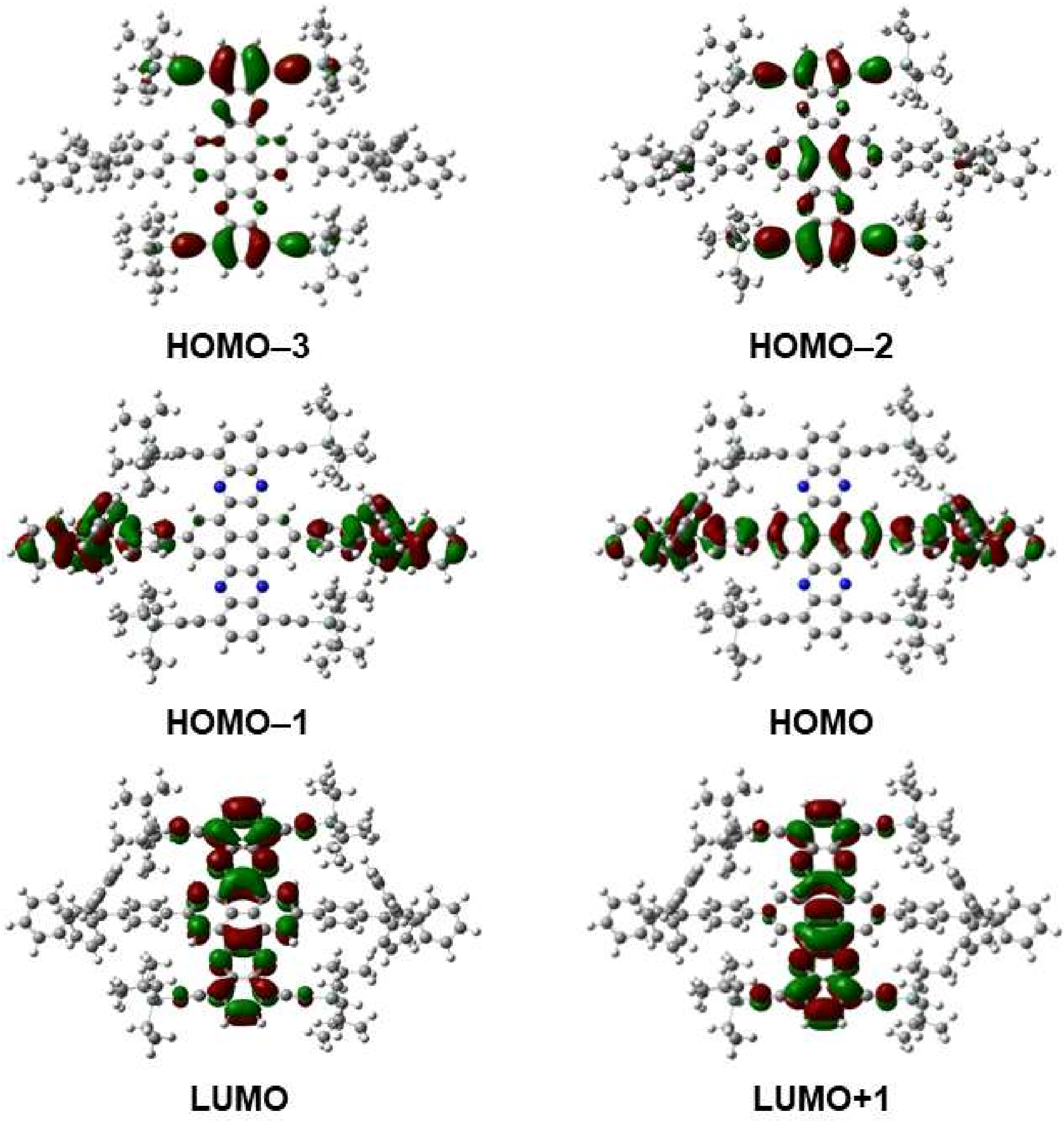

Transition	FMOs	Calc. λ_{max} [nm]	f_{os} of λ_{max}
$S_0 \rightarrow S_1$	HOMO→LUMO (0.34)<br>HOMO–2→LUMO (0.42)<br>HOMO–3→LUMO+1 (0.37)	379.32	0.9154
$S_0 \rightarrow S_2$	HOMO→LUMO+1 (0.32)<br>HOMO–2→LUMO+1 (0.45)<br>HOMO–3→LUMO (0.39)	377.68	0.0004
$S_0 \rightarrow S_3$	HOMO→LUMO+2 (0.13)<br>HOMO–2→LUMO+2 (0.13)<br>HOMO–4→LUMO (0.60)	358.03	0.5422

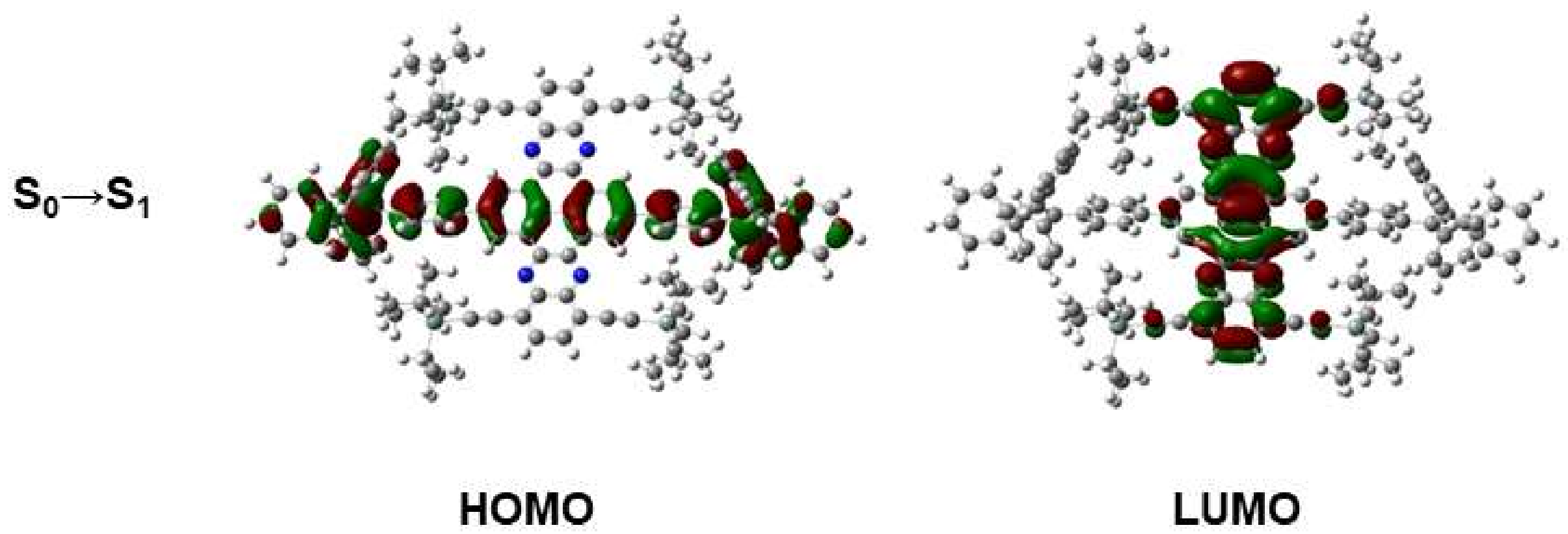

Figure S78. Natural transition orbitals (NTOs) of **1** for the $S_0 \rightarrow S_1$ transition.

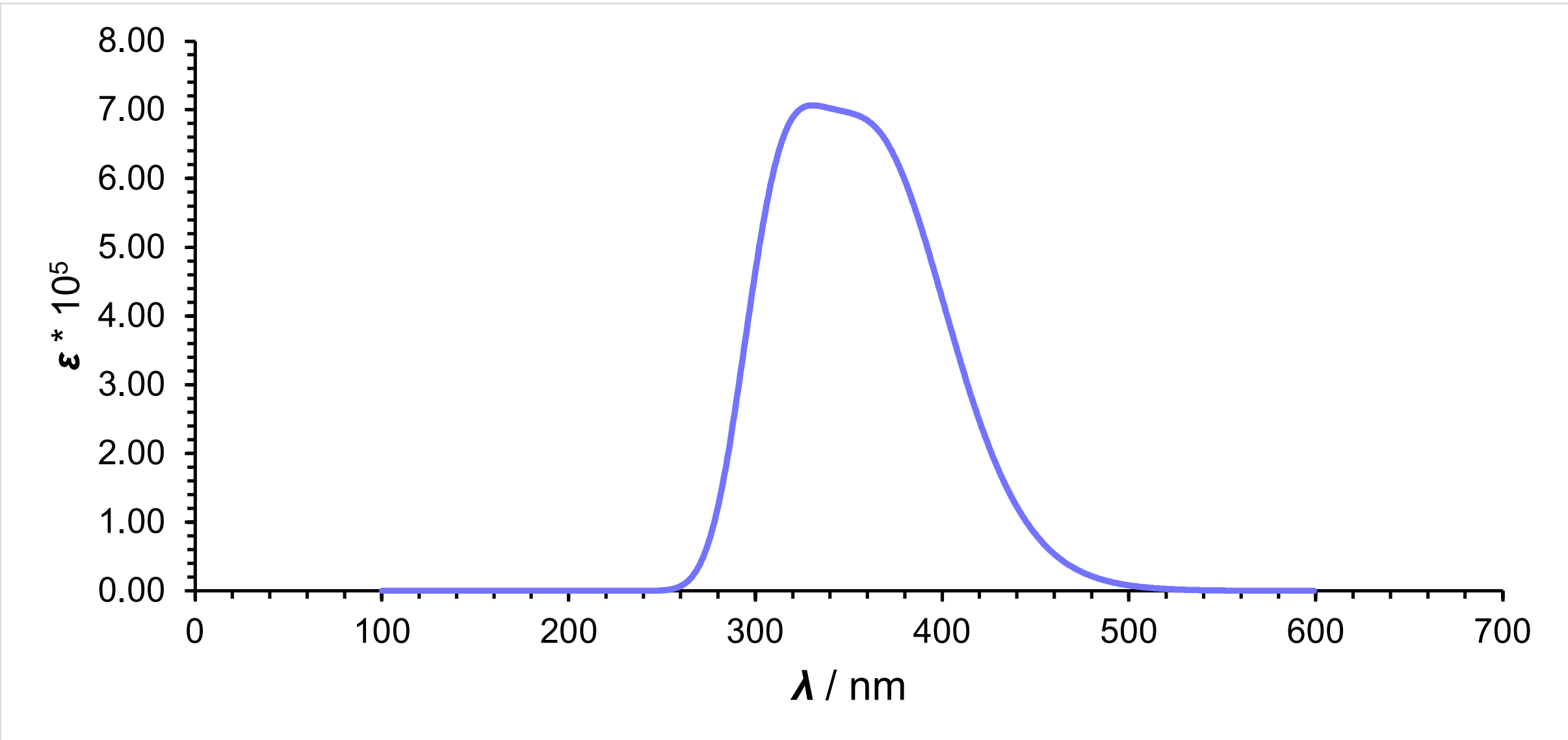

Figure S79. Calculated absorption spectrum of **1** (level of theory: CAM-B3LYP/6-31G(d)).

Table S8. HOMO–2, HOMO–1, HOMO, LUMO, LUMO+1. and LUMO+2 representation of **2** (level of theory: CAM-B3LYP/6-31G(d)).

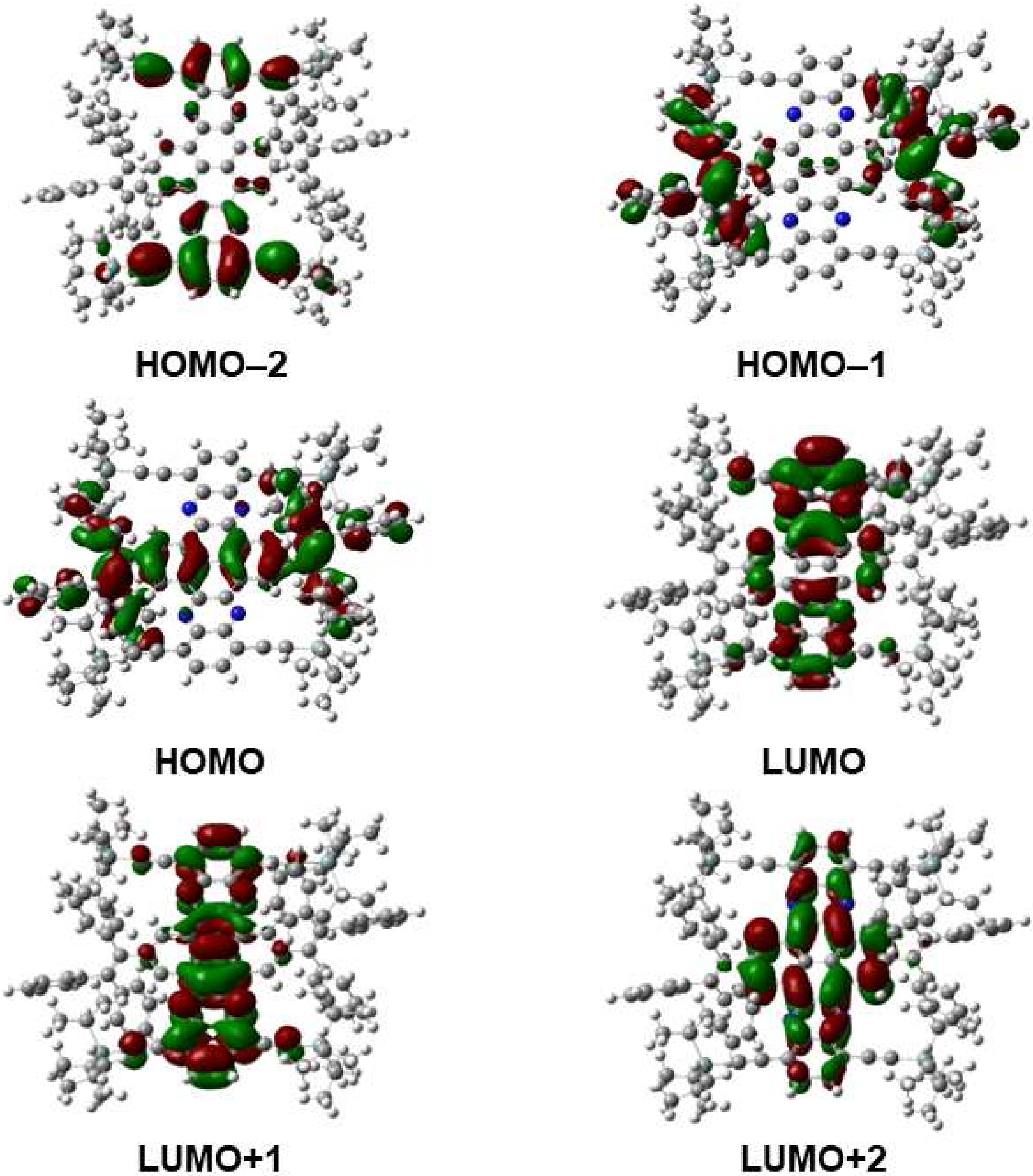

Transition	FMOs	Calc. λ_{max} [nm]	f_{os} *of* λ_{max}
$S_0 \rightarrow S_1$	HOMO→LUMO (0.44)<br>HOMO–2→LUMO (0.17)	379.16	0.6561
$S_0 \rightarrow S_2$	HOMO→LUMO+1 (0.45)<br>HOMO–2→LUMO (–0.34)<br>HOMO–2→LUMO+1 (–0.35)	377.72	0.0171
$S_0 \rightarrow S_3$	HOMO–4→LUMO (0.58)<br>HOMO–4→LUMO+1 (0.15)	360.29	0.4961

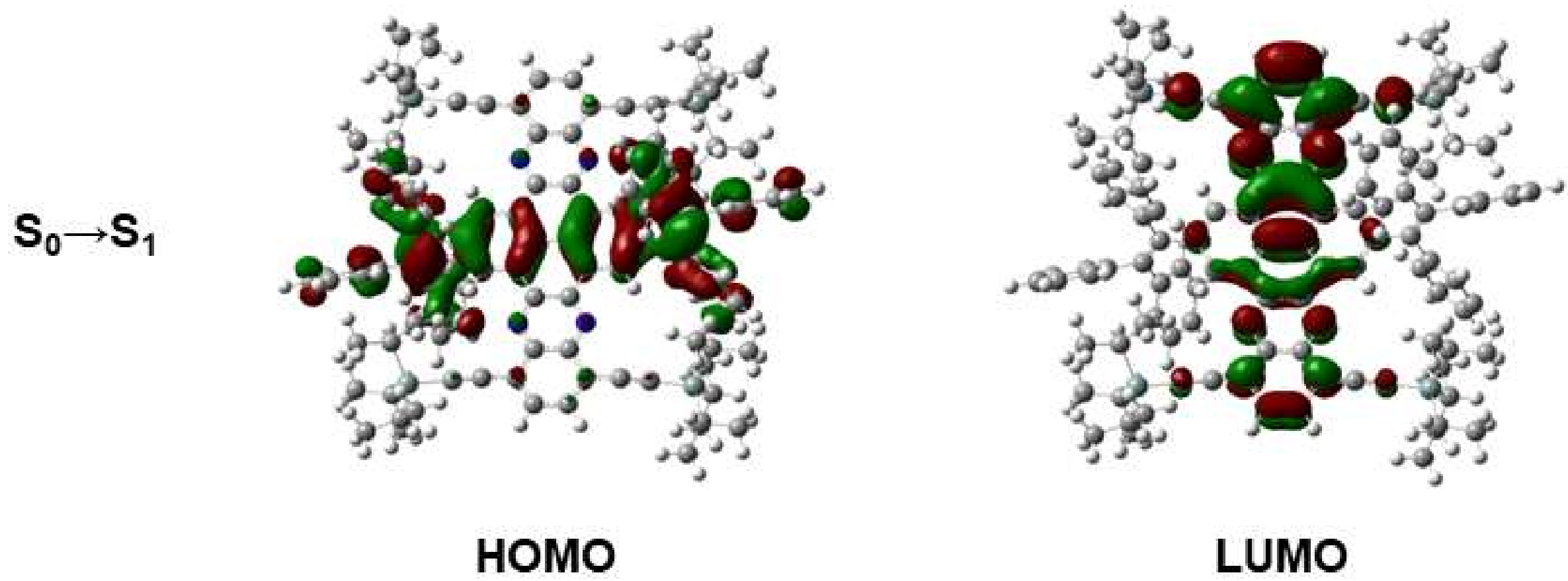

Figure S80. Natural transition orbitals (NTOs) of **2** for the $S_0 \rightarrow S_1$ transition.

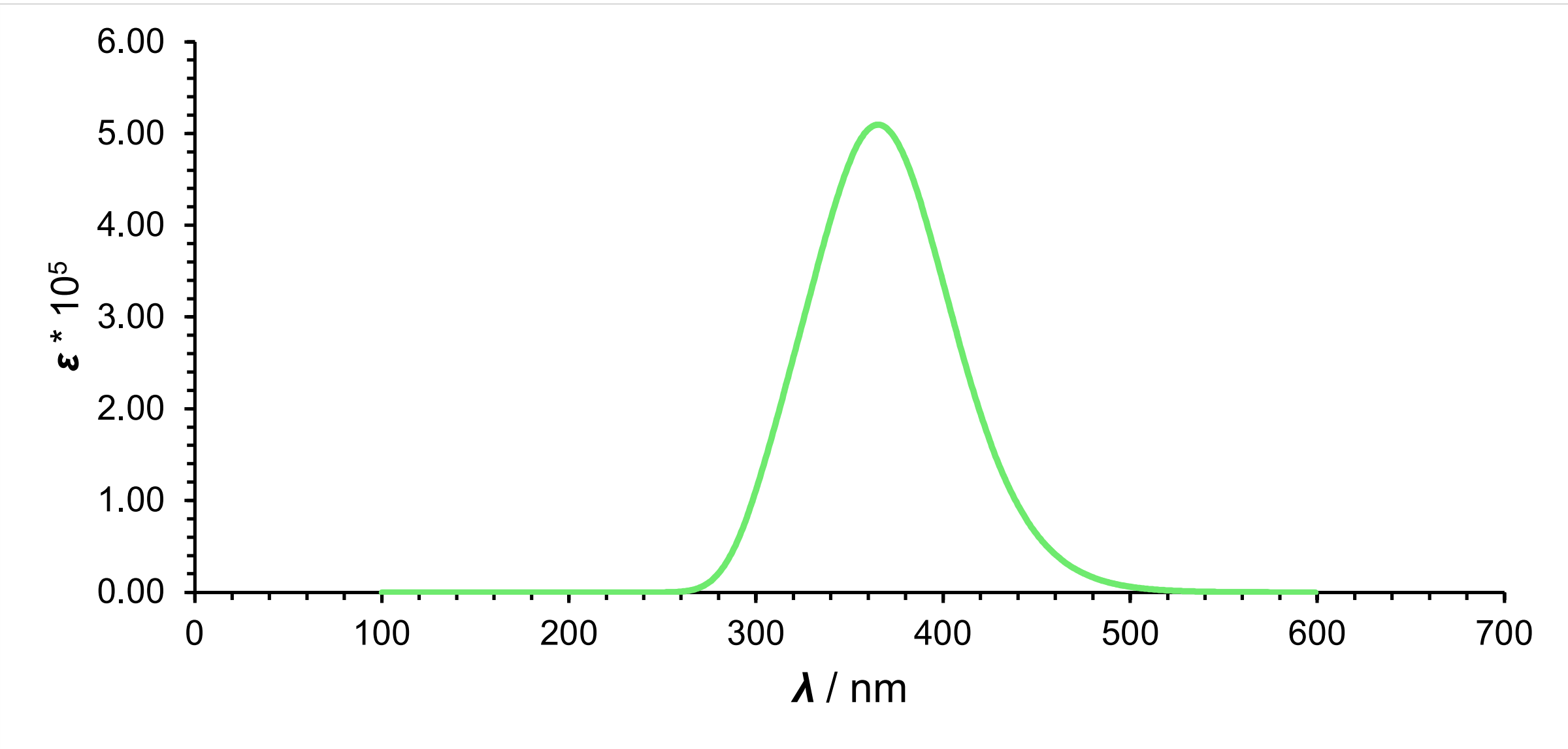

Figure **S81**. Calculated absorption spectrum of **2** (level of theory: CAM-B3LYP/6-31G(d)).

Table S9. HOMO–2, HOMO–1, HOMO, LUMO, LUMO+1. and LUMO+2 representation of **3** (level of theory: CAM-B3LYP/6-31G(d)).

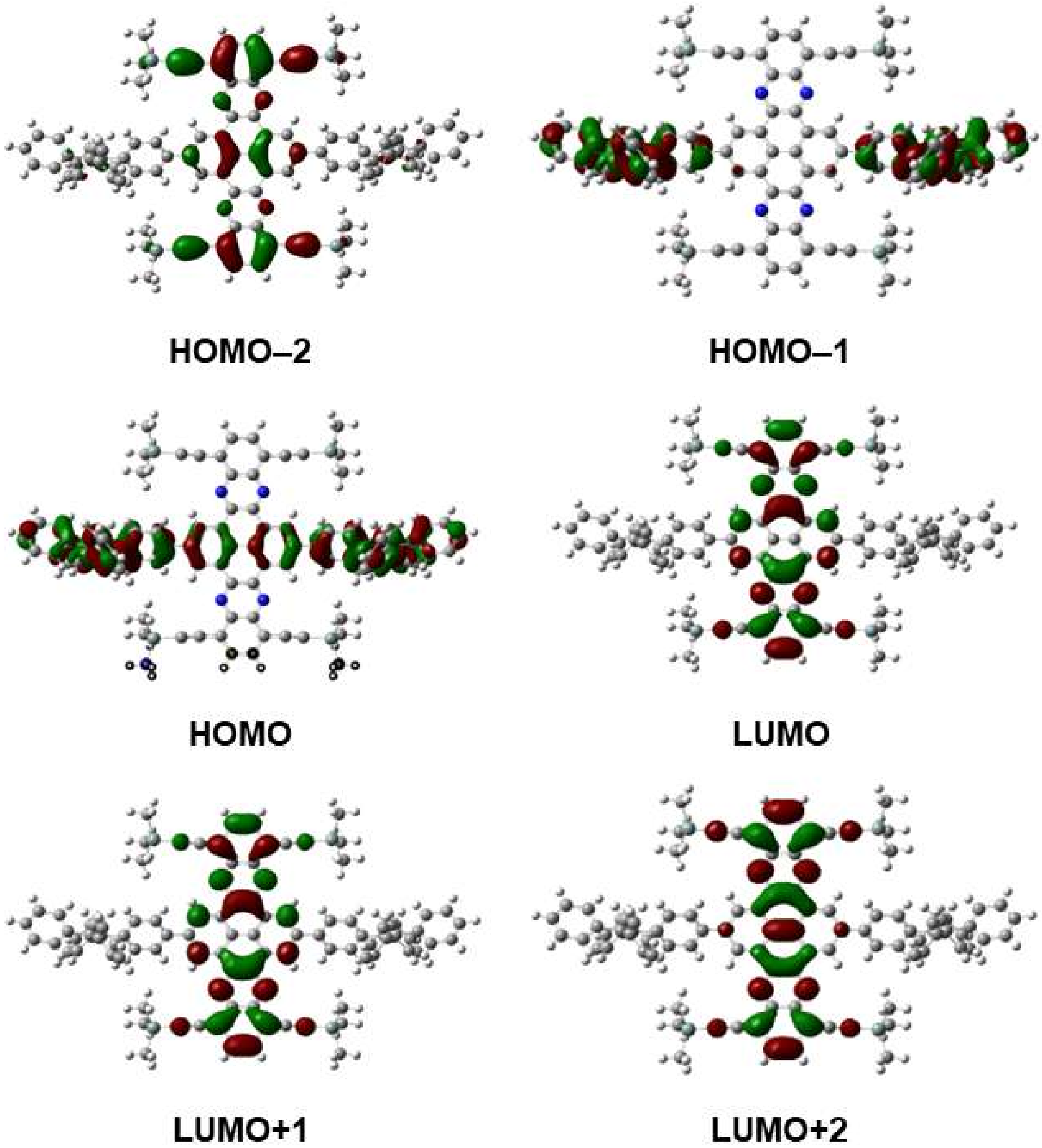

Transition	FMOs	Calc. λ_{max} [nm]	f_{os} *of* λ_{max}
$S_0 \rightarrow S_1$	HOMO→LUMO (0.35)<br>HOMO–2→LUMO (0.43)<br>HOMO–3→LUMO+1 (0.38)	376.44	0.8204
$S_0 \rightarrow S_2$	HOMO→LUMO+1 (0.33)<br>HOMO–2→LUMO+1 (0.45)<br>HOMO–3→LUMO (0.39)	374.40	0.0011
$S_0 \rightarrow S_3$	HOMO→LUMO+2 (0.14)<br>HOMO–2→LUMO+2 (0.13)<br>HOMO–4→LUMO (0.61)	356.92	0.5379

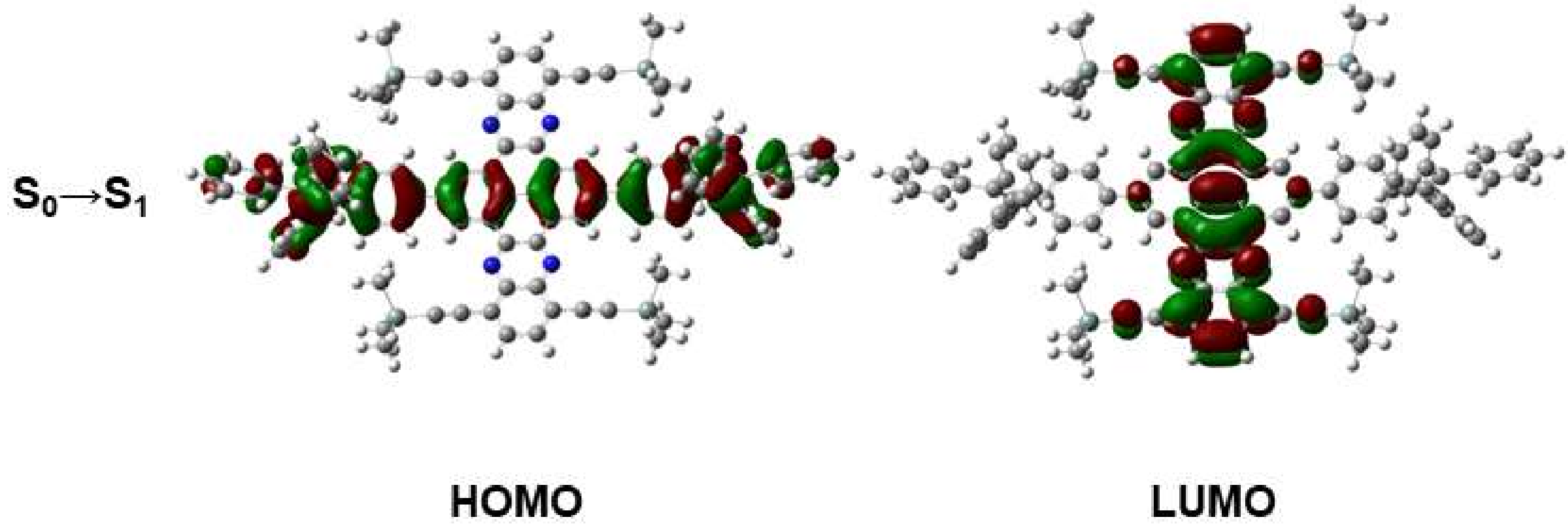

Figure S82. Natural transition orbitals (NTOs) of **3** for the $S_0 \rightarrow S_1$ transition.

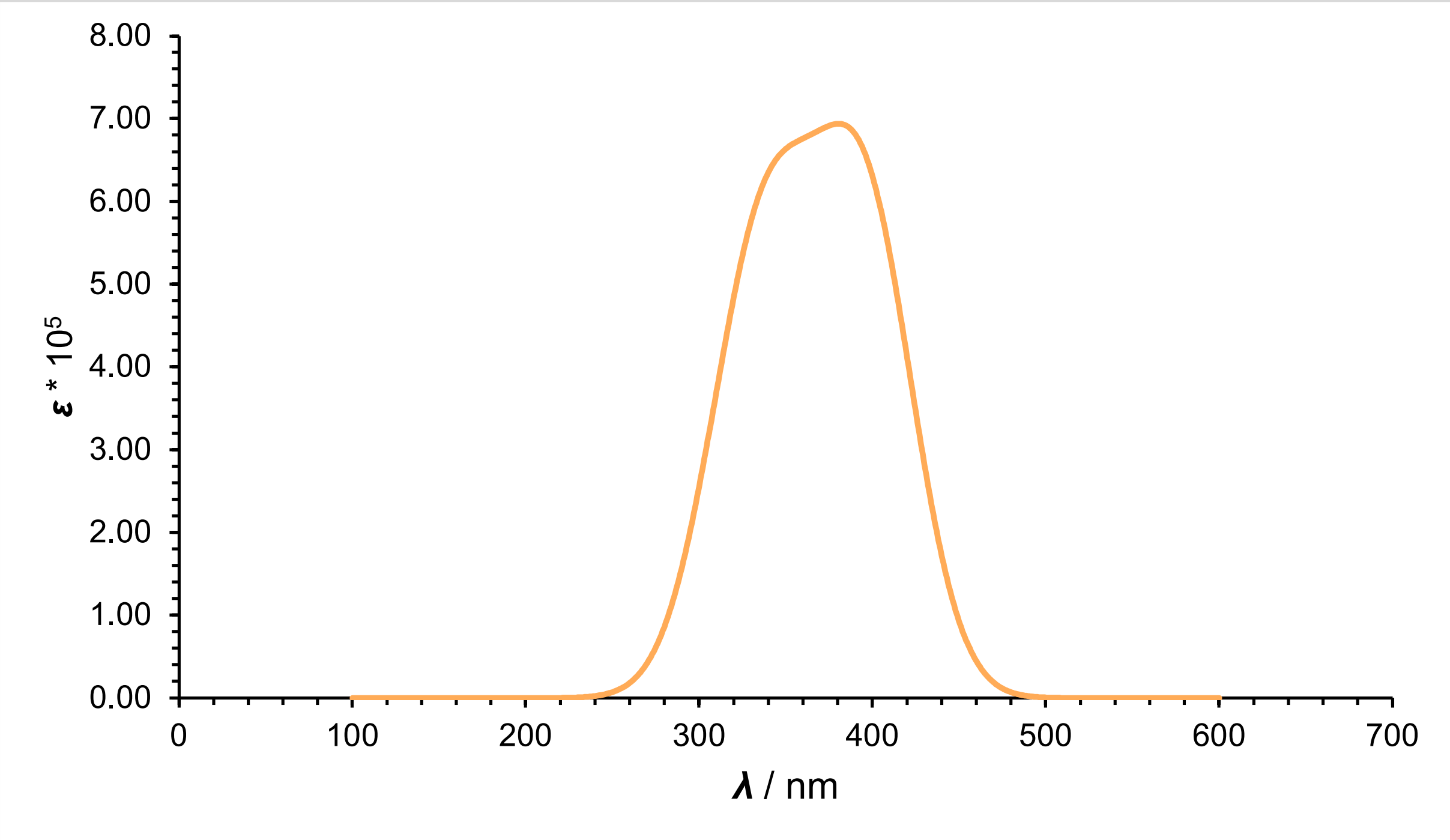

Figure S83. Calculated absorption spectrum of **3** (level of theory: CAM-B3LYP/6-31G(d)).

Table S10. HOMO–2, HOMO–1, HOMO, LUMO, LUMO+1. and LUMO+2 representation of **4** (level of theory: CAM-B3LYP/6-31G(d)).

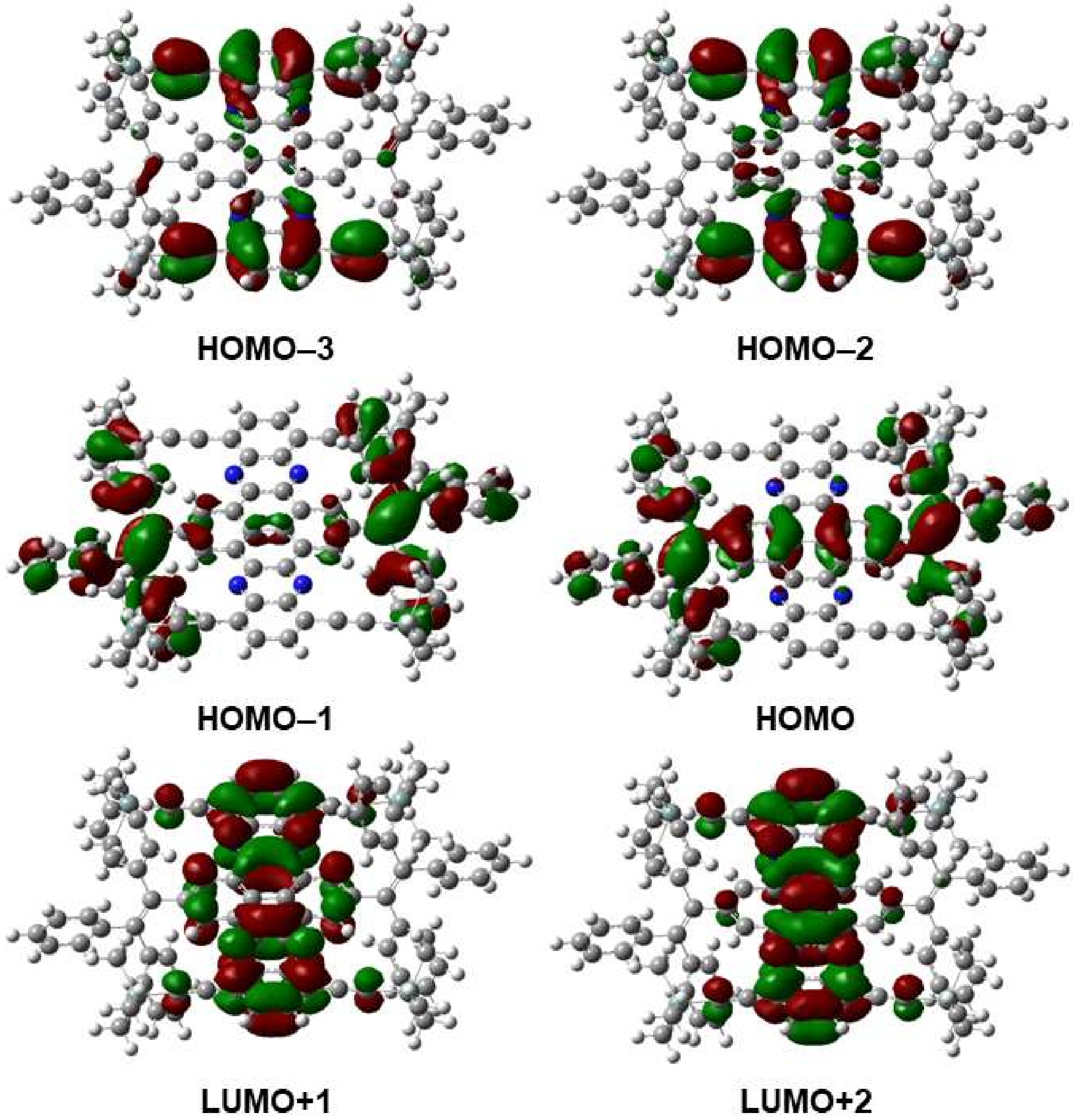

Transition	FMOs	Calc. λ_{max} [nm]	f_{os} *of* λ_{max}
$S_0 \rightarrow S_1$	HOMO→LUMO (0.49)<br>HOMO–2→LUMO+1 (0.34)<br>HOMO–3→LUMO+1 (0.31)	378.30	0.6193
$S_0 \rightarrow S_2$	HOMO→LUMO+1 (0.49)<br>HOMO–2→LUMO (0.34)<br>HOMO–3→LUMO+1 (0.33)	376.21	0.0000
$S_0 \rightarrow S_3$	HOMO→LUMO+2 (– 0.20)<br>HOMO–1→LUMO+1 (–0.14)<br>HOMO–4→LUMO (0.60)	360.21	0.4567

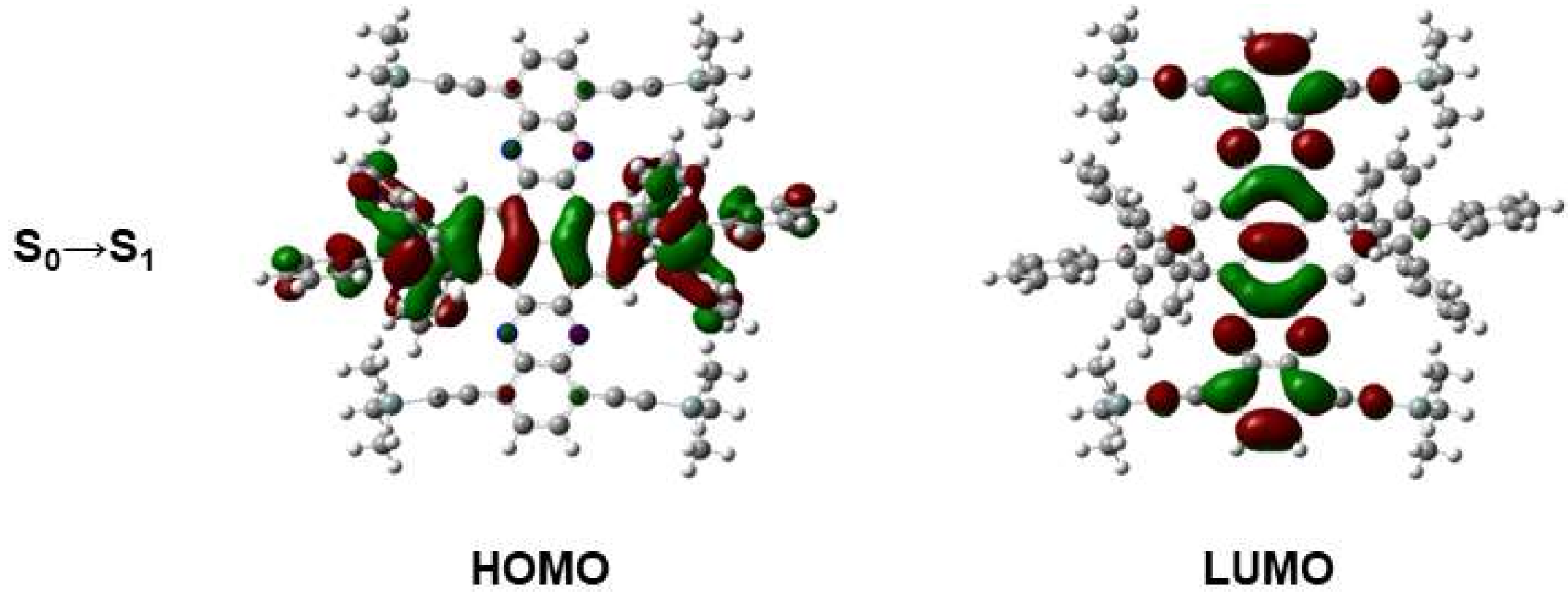

Figure S84. Natural transition orbitals (NTOs) of **4** for the $S_0 \rightarrow S_1$ transition.

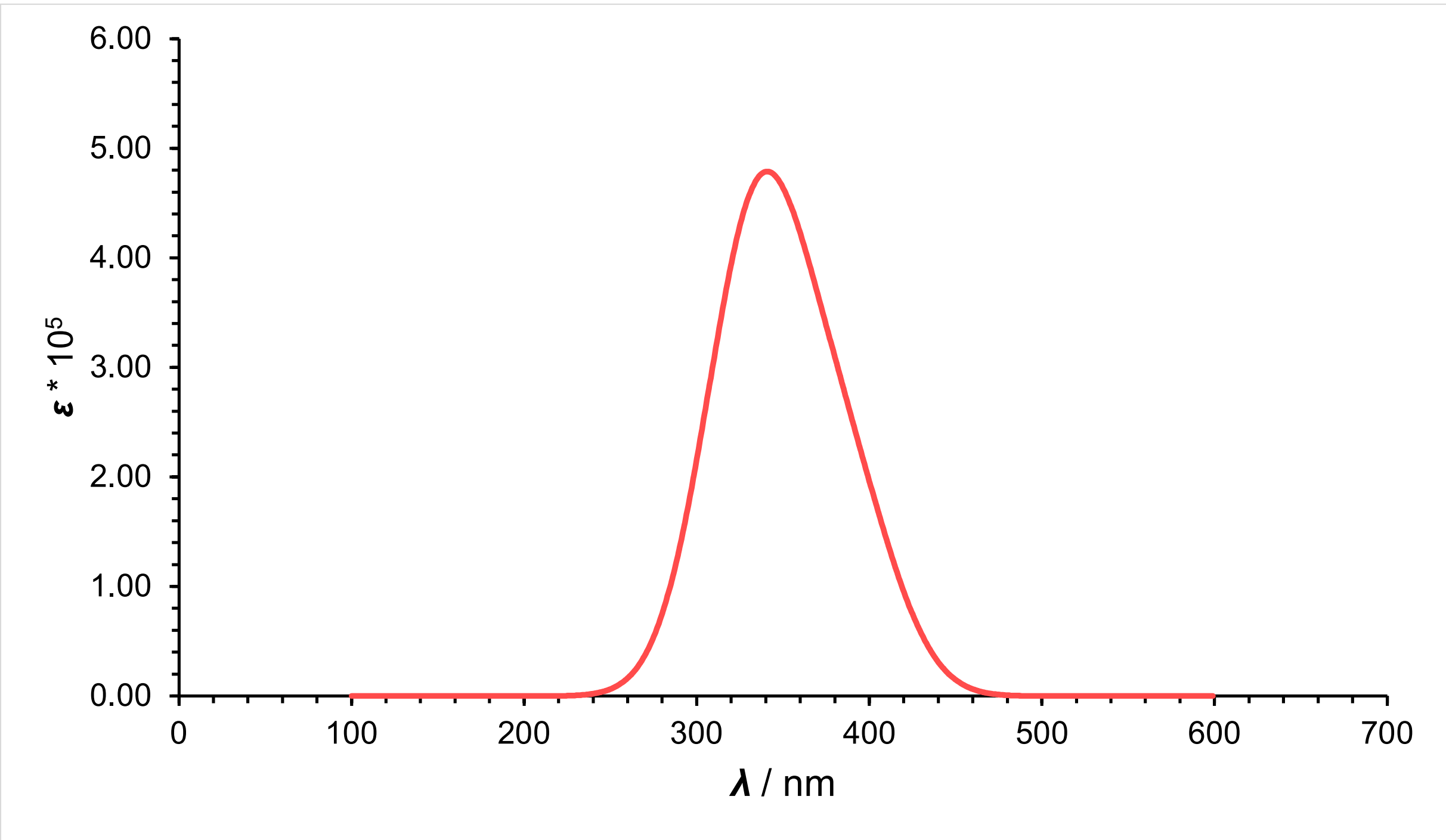

Figure **S85**. Calculated absorption spectrum of **4** (level of theory: CAM-B3LYP/6-31G(d)).

Table S11. Calculated energies[a] of the frontier MOs of **1**–**4** (level of theory: CAM-B3LYP/6-31G(d)), and experimental values obtained by CV experiments.

**Compound**	E_{HOMO}[a] / [eV]	E_{LUMO}[a] / [eV]	E_{HOMO}[b] / [eV]	E_{LUMO}[c] / [eV]	$E^{\mathrm{I}}_{1/2}$[d] [eV]	$E^{\mathrm{II}}_{1/2}$[d] [eV]	E_{pc}[d] [eV]
**1**	–6.48	–3.21	–6.20	–3.47	−1.33	−1.57	−1.73
**2**	–6.54	–3.27	–6.23	–3.52	−1.28	−1.40	−1.93
**3**	–6.48	–3.19	–6.25	–3.53	−1.27	−1.52	−1.69
**4**	–6.49	–3.21	–6.35	–3.62	−1.18	−1.29	−1.48

[a]The energy of HOMO is taken from the KS wavefunction obtained by the SCF process. The energy of LUMO is calculated as the energy of HOMO with the addition of the $S_0 \rightarrow S_1$ vertical excitation energy

from the TD-CAM-B3LYP calculation. [b]$E_{HOMO} = E_{LUMO} - E_g$. [c]$E_{LUMO} = -E^{I}_{onset} + 4.80$ eV. (E^{I}_{onset} = onset potential of the first reduction wave). [d]Measured in CH_2Cl_2 containing 0.1 M n-Bu_4NPF_6.

9. Aggregation investigation in THF-Water mixtures

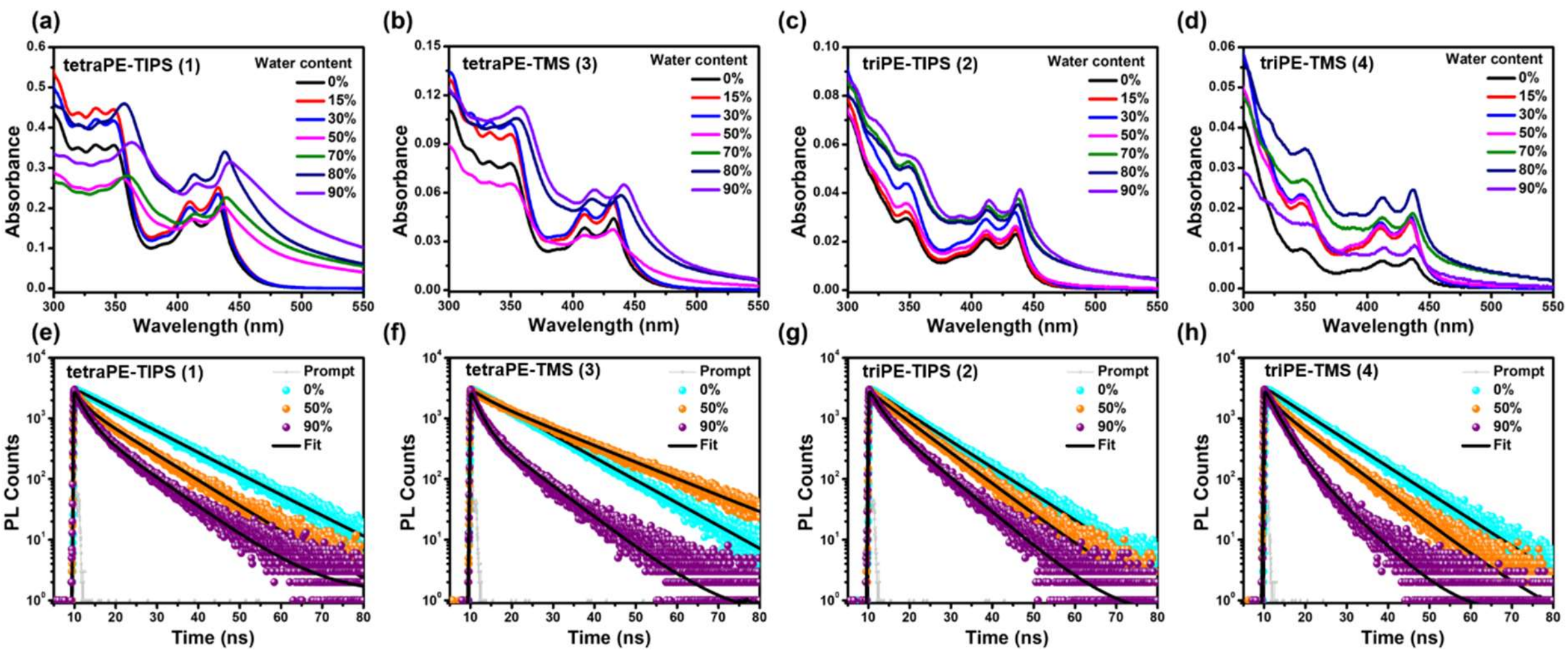

Figure **S86**. Absorption spectra and lifetime of (**a**,**e**) tetraPE-TIPS (**1**), (**b**,**f**) tetraPE-TMS (**3**), (**c**,**g**) triPE-TIPS (**2**), and (**d**,**h**) triPE-TMS (**4**) in THF/water binary mixtures.

Table S12. Photoluminescence lifetime of all luminogens **1-4** in THF/water mixtures.

Water %	tetraPE-TIPS (**1**)	tetraPE-TMS (**3**)	triPE-TIPS (**2**)	triPE-TMS (**4**)
0%	12.3 ns	11.16 ns	9.65 ns	9.85ns
50%	6.63 ns	13.19 ns	7.06 ns	6.22 ns
90%	4.58 ns	3.72 ns	4.75 ns	3.66 ns

10. Hirshfeld surface analysis

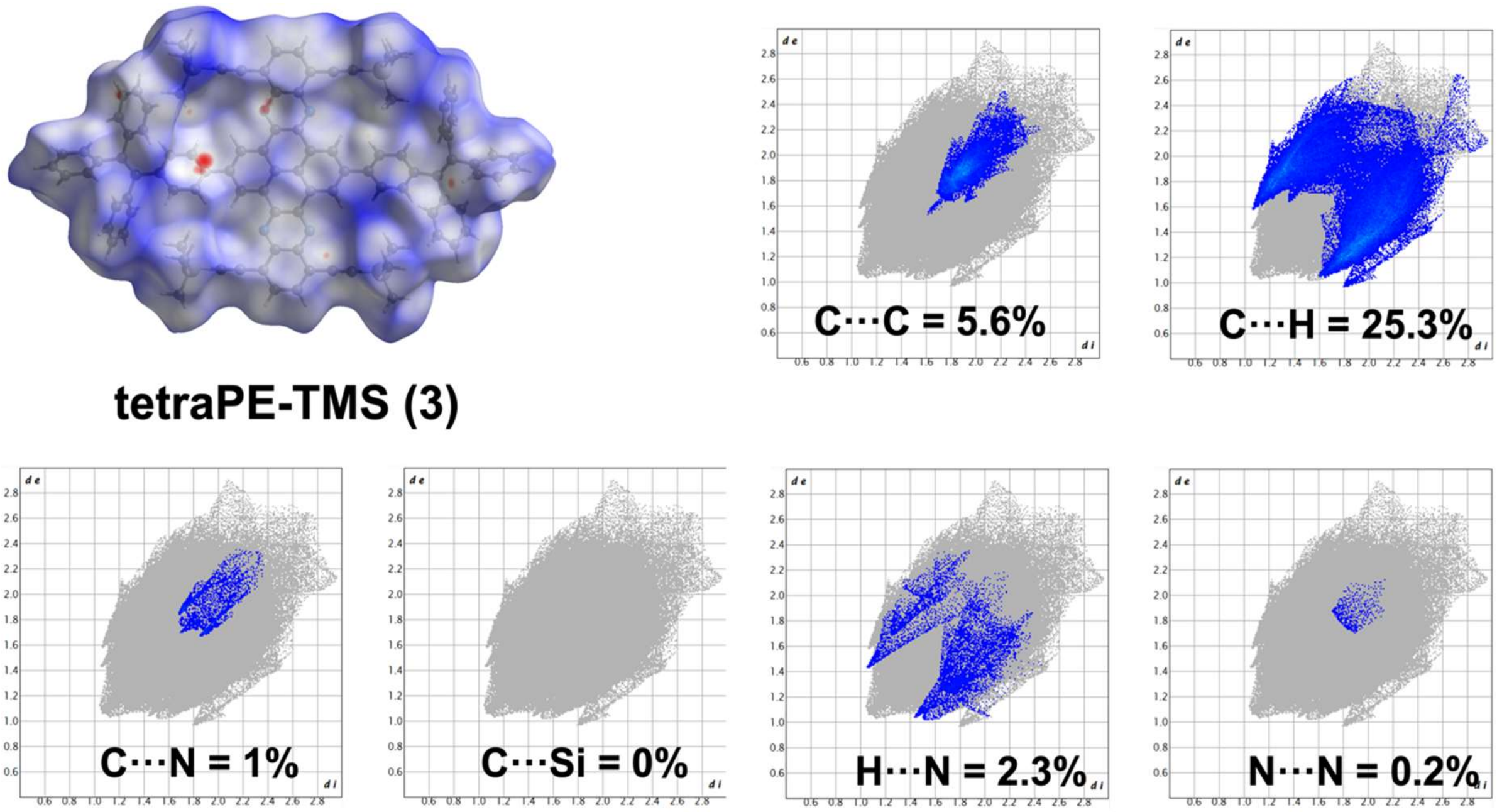

Figure **S87**. Normalized distance (d_{norm}) mapped over the Hirshfeld surface of azaacene **3** (top left), generated fingerprint plots from d_{norm} for C···C (top right), C···N, C···Si, H···N and N···N interactions (bottom). The grey part in 2D finger plots indicates the total interactions calculated from dnorm.

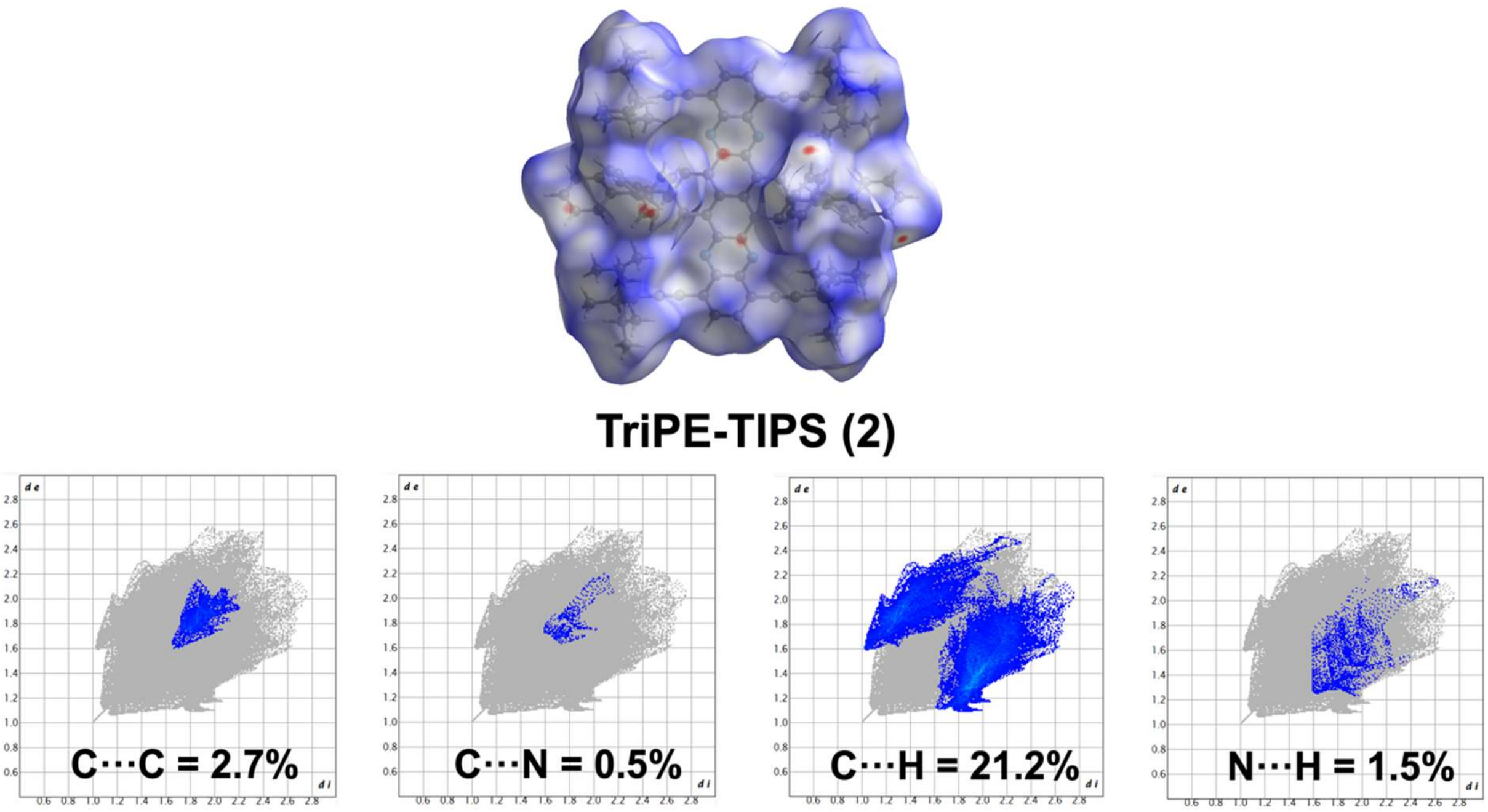

Figure **S88**. Normalized distance (d_{norm}) mapped over the Hirshfeld surface of azaacene **2** (top), generated fingerprint plots from d_{norm} for C···C, C···N, C···H, and N···H interactions (bottom). The grey part in 2D finger plots indicates the total interactions calculated from dnorm.

Cartesian Coordinates:

1, CAM-B3LYP/6-31G(d), E = –6177.399675 Hartree.

C	1.43862300	1.28128000	-0.14935700
C	2.83741800	1.27628600	-0.17043500
C	3.56617500	0.07822100	-0.16431600
C	2.85662100	-1.12981000	-0.12855300
C	1.46005000	-1.15789300	-0.11094500
C	0.72447100	0.05474700	-0.12707900
C	-0.72406000	0.03808000	-0.12347800
C	-1.43599400	-1.18845900	-0.09970700
C	-2.83337600	-1.18757300	-0.09281000
C	-3.56694600	0.00662100	-0.11403500
C	-2.86097200	1.21716700	-0.14544100
C	-1.46292800	1.24780200	-0.14892400
C	0.74376400	-2.43536200	-0.09354600
C	-0.69357100	-2.45039500	-0.09478000
C	-0.74567500	2.52437700	-0.16346800
C	0.69325400	2.54363500	-0.14518600
N	1.43963800	-3.56532600	-0.07865900
C	0.76577200	-4.73332100	-0.07204900
C	-0.66777100	-4.74797400	-0.08613600
N	-1.36577200	-3.59446800	-0.09406000
N	-1.44478600	3.65138500	-0.19118000
C	-0.77634500	4.82182200	-0.18485700
C	0.65690500	4.84316200	-0.13596100
N	1.36035900	3.69148600	-0.12475800
C	-1.51561000	6.05523700	-0.22181800
C	-0.80470700	7.25033800	-0.19910800
C	0.59845000	7.27276800	-0.13428900
C	1.35099400	6.10294800	-0.09835600
C	1.49599000	-5.97309100	-0.05418700
C	0.77471500	-7.16255900	-0.05579400
C	-0.63083400	-7.17680300	-0.07606900
C	-1.37419100	-6.00151800	-0.09266000
C	2.76845200	6.18069200	-0.01124500
C	3.97563200	6.34940100	0.08024400
C	-2.93687100	6.07194700	-0.27873100
C	-4.15380200	6.17657500	-0.32795100
Si	-5.98724000	6.37046100	-0.41748100
C	-6.74417700	5.00001400	0.68866600
C	-6.45122200	8.11655700	0.24542900
C	-6.50339200	6.12493700	-2.24690000
Si	5.79005200	6.64564800	0.25014900
C	6.02787300	8.49866200	0.68617700
C	6.39418700	5.55967600	1.71152000
C	6.56691100	6.29745000	-1.46918000
C	5.04909700	0.07474400	-0.20813000
C	5.79238700	-0.87595800	0.51178100
C	7.18186900	-0.90120500	0.45161400
C	7.89440700	0.02240000	-0.33303300
C	7.14785000	0.96413300	-1.06349700

C	5.75795300	0.99844000	-0.99343400
C	9.38214300	-0.02770400	-0.45004400
C	9.90267700	0.10337100	-1.84671900
C	10.20954000	-0.18145000	0.62738200
C	9.73396100	-0.05433500	2.03894900
C	11.66553000	-0.49146100	0.48821900
C	10.90113400	1.03871400	-2.16620600
C	11.35759800	1.17983100	-3.47596300
C	10.82630000	0.38753900	-4.49535200
C	9.82633600	-0.53994700	-4.19518500
C	9.36210400	-0.67231800	-2.88741100
C	12.62085000	0.22654100	1.22935000
C	13.97895500	-0.07004500	1.12674900
C	14.41074800	-1.10787200	0.29812700
C	13.47194300	-1.84455300	-0.42668300
C	12.11498400	-1.53921400	-0.33338900
C	10.07692600	-1.02573100	2.99655900
C	9.65413700	-0.90820400	4.31982200
C	8.89940300	0.19642600	4.72070600
C	8.57222900	1.18090700	3.78635100
C	8.98404100	1.05655500	2.46017900
C	-5.05018000	-0.01566000	-0.10567400
C	-5.75635500	-0.97768200	0.63614400
C	-7.14731500	-1.00622200	0.63895200
C	-7.89697000	-0.07763800	-0.10335600
C	-7.18805000	0.87283700	-0.85926000
C	-5.79689100	0.91255400	-0.85057100
C	-9.38852000	-0.13319000	-0.15009100
C	-9.97265800	-0.03609600	-1.52362400
C	-10.16105300	-0.26424200	0.97033800
C	-11.00603200	0.87056100	-1.81443500
C	-11.52149200	0.97916400	-3.10517700
C	-11.01549400	0.18323400	-4.13466300
C	-9.98074200	-0.71465200	-3.86419200
C	-9.45812800	-0.81447000	-2.57571000
C	-9.61748500	-0.09488400	2.35243200
C	-11.61951500	-0.58749700	0.90936600
C	-12.54554600	0.14532700	1.67275500
C	-13.90441200	-0.16373900	1.64160900
C	-14.36511400	-1.22839200	0.86412400
C	-13.45458200	-1.97937900	0.11821900
C	-12.09734200	-1.66194600	0.13993300
C	-9.92693900	-1.02518500	3.36062500
C	-9.44318900	-0.86382400	4.65799900
C	-8.65740100	0.24404300	4.98254600
C	-8.35999400	1.18784300	3.99751600
C	-8.83401900	1.01951000	2.69759500
C	2.91809900	-5.99659400	-0.03621700
C	-2.79522500	-6.04564400	-0.12150600
C	-4.01341500	-6.13959900	-0.15418800
C	4.13663700	-6.09198100	-0.01921100
Si	5.97773100	-6.22926500	-0.00395400
C	6.60335200	-4.95805500	1.28832300
C	6.45537600	-8.02089800	0.50777700
Si	-5.85235200	-6.29403100	-0.20173000
C	-6.26740900	-7.99003800	-0.99239500

C	-6.43760300	-6.25981000	1.62570700
C	-6.56500400	-4.81483500	-1.19903800
C	6.60471400	-5.77006800	-1.75545200
H	3.34866100	2.23267800	-0.17226200
H	3.38131800	-2.07840400	-0.14394800
H	-3.33956200	-2.14629500	-0.09465500
H	-3.38800700	2.16476400	-0.14186200
H	-1.35179300	8.18704300	-0.22751900
H	1.11477700	8.22680600	-0.10829300
H	1.31583100	-8.10310200	-0.04338500
H	-1.15348400	-8.12779100	-0.08039300
H	-6.39257900	4.05380200	0.25076000
H	-6.53745100	7.98432000	1.33517400
H	-7.58865400	6.30860400	-2.28295300
H	7.11409800	8.64908900	0.79219500
H	5.90547000	6.02188600	2.58330000
H	5.98117700	6.94275600	-2.14223700
H	5.27581700	-1.59552900	1.14076600
H	7.72621500	-1.64565800	1.02299600
H	7.66526100	1.67246100	-1.70369900
H	5.21426300	1.73131400	-1.58276500
H	11.31872700	1.65786300	-1.37828100
H	12.12768400	1.91334500	-3.70036700
H	11.18328200	0.49652400	-5.51602300
H	9.40263100	-1.15901000	-4.98178400
H	8.57513100	-1.38761700	-2.66444300
H	12.29124700	1.02647800	1.88645500
H	14.70055700	0.50723400	1.69911500
H	15.46873000	-1.34497800	0.22360900
H	13.79557800	-2.66284100	-1.06473700
H	11.39106500	-2.11702000	-0.89935100
H	10.67665700	-1.87997300	2.69490900
H	9.91975300	-1.67777400	5.04005000
H	8.57573800	0.29259500	5.75365400
H	7.99686700	2.05172700	4.08996300
H	8.72570800	1.82677700	1.74016800
H	-5.21034100	-1.70208100	1.23379300
H	-7.66467600	-1.75512200	1.23026100
H	-7.73765400	1.58413500	-1.46912100
H	-5.28271600	1.65258100	-1.45743700
H	-11.40405200	1.49325400	-1.01934900
H	-12.31777500	1.69088700	-3.30723300
H	-11.41861900	0.26714300	-5.14039700
H	-9.57549600	-1.33576600	-4.65881900
H	-8.64261300	-1.50442600	-2.37659100
H	-12.19274000	0.96661900	2.29025800
H	-14.60379000	0.42495900	2.22970500
H	-15.42339100	-1.47496600	0.84546700
H	-13.80042200	-2.81812700	-0.48038900
H	-11.39521700	-2.25071700	-0.44208500
H	-10.54848400	-1.88284300	3.11919100
H	-9.68409800	-1.60292400	5.41773200
H	-8.28606300	0.37420400	5.99552900
H	-7.76034700	2.06107600	4.24171400
H	-8.59750900	1.75665400	1.93653300
H	6.22530300	-3.99128500	0.92337000

H	6.43946000	-8.00668800	1.60871500
H	-7.36636200	-8.03735000	-1.04757300
H	-5.90537900	-7.09917200	2.09845600
H	-6.62766200	-3.99053500	-0.47226500
H	7.69942800	-5.88812700	-1.72568900
C	7.88641600	-8.40304400	0.07546000
H	8.63902600	-7.69029900	0.43069500
H	8.15883000	-9.39085500	0.47130600
H	7.97564300	-8.45808900	-1.01627000
C	5.45067400	-9.09887300	0.05793200
H	5.73971200	-10.08380800	0.44993900
H	4.43675400	-8.88087800	0.40826000
H	5.40890800	-9.18457000	-1.03458000
C	6.06017600	-6.70998000	-2.84868100
H	6.44394400	-6.41997800	-3.83644100
H	6.34487200	-7.75465100	-2.68090600
H	4.96539700	-6.66791500	-2.89705700
C	6.29677500	-4.30283000	-2.11271500
H	6.74657900	-3.59452600	-1.40803900
H	6.67940100	-4.06034100	-3.11391700
H	5.21597100	-4.11611900	-2.12300700
C	8.13900400	-4.85655400	1.36764000
H	8.59714900	-4.65758500	0.39147600
H	8.44178400	-4.04470100	2.04353700
H	8.58310700	-5.77947600	1.76087400
C	5.99845700	-5.18855800	2.68607900
H	6.30093100	-4.39118100	3.37854900
H	4.90366200	-5.20948000	2.65731800
H	6.33878100	-6.13713100	3.12111500
C	-7.94803100	-6.51072400	1.79970600
H	-8.20963400	-6.58559000	2.86402400
H	-8.54299900	-5.68912700	1.38204200
H	-8.27754900	-7.43795600	1.31596200
C	-6.00833200	-4.97719800	2.36221100
H	-6.51499200	-4.09238600	1.95550800
H	-6.26711100	-5.03279800	3.42831700
H	-4.92854300	-4.80732100	2.28839300
C	-5.65358900	-4.33001000	-2.34343700
H	-6.07146700	-3.42817200	-2.81146300
H	-4.64641400	-4.08912000	-1.98919200
H	-5.55216000	-5.08689900	-3.13065700
C	-7.99370500	-5.07912100	-1.71780900
H	-8.68555200	-5.37355200	-0.92047200
H	-8.40670200	-4.17746800	-2.19072500
H	-8.00687300	-5.87346400	-2.47422300
C	-5.71959500	-8.13117800	-2.42575300
H	-5.97789000	-9.11285700	-2.84620500
H	-6.12250100	-7.36905300	-3.10174700
H	-4.62653600	-8.04328500	-2.44311600
C	-5.79068800	-9.16710800	-0.11943200
H	-6.04746300	-10.12731200	-0.58740700
H	-4.70151000	-9.14837800	0.01126000
H	-6.24408900	-9.15710500	0.87813500
C	-8.28486500	4.97854700	0.66766500
H	-8.68977900	4.90036100	-0.34845100
H	-8.67105900	4.12394300	1.23995300

H	-8.70641300	5.88247200	1.12466100
C	-6.21773700	5.05445100	2.13519000
H	-6.54027900	5.96915500	2.64916100
H	-6.59865600	4.20564100	2.71994100
H	-5.12368100	5.01913800	2.17156300
C	-5.37539800	9.19336600	0.00713500
H	-5.66978300	10.14328600	0.47431700
H	-4.40662300	8.89904300	0.42306500
H	-5.22777100	9.39006700	-1.06172600
C	-7.82295400	8.60364100	-0.26655300
H	-7.80782600	8.78273900	-1.34847400
H	-8.62790300	7.88851100	-0.06352700
H	-8.09944800	9.55230000	0.21327700
C	-5.82447100	7.13107200	-3.19645200
H	-6.15082000	6.96863500	-4.23281200
H	-6.05825000	8.17009700	-2.93869700
H	-4.73359000	7.02153600	-3.17432100
C	-6.25434600	4.68326600	-2.73030200
H	-6.80794600	3.94661000	-2.13692800
H	-6.56683300	4.56372500	-3.77653100
H	-5.18933800	4.42531400	-2.67505300
C	5.53471600	9.42900800	-0.43895300
H	4.46082900	9.29605400	-0.61986800
H	5.69425000	10.48264100	-0.17211000
H	6.05401200	9.24909000	-1.38699300
C	5.37069200	8.88305600	2.02556400
H	5.52746900	9.94809500	2.24464500
H	4.28786600	8.70917400	2.00085800
H	5.77814100	8.31321500	2.86812300
C	7.91526300	5.63271000	1.94948700
H	8.18715000	5.11956100	2.88211900
H	8.47213700	5.14439900	1.14072200
H	8.28069700	6.66389800	2.02665200
C	5.91334300	4.09769600	1.65721000
H	6.37864700	3.54254500	0.83491300
H	6.17122500	3.57154000	2.58709700
H	4.82768400	4.03157500	1.52905800
C	8.04196500	6.72771900	-1.59304100
H	8.69783600	6.11543900	-0.96325000
H	8.39130000	6.61314500	-2.62820000
H	8.19694100	7.77539700	-1.31025600
C	6.37455300	4.84979100	-1.95680900
H	6.96522700	4.14414200	-1.36096400
H	5.32539900	4.53826000	-1.90138100
H	6.70042600	4.74482900	-3.00080000

2, CAM-B3LYP/6-31G(d), E = –5715.499194 Hartree.

0 1

C	1.58692700	1.07270100	0.13761700
C	2.97228000	0.89517800	0.21929300
C	3.54557100	-0.38200200	0.21181100
C	2.70256300	-1.49283400	0.08238200
C	1.31187600	-1.34884700	0.04249800

C	0.72965300	-0.05512800	0.08058200
C	-0.71054500	0.11006300	0.06907200
C	-1.56709100	-1.01937000	0.02327800
C	-2.95532000	-0.84427700	0.01374400
C	-3.53036600	0.42998100	0.08179500
C	-2.68413400	1.54372700	0.16043600
C	-1.29249100	1.40430600	0.12466900
C	0.44290200	-2.52593400	-0.01613400
C	-0.98609400	-2.36362200	-0.00485900
C	-0.42145400	2.58313000	0.12347600
C	1.00788900	2.41685900	0.10461100
N	0.99447300	-3.73180600	-0.06266900
C	0.18232100	-4.80772100	-0.08811500
C	-1.24220000	-4.64751900	-0.04505400
N	-1.79364000	-3.41713200	-0.00839700
N	-0.96835300	3.79257500	0.11658700
C	-0.15176500	4.86591300	0.07334600
C	1.27152500	4.69812200	0.01544300
N	1.81851300	3.46571900	0.04098600
C	-0.71751900	6.18874600	0.06283400
C	0.14739300	7.27687400	0.00301300
C	1.54000300	7.11139800	-0.07453800
C	2.12728900	5.85048900	-0.08494300
C	0.75643800	-6.12547800	-0.14602000
C	-0.10304700	-7.21906000	-0.15542400
C	-1.49825800	-7.06317200	-0.09697100
C	-2.09358500	-5.80742200	-0.03486000
C	3.53597000	5.72673400	-0.23833700
C	4.74468400	5.70941000	-0.42027500
C	-2.12269200	6.40548300	0.08540200
C	-3.31183500	6.68782400	0.07984400
Si	-5.10169700	7.13056100	0.02851200
C	-5.68113600	7.56297800	1.80988600
C	-5.30472500	8.63930600	-1.15071100
C	-6.05557200	5.55839300	-0.51763300
Si	6.55079200	5.73296800	-0.79923800
C	7.45814300	6.09938400	0.85091200
C	7.04226100	4.01807800	-1.51020700
C	6.85144700	7.16454600	-2.03880800
C	2.16379200	-6.32615500	-0.18412100
C	-3.50816400	-5.69554200	0.06049600
C	-4.72547400	-5.68568000	0.16935600
C	3.35689600	-6.58703000	-0.21325000
Si	5.14355300	-7.03194700	-0.24643900
C	6.06223800	-5.96439500	1.01284400
C	5.30533100	-8.86056900	0.20966800
Si	-6.56134100	-5.70854000	0.37651100
C	-7.01968200	-4.27988900	1.57740100
C	-7.27144300	-5.35703500	-1.37242600
C	5.81771400	-6.73445300	-1.98628400
C	-5.02069400	0.58484600	0.13068400
C	-5.54118700	1.20876400	1.38314200
C	-5.81215900	0.15155200	-0.89350700
C	-5.24176500	-0.26278500	-2.21305500
C	-7.29951900	0.04068800	-0.79368900
C	-4.36796800	0.57514000	-2.92592600

C	-3.86559100	0.19300800	-4.16954700
C	-4.22490700	-1.03567100	-4.72574600
C	-5.10252600	-1.87378300	-4.03440200
C	-5.61459300	-1.48538200	-2.79798200
C	-7.91649600	-0.58655100	0.30252300
C	-9.30310300	-0.72077400	0.36425600
C	-10.10481200	-0.22711300	-0.66747000
C	-9.50621600	0.39292200	-1.76591500
C	-8.11878300	0.51500300	-1.83320800
C	-4.99722600	0.85243100	2.63052300
C	-5.46699100	1.42717400	3.81081400
C	-6.48043300	2.38590500	3.77083500
C	-7.01757700	2.76525600	2.53815800
C	-6.55219100	2.18599200	1.35892900
C	5.03126900	-0.55077500	0.29788300
C	5.73460300	-0.07968000	1.36892700
C	7.22596300	0.01021500	1.39730300
C	5.05471800	0.39169800	2.61585600
C	5.37773900	1.63859200	3.17812500
C	4.76475000	2.07514500	4.35111900
C	3.83311000	1.26310300	5.00166600
C	3.52008500	0.01186900	4.46805200
C	4.12387400	-0.41883400	3.28703500
C	7.94616500	0.57575000	0.33071600
C	9.33442100	0.69196300	0.38533200
C	10.03501000	0.24150200	1.50628800
C	9.33311900	-0.31627000	2.57666300
C	7.94357900	-0.42000100	2.52726700
C	5.65039800	-1.25407800	-0.86365600
C	5.24246600	-0.95205600	-2.17562500
C	5.80094700	-1.60955000	-3.27096000
C	6.76706700	-2.59880600	-3.07898500
C	7.16725300	-2.92492400	-1.78073900
C	6.61438300	-2.26208600	-0.68619700
H	3.59993500	1.77596600	0.29061000
H	3.11441900	-2.49562200	0.04183700
H	-3.58318200	-1.72650000	-0.03709400
H	-3.09714200	2.54457200	0.22863700
H	-0.27130600	8.27796000	-0.00199500
H	2.17753600	7.98672500	-0.14456000
H	0.32025700	-8.21723400	-0.20008500
H	-2.13264300	-7.94359300	-0.09290600
H	-6.78219200	7.55235500	1.75594000
H	-6.24891900	9.11974100	-0.84666300
H	-5.94363700	4.88763600	0.34748200
H	7.01074500	7.04104000	1.20393200
H	7.21428400	3.39209100	-0.62144800
H	7.92511200	7.13109600	-2.28211900
H	5.96981000	-4.90109900	0.76635600
H	4.90330700	-9.05955300	1.20958700
H	-7.06829200	-3.39680300	0.92097900
H	-6.67528200	-4.49662700	-1.71580900
H	6.89591600	-6.93630500	-2.02192600
H	-4.08932800	1.53549700	-2.50357300
H	-3.19547200	0.85993700	-4.70574600
H	-3.83018800	-1.33519200	-5.69296700

H	-5.39100500	-2.83161000	-4.45930000
H	-6.30839200	-2.13594800	-2.27295100
H	-7.30070500	-0.96745600	1.11088000
H	-9.75694200	-1.21294700	1.22048200
H	-11.18553400	-0.33008000	-0.61821300
H	-10.11974800	0.77779400	-2.57640500
H	-7.65992900	0.98496400	-2.69843300
H	-4.20490700	0.11091400	2.67343900
H	-5.03726500	1.12637400	4.76271800
H	-6.84418600	2.83832700	4.68940600
H	-7.80175500	3.51650800	2.49305800
H	-6.97656100	2.48810800	0.40732400
H	6.10846000	2.27195300	2.68414600
H	5.01777200	3.05033400	4.75884400
H	3.35992500	1.60046600	5.92001800
H	2.80674200	-0.63420300	4.97319200
H	3.87929900	-1.39550700	2.88104700
H	7.40901700	0.92469200	-0.54521500
H	9.86876200	1.13784900	-0.44960800
H	11.11732400	0.33053900	1.54794600
H	9.86743500	-0.66688600	3.45589500
H	7.40381700	-0.84220400	3.37004000
H	4.48578200	-0.18942100	-2.33517600
H	5.47703300	-1.35053100	-4.27556300
H	7.20010300	-3.11510900	-3.93152000
H	7.91075600	-3.70067600	-1.61810600
H	6.93206000	-2.52270200	0.31819400
C	6.55509400	8.54157100	-1.41371300
H	6.74802600	9.34639200	-2.13619600
H	5.50384800	8.62057000	-1.10988400
H	7.17121700	8.74012100	-0.52949000
C	6.06719900	6.99490400	-3.35416500
H	6.33281100	6.07144100	-3.88032000
H	4.98618200	6.97159800	-3.17126900
H	6.26800400	7.83153600	-4.03750900
C	8.97258900	6.33574600	0.69144200
H	9.20030300	7.11873400	-0.04150700
H	9.42079500	6.64098000	1.64684100
H	9.49069300	5.42245900	0.37392900
C	7.18449200	5.03240100	1.92727200
H	6.11083200	4.87401600	2.07932300
H	7.63212400	4.06730400	1.65731800
H	7.61974700	5.32960900	2.89127000
C	8.36273800	4.05486500	-2.30690300
H	9.19363400	4.47379400	-1.72800600
H	8.65633200	3.04220000	-2.61613400
H	8.26484100	4.65255800	-3.22141300
C	5.93141000	3.33684300	-2.33372300
H	6.23186700	2.31933800	-2.62121800
H	4.99227400	3.26763400	-1.77569500
H	5.72149600	3.88433000	-3.26057600
C	-4.17466900	9.67686100	-0.98774400
H	-3.21134300	9.25269100	-1.29363600
H	-4.36578300	10.55476800	-1.62016100
H	-4.06510100	10.02920800	0.04207800
C	-5.43406900	8.26118500	-2.63942400

H	-4.53995900	7.73650200	-2.99788600
H	-6.29826700	7.61952700	-2.83752000
H	-5.54797000	9.16389700	-3.25545700
C	-5.26346200	8.95958900	2.30540400
H	-5.65286400	9.76133900	1.66867100
H	-5.64160300	9.13617100	3.32181400
H	-4.17179700	9.06162500	2.34511800
C	-5.25260800	6.48976000	2.83022900
H	-5.65411000	6.72231100	3.82630100
H	-5.60232700	5.48682800	2.56294800
H	-4.16075800	6.44324700	2.91699700
C	-7.56622800	5.79782900	-0.71197400
H	-8.08808300	4.85114400	-0.90904200
H	-8.03436800	6.24892200	0.17128900
H	-7.76963400	6.45725200	-1.56450100
C	-5.44741100	4.81968400	-1.72511600
H	-4.38003900	4.62113600	-1.57939300
H	-5.95056100	3.85486500	-1.87995100
H	-5.55532400	5.39154200	-2.65333100
C	-7.00081300	-6.51529700	-2.35163100
H	-7.56915500	-7.41409600	-2.08142500
H	-7.29854600	-6.24095200	-3.37293100
H	-5.93945000	-6.78737800	-2.37765400
C	-8.75094800	-4.92850700	-1.41337600
H	-9.05605100	-4.70204500	-2.44430100
H	-9.41929700	-5.71765000	-1.04816800
H	-8.93734100	-4.02873400	-0.81680800
C	-8.40371600	-4.43386400	2.24000600
H	-8.66072600	-3.52919400	2.80847200
H	-9.20618700	-4.60376700	1.51394700
H	-8.41603800	-5.26984300	2.94960600
C	-5.94571900	-3.99398400	2.64563100
H	-4.96227100	-3.81691600	2.19979700
H	-6.21632200	-3.10703100	3.23558800
H	-5.84351600	-4.83041900	3.34786700
C	-6.97061400	-7.47949200	0.99650200
H	-6.47091500	-8.12689500	0.25884800
C	-8.46581600	-7.85352800	0.98936000
H	-8.60011800	-8.90234300	1.28824700
H	-9.04459500	-7.24268300	1.69124800
H	-8.91827000	-7.73753200	-0.00154200
C	-6.33755600	-7.78529100	2.36820200
H	-6.80442700	-7.19679800	3.16764300
H	-6.46825000	-8.84446600	2.62964100
H	-5.26360100	-7.56892900	2.37999900
C	5.50732100	-6.17175800	2.43434600
H	6.16222700	-5.71043600	3.14369800
H	4.53559500	-5.72957500	2.50591200
H	5.43858300	-7.21934100	2.64109400
C	7.56132600	-6.31617300	1.03709400
H	7.97126300	-6.19829200	0.05579100
H	8.06966200	-5.66447500	1.71664200
H	7.68462700	-7.33016800	1.35570000
C	6.78286500	-9.29467300	0.20287500
H	7.21601400	-9.10068400	1.16185900
H	6.84622300	-10.34094900	-0.01205100

H	7.31295700	-8.74400600	-0.54590300
C	4.54871900	-9.75703300	-0.78807400
H	4.94358900	-10.75058800	-0.74532100
H	3.50941400	-9.77505400	-0.53426200
H	4.66745600	-9.36832600	-1.77787600
C	5.59451300	-5.27662700	-2.42959300
H	5.34995300	-4.67805600	-1.57706600
H	6.48738900	-4.90148500	-2.88450100
H	4.79111600	-5.23743500	-3.13522600
C	5.13406000	-7.65392300	-3.01526100
H	5.08764700	-7.15737000	-3.96193000
H	5.69658600	-8.55871900	-3.11429700
H	4.14294300	-7.88453000	-2.68449500

3, CAM-B3LYP/6-31G(d), E = –5234.554861 Hartree.

C	-1.44852900	-1.21239200	-0.31291500
C	-2.84576600	-1.19809900	-0.30812800
C	-3.56630100	0.00342800	-0.27410600
C	-2.84869100	1.20741200	-0.24864100
C	-1.45045200	1.22435900	-0.25639900
C	-0.72436100	0.00650600	-0.28624000
C	0.72432000	0.00647200	-0.28624100
C	1.45046600	1.22428900	-0.25638800
C	2.84870000	1.20728500	-0.24859000
C	3.56627300	0.00327800	-0.27400500
C	2.84568100	-1.19821800	-0.30808900
C	1.44844100	-1.21245600	-0.31293800
C	-0.71918400	2.49321200	-0.24582900
C	0.71925600	2.49317600	-0.24583500
C	0.71855000	-2.48124900	-0.33086300
C	-0.71869400	-2.48121600	-0.33078900
N	-1.40278600	3.63027100	-0.23840800
C	-0.71662800	4.79087400	-0.23336400
C	0.71681900	4.79083700	-0.23339000
N	1.40291700	3.63019800	-0.23844100
N	1.40252400	-3.61788600	-0.34501500
C	0.71633200	-4.77812700	-0.35657400
C	-0.71660000	-4.77809100	-0.35639000
N	-1.40272900	-3.61781700	-0.34476300
C	1.43533800	-6.02432900	-0.36623700
C	0.70316100	-7.20675200	-0.37902100
C	-0.70355900	-7.20671500	-0.37878500
C	-1.43567300	-6.02425700	-0.36578300
C	-1.43513100	6.03721600	-0.23161300
C	-0.70282800	7.21975500	-0.22415900
C	0.70316300	7.21971700	-0.22419100
C	1.43539600	6.03713500	-0.23167700
C	-2.85764000	-6.04666700	-0.35578100
C	-4.07754700	-6.11034900	-0.34240000
C	2.85730700	-6.04681000	-0.35681300
C	4.07721600	-6.11055000	-0.34398100
Si	5.91623800	-6.20246000	-0.31658700

C	6.58059200	-4.85497700	0.83026000
C	6.42281000	-7.90873600	0.32117000
C	6.56287100	-5.94569700	-2.07442000
Si	-5.91655500	-6.20219400	-0.31403300
C	-6.42282700	-7.90797600	0.32527800
C	-6.58031800	-4.85384600	0.83214700
C	-6.56409200	-5.94676500	-2.07173100
C	-5.04951500	-0.00642100	-0.26039500
C	-5.77653000	0.91790200	0.50778700
C	-7.16784400	0.89521900	0.53372400
C	-7.89466100	-0.04836600	-0.21187500
C	-7.16624100	-0.95430200	-1.00281100
C	-5.77471500	-0.94337000	-1.01622500
C	-9.38819700	-0.05777000	-0.21946900
C	-10.00451500	-0.11954100	-1.58054100
C	-10.13077700	-0.01727600	0.92732900
C	-9.53308300	-0.22629000	2.28173800
C	-11.60388500	0.23656100	0.92697200
C	-11.00271000	-1.05979300	-1.88747900
C	-11.54564800	-1.13531900	-3.16920500
C	-11.10334300	-0.27153900	-4.17308300
C	-10.10480900	0.66162900	-3.88638900
C	-9.55413200	0.72877200	-2.60752500
C	-12.46673300	-0.56918500	1.69101400
C	-13.83912100	-0.32652500	1.71692600
C	-14.37728200	0.74251900	0.99745300
C	-13.53030300	1.56496900	0.25198300
C	-12.15948900	1.31436400	0.21658300
C	-9.83522700	0.65077800	3.33852800
C	-9.29975700	0.45215100	4.61006500
C	-8.46846900	-0.64203000	4.85936100
C	-8.17811900	-1.53443600	3.82566200
C	-8.70406800	-1.32858800	2.55110500
C	5.04947800	-0.00662300	-0.26015700
C	5.77646900	0.91784800	0.50787400
C	7.16777800	0.89515500	0.53391200
C	7.89464700	-0.04861900	-0.21139700
C	7.16625300	-0.95469500	-1.00221000
C	5.77473100	-0.94372800	-1.01574700
C	9.38817300	-0.05802400	-0.21896100
C	10.00451100	-0.12040400	-1.57999900
C	10.13077600	-0.01702600	0.92781200
C	11.00274400	-1.06076000	-1.88648200
C	11.54571000	-1.13685700	-3.16816500
C	11.10338700	-0.27356000	-4.17244900
C	10.10481300	0.65970400	-3.88620500
C	9.55411100	0.72741800	-2.60738200
C	9.53313800	-0.22553200	2.28232800
C	11.60388000	0.23681700	0.92732300
C	12.46674300	-0.56863000	1.69166700
C	13.83912900	-0.32594900	1.71747600
C	14.37727500	0.74282400	0.99758700
C	13.53028300	1.56498000	0.25180900
C	12.15947100	1.31435100	0.21651500
C	9.83523900	0.65200000	3.33874500
C	9.29985000	0.45384000	4.61038900

C	8.46870400	-0.64033400	4.86017700
C	8.17842200	-1.53321000	3.82686500
C	8.70428400	-1.32783000	2.55219800
C	-2.85688700	6.07025700	-0.24566800
C	2.85715300	6.07008100	-0.24579400
C	4.07492900	6.16569700	-0.26491500
C	-4.07465900	6.16594000	-0.26475900
Si	-5.90761400	6.33657400	-0.31159400
C	-6.68137000	4.97269700	0.74213200
C	-6.36793400	8.03546800	0.37887600
Si	5.90789300	6.33623700	-0.31177600
C	6.36831200	8.03506500	0.37879500
C	6.68159300	4.97223800	0.74183900
C	6.48374300	6.18150900	-2.10562800
C	-6.48349500	6.18176300	-2.10543200
H	-3.36129100	-2.15198100	-0.30435300
H	-3.36660600	2.16015300	-0.24584100
H	3.36663800	2.16001300	-0.24578700
H	3.36115100	-2.15213000	-0.30429400
H	1.23501700	-8.15263600	-0.38651900
H	-1.23546600	-8.15257300	-0.38608400
H	-1.23503700	8.16543900	-0.22211900
H	1.23542400	8.16537200	-0.22217800
H	7.67494900	-4.91018700	0.89287900
H	6.31473800	-3.85411300	0.47149400
H	6.18067500	-4.96480500	1.84465700
H	7.51540400	-8.00462900	0.34851000
H	6.04665800	-8.08257200	1.33571000
H	6.03340200	-8.70684800	-0.32106100
H	7.65895300	-5.99044200	-2.09518400
H	6.18332400	-6.71574000	-2.75547900
H	6.25989500	-4.97058200	-2.47217500
H	-7.51540400	-8.00392200	0.35304400
H	-6.03356800	-8.70658700	-0.31642500
H	-6.04633500	-8.08096000	1.33983700
H	-7.67468100	-4.90872000	0.89498600
H	-6.18025300	-4.96322000	1.84653500
H	-6.31424400	-3.85326600	0.47276100
H	-7.66018200	-5.99158600	-2.09189800
H	-6.26137000	-4.97193500	-2.47037100
H	-6.18485800	-6.71730200	-2.75240700
H	-5.24573700	1.64819900	1.11216500
H	-7.70172900	1.61477800	1.14609500
H	-7.70057400	-1.67424700	-1.61610100
H	-5.24203300	-1.65124200	-1.64538900
H	-11.35098000	-1.73502600	-1.11212100
H	-12.31344500	-1.87394100	-3.38457900
H	-11.52771500	-0.32985800	-5.17187000
H	-9.74955200	1.33598000	-4.66134900
H	-8.76867100	1.44884600	-2.39545500
H	-12.05360600	-1.39454800	2.26403900
H	-14.48845300	-0.97060800	2.30437500
H	-15.44616900	0.93722600	1.02346300
H	-13.93666700	2.40763800	-0.30141400
H	-11.50715800	1.95874200	-0.36442100
H	-10.49134900	1.49706000	3.15506800

H	-9.53587900	1.15083800	5.40858300
H	-8.05618400	-0.80130400	5.85218500
H	-7.54235800	-2.39633400	4.01061400
H	-8.47423200	-2.02855900	1.75372900
H	5.24566500	1.64831100	1.11204400
H	7.70161700	1.61487500	1.14613300
H	7.70061100	-1.67476300	-1.61533200
H	5.24210100	-1.65170100	-1.64484200
H	11.35102700	-1.73562100	-1.11080400
H	12.31353600	-1.87555200	-3.38318400
H	11.52777600	-0.33232600	-5.17120200
H	9.74954900	1.33368300	-4.66148500
H	8.76862500	1.44756400	-2.39565500
H	12.05362400	-1.39377400	2.26501200
H	14.48847300	-0.96979800	2.30516800
H	15.44616100	0.93754700	1.02351400
H	13.93663700	2.40743800	-0.30191700
H	11.50713500	1.95850500	-0.36473000
H	10.49125900	1.49827900	3.15490400
H	9.53592900	1.15288800	5.40860500
H	8.05648800	-0.79924900	5.85308700
H	7.54279000	-2.39511500	4.01222200
H	8.47452200	-2.02817100	1.75512400
H	-7.77385100	5.07328800	0.76267900
H	-6.44463300	3.98026600	0.34256100
H	-6.32175400	5.01486000	1.77648800
H	-7.45560000	8.17866200	0.36473800
H	-6.02934100	8.14961200	1.41489300
H	-5.91718100	8.84171700	-0.21091800
H	7.45596000	8.17835200	0.36431400
H	5.91729200	8.84137000	-0.21071800
H	6.03005700	8.14901400	1.41494500
H	7.77409100	5.07266800	0.76223900
H	6.32212000	5.01443300	1.77624300
H	6.44466300	3.97984700	0.34227900
H	7.57465500	6.27821400	-2.17261300
H	6.20621000	5.20957600	-2.52874800
H	6.03905200	6.95961600	-2.73620000
H	-7.57440900	6.27844400	-2.17240800
H	-6.03882600	6.95984700	-2.73604700
H	-6.20595100	5.20981400	-2.52850900

4, CAM-B3LYP/6-31G(d), E = –4772.709189 Hartree.

C	1.58258100	1.03808100	-0.08649400
C	2.96943300	0.85546100	-0.11374900
C	3.53750400	-0.42472600	-0.09948800
C	2.68282900	-1.53464500	-0.06892200
C	1.29399700	-1.38323300	-0.00753300
C	0.71929200	-0.08581100	-0.02200700
C	-0.71927700	0.08578700	0.02211100
C	-1.58256600	-1.03810300	0.08663700
C	-2.96942100	-0.85548900	0.11390400
C	-3.53749700	0.42469100	0.09957800
C	-2.68282000	1.53461700	0.06894400

C	-1.29398500	1.38320700	0.00754500
C	0.41996900	-2.55506700	0.08189500
C	-1.00744000	-2.38422600	0.13451300
C	-0.41996100	2.55503800	-0.08188600
C	1.00745200	2.38421000	-0.13436900
N	0.96637400	-3.76328300	0.12642100
C	0.15084300	-4.83258900	0.22439600
C	-1.27196700	-4.66243400	0.28260600
N	-1.81823200	-3.43020900	0.23488500
N	-0.96637500	3.76323600	-0.12653700
C	-0.15085300	4.83254600	-0.22453800
C	1.27197200	4.66242200	-0.28251800
N	1.81825100	3.43020700	-0.23464600
C	-0.72024800	6.15239000	-0.28019600
C	0.14210600	7.23881500	-0.38361400
C	1.53593200	7.07248800	-0.44262900
C	2.12664800	5.81357400	-0.39967700
C	0.72021100	-6.15245500	0.27983900
C	-0.14215500	-7.23886700	0.38329900
C	-1.53596400	-7.07250100	0.44265900
C	-2.12664700	-5.81356600	0.39993500
C	3.54163200	5.69539500	-0.48320000
C	4.76013200	5.69684000	-0.57226400
C	-2.12640900	6.36106400	-0.24288000
C	-3.31869300	6.62654000	-0.22576600
Si	-5.10561700	7.07024900	-0.21703800
C	-5.72238800	7.08618500	1.56904800
C	-5.29321200	8.78871600	-0.98498300
C	-6.05647800	5.79520800	-1.23641700
Si	6.59264000	5.76990300	-0.72884400
C	7.35347100	5.77335900	1.00207300
C	7.19409100	4.27381800	-1.71155900
C	7.04656300	7.36640300	-1.63559500
C	2.12636600	-6.36116700	0.24220000
C	-3.54159100	-5.69531300	0.48386900
C	-4.76005900	-5.69670900	0.57332200
C	3.31863200	-6.62671500	0.22472200
Si	5.10554900	-7.07044100	0.21541000
C	6.05668600	-5.79584900	1.23508600
C	5.29329400	-8.78923000	0.98260300
Si	-6.59251900	-5.76948100	0.73059800
C	-7.04632900	-7.36553400	1.63819400
C	-7.19340100	-4.27287600	1.71286500
C	-7.35398700	-5.77359500	-1.00004000
C	5.72187200	-7.08573400	-1.57084100
C	-5.02135200	0.60626300	0.17846800
C	-5.48421900	1.45663600	1.31556600
C	-5.86640000	0.02438300	-0.72286500
C	-5.37266300	-0.60619500	-1.98555300
C	-7.34743900	-0.03433200	-0.52987600
C	-4.50454800	0.07924300	-2.85227000
C	-4.07541900	-0.50349800	-4.04409400
C	-4.50408900	-1.78483100	-4.39484700
C	-5.37680000	-2.47365600	-3.54980500
C	-5.81543300	-1.88525200	-2.36527100
C	-7.91061500	-0.46619200	0.68339400

C	-9.29316100	-0.55185700	0.84078200
C	-10.14419500	-0.20413900	-0.21016400
C	-9.59962500	0.21870400	-1.42449900
C	-8.21678700	0.29155800	-1.58589900
C	-4.92260700	1.29930300	2.59545000
C	-5.33404300	2.09517100	3.66348700
C	-6.30450700	3.08039400	3.47272000
C	-6.85783800	3.26222000	2.20295200
C	-6.45123000	2.46138600	1.13685100
C	5.02134500	-0.60626000	-0.17848700
C	5.86642400	-0.02446200	0.72286400
C	7.34743500	0.03439500	0.52975700
C	5.37271600	0.60590600	1.98565600
C	5.81547900	1.88490800	2.36557100
C	5.37686700	2.47312400	3.55020300
C	4.50418100	1.78415800	4.39515900
C	4.07552300	0.50287500	4.04421600
C	4.50462900	-0.07967300	2.85228900
C	7.91047000	0.46651700	-0.68348700
C	9.29299400	0.55229500	-0.84099300
C	10.14415300	0.20442400	0.20980300
C	9.59972700	-0.21867800	1.42411100
C	8.21690600	-0.29167000	1.58562400
C	5.48417600	-1.45654000	-1.31566400
C	4.92243600	-1.29921100	-2.59548900
C	5.33382900	-2.09502200	-3.66358600
C	6.30434800	-3.08021300	-3.47292700
C	6.85777700	-3.26206600	-2.20320300
C	6.45123900	-2.46126400	-1.13705500
H	3.60097300	1.73543300	-0.15495700
H	3.08704800	-2.54124200	-0.07485900
H	-3.60095300	-1.73546900	0.15506300
H	-3.08704100	2.54121400	0.07487300
H	-0.27769800	8.23858800	-0.42537200
H	2.17340600	7.94633000	-0.52960700
H	0.27762600	-8.23865700	0.42486100
H	-2.17344100	-7.94633100	0.52974600
H	-6.78100600	7.37283300	1.60992300
H	-5.62239000	6.09799900	2.03117400
H	-5.15878900	7.80216000	2.17794200
H	-6.34708500	9.09366600	-1.00082800
H	-4.73607800	9.54285700	-0.41749800
H	-4.92460700	8.80692600	-2.01689000
H	-7.12724200	6.03397600	-1.25884200
H	-5.69823800	5.76545500	-2.27160700
H	-5.94365400	4.79167300	-0.81204300
H	8.44866000	5.80450300	0.94577500
H	7.02224300	6.64352600	1.58012500
H	7.07272700	4.87438300	1.56213200
H	8.28084900	4.31785600	-1.85573200
H	6.96702800	3.33581300	-1.19348100
H	6.72507900	4.23418900	-2.70124600
H	8.13477200	7.45418600	-1.74484800
H	6.60659500	7.39365300	-2.63892500
H	6.69309300	8.24957400	-1.09141000
H	7.12744900	-6.03465700	1.25714400

H	5.94378300	-4.79212700	0.81118100
H	5.69870500	-5.76654300	2.27038100
H	6.34713800	-9.09430500	0.99785300
H	4.92514900	-8.80781300	2.01466800
H	4.73582100	-9.54307900	0.41506300
H	-8.13450600	-7.45309100	1.74795100
H	-6.69324700	-8.24897900	1.09419900
H	-6.60592700	-7.39247000	2.64134000
H	-8.28016100	-4.31651400	1.85714200
H	-6.72428900	-4.23305800	2.70249500
H	-6.96607500	-3.33512800	1.19444200
H	-8.44915700	-5.80466000	-0.94331800
H	-7.07341200	-4.87486800	-1.56058800
H	-7.02302400	-6.64402600	-1.57784700
H	6.78050700	-7.37227200	-1.61208100
H	5.15819000	-7.80158400	-2.17980900
H	5.62166500	-6.09742100	-2.03264900
H	-4.17106000	1.07831200	-2.58956500
H	-3.40811900	0.04780600	-4.70149300
H	-4.16646700	-2.24051300	-5.32186200
H	-5.71854500	-3.47100300	-3.81400200
H	-6.50609500	-2.42076800	-1.72001000
H	-7.25601800	-0.73563400	1.50614800
H	-9.70517900	-0.89444600	1.78638200
H	-11.22197800	-0.26888200	-0.08642800
H	-10.25244600	0.48791000	-2.25085800
H	-7.80043200	0.60891800	-2.53769900
H	-4.16221900	0.53977100	2.75289900
H	-4.89286700	1.94665600	4.64562500
H	-6.62317500	3.70419800	4.30352800
H	-7.60768800	4.03177100	2.04003800
H	-6.88792100	2.60971400	0.15454200
H	6.50612200	2.42052800	1.72038300
H	5.71860300	3.47043800	3.81453900
H	4.16656800	2.23969400	5.32224800
H	3.40825100	-0.04854500	4.70154500
H	4.17114000	-1.07870200	2.58944100
H	7.25577900	0.73606100	-1.50613400
H	9.70489800	0.89508900	-1.78656900
H	11.22191900	0.26925900	0.08596800
H	10.25263700	-0.48800900	2.25035800
H	7.80067100	-0.60925100	2.53740100
H	4.16199600	-0.53971100	-2.75285900
H	4.89257400	-1.94648400	-4.64568400
H	6.62297800	-3.70397400	-4.30378000
H	7.60764800	-4.03161100	-2.04036100
H	6.88800100	-2.60961700	-0.15478400

9. References and Notes

1 N. Keller, A. D. Bromby and T. C. Sutherland, *Eur. J. Org. Chem.*, **2017**, *2017*, 3980–3985.

2 N. Grabicki, K. T. D. Nguyen, S. Weidner and O. Dumele, *Angew. Chem. Int. Ed.*, **2021**, *60*, 14909−14914.

3 L. Ueberricke, D. Mizioch, F. Ghalami, F. Mildner, F. Rominger, T. Oeser, M. Elstner, M. Mastalerz, *Eur. J. Org. Chem.*, **2021**, 4816−4823.

4 J. J. Bryant, Y. Zhang, B. D. Lindner, E. A. Davey, A. L. Appleton, X. Qian and U. H. F. Bunz, *J. Org. Chem.*, **2012**, *77*, 7479−7486.

5 M. Ganschow, S. S. Koser, M. Hodecker, F. Rominger, J. Freudenberg, A. Dreuw and U. H. F. Bunz, *Chem. Eur. J.*, **2018**, *24*, 13667−13675.

6 G. M. Sheldrick, Crystal structure refinement with SHELXL, *Acta Cryst.* **2015**, *C71*, 3.

7 G. M. Sheldrick, SHELXT - Integrated space-group and crystal-structure determination, *Acta Cryst.* **2015**, *A71*, 3–8.

8 M. J. Frisch, G. W. Trucks, H. B. Schlegel, G. E. Scuseria, M. A. Robb, J. R. Cheeseman, G. Scalmani, V. Barone, G. A. Petersson, H. Nakatsuji, X. Li, M. Caricato, A. V. Marenich, J. Bloino, B. G. Janesko, R. Gomperts, B. Mennucci, H. P. Hratchian, J. V. Ortiz, A. F. Izmaylov, J. L. Sonnenberg, D. Williams-Young, F. Ding, F. Lipparini, F. Egidi, J. Goings, B. Peng, A. Petrone, T. Henderson, D. Ranasinghe, V. G. Zakrzewski, J. Gao, N. Rega, G. Zheng, W. Liang, M. Hada, M. Ehara, K. Toyota, R. Fukuda, J. Hasegawa, M. Ishida, T. Nakajima, Y. Honda, O. Kitao, H. Nakai, T. Vreven, K. Throssell, J. A. Montgomery Jr., J. E. Peralta, F. Ogliaro, M. J. Bearpark, J. J. Heyd, E. N. Brothers, K. N. Kudin, V. N. Staroverov, T. A. Keith, R. Kobayashi, J. Normand, K. Raghavachari, A. P. Rendell, J. C. Burant, S. S. Iyengar, J. Tomasi, M. Cossi, J. M. Millam, M. Klene, C. Adamo, R. Cammi, J. W. Ochterski, R. L. Martin, K. Morokuma, O. Farkas, J. B. Foresman and D. J. Fox, **2016**.
